# Reenvisioning the De Mayo Reaction: A Boron‐Enabled Cycloaddition Approach

**DOI:** 10.1002/anie.202525317

**Published:** 2026-06-03

**Authors:** Neetu Sharma, Yanyao Liu, Partha Sarathi Hazra, Evan B. Piper, Ryan Van Hoveln, Michael Kevin Brown

**Affiliations:** ^1^ Department of Chemistry Indiana University Bloomington Indiana USA; ^2^ Department of Chemistry and Physics Indiana State University Terre Haute Indiana USA

**Keywords:** conformational lock, cycloaddition, De Mayo, energy transfer, photochemistry

## Abstract

The De Mayo reaction is an established process in chemical synthesis for the synthesis of 1,5‐dicarbonyls or cyclobutanols in selected cases. Herein, we present a reevisioned approach that utilizes alkenylboronates as surrogates for 1,3‐dicarbonyls. The process allows for delayed controlled synthesis of the traditional De Mayo products. Elaboration of the C─B bond in the product was also demonstrated. A key aspect of the study is the identification of a rigid oxy‐boracycle as a key intermediate to allow for bimolecular cycloaddition to occur. Mechanistic experiments are provided to clarify the importance of the chelate.

## Graphical abstract

An oxa‐boracycle conformational lock strategy enables photocatalytic [2+2] cycloaddition to afford densely substituted cyclobutylboronates. The conformational constraint suppresses triplet relaxation and promotes productive bimolecular reactivity. Divergent oxidation conditions provide access to either 1,5‐dicarbonyls or cyclobutanols, and the synthetic utility of the products was further demonstrated through C─B bond functionalization.

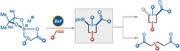

The De Mayo reaction is defined as the photochemical [2+2]‐cycloaddition of alkenes with β‐keto carbonyls to deliver 1,5‐dicarbonyls or cyclobutanols in select cases (Scheme 1A) [1]. The reaction has been investigated in a variety of contexts but some recent innovations include demonstrated use in several natural product syntheses [2, 3] as well use of visible light photosensitizers to enable the reaction under mild conditions [4, 5, 6, 7, 8]. We envisioned a conceptually novel approach that would involve controlled synthesis of either the 1,5‐dicarbonyl (**III**) or cyclobutanol (**II**) by taking advantage of the reactivity of alkenyl boronates (Scheme 1B). This would serve two key aspects. The first is that the cyclobutanol is a class of 4‐membered rings with broad application in chemical synthesis [9, 10, 11, 12]. In addition, when designing a synthetic scheme, it may be optimal to pass via the cyclobutanol, and only reveal the 1,5‐dicarbonyl at the desired stage. Our design hinges on [2+2]‐cycloaddition of an alkenyl boronate (**IV**) with an alkene to generate a cyclobutyl boronate [13, 14, 15, 16, 17, 18, 19, 20, 21, 22, 23, 24, 25, 26] (**V**). This intermediate can act as a masked cyclobutanol (**II**), by oxidation of the C─B bond or transformed to other structures (**VII**). Moreover, key to development of the strategy was the identification of a rigid oxa‐boracycle (**VI)** that was crucial to enable reactivity (Scheme 1B).

**SCHEME 1:**
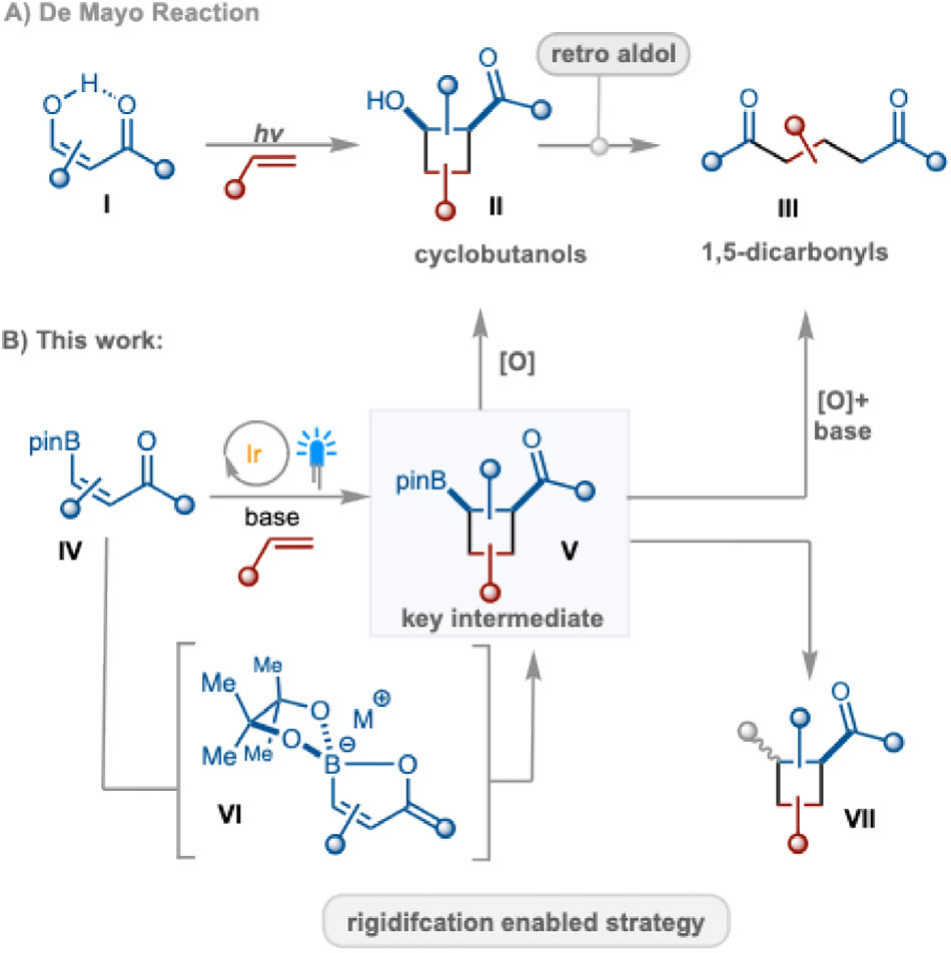
Advances in the de Mayo reaction.

We initiated our studies with cycloaddition of *E*‐β‐Bpin ester (prepared in one step, Scheme 2A) [27] with styrene in the presence of a variety of sensitizers under 450 nm irradiation in an integrated photoreactor (IPR) [28, 29, 30] (See Supporting Information for details). However, under all conditions attempted, no desired cycloaddition was observed. While cycloaddition of cinnamates is known [31, 32, 33], it is possible that steric demands of borylated substrate slows the rate of productive cycloaddition. We noted that the primary product was alkene isomerization of the β‐Bpin ester **4** to the *Z*‐isomer (*vida infra*). It was reasoned that the lack of reactivity might be due to relaxation of the triplet state prior to bimolecular cycloaddition [34, 35, 36, 37, 38]. To address this issue, the hypothesis was made that stronger coordination between the carbonyl oxygen and the boron atom would rigidify the structure and limit bond rotation, allow for bimolecular cycloaddition to occur [39, 40, 41]. To increase coordination strength, the β‐Bpin acrylamide **5** was prepared and tested (Scheme 2A). This type of compounds feature intramolecular dative coordination of amide to boron, therefore a tetrahedral geometry at boron which is in sharp contrast to its counterpart in ester compound **4** [42]. However, attempted cycloaddition again did not give rise to desired product and alkene isomerization was observed. To further increase the strength of the coordination, it was reasoned that a deprotonated amide would significantly increase the Lewis basicity. For this strategy, it was found that performing the cycloaddition of benzylamide **6** with styrene in the presence of NaO*t*‐Bu allowed formation of **8** in 42% yield (Scheme 2A).

**SCHEME 2:**
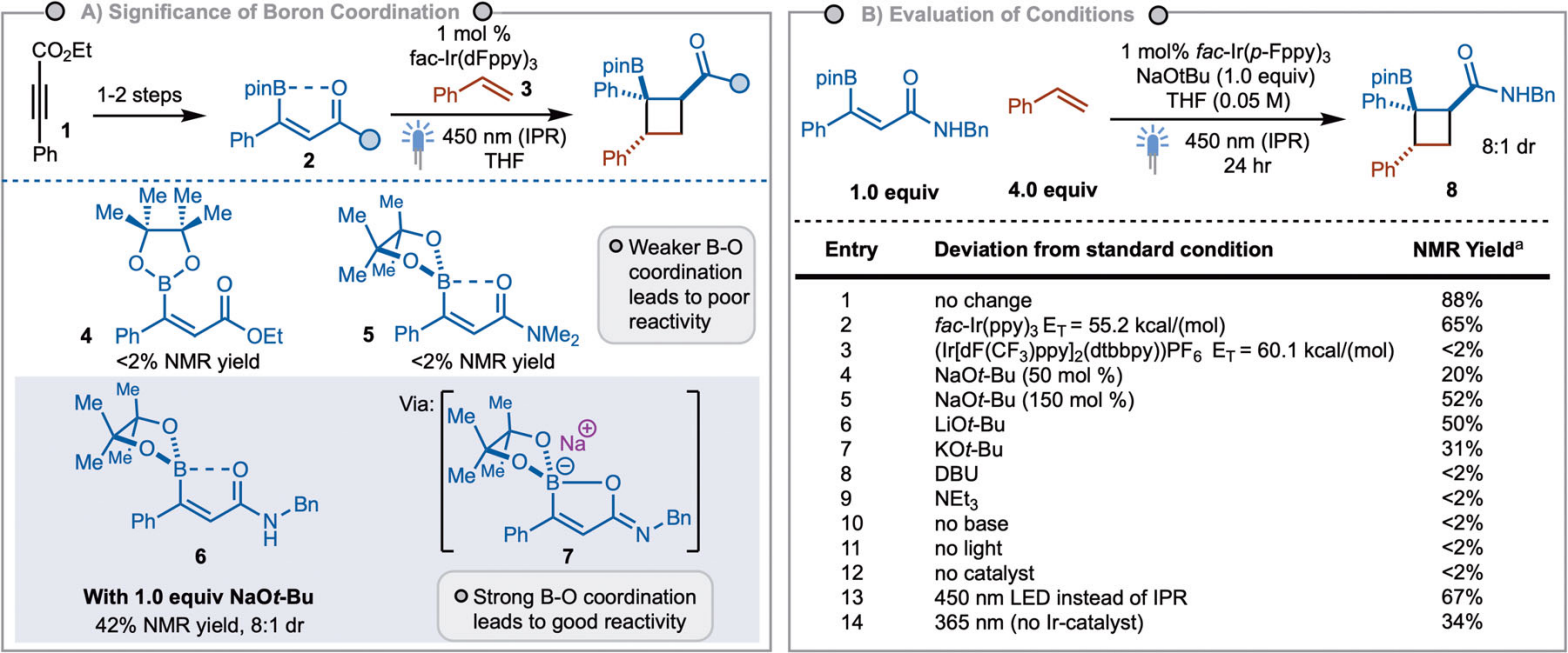
Initial investigations. ^a^ NMR yield determined by analysis of the unpurified reaction mixture by ^1^H NMR in the presence of an internal standard.

We hypothesize that, in the presence of base, substrate **6** forms a chelated intermediate **7**. Upon photoirradiation, this species generates a long‐lived triplet diradical, which subsequently undergoes a bimolecular reaction with styrene to afford the desired product.

Through evaluation of standard parameters, the reaction was optimized to the result shown in Scheme 2B, entry 1 (See Supporting Information for details). The key was the identification of a sensitizer with a suitable triplet energy. (*fac*‐Ir(*p‐*Fppy)_3_ *E*_T_ = 58.6 kcal/(mol) was found optimal compared to catalysts that have either higher energy, *fac*‐Ir(dFppy)_3_ *E*_T_ = 60.1 kcal/(mol), Scheme 2A or lower energy *fac*‐Ir(ppy)_3_ *E*_T_ = 55.2 kcal/(mol), Scheme 2B, entry 2). Use of cationic sensitizers did not work well, perhaps due to instability in the presence of strong base (Scheme 2B, entry 3). Reaction with higher or lower equivalents of base, or use of a different base, led to lower yields (Scheme 2B, entries 4–‐7). Organic bases like triethylamine or DBU were completely ineffective (Scheme 2B, entries 8 and 9). Finally, control experiments confirmed the requirement for base, light and catalyst (Scheme 2B, entries 10–12). Among the light sources tested, the integrated photoreactor (IPR) was selected due to its superior reaction efficiency (compare Scheme 2B, entries 1 and 13). Finally, product **8** was obtained upon direct irradiation of compound **7** with styrene under 365 nm light in the absence of a photocatalyst (Scheme 2A, entry 14). This result suggests the reaction proceeds via excited state intermediates, as opposed to pathways involving single electron transfer.

Under the optimal reaction conditions, the scope of the process was investigated (Scheme 3). First, with respect to the alkene component, various substituted styrenes functioned well. For example, substrates with electron‐donating (products **9**,**11**, **13**, **14,** and **20**) and electron‐withdrawing groups (products **10, 12,** and **15**) worked well. Sterically demanding groups also do not impede the reaction (products **9** and **10**). Within this series, tolerance to various functional groups, including boronic esters (product **17**), cyano (product **15**), and halides (products **10** and **12**), was tolerated. Various heterocycles also worked well, such as pyridine, thiophene, and furan (products **22–26**). These results are particularly significant owing to the central role of heterocycles in pharmaceutical and agrochemical development [43]. *Trans* β‐methyl styrene was also tolerated and led to the formation of product **16** with moderate yield. Oxetane alkenyl arene was used to generate spirocycle product **21** in good yield. These compounds have emerged as valuable scaffolds in medicinal chemistry, with increasing interest in their incorporation as saturated building blocks within chemical libraries for drug discovery and development [44]. Isoprene was tested and it worked well to generate vicinal fully substituted carbon; however, a mixture of regioisomers was observed (product **18**). An enyne was also attempted, and product **19** was formed, albeit in moderate yield.

**SCHEME 3:**
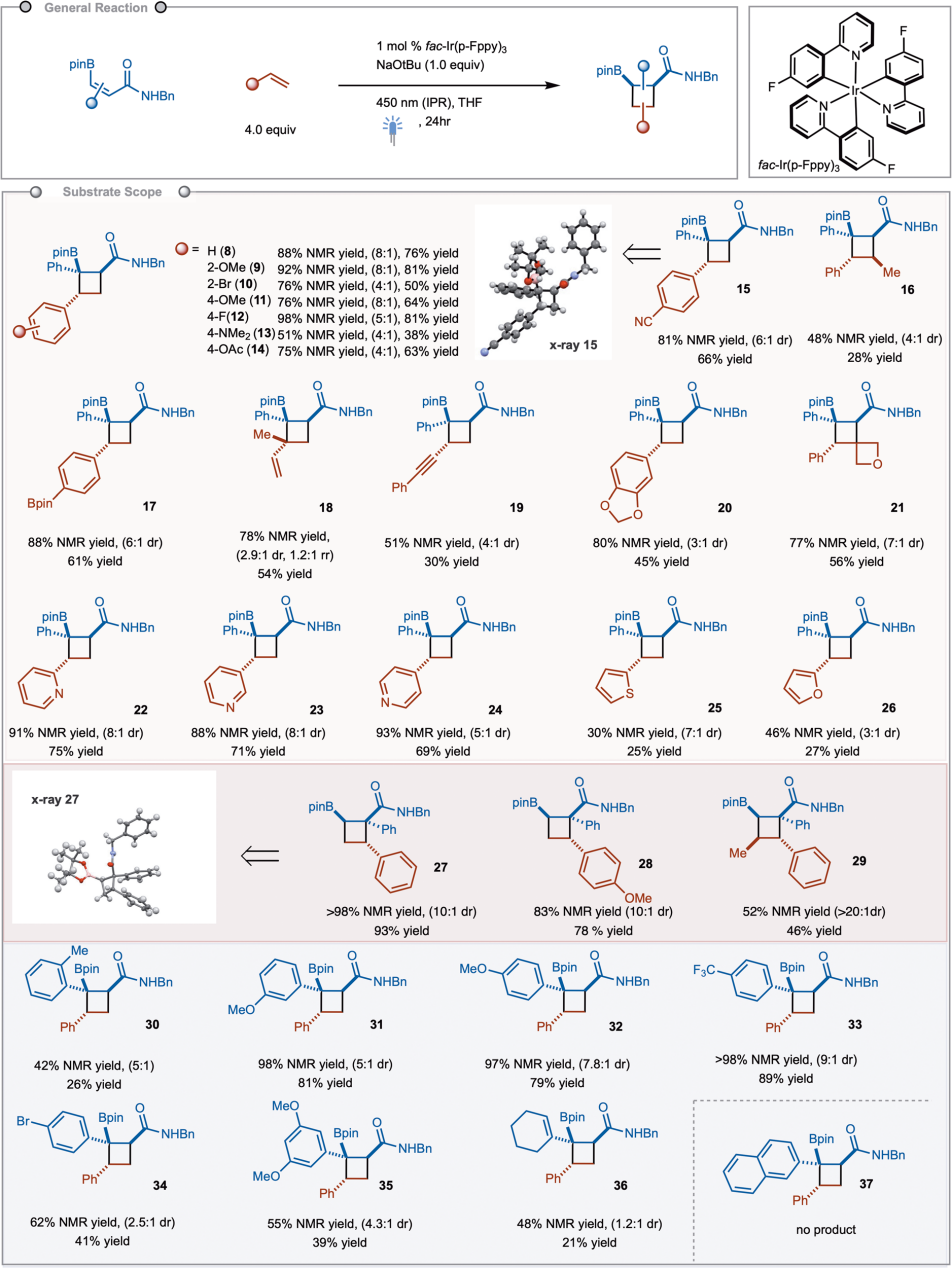
Substrate scope. NMR yields refers to yield determined by ^1^H NMR analysis of the unpurified reaction mixture of the borylated Cyclobutane product. Diastereomeric ratio (dr) determined of the unpurified reaction mixture by ^1^H NMR analysis. Isolated yield is of the major diastereomer except for **14**, **27**, **28**, **31**, and **35**, which were isolated as mixture of diastereoisomers. Product **18** isloated as mixture of diastereomers and regioisomers.

With respect to the alkenylboron component, the position of the aryl group can be moved to generate products **27–29** in good yield and high diastereoselectivity. In these cases, the initial bond formation occurs proximal to the Bpin unit to deliver the other regioisomer of product. This aspect greatly extends the scope of products than can be prepared with the method.

In addition, substitution with electron‐withdrawing (product **33**) and electron‐donating groups (products **30**, **31**, **32,** and **35**) worked well. Sterically demanding substitution was tolerated, albeit in reduced yield (product **30**). Finally, a diene was attempted and allowed for the formation of product **36** in 48% NMR yield.

A limitation was noted when naphthyl substitution was tested. It is likely that the photophysical properties of the substrate change, probably due to spin density of the triplet state is high on the naphthalene ring. In addition, unactivated alkenes, such as 1‐hexene, do not allow for product formation. In this case, capture of the triplet state is too slow relative to relaxation (See Supporting Information for details).

To demonstrate preparative utility, the reaction was conducted on a 5.0 mmol scale with no compromise in yield or selectivity (Scheme 4A). A key aspect of this study is the oxidation of the products to generate either the ring‐opened 1,5‐dicarbonyl or the cyclobutanol (Scheme 4B). It was found that treatment with NaBO_3_‐H_2_O resulted in the formation of 1,5‐dicarbonyl products **38** and **39**. On the other hand, oxidation with peroxide under buffered conditions allowed for the isolation of cyclobutanols **40** and **41**. Thus, either De Mayo products (1,5‐dicarbonyls) or interrupted De Mayo products (cyclobutanols) can be generated. It is worth noting that this strategy provides a platform for the synthesis of 1,5‐dicarbonyls and less stable β‐hydroxycyclobutanecarboxamides through a common intermediate. Finally, it should be noted that functionalization of the tertiary benzylic C−Bpin proved challenging.

**SCHEME 4:**
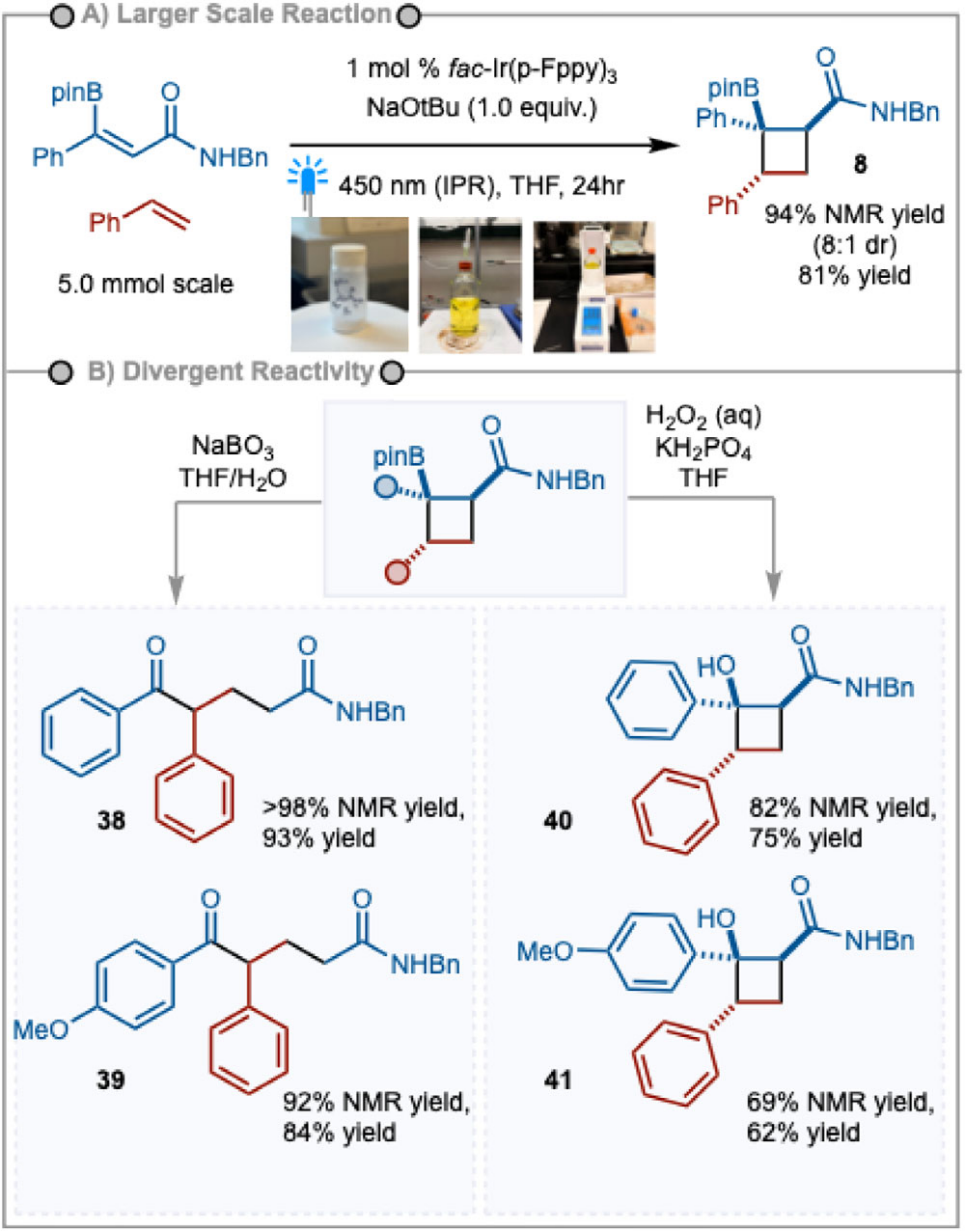
Utility of the reaction.

With the other regioisomer of the starting material, the reaction could also be run on a 1.0 mmol scale to provide product **27** in good yield and dr (Scheme 5A). The C─Bpin bond could be functionalized in several ways (Scheme 5B). First, photochemical coupling [45] with an aldehyde provided **43** as a 2:1 mixture of diastereomers in 41% isolated yield. In addition, Giese coupling [46] also worked well to generate **45** in good yield and 10:1 dr. Finally, oxidation could be carried out to generate cyclobutanol **44** in 81% yield.

**SCHEME 5:**
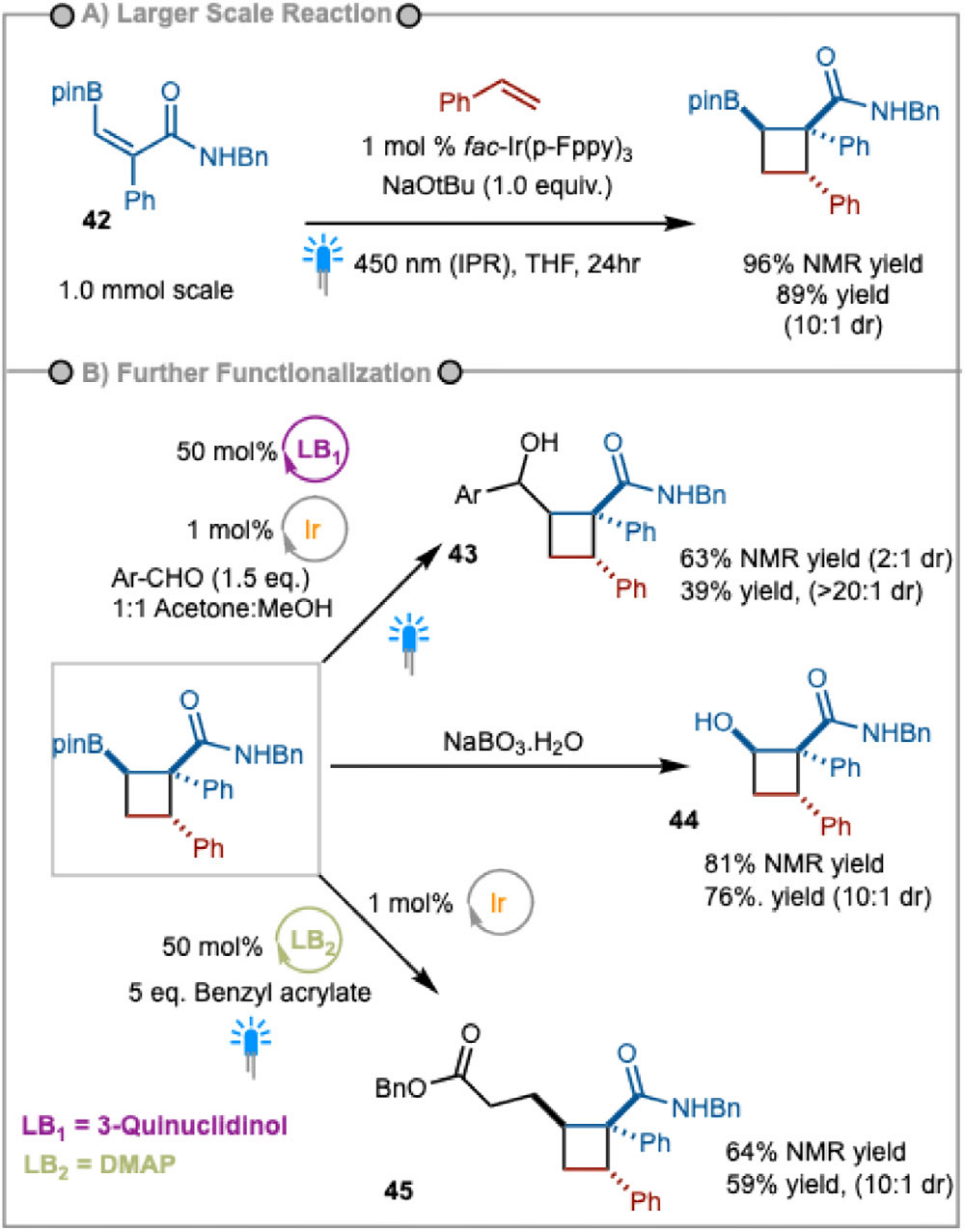
Further transformations.

Finally, the mechanism of the reaction was explored. In particular, the role of base was probed further (Scheme 6). Based on the design outlined in Scheme 1, the cyclic boronate rigidified the structure to allow for bimolecular cycloaddition. It was found that irradiation of the ester (**4**) or amide substrate (**5**) resulted in photoisomerization to the *Z*‐alkenes **46** and **47**, respectively. These experiments demonstrate that sensitization does occur, but that relaxation is more rapid than cycloaddition. In the case of cyclic boronate (generated **7** by treatment with NaO*t*‐Bu), photoisomerization was not observed, with dimer **48** being the only observable product formed (Scheme 5A). The reaction of **6‐Z** in the presence of base was also attempted and found to deliver product **8** in 40% yield, 8:1 dr. It appears that photocycloaddition to **6‐Z** can take place, but is less efficient (See Supporting Information for details). Triplet energies and geometries were also calculated for compounds **VII**‐**X** (corresponding to **4**‐**7**, respectively). As expected, geometry **VII** is twisted due to the weak coordination of the ester and boron atom. Moreover, with the amides **VII**‐**X**, the triplet energies are higher because bond rotation is more challenging due to the stronger coordination of the amide and boron atom (Scheme 6B). A Stern–Volmer experiment further confirms the quenching of the excited state of the photocatalyst with the cyclic boronate (Scheme 6C). Although at high styrene concentration, minor alkene involvement in excited‐state quenching cannot be fully excluded. The strength of the coordination was demonstrated by the evaluation of the ^11^B NMR spectra. By looking at the series of ester **4**, amide **5**, and deprotonated amide **7** (also compare with **6**, ^11^B NMR = 13.25 ppm, see Supporting Information), a decrease in the boron chemical shift was observed, indicating increased electron density on the boron atom (Scheme 6D). Based on the acquired data, a mechanism is proposed in Scheme 6E. Sensitization of deprotonated amide **XI** with the excited state of the Ir‐catalyst occurs to generate **XII** via dexter energy transfer [47, 48, 49, 50]. Addition of styrene results in the formation of **XIII**, which, undergoes intersystem crossing and bond formation. **XIV** is generated after workup.

**SCHEME 6:**
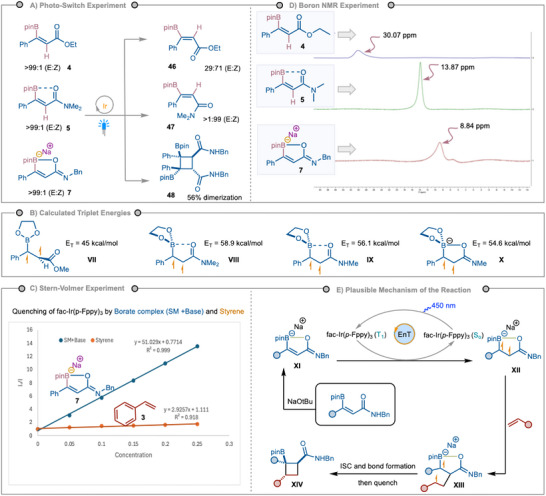
Mechanistic investigation.

In summary, we have described a method for the cycloaddition of β‐boryl acrylamides to prepare densely substituted cyclobutylboronates. A key aspect of the method is the use of base to drive the conformational lock to an oxa‐boracycle. The strategy limits unproductive relaxation of the triplet state by bond rotation such that bimolecular cycloaddition can occur. Finally, divergent oxidation conditions allow for the synthesis of either 1,5‐dicarbonyls or cyclobutanols. The synthetic utility of the C─B bond in the product was further showcased through downstream transformations. The strategy presented here is expected to have broader implications to drive otherwise challenging cycloadditions.

## Conflicts of Interest

The authors declare no conflicts of interest.

## Supporting information




**Supporting File 1**: anie72919‐sup‐0001‐SuppMat.pdf.

**Supporting File 2**: anie72919‐sup‐0002‐DataFile.zip.

## Data Availability

The data that support the findings of this study are available in the Supporting Information of this article.
