# Supplementary material for: Reenvisioning the De Mayo Reaction: A Boron‐Enabled Cycloaddition Approach
Source: Angew Chem Int Ed Engl. 2026 Jun 3;65(29):e25317. doi: 10.1002/anie.202525317 (PMC13360748; doi:10.1002/anie.202525317)
Supplement: Supplementary file 1 — Supporting File 1: anie72919‐sup‐0001‐SuppMat.pdf. [file ANIE-65-e25317-s001.pdf]

Reenvisioning the De Mayo Reaction: A Boron-Enabled Cycloaddition Approach

Neetu Sharma,[a] Yanyao Liu,[a] Partha Sarathi Hazra[a], Evan Piper,[a] Ryan Van Hoveln,[b] and M. Kevin Brown[a]*

[a] Department of Chemistry, Indiana University, 800 E. Kirkwood Ave. Bloomington, IN, 47401

[b]Department of Chemistry and physics, Indiana State University, 600 Chestnut Street, Terre Haute, IN 47809, USA

Supplementary Information – Table of contents

1) General Information ……….……………………………………..……………..... 02

2) Reagents, Catalysts and Photoreaction Setup……………………………………......….03

3) Substrate Synthesis……………………………………………………………...……05

4) Reaction Optimization and Control Experiments……………………………….....15

5) Experimental Procedure of photochemical reaction ……………………………....22

6) Final Product Characterization …………………………………………………....23

7) Large-Scale Reaction and Further Transformation.....…………………….……….....43

8) Further Transformation…………………………………………….……………....44

9) Mechanistic Investigation………………………………………………….….…...50

10) Computational Studies..58

11) X-ray structure …………………………………………………………….…....70

12) Spectra……………………………………………………………………...…..99

13) References……………………………………………………………..….......151

1. **General Information:**

NMR Spectroscopy

All ^{1}H NMR spectra were acquired at room temperature using either a Varian I400 (400 MHz), Varian I500 (500 MHz), Bruker 500 (500 MHz), or Varian I600 (600 MHz) spectrometer. Chemical shifts (δ) are expressed in parts per million (ppm) relative to tetramethylsilane (TMS), with the residual solvent peak in $CDCl_3$ (δ = 7.26 ppm) serving as the internal reference. Reported data include the chemical shift, multiplicity (s = singlet, d = doublet, t = triplet, q = quartet, br = broad, m = multiplet), coupling constants (J, Hz), and proton integration.

^{13}C NMR spectra were collected with full proton decoupling on either a Varian I500 or Bruker 500 spectrometer, both operating at 126 MHz. Chemical shifts were referenced to the $CDCl_3$ solvent peak at δ = 77.16 ppm. In addition, ^{19}F NMR spectra were recorded at 500 MHz on a Varian Bruker 500 spectrometer. Also, ^{11}B NMR spectra were acquired on a Bruker 500 (500MHz).

High-Resolution Mass Spectrometry (HRMS)

Accurate mass measurements were performed using electrospray ionization time-of-flight (ESI-TOF) on a Waters/Micromass LCT Classic instrument. The results are reported as m/z values with corresponding relative intensities.

Solvent Purification

Dichloromethane (DCM), tetrahydrofuran (THF), and dimethylformamide (DMF) were dried and purified by passing through two activated alumina columns under a positive argon pressure. Toluene (PhMe) was purified using a Grubbs-type solvent purification system by flowing through columns containing activated alumina and a Q5 catalyst.

Reaction Conditions

Unless otherwise specified, all reactions were carried out under a nitrogen atmosphere in glassware that had been pre-dried in an oven at 120 °C and flame-dried under vacuum. Anhydrous solvents were distilled, degassed, and handled using standard Schlenk line techniques. Reaction workups and purifications were performed in air with commercially available reagent-grade solvents.

Purification Procedures
Crude products were purified via flash column chromatography on ZEOprep 60/40–63 μm silica gel or using a Teledyne ISCO CombiFlash Rf 150 system equipped with pre-packed silica columns. Medium-pressure liquid chromatography (MPLC) was also performed on the same instrument as needed.

Photochemistry
Photochemical reactions were conducted using a Penn PhD Photoreactor M2, an integrated LED-based photoreactor system[i].

2. Reagents, Catalysts and Photoreaction setup

1,8-Diazabicyclo[5.4.0]undec-7-ene (DBU) was purchased from Oakwood and used as received.
1-Bromo-2-vinylbenzene was purchased from Ambeed and used as received.
1-Bromo-4-ethynylbenzene was purchased from Ambeed and used as received.
1-Ethynyl-2-methylbenzene was purchased from Ambeed and used as received.
1-Ethynyl-3-methoxybenzene was purchased from Combi-Blocks and used as received.
1-Ethynyl-3,5-dimethoxybenzene was purchased from Ambeed and used as received.
1-Ethynyl-4-(trifluoromethyl)benzene was purchased from Ambeed and used as received.
1-Ethynyl-4-methoxybenzene was purchased from Combi-Blocks and used as received.
1-Ethynylcyclohex-1-ene was purchased from Ambeed and used as received.
1-Fluoro-4-vinylbenzene was purchased from Oakwood and used as received.
1-Methoxy-2-vinylbenzene was purchased from Ambeed and used as received.
1-Methoxy-4-vinylbenzene was purchased from Ambeed and used as received.
2-Ethynylnaphthalene was purchased from Ambeed and used as received.
2-Vinylpyridine was purchased from Combi-Blocks and used as received.
2-Vinylthiophene was purchased from Ambeed and used as received.
3-Vinylpyridine was purchased from Combi-Blocks and used as received.
4,4,5,5-Tetramethyl-1,3,2-dioxaborolane was purchased from Oakwood and used as received.
4-Vinylbenzonitrile was purchased from Combi-Blocks and used as received.
4-Vinylphenyl acetate was purchased from Ambeed and used as received.
5-Vinylbenzo[d][1,3]dioxole was purchased from Combi-Blocks and used as received.
[Ir(dF(CF$_3$)ppy)$_2$(bpy)]PF$_6$ was purchased from Strem and used as received.
[Ir(dF(CF$_3$)ppy)$_2$(dtbbpy)]PF$_6$ was purchased from Strem and used as received.
[Ir(ppy)$_2$(dtbbpy)]PF$_6$ was purchased from Strem and used as received.
Benzylamine (NH$_2$Bn) was purchased from Sigma-Aldrich and used as received.
Dimethylamine (NHMe$_2$) was purchased from Sigma-Aldrich and used as received.
Ethyl 3-phenylpropiolate was purchased from Combi-Blocks and used as received.
Ethyl but-2-ynoate was purchased from Oakwood and used as received.
Ethyl chloroformate (ethyl carbonochloridate) was purchased from Sigma-Aldrich and used as received.
Ethynylcyclopropane was purchased from Ambeed and used as received.
(E)-Prop-1-en-1-ylbenzene (trans-β-methylstyrene) was purchased from TCI and used as received.
fac-Ir(dFppy)$_3$ was purchased from Strem and used as received.
fac-Ir(p-Fppy)$_3$ was purchased from Sigma-Aldrich and used as received or prepared according to the known literature procedure
fac-Ir(ppy)$_3$ was purchased from Strem and used as received.
Hydrogen peroxide (30%) was purchased from Fisher Scientific and used as received.
Isoprene was purchased from Alfa Aesar and used as received.
Lithium tert-butoxide was purchased from Strem and used as received.
Methyltriphenylphosphonium bromide (MePPh$_3$Br) was purchased from Ambeed and used as received.
n-Butyllithium solution in hexane (2.5 M) was purchased from Sigma-Aldrich and titrated in THF at 0 °C with sec-butanol and phenanthroline as an indicator prior to use.
N,N-Dimethyl-4-vinylaniline was purchased from Combi-Blocks and used as received.
Potassium dihydrogen phosphate (KH$_2$PO$_4$) was purchased from Fisher Scientific and used as received.

Potassium hydroxide was purchased from Macro and ground into powder before use.
Potassium tert-butoxide was purchased from Strem and used as received.
Sodium perborate tetrahydrate was purchased from Merck and used as received.
Sodium tert-butoxide was purchased from Strem and used as received.
Styrene was purchased from Sigma-Aldrich and used as received.
Triethylamine was purchased from Sigma-Aldrich and used as received.
Tributylphosphine was purchased from Sigma-Aldrich and used as received.
4-Vinylpyridine was purchased from Combi-Blocks and used as received.

Photoreaction setup:

Blue LED strips from Creative Lighting Solutions were wrapped 3–4 times around the inside of a 125 × 65 mm crystallization dish using their adhesive backing. The dish was placed in a water bath and equipped with a fan to maintain a stable temperature during irradiation. Reaction vials were positioned roughly 1 cm from the LEDs using a custom-made mold to ensure uniform and efficient light exposure (Figure **1A**).

The Penn PhD Photoreactor M2 is a compact, modular system designed for reproducible photochemical reactions. It allows control over light intensity, temperature, stirring, and time, with interchangeable LED wavelengths. Its reflective chamber ensures uniform 360° illumination, supporting various vial sizes (1–100 mL) (Figure **1B**).

Figure 1 Photochemical setup for [2+2] photocycloaddition

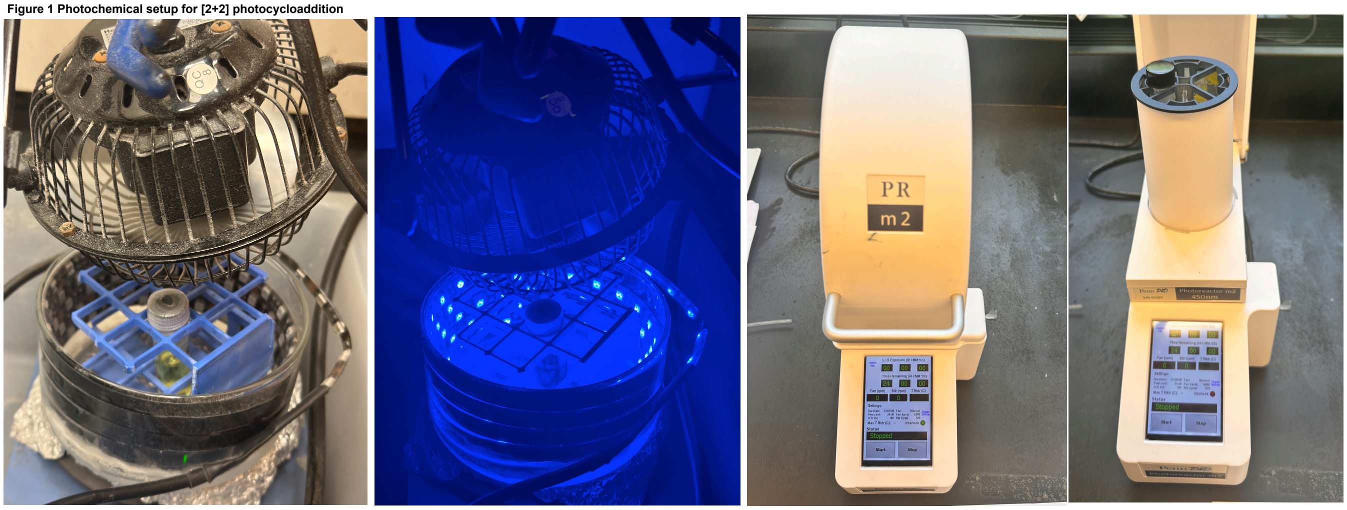

A) Double-density sapphire blue LEDs (12 V, 450 nm)

B) Penn PhD Photoreactor M2 integrated photoreactor

3. **Substrate Synthesis:**

General Procedure A:

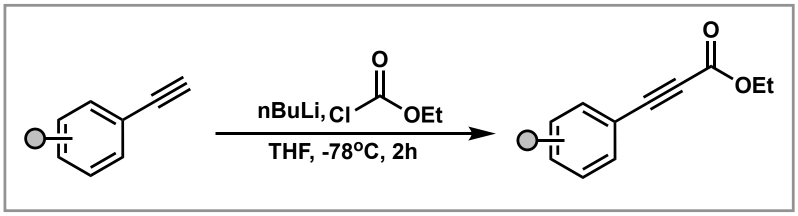

An oven-dried 100 mL round-bottom flask equipped with a magnetic stir bar was sealed with a septum and subjected to three cycles of evacuation and nitrogen backfilling using standard Schlenk line techniques. Anhydrous tetrahydrofuran (THF, 60 mL) was added under a nitrogen atmosphere, followed by the addition of the terminal alkyne (15 mmol, 1.0 equiv.). The reaction mixture was cooled to −78 °C using a dry ice/acetone bath.

n-Butyllithium (2.5 M in hexanes, 15 mmol, 1.0 equiv.) was then added dropwise via syringe at −78 °C. The reaction mixture was stirred at this temperature for 15 minutes. Subsequently, ethyl chloroformate (15 mmol, 1.0 equiv.) was added dropwise at −78 °C. The mixture was then allowed to warm to room temperature and stirred for an additional 2–3 hours.

Reaction progress was monitored by thin-layer chromatography (TLC). Upon completion (as indicated by the disappearance of starting material), the reaction was diluted by the addition of ethyl acetate (30 mL), followed by cold water (20 mL). The organic layer was separated, washed with brine (10 mL), and dried over anhydrous sodium sulfate. The solvent was removed under reduced pressure, and the crude product was purified by flash column chromatography (silica gel, 1:10 ethyl acetate:hexanes), affording the desired ester product.

Following alkynoate Esters SI-02, 04 and 05[ii], SI-03[iii], SI-06[iv], SI-07[v], SI-08[vi], SI-09[vii], and SI-11[viii] were prepared according to the general procedure A and the characterization are consistent with the known literature. SI-01 and SI-10 were purchased and used as received.

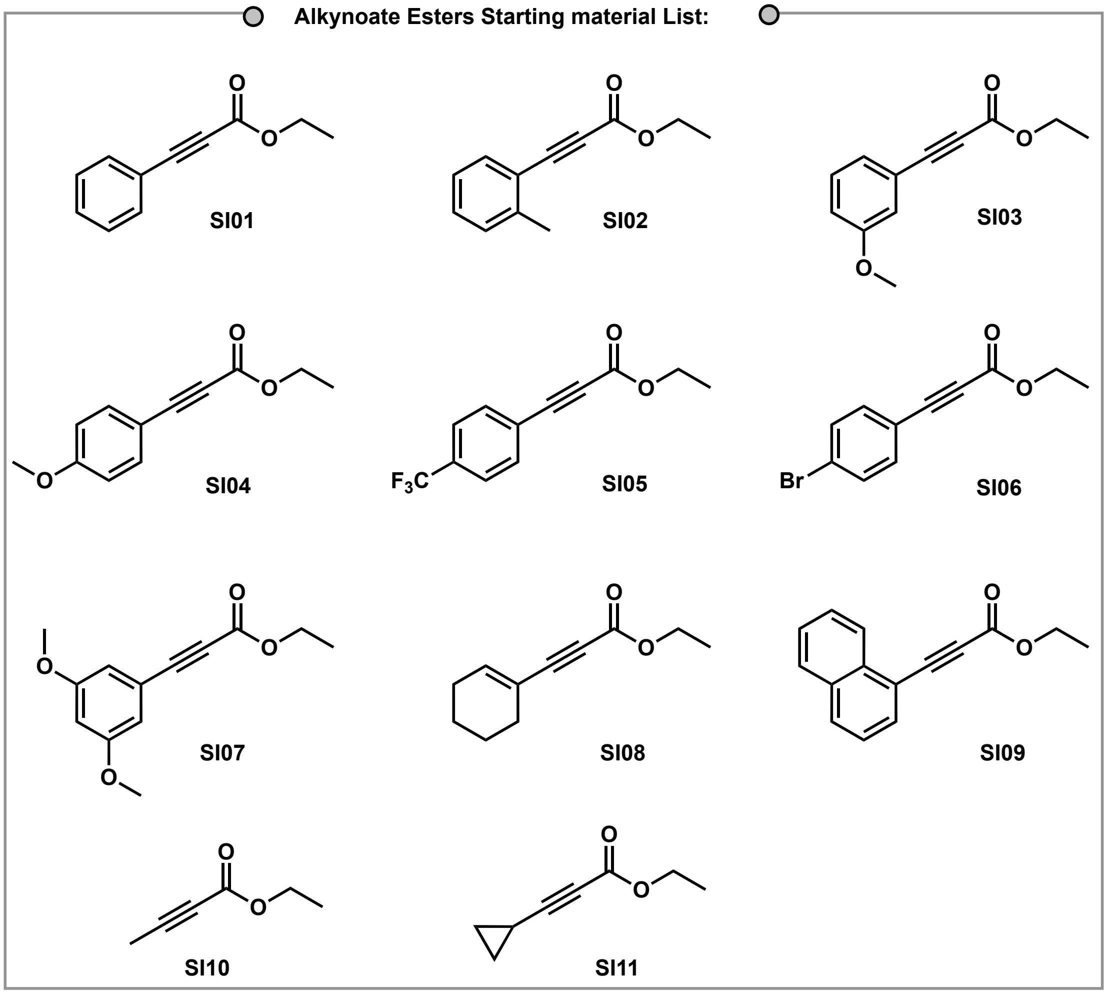

General Procedure B: Hydroboration of Alkynoate Esters to Access (E)-β-Boryl Acrylates

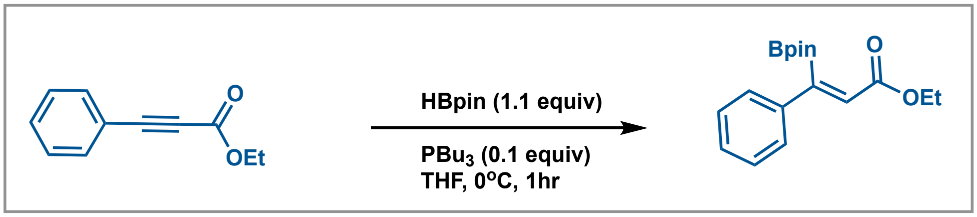

A flame-dried two-neck round-bottom flask equipped with a magnetic stir bar was sealed with a septum and subjected to three cycles of evacuation and nitrogen backfilling using standard Schlenk line technique. Anhydrous tetrahydrofuran (THF, 10 mL) was added under Nitrogen atmosphere, followed by the addition of ethyl 3-phenylpropiolate (871 mg, 5.0 mmol, 1.0 equiv.). Pinacolborane (0.8 ml, 5.5 mmol, 1.1 equiv.) was then added, and the reaction mixture was cooled to 0°C using an ice bath. Subsequently, tri-n-

butylphosphine (0.12 ml, 0.5 mmol, 0.1 equiv.) was added dropwise. The mixture was stirred at ambient temperature for 1-2 hour.[ix]

Reaction progress was monitored by thin-layer chromatography (TLC). Upon completion (disappearance of starting material), the solvent was removed under reduced pressure and the reaction mixture was further purified by flash column chromatography (silica gel, 1:10 ethyl acetate:hexanes), affording the desired (E)-β-boryl acrylate as a colorless oil 86% yield (1.120 g) after purification.

General Procedure C:

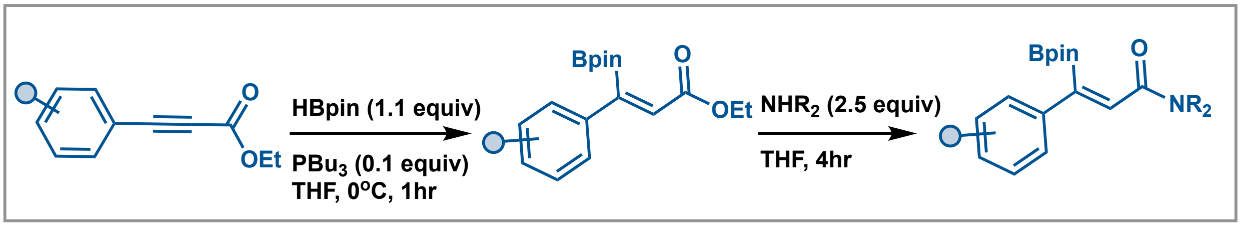

The desired (E)-β-boryl acrylate was obtained following General Procedure B. The crude reaction mixture containing the (E)-β-boryl acrylate (1.0 equiv.) was transferred to a 25 mL round-bottom flask equipped with a magnetic stir bar. THF (10 mL) and the corresponding amine (2.5 equiv.) were added under ambient atmosphere at room temperature. The reaction mixture was stirred at room temperature until complete consumption of the (E)-β-boryl acrylate, as monitored by TLC (typically 2-4 hours). Reaction progress was monitored by thin-layer chromatography (TLC).

Upon completion, the mixture was concentrated under reduced pressure and purified by flash column chromatography (20% ethyl acetate in dichloromethane) to yield the corresponding (E)-β-borylacrylamide as a white solid. In all cases, the ^{13}C NMR signal corresponding to the carbon directly bonded to the boron atom was not observed[x].

(E)-β-boryl Acrylamides List:

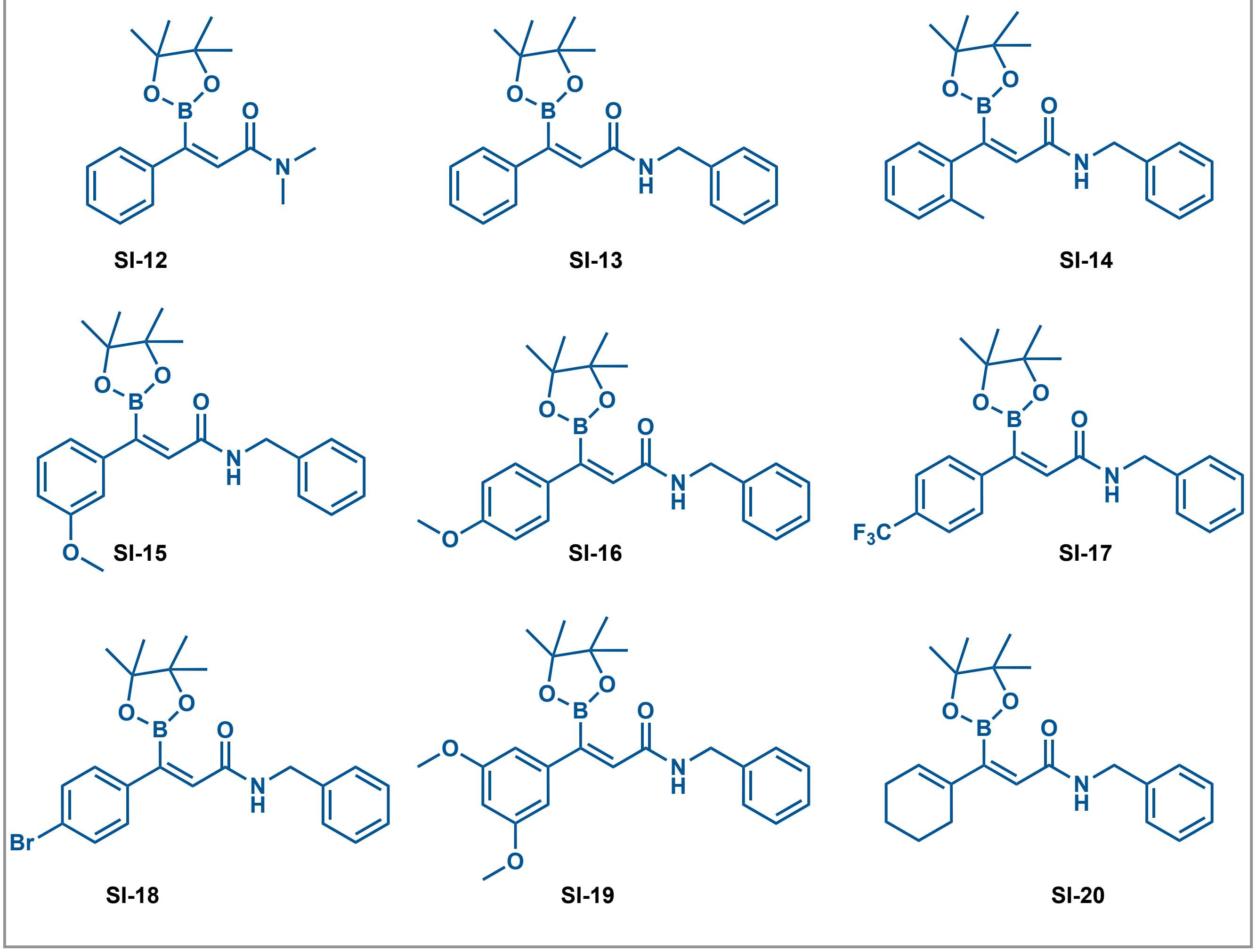

(*E*)-*N*-benzyl-3-phenyl-3-(4,4,5,5-tetramethyl-1,3,2-dioxaborolan-2-yl)acrylamide (SI-13)

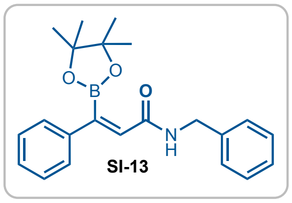

SI-13 was prepared in 20 mmol scale using the general procedure C (**3.3 gm, 91% yield, white solid**)

^{1}H NMR (500 MHz, $CDCl_3$): δ 9.90 (s, 1H), 7.67 – 7.52 (m, 2H), 7.38 – 7.29 (m, 3H), 7.22 (dd, *J* = 4.9, 1.9 Hz, 3H), 7.05 (dd, *J* = 6.6, 3.0 Hz, 2H), 6.53 (s, 1H), 4.46 – 3.96 (m, 2H), 1.15 (s, 12H).

^{13}C NMR (126 MHz, CDCl$_3$): δ 173.30, 137.72, 136.00, 128.98, 128.53, 128.22, 128.14, 127.86, 127.77, 120.77, 80.83, 45.03, 25.88. (Signal of carbon directly bonded to boron was not detected because of quadrupolar relaxation)

HRMS (ESI): Calc'd for $C_{22}H_{26}BNO_3Na[M+Na^+]$ 386.1898, found 386.1898

(*E*)-*N*-benzyl-3-(4,4,5,5-tetramethyl-1,3,2-dioxaborolan-2-yl)-3-(*o*-tolyl) acrylamide (SI-14)

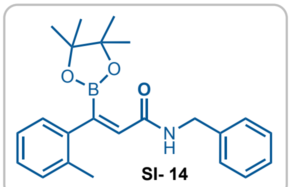

SI- 14

SI-14 was prepared in 3.5 mmol scale using the general procedure C (**1.2 gm, 89% yield, white solid**)

^{1}H NMR (500 MHz, CDCl$_3$): δ 8.00 (s, 1H), 7.38 (dd, *J* = 7.4, 1.4 Hz, 1H), 7.31 (ddt, *J* = 6.9, 5.1, 3.5 Hz, 3H), 7.23 (dd, *J* = 7.7, 1.8 Hz, 2H), 7.19 – 7.13 (m, 2H), 7.09 (td, *J* = 7.2, 2.1 Hz, 1H), 6.22 (s, 1H), 4.45 (d, *J* = 5.7 Hz, 2H), 2.36 (s, 3H), 1.11 (s, 11H).

^{13}C NMR (126 MHz, CDCl$_3$): δ 173.65, 139.20, 136.20, 135.13, 130.61, 129.29, 128.74, 128.59, 128.18, 127.69, 125.44, 123.21, 81.29, 45.86, 25.70, 21.29. (Signal of carbon directly bonded to boron was not detected because of quadrupolar relaxation)

HRMS (ESI): Calc'd for $C_{23}H_{28}BNO_3Na[M+Na^+]$ 400.2054, found 400.2056

(*E*)-*N*-benzyl-3-(3-methoxyphenyl)-3-(4,4,5,5-tetramethyl-1,3,2-dioxaborolan-2-yl)acrylamide (SI-15)

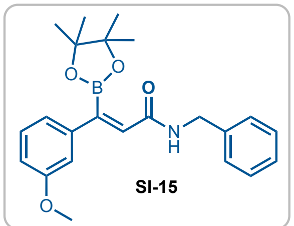

SI-15

SI-15 was prepared in 10 mmol scale using the general procedure C (**2.5 gm, 82% yield, white solid**)

^{1}H NMR (500 MHz, CDCl$_3$): δ 9.72 (s, 1H), 7.28 (dd, *J* = 2.7, 1.4 Hz, 1H), 7.26 – 7.18 (m, 4H), 7.15 – 7.05 (m, 3H), 6.89 (ddd, *J* = 8.2, 2.7, 1.0 Hz, 1H), 6.51 (s, 1H), 4.27 (s, 2H), 3.76 (s, 3H), 1.57 (s, 0H), 1.16 (s, 11H).

^{13}C NMR (126 MHz, CDCl$_3$): δ 173.22, 159.42, 139.14, 135.99, 129.13, 128.54, 128.16, 127.78, 121.08, 119.97, 115.04, 113.02, 80.82, 55.02, 45.11, 25.90. (Signal of carbon directly bonded to boron was not detected because of quadrupolar relaxation)

HRMS (ESI): Calc'd for $C_{23}H_{28}BNO_4Na[M+Na^+]$ 416.2003, found 416.2005

(*E*)-*N*-benzyl-3-(4-methoxyphenyl)-3-(4,4,5,5-tetramethyl-1,3,2-dioxaborolan-2-yl)acrylamide (SI-16)

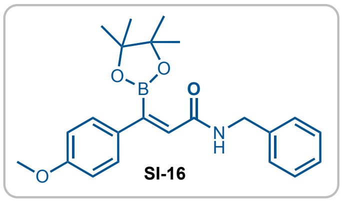

SI-16 was prepared in 10.0 mmol scale using the general procedure C (**2.8 gm, 93% yield, white solid**)

^{1}H NMR (500 MHz, CDCl$_3$): δ 7.56 (d, *J* = 8.2 Hz, 2H), 7.24 (dd, *J* = 4.9, 1.8 Hz, 3H), 7.09 (dd, *J* = 6.7, 3.0 Hz, 2H), 6.94 – 6.72 (m, 2H), 6.39 (s, 1H), 4.25 (s, 2H), 3.82 (s, 3H), 1.19 (s, 11H).

^{13}C NMR (126 MHz, CDCl$_3$): δ 174.01, 161.03, 136.78, 130.81, 130.17, 129.03, 128.61, 128.23, 118.99, 114.08, 81.26, 55.63, 45.54, 26.58. (Signal of carbon directly bonded to boron was not detected because of quadrupolar relaxation)

HRMS (ESI): Calc'd for $C_{23}H_{28}BNO_4Na$[M+Na$^+$] 416.2003, found 416.2006

(*E*)-*N*-benzyl-3-(4,4,5,5-tetramethyl-1,3,2-dioxaborolan-2-yl)-3-(4-(trifluoromethyl)-phenyl)acrylamide (SI-17)

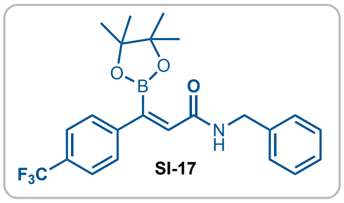

SI-17 was prepared in 5 mmol scale using the general procedure C (**1.6 gm, 74 % yield, white solid**)

^{1}H NMR (500 MHz, CDCl$_3$): δ 10.27 (s, 0H), 7.65 (d, *J* = 8.0 Hz, 1H), 7.58 (d, *J* = 8.1 Hz, 1H), 7.29 – 7.24 (m, 2H), 7.04 (dd, *J* = 7.2, 2.5 Hz, 1H), 6.60 (s, 0H), 4.22 (s, 1H), 1.17 (s, 6H).

^{13}C NMR (126 MHz, CDCl$_3$): δ 173.11, 141.04, 135.22, 130.82 (d, *J* = 32.5 Hz), 128.69, 128.11, 127.86, 127.73, 125.21 (q, *J* = 3.7 Hz), 123.85 (d, *J* = 272.1 Hz), 122.46, 81.05, 45.22, 25.79. (Signal of carbon directly bonded to boron was not detected because of quadrupolar relaxation)

^{19}F NMR (471 MHz, CDCl$_3$): δ -62.13.

HRMS (ESI): Calc'd for $C_{23}H_{25}BNO_3F_3$ Na[M+Na$^+$]454.1771, found 454.1764

(*E*)-*N*-benzyl-3-(4-bromophenyl)-3-(4,4,5,5-tetramethyl-1,3,2-dioxaborolan-2-yl)acrylamide (SI-18)

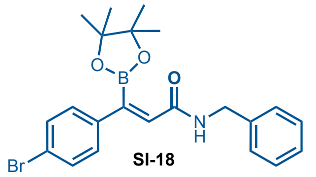

SI-18

SI-18 was prepared in 5.0 mmol scale using the general procedure C (**1.05 gm, 70% yield, white solid**)

^{1}H NMR (500 MHz, CDCl$_3$): δ 10.16 – 9.63 (m, 1H), 7.48 – 7.32 (m, 4H), 7.24 (dd, *J* = 4.1, 2.6 Hz, 3H), 7.05 (dd, *J* = 6.7, 3.0 Hz, 2H), 6.46 (s, 1H), 4.20 (s, 2H), 1.14 (s, 12H).

^{13}C NMR (126 MHz, CDCl$_3$): δ 173.25, 136.42, 135.63, 131.52, 129.29, 128.64, 128.60, 128.32, 128.02, 128.00, 127.95, 127.88, 123.45, 120.99, 80.90, 45.15, 25.93, 25.89. (Signal of carbon directly bonded to boron was not detected because of quadrupolar relaxation)

HRMS (ESI): Calc'd for $C_{22}H_{26}BBrNO_3$[M+H$^+$] 444.1163, found 444.1151

(*E*)-*N*-benzyl-3-(3,5-dimethoxyphenyl)-3-(4,4,5,5-tetramethyl-1,3,2-dioxaborolan-2-yl)acrylamide (SI-19)

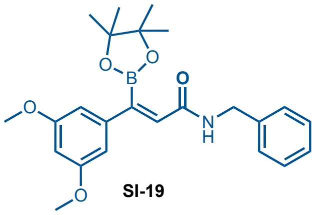

SI-19

SI-19 was prepared in 10.0 mmol scale using the general procedure C (**2.9 gm, 69 % yield, white solid**)

^{1}H NMR (500 MHz, CDCl$_3$): δ 10.38 (t, *J* = 6.0 Hz, 0H), 7.36 (d, *J* = 1.9 Hz, 1H), 7.19 (dd, *J* = 5.0, 1.9 Hz, 1H), 7.02 (dt, *J* = 7.3, 3.9 Hz, 1H), 6.75 (d, *J* = 8.3 Hz, 0H), 6.50 (s, 1H), 4.18 (s, 1H), 3.88 (s, 2H), 3.80 (s, 1H), 1.15 (s, 6H).

^{13}C NMR (126 MHz, CDCl$_3$): δ 173.71, 149.95, 148.46, 135.99, 130.61, 128.42, 127.89, 127.64, 120.73, 118.85, 111.46, 110.56, 80.56, 55.67, 55.57, 44.93, 26.06. (Signal of carbon directly bonded to boron was not detected because of quadrupolar relaxation)

HRMS (ESI): Calc'd for $C_{24}H_{30}BNO_5Na$[M+Na$^+$]446.3102, found 446.2102

(*E*)-*N*-benzyl-3-(cyclohex-1-en-1-yl)-3-(4,4,5,5-tetramethyl-1,3,2-dioxaborolan-2-yl)acrylamide

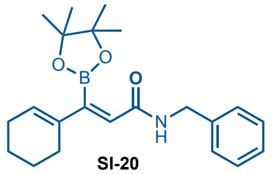

SI-20

SI-20 was prepared in 2.5 mmol scale using the general procedure C (**740 mg, 80% yield, white solid**)

^{1}H NMR (500 MHz, CDCl$_3$): δ 9.00 (s, 1H), 7.46 – 7.07 (m, 6H), 6.68 (s, 1H), 6.09 (s, 1H), 4.42 (d, J = 5.8 Hz, 2H), 2.26 (dt, J = 9.0, 4.3 Hz, 2H), 2.22 – 2.10 (m, 2H), 1.75 – 1.66 (m, 2H), 1.66 – 1.55 (m, 2H), 1.22 (s, 12H).

^{13}C NMR (126 MHz, CDCl$_3$): δ 173.24, 136.87, 136.62, 135.68, 128.54, 128.01, 127.67, 117.31, 80.79, 44.80, 26.31, 26.28, 25.95, 22.48, 21.86. (Signal of carbon directly bonded to boron was not detected because of quadrupolar relaxation)

HRMS (ESI): Calc'd for $C_{22}H_{30}BNO_3Na$[M+Na$^+$] 390.2211, found 390.2213

General Procedure D:

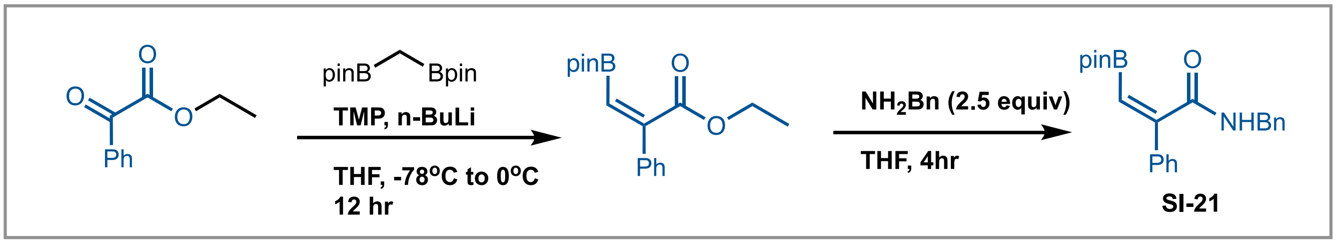

A 50 mL round-bottom flask equipped with a magnetic stir bar was flame-dried under vacuum, allowed to cool to room temperature under vacuum, and backfilled with N_2. Under a nitrogen atmosphere, 2,2,6,6-tetramethylpiperidine (1.1 mL, 6.5 mmol, 1.3 equiv) and THF (12 mL) were added to the flask. The reaction mixture was cooled to −78 °C using a dry ice/acetone bath, and n-BuLi (2.2 M in hexanes, 3.0 mL, 6.5 mmol, 1.3 equiv) was added dropwise, resulting in the formation of a yellow solution. The reaction mixture was warmed to 0 °C and stirred for 1 h.

A solution of bis(4,4,5,5-tetramethyl-1,3,2-dioxaborolan-2-yl) methane (1.6 g, 6.0 mmol, 1.2 equiv) in THF (5 mL) was then added dropwise at 0 °C, and the reaction mixture was stirred for 5 min. The flask was subsequently cooled back to −78 °C, during which formation of a white precipitate was observed. A solution of ketone (5.0 mmol, 1.0 equiv) in THF (5 mL) was added dropwise, and the reaction mixture was stirred overnight while warming gradually to room temperature.

Upon completion, the reaction was quenched with DI H_2O (15 mL). The aqueous layer was extracted with Et2O (3 × 10 mL), and the combined organic extracts were washed with brine, dried over MgSO4, filtered, and concentrated under reduced pressure.

The crude reaction mixture containing the (E)-β-boryl acrylate (1.0 equiv.) was transferred to a 25 mL round-bottom flask equipped with a magnetic stir bar. THF (10 mL) and the corresponding amine (2.0 equiv.) were added under ambient atmosphere at room temperature. The reaction mixture was stirred at room temperature until complete consumption of the (E)-β-boryl acrylate, as monitored by TLC (typically 2-4 hours). Reaction progress was monitored by thin-layer chromatography (TLC).

Upon completion, the mixture was concentrated under reduced pressure and purified by flash column chromatography (20% ethyl acetate in dichloromethane) to yield the corresponding (E)-β-borylacrylamide as a white solid.

^{1}H NMR (500 MHz, $CDCl_3$): δ 7.40 – 7.35 (m, 5H), 7.34 (d, J = 0.9 Hz, 1H), 7.32 – 7.28 (m, 4H), 6.72 (d, J = 2.7 Hz, 1H), 6.30 (t, J = 5.5 Hz, 1H), 4.64 (d, J = 5.7 Hz, 2H), 1.36 (s, 12H).

^{13}C NMR (126 MHz, $CDCl_3$): δ 170.98, 143.60, 136.13, 135.28, 128.92, 128.78, 128.43, 127.94, 127.88, 127.76, 81.73, 45.33, 24.94. (Signal of carbon directly bonded to boron was not detected because of quadrupolar relaxation)

HRMS (**ESI**): Calc'd for $C_{22}H_{26}BNO_3[MH^+]$ 364.2078, found 364.2073

General Procedure E:

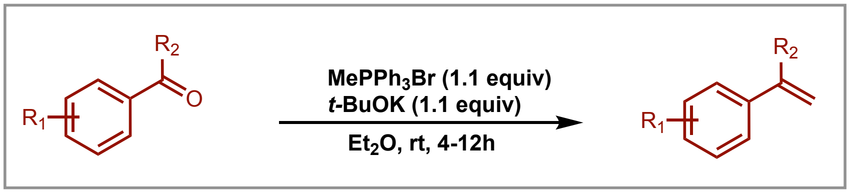

An oven-dried 250 mL round-bottom flask equipped with a magnetic stir bar was cooled under vacuum. Potassium tert-butoxide (KO^{t}Bu, 1.23 g, 11.0 mmol, 1.10 equiv.) was added, and the flask was sealed with a septum. The system was evacuated and backfilled with nitrogen three times before adding anhydrous tetrahydrofuran (THF, 50 mL). The suspension was placed in a water bath at room temperature, and methyltriphenylphosphonium bromide ($MePPh_3Br$, 3.93 g, 11.0 mmol, 1.10 equiv.) was added portion-wise. The mixture was stirred at room temperature for 30 minutes, then the aldehyde or ketone (10 mmol, 1.0 equiv.) was introduced. The reaction was allowed to stir overnight. Upon completion, the solvent was removed under reduced pressure, and the crude product was precipitated by adding pentane (50 mL) followed by sonication. The resulting suspension was filtered through a silica gel pad and washed with hexane/diethyl ether (ratio determined by TLC) to afford the desired styrene product[xi].

Alkene Scope List:

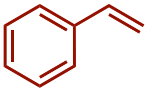

SI-22

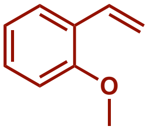

SI-23

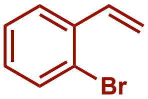

SI-24

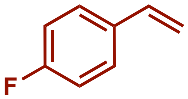

SI-25

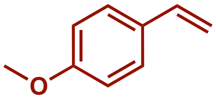

SI-26

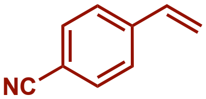

SI-27

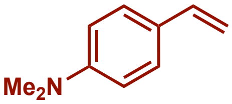

SI-28

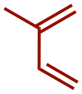

SI-29

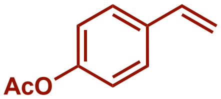

SI-30

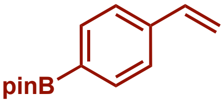

SI-31

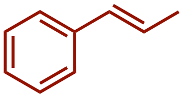

SI-32

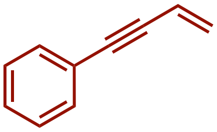

SI-33

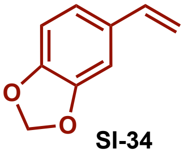

SI-34

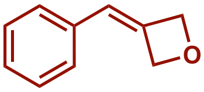

SI-35

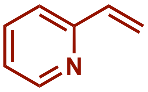

SI-36

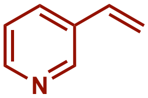

SI-37

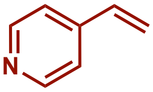

SI-38

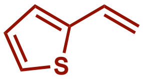

SI-39

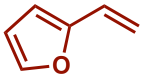

SI-40

4. Optimization Studies

General procedure 4a:

An oven-dried 2-dram vial equipped with a magnetic stir bar was brought into a N_2-filled glovebox and charged with [Ir]-Photocatalyst (1 mol%), ethyl (*E*)-3-phenyl-3-(4,4,5,5-tetramethyl-1,3,2-dioxaborolan-2-yl) acrylate (30.0 mg, 0.100 mmol, 1.00 equiv.). The vial(s) was sealed with a septum and removed from the glovebox. THF (0.05 M, 2ml) was added to the reaction vial via syringe, followed by the addition of styrene (46 µl, 0.400 mmol, 4 equiv.). The septum on the reaction vial was quickly replaced by a screw cap and the reaction mixture then was irradiated in 450 nm light source for 24 hours at room temperature with continuous stirring. Upon completion, the volatiles were removed under reduced pressure. The residue was dissolved in $CDCl_3$, CH_2Br_2 was added as an internal standard, and the crude reaction mixture was analyzed by ^{1}H NMR spectroscopy.

A) Optimization Table

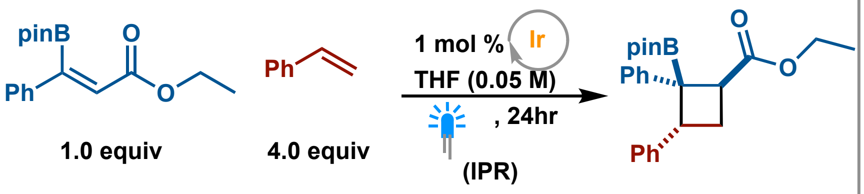

entry	sensitizer	E_T(kcal/mol)	yield[a]
1	*fac*-Ir(*p*-Fppy)$_3$	58.6	<5%
2	*fac*-Ir(dFppy)$_3$	60.1	<5%
3	(Ir[dF(CF$_3$)ppy]$_2$(dtbbpy))PF$_6$	60.1	<5%
4	(Ir[dF(CF$_3$)ppy]$_2$(bpy))PF$_6$	60.4	<5%

Condition: The reactions were performed on a 0.1mmol scale, with 4 equiv of styrene, 1 mol% photocatalyst and 2 ml THF (0.05M).

Note: The isomerization of SM and homocoupling of styrene was only observed

General procedure 4b:

An oven-dried 2-dram vial equipped with a stir bar was brought into a N_2-filled glovebox and charged with [Ir]-Photocatalyst (1 mol%), (*E*)-*N*, *N*-dimethyl-3-phenyl-3-(4,4,5,5-tetramethyl-1,3,2-dioxaborolan-2-yl)acrylamide (30.1 mg, 0.100 mmol, 1.00 equiv.). The vial(s) was sealed with a septum and removed from the glovebox. THF (0.05 M, 2ml) was added to the reaction vial via syringe, followed by the addition of styrene (46 µl, 0.400 mmol, 4 equiv.). The septum on the reaction vial was quickly replaced by a screw cap and the reaction mixture then was irradiated in 450 nm light source for 24 hours at room temperature with continuous stirring. Upon completion, the volatiles were removed under reduced pressure. The residue was dissolved in $CDCl_3$, CH_2Br_2 was added as an internal standard, and the crude reaction mixture was analyzed by ^{1}H NMR spectroscopy.

B) Optimization Table

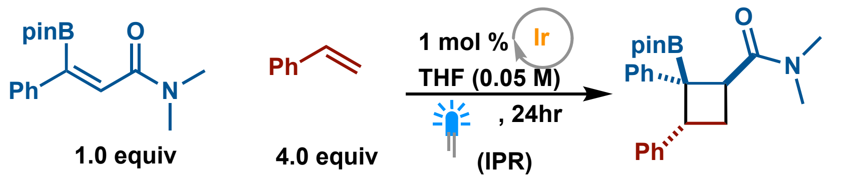

entry	sensitizer	E_T(kcal/mol)	yield[a]
1	*fac*-Ir(*p*-Fppy)$_3$	58.6	<2%
2	*fac*-Ir(dFppy)$_3$	60.1	<2%
3	(Ir[dF(CF$_3$)ppy]$_2$(dtbbpy))PF$_6$	60.1	<2%
4	(Ir[dF(CF$_3$)ppy]$_2$(bpy))PF$_6$	60.4	<2%

Condition: The reactions were performed on a 0.1mmol scale, with 4 equiv of styrene, 1 mol% photocatalyst and 2 ml THF (0.05M).

Note: The isomerization of SM and homocoupling of styrene was only observed

General procedure 4c for optimization:

An oven-dried 2-dram vial equipped with a magnetic stir bar was charged with (*E*)-*N*-benzyl-3-phenyl-3-(4,4,5,5-tetramethyl-1,3,2-dioxaborolan-2-yl) acrylamide (1.0 equiv.) and [Ir]-Photocatalyst (1 mol%). The vial was transferred into a nitrogen-filled glovebox, and base (xx equiv.) was added. The vial was sealed with a septum and removed from the glovebox. Solvent was added to the reaction vial via syringe under nitrogen. The reaction mixture was stirred at room temperature under nitrogen for 30 min. Styrene (46 µl, 0.400 mmol, 4 equiv.) was then added dropwise via syringe. The septum was quickly replaced with a screw cap, and the reaction mixture was irradiated with 450 nm light (light source: Integrated Photoreactor, IPR) at room temperature for 24 h with continuous stirring. Upon completion, the reaction was quenched with saturated aqueous NH_4Cl (3.0 mL) and extracted with EtOAc (3 × 3.0 mL). The combined organic layers were washed with brine (3.0 mL), dried over anhydrous Na_2SO_4, filtered, and concentrated under reduced pressure. CH_2Br_2 was added as an internal standard, and a small aliquot of the crude mixture was analyzed by ^{1}H NMR spectroscopy to determine the NMR yield and diastereomeric ratio (dr).

Table SI-1 Photocatalyst Screening:

An oven-dried 2-dram vial equipped with a magnetic stir bar was charged with (*E*)-*N*-benzyl-3-phenyl-3-(4,4,5,5-tetramethyl-1,3,2-dioxaborolan-2-yl) acrylamide (36.3 mg, 0.2 mmol, 1.0 equiv.) and *Photocatalyst*. The vial was transferred into a nitrogen-filled glovebox, and sodium *tert*-butoxide (9.61 mg, 0.2 mmol, 1.0 equiv.) was added. The vial was sealed with a septum and removed from the glovebox. Dry THF (2.0 mL, 0.05 M) was added under nitrogen. The reaction mixture was stirred at room temperature under nitrogen for 30 min. Styrene (46 µl, 0.400 mmol, 4 equiv.) was then added dropwise via syringe. The septum was quickly replaced with a screw cap, and the reaction mixture was irradiated with 450 nm light (light source: Integrated Photoreactor, IPR) at room temperature for 24 h with continuous stirring. Upon completion, the reaction was quenched with saturated aqueous NH_4Cl (3.0 mL) and extracted with EtOAc (3 × 3.0 mL). The combined organic layers were washed with brine (3.0 mL), dried over anhydrous Na_2SO_4, filtered, and concentrated under reduced pressure. CH_2Br_2 was added as an internal standard, and a small aliquot of the crude mixture was analyzed by ^1H NMR spectroscopy to determine the NMR yield and diastereomeric ratio (dr).

C) Optimization Table

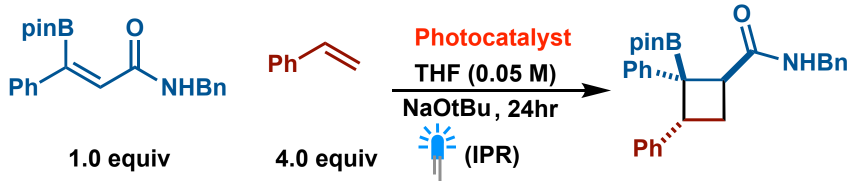

entry	sensitizer	E_T(kcal/mol)	yield[a]
1	[Ir (ppy)$_2$(dtbbpy)]PF$_6$	49.2	20%
2	*fac*-Ir(ppy)$_3$	55.2	65%
3	*fac*-Ir(*p*-Fppy)$_3$	58.6	88%
4	*fac*-Ir(dFppy)$_3$	60.1	42%
5	(Ir[dF(CF$_3$)ppy]$_2$(dtbbpy))PF$_6$	60.1	<2%
6	(Ir[dF(CF$_3$)ppy]$_2$(bpy))PF$_6$	60.4	17%
7	ITX	62	<2%
8	4CZIPN	60	<2%
9	3DPA2FBN	53	<2%

Condition: The reactions were performed on a 0.1mmol scale, with 4 equiv of styrene, Ir-catalyst (entry 1-6) has taken 1 mol% and organophotocatalyst (entry 7-9) has taken 10 mol%, and 2 ml THF (0.05M).

Table SI-2 Base Screening:

An oven-dried 2-dram vial equipped with a magnetic stir bar was charged with (*E*)-*N*-benzyl-3-phenyl-3-(4,4,5,5-tetramethyl-1,3,2-dioxaborolan-2-yl) acrylamide (36.3 mg, 0.2 mmol, 1.0 equiv.) and *fac*-Ir(p-Fppy)$_3$ (0.7 mg, 1 mol%, 0.01 equiv.). The vial was transferred into a nitrogen-filled glovebox, and *base (xx mol%)*. was added. The vial was sealed with a septum and removed from the glovebox. Dry THF (2.0 mL, 0.05 M) was added under nitrogen. The reaction mixture was stirred at room temperature under nitrogen for 30 min. Styrene (46 µl, 0.400 mmol, 4 equiv.) was then added dropwise via syringe. The septum was quickly replaced with a screw cap, and the reaction mixture was irradiated with 450 nm light (light source: Integrated Photoreactor, IPR) at room temperature for 24 h with continuous stirring. Upon completion, the reaction was quenched with saturated aqueous NH_4Cl (3.0 mL) and extracted with EtOAc (3 × 3.0 mL). The combined organic layers were washed with brine (3.0 mL), dried over anhydrous Na_2SO_4, filtered, and concentrated under reduced pressure. CH_2Br_2 was added as an internal standard, and a small aliquot of the crude mixture was analyzed by ^1H NMR spectroscopy to determine the NMR yield and diastereomeric ratio (dr).

D) Optimization Table

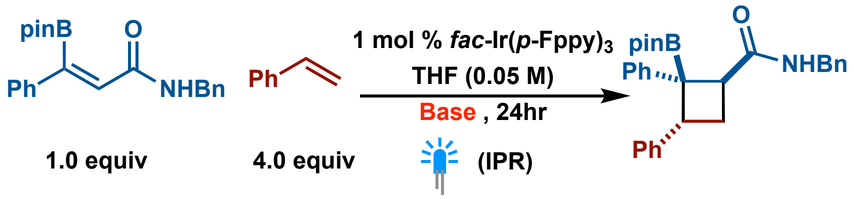

entry	Base	mol%	yield[a]
1	LiOtBu	100%	50%
2	NaOtBu	100%	88%
3	KOtBu	100%	31%
4	Cs_2CO_3	100%	19%
5	KOH	100%	16%
6	DBU	100%	<2%
7	NEt_3	100%	<2%

Condition: The reactions were performed on a 0.1mmol scale, with 4 equiv of styrene, 1 mol% *fac*-Ir(*p*-Fppy)$_3$ and 1 equiv of base in 2 ml THF (0.05M).

Table SI-3 Base equivalent Screening:

An oven-dried 2-dram vial equipped with a magnetic stir bar was charged with (*E*)-*N*-benzyl-3-phenyl-3-(4,4,5,5-tetramethyl-1,3,2-dioxaborolan-2-yl) acrylamide (36.3 mg, 0.2 mmol, 1.0 equiv.) and *fac*-Ir(p-Fppy)$_3$ (0.7 mg, 1 mol%, 0.01 equiv.). The vial was transferred into a nitrogen-filled glovebox, and *sodium* tert-*butoxide (xx mol%)*. was added. The vial was sealed with a septum and removed from the glovebox. Dry THF (2.0 mL, 0.05 M) was added under nitrogen. The reaction mixture was stirred at room temperature under nitrogen for 30 min. Styrene (46 µl, 0.400 mmol, 4 equiv.) was then added dropwise

via syringe. The septum was quickly replaced with a screw cap, and the reaction mixture was irradiated with 450 nm light (light source: Integrated Photoreactor, IPR) at room temperature for 24 h with continuous stirring. Upon completion, the reaction was quenched with saturated aqueous NH_4Cl (3.0 mL) and extracted with EtOAc (3 × 3.0 mL). The combined organic layers were washed with brine (3.0 mL), dried over anhydrous Na_2SO_4, filtered, and concentrated under reduced pressure. CH_2Br_2 was added as an internal standard, and a small aliquot of the crude mixture was analyzed by ^1H NMR spectroscopy to determine the NMR yield and diastereomeric ratio (dr).

E) Optimization Table

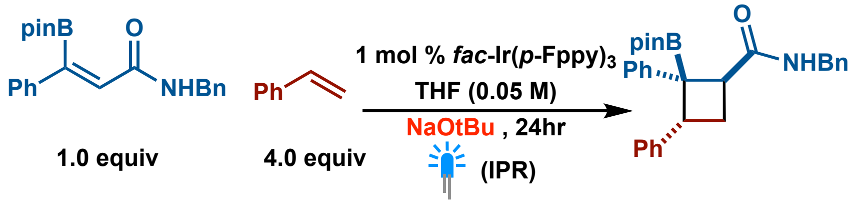

entry	Base	mol%	yield[a]
1	NaOtBu	20%	<2%
2	NaOtBu	50%	20%
3	NaOtBu	100%	88%
4	NaOtBu	150%	52%

Condition: The reactions were performed on a 0.1mmol scale, with 4 equiv of styrene, 1 mol% *fac*-Ir(*p*-Fppy)$_3$ and base in 2 ml THF (0.05M).

Table SI-4 Solvent Screening:

An oven-dried 2-dram vial equipped with a magnetic stir bar was charged with (*E*)-*N*-benzyl-3-phenyl-3-(4,4,5,5-tetramethyl-1,3,2-dioxaborolan-2-yl) acrylamide (36.3 mg, 0.2 mmol, 1.0 equiv.) and *fac*-Ir(p-Fppy)$_3$ (0.7 mg, 1 mol%, 0.01 equiv.). The vial was transferred into a nitrogen-filled glovebox, and sodium *tert*-butoxide (19 mg, 0.2 mmol, 1.0 equiv.) was added. The vial was sealed with a septum and removed from the glovebox. *Solvent* was added under nitrogen. The reaction mixture was stirred at room temperature under nitrogen for 30 min. Styrene (46 µl, 0.400 mmol, 4 equiv.) was then added dropwise via syringe. The septum was quickly replaced with a screw cap, and the reaction mixture was irradiated with 450 nm light (light source: Integrated Photoreactor, IPR) at room temperature for 24 h with continuous stirring. Upon completion, the reaction was quenched with saturated aqueous NH_4Cl (3.0 mL) and extracted with EtOAc (3 × 3.0 mL). The combined organic layers were washed with brine (3.0 mL), dried over anhydrous Na_2SO_4, filtered, and concentrated under reduced pressure. CH_2Br_2 was added as an internal standard, and a small aliquot of the crude mixture was analyzed by ^1H NMR spectroscopy to determine the NMR yield and diastereomeric ratio (dr).

F) Optimization Table

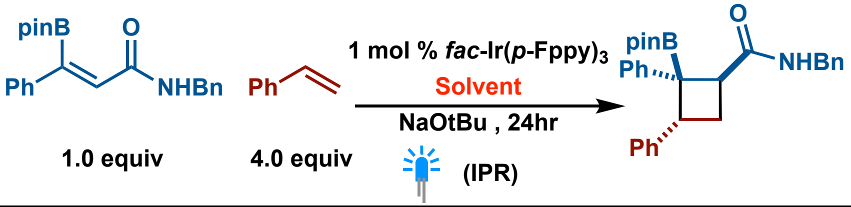

entry	Solvent	Molarity (M)	yield[a]
1	Toulene	0.05	<2%
2	DCM	0.05	51%
3	DMF	0.05	81%
4	MeCN	0.05	28%
5	THF	0.05	88%
6	THF	0.06	60%
7	THF	0.1	17%

Condition: The reactions were performed on a 0.1mmol scale, with 4 equiv of styrene, 1 mol% *fac*-Ir(*p*-Fppy)$_3$ and 1 equiv NaOtBu in solvent.

Table SI-5 Control Experiment:

To gain deeper insight into the reaction mechanism, a series of control experiments were conducted using (E)-N-benzyl-3-phenyl-3-(4,4,5,5-tetramethyl-1,3,2-dioxaborolan-2-yl) acrylamide as the model substrate. These studies demonstrated that the transformation does not proceed in the absence of any one of the following critical components: the [Ir]-catalyst, a base, or 450nm irradiation. This clearly indicates that all three components are essential for the photocatalytic process. The reaction was also conducted under conventional LED irradiation, which provided a 67% yield, compared to the integrated photoreactor that worked more efficiently and was selected for further substrate scope studies. A direct excitation experiment performed in the absence of a photocatalyst gave a 34% yield, suggesting that the reaction proceeds through an excited intermediate.

Furthermore, when the N-benzyl acrylamide moiety was replaced with an *N,N*-dimethylamide (NMe_2) analogue under otherwise identical conditions including the presence of photocatalyst, base, and light no reaction was observed. This outcome highlights the importance of the N–H amide functionality in the substrate. The lack of reactivity in NMe_2 variant suggests that the formation of a five-membered cyclic intermediate, likely involving deprotonation of N–H amide which leads to coordination between the amide oxygen and the boron center, is a crucial mechanistic step. Due to electronic factors, the NMe_2 group cannot participate in stable chelation, likely preventing the formation of the cyclic intermediate. As a result, the catalytic cycle is interrupted because the short-lived triplet radical cannot undergo a bimolecular collision with styrene.

These findings collectively support a mechanistic hypothesis wherein the substrate must adopt a conformation capable of intramolecular coordination, facilitating key steps in the reaction pathway potentially including energy transfer.

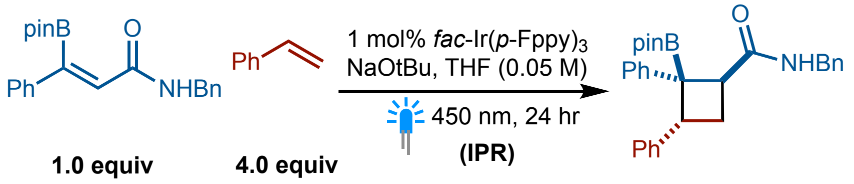

Entry	Deviation from standard condition	NMR Yield
1	no change	88%
2	no base	<2%
3	no light	<2%
4	no catalyst	<2%
5	LED	67%
7	365 nm (no photocatalyst)	34%

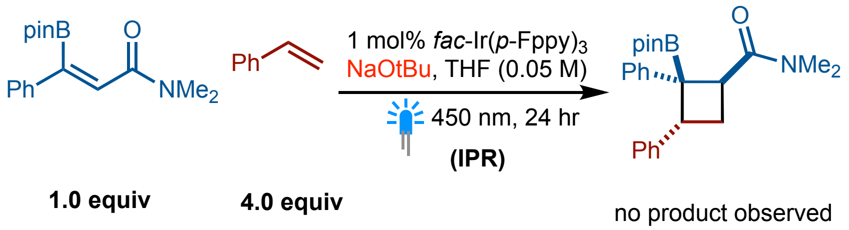

no product observed

Condition: The reactions were performed on a 0.1mmol scale. NMR yield determined by analysis of the unpurified ^{1}H NMR spectrum in the presence of an internal standard

5. Experimental Procedure of Photochemical cycloaddition: General Procedure F

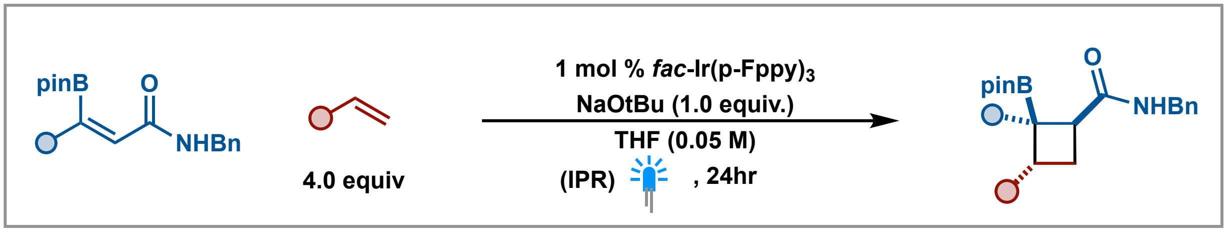

An oven-dried 2-dram vial equipped with a magnetic stir bar was charged with alkenylboronate (0.2 mmol, 1.0 equiv.) and *fac*-Ir(p-Fppy)$_3$ (1.4 mg, 1 mol%, 0.01 equiv.). The vial was transferred into a nitrogen-filled glovebox, and sodium *tert*-butoxide (19 mg, 0.2 mmol, 1.0 equiv.) was added. The vial was sealed with a septum and removed from the glovebox. Dry THF (4.0 mL, 0.05 M) was added under nitrogen. The reaction mixture was stirred at room temperature under nitrogen for 30 min.

Alkene (3-4 equiv) was then added dropwise via syringe under nitrogen flow. The septum was quickly replaced with a screw cap, and the reaction mixture was irradiated with 450 nm light (light source: Integrated Photoreactor, IPR) at room temperature for 24 h with continuous stirring.

Upon completion, the reaction was quenched with saturated aqueous NH_4Cl (3.0 mL) and extracted with EtOAc (3 × 3.0 mL). The combined organic layers were washed with brine (3.0 mL), dried over anhydrous Na_2SO_4, filtered, and concentrated under reduced pressure.

CH_2Br_2 was added as an internal standard, and a small aliquot of the crude mixture was analyzed by ^1H NMR spectroscopy to determine the NMR yield and diastereomeric ratio (dr). The crude product was purified by silica gel column chromatography.

6. Final Product Characterizations

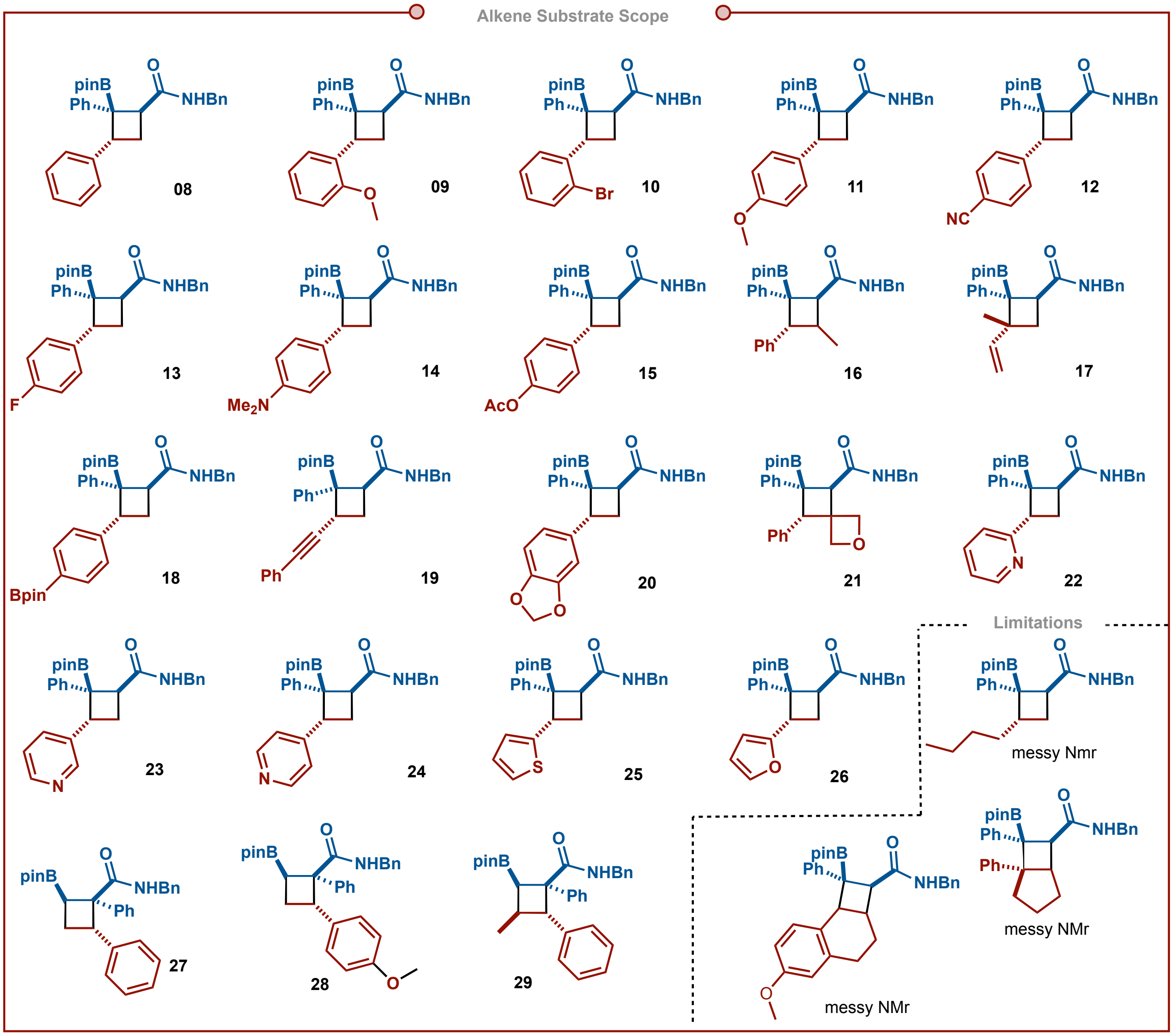

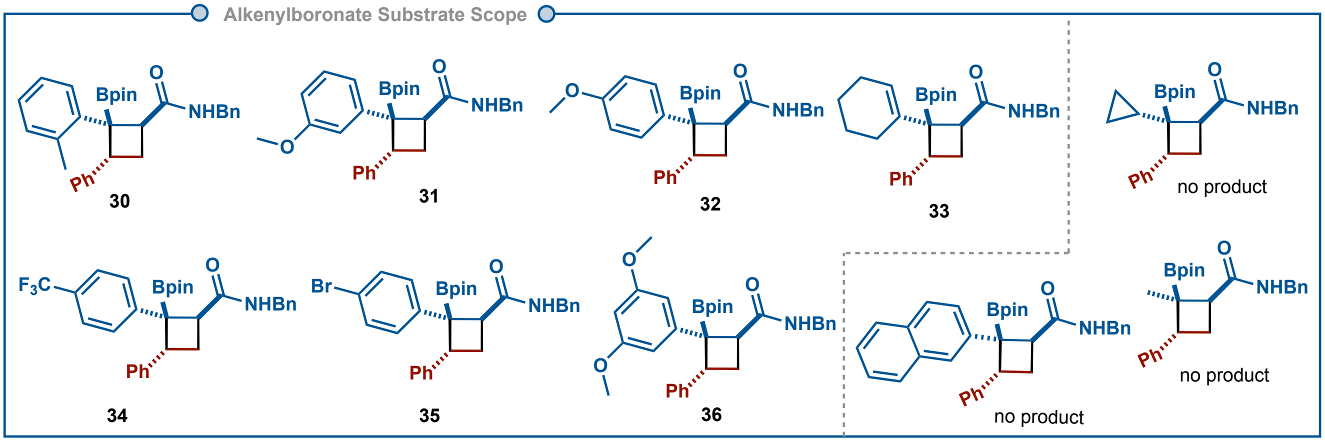

(1*S*,2*S*,3*S*)-*N*-benzyl-2,3-diphenyl-2-(4,4,5,5-tetramethyl-1,3,2-dioxaborolan-2-yl)cyclobutane-1-carboxamide (08)

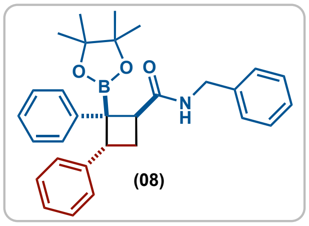

The title compound was prepared according to General Procedure F with alkenylboronate **SI-13** (72.7 mg, 0.20 mmol, 1 equiv.) and phenyl styrene (92 µl, 0.80 mmol, 4 equiv.). The yield of the reaction was obtained by NMR analysis of the unpurified reaction mixture with dibromomethane as an internal standard (88% NMR yield, 8:1 dr). The crude mixture was purified by flash chromatography (1-5 % Et_2O in DCM) to provide the product **08** (69.3 mg, 76 % yield as the major diastereomer).

Physical State: off-white solid

^{1}H NMR (600 MHz, $CDCl_3$): δ 7.38 – 7.31 (m, 4H), 7.31 – 7.26 (m, 1H), 7.09 – 6.97 (m, 7H), 6.96 – 6.91 (m, 1H), 6.87 – 6.81 (m, 2H), 6.01 (t, *J* = 5.7 Hz, 1H), 4.60 (dd, *J* = 14.7, 6.0 Hz, 1H), 4.45 (dd, *J* = 14.7, 5.4 Hz, 1H), 4.30 (dd, *J* = 8.8, 6.1 Hz, 1H), 3.76 (dd, *J* = 9.6, 7.0 Hz, 1H), 2.78 (ddd, *J* = 11.5, 8.9, 6.9 Hz, 1H), 2.54 (ddd, *J* = 11.5, 9.6, 6.2 Hz, 1H), 1.24 (d, *J* = 13.6 Hz, 12H).

^{13}C NMR (126 MHz, $CDCl_3$): δ 175.25, 142.37, 142.15, 138.81, 129.06, 128.27, 128.08, 127.95, 127.87, 127.84, 127.58, 126.13, 125.03, 84.02, 44.86, 44.63, 44.18, 28.97, 25.25, 25.10. (Signal of carbon directly bonded to boron was not detected because of quadrupolar relaxation)

HRMS (ESI): Calc'd for $C_{30}H_{24}BNO_3Na$[M+Na$^+$] 490.2524, found 490.2530

(1*S*,2*S*,3*S*)-*N*-benzyl-3-(2-methoxyphenyl)-2-phenyl-2-(4,4,5,5-tetramethyl-1,3,2-dioxaborolan-2-yl)cyclobutane-1-carboxamide (09)

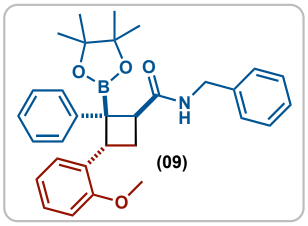

The title compound was prepared according to General Procedure F with alkenylboronate **SI-13** (72.7 mg, 0.20 mmol, 1equiv.) and 1-methoxy-2-vinylbenzene (107 µl, 0.80 mmol, 4 equiv.). The yield of the reaction was obtained by NMR analysis of the unpurified reaction mixture with dibromomethane as an internal standard (92% NMR yield, 8:1 dr). The crude mixture was purified by flash chromatography (1-5 % Et_2O in DCM) to provide the product **09** (69.3 mg, 71 % yield as the major diastereomer).

Physical State: off-white solid

^{1}H NMR (500 MHz, $CDCl_3$): δ 7.35 – 7.32 (m, 4H), 7.28 (dt, *J* = 6.2, 2.6 Hz, 1H), 7.04 – 6.95 (m, 4H), 6.91 – 6.84 (m, 3H), 6.70 (td, *J* = 7.5, 1.2 Hz, 1H), 6.58 (dd, *J* = 8.2, 1.1 Hz, 1H), 5.93 (t, *J* = 5.8 Hz, 1H), 4.65 (dd, *J* = 14.8, 6.4 Hz, 1H), 4.44 (dd, *J* = 9.6, 3.7 Hz, 1H), 4.35 (dd, *J* = 14.8, 5.1 Hz, 1H), 3.92 (t, *J* = 9.6 Hz, 1H), 3.63 (s, 3H), 2.95 (dt, *J* = 11.5, 9.6 Hz, 1H), 2.45 (ddd, *J* = 11.5, 9.8, 3.7 Hz, 1H), 1.24 (d, *J* = 11.3 Hz, 12H).

^{13}C NMR (126 MHz, $CDCl_3$): δ 174.85, 157.52, 143.25, 138.63, 130.53, 128.65, 128.30, 127.88, 127.38, 127.00, 126.74, 124.37, 120.05, 109.88, 83.65, 54.93, 44.47, 43.63, 37.70, 26.70, 24.85, 24.57. (Signal of carbon directly bonded to boron was not detected because of quadrupolar relaxation)

HRMS (ESI): Calc'd for $C_{31}H_{37}BNO_4$[M+H$^+$] 498.2810, found 498.2798

(1*S*,2*S*,3*S*)-*N*-benzyl-3-(2-bromophenyl)-2-phenyl-2-(4,4,5,5-tetramethyl-1,3,2-dioxaborolan-2-yl)cyclobutane-1-carboxamide (10)

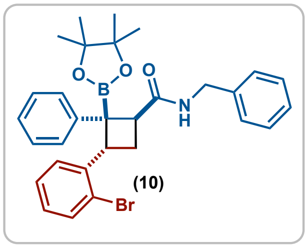

The title compound was prepared according to General Procedure F with alkenylboronate **SI-13** (72.7 mg, 0.20 mmol, 1equiv.) and 1-bromo-2-vinylbenzene (100 µl, 0.80 mmol, 4 equiv.). The yield of the reaction was obtained by NMR analysis of the unpurified reaction mixture with dibromomethane as an internal standard (76% NMR yield, 4:1 dr). The crude mixture was purified by flash chromatography (1-5 % Et_2O in DCM) to provide the product **10** (55 mg, 50 % yield as the major diastereomer).

Physical State: off-white solid

^{1}H NMR (500 MHz, $CDCl_3$): δ 7.34 – 7.29 (m, 1H), 7.27 (d, *J* = 4.4 Hz, 4H), 7.24 – 7.20 (m, 1H), 6.97 – 6.82 (m, 7H), 6.76 (ddd, *J* = 7.9, 7.0, 1.9 Hz, 1H), 5.84 (t, *J* = 5.8 Hz, 1H), 4.60 (dd, *J* = 14.8, 6.4 Hz, 1H), 4.57 – 4.52 (m, 1H), 4.28 (dd, *J* = 14.8, 5.0 Hz, 1H), 3.90 – 3.82 (m, 1H), 3.02 (dt, *J* = 11.9, 9.8 Hz, 1H), 2.27 (ddd, *J* = 11.8, 10.0, 3.3 Hz, 1H), 1.18 (d, *J* = 12.2 Hz, 12H).

^{13}C NMR (126 MHz, $CDCl_3$): δ 174.63, 142.99, 142.20, 138.84, 132.70, 129.09, 128.93, 128.25, 127.87, 127.78, 127.69, 127.46, 127.43, 126.84, 125.03, 84.31, 44.77, 44.06, 43.05, 28.55, 25.20, 25.06 (Signal of carbon directly bonded to boron was not detected because of quadrupolar relaxation)

HRMS (ESI): Calc'd for $C_{30}H_{33}BBrNO_3Na$[M+Na$^+$] 570.16086, found 568.1633

(1*S*,2*S*,3*S*)-*N*-benzyl-3-(4-methoxyphenyl)-2-phenyl-2-(4,4,5,5-tetramethyl-1,3,2-dioxaborolan-2-yl)cyclobutane-1-carboxamide (11)

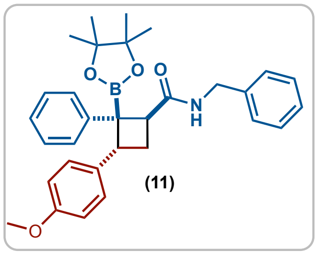

The title compound was prepared according to General Procedure F with alkenylboronate **SI-13** (72.7 mg, 0.20 mmol, 1equiv.) and 1-methoxy-4-vinylbenzene (107 µl, 0.80 mmol, 4 equiv.). The yield of the reaction was obtained by NMR analysis of the unpurified reaction mixture with dibromomethane as an internal standard (76% NMR yield, 8:1 dr). The crude mixture was purified by flash chromatography (1-5 % Et_2O in DCM) to provide the product **11** (63.7 mg, 64 % yield as the major diastereomer).

Physical State: off-white solid

^{1}H NMR (500 MHz, $CDCl_3$): δ 7.36 – 7.33 (m, 4H), 7.33 – 7.26 (m, 1H), 7.04 (t, J = 7.6 Hz, 2H), 6.98 – 6.92 (m, 1H), 6.89 (d, J = 8.5 Hz, 2H), 6.86 – 6.78 (m, 2H), 6.63 – 6.58 (m, 2H), 5.94 (t, J = 5.8 Hz, 1H), 4.60 (dd, J = 14.7, 6.0 Hz, 1H), 4.45 (dd, J = 14.8, 5.4 Hz, 1H), 4.25 (dd, J = 8.8, 6.4 Hz, 1H), 3.75 – 3.70 (m, 1H), 3.69 (d, J = 0.8 Hz, 3H), 2.74 (ddd, J = 11.7, 8.9, 6.7 Hz, 1H), 2.56 – 2.41 (m, 1H), 1.22 (d, J = 10.6 Hz, 12H).

^{13}C NMR (126 MHz, $CDCl_3$): δ 174.76, 157.54, 141.65, 138.36, 134.04, 129.51, 128.57, 127.79, 127.42, 127.34, 127.14, 124.52, 112.88, 83.48, 54.99, 44.04, 43.69, 43.68, 28.94, 24.73, 24.60. (Signal of carbon directly bonded to boron was not detected because of quadrupolar relaxation)

HRMS (ESI): Calc'd for $C_{31}H_{36}BNO_4Na[M+Na^+]$ 520.2629, found 520.2633

(1*S*,2*S*,3*S*)-*N*-benzyl-3-(4-fluorophenyl)-2-phenyl-2-(4,4,5,5-tetramethyl-1,3,2-dioxaborolan-2-yl)cyclobutane-1-carboxamide (12)

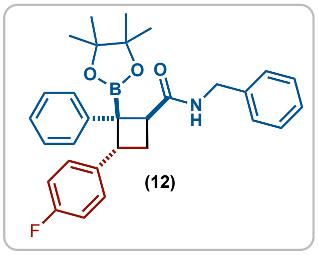

The title compound was prepared according to General Procedure F with alkenylboronate **SI-13** (72.7 mg, 0.20 mmol, 1equiv.) and 1-fluoro-4-vinylbenzene (95.4 µl, 0.80 mmol, 4 equiv.). The yield of the reaction was obtained by NMR analysis of the unpurified reaction mixture with dibromomethane as an internal standard (98% NMR yield, 5:1 dr). The crude mixture was purified by flash chromatography (1-5 % Et_2O in DCM) to provide the product **12** (78.6 mg, 81 % yield as the major diastereomer).

Physical State: white solid

^{1}H NMR (500 MHz, CDCl$_3$): δ 7.34 (d, *J* = 4.4 Hz, 4H), 7.31 – 7.26 (m, 1H), 7.04 (dd, *J* = 8.3, 7.0 Hz, 2H), 6.98 – 6.89 (m, 3H), 6.84 – 6.79 (m, 2H), 6.77 – 6.71 (m, 2H), 6.01 – 5.91 (m, 1H), 4.59 (dd, *J* = 14.7, 6.0 Hz, 1H), 4.45 (dd, *J* = 14.7, 5.4 Hz, 1H), 4.37 – 4.30 (m, 1H), 3.74 – 3.65 (m, 1H), 2.72 (ddd, *J* = 11.4, 8.8, 6.3 Hz, 1H), 2.51 (ddd, *J* = 11.5, 9.6, 6.9 Hz, 1H), 1.23 (d, *J* = 10.1 Hz, 12H).

^{13}C NMR (126 MHz, CDCl$_3$): δ 175.19, 161.55 (d, *J* = 243.6 Hz), 141.63, 138.75, 137.96 (d, *J* = 3.1 Hz), 130.34 (d, *J* = 7.8 Hz), 129.08, 128.28, 128.04, 127.88, 127.62, 125.25, 114.66 (d, *J* = 21.0 Hz), 84.06, 44.26, 44.22, 29.17, 25.25, 25.14. (Signal of carbon directly bonded to boron was not detected because of quadrupolar relaxation)

^{19}F NMR (471 MHz, CDCl$_3$): δ -110.69 – -124.07 (m).

HRMS (ESI): Calc'd for $C_{30}H_{33}FBNO_3Na$[M+Na] 508.2429, found 508.2437

(1*S*,2*S*,3*S*)-*N*-benzyl-3-(4-(dimethylamino)phenyl)-2-phenyl-2-(4,4,5,5-tetramethyl-1,3,2-dioxaborolan-2-yl)cyclobutane-1-carboxamide (13)

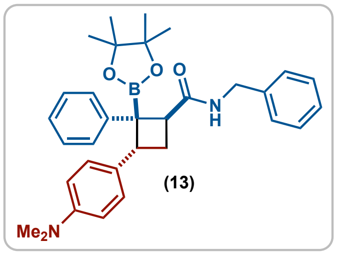

The title compound was prepared according to General Procedure F with alkenylboronate **SI-13** (72.7 mg, 0.20 mmol, 1equiv.) and N,N-dimethyl-4-vinylaniline (120 µl, 0.80 mmol, 4 equiv.). The yield of the reaction was obtained by NMR analysis of the unpurified reaction mixture with dibromomethane as an internal standard (51% NMR yield, 4:1 dr). The crude mixture was purified by flash chromatography (1-5 % Et_2O in DCM) to provide the product **13** (38.8 mg, 38 % yield as the major diastereomer).

Physical State: white solid

^{1}H NMR (500 MHz, CDCl$_3$): δ 7.37 – 7.32 (m, 4H), 7.28 (ddd, *J* = 8.7, 6.6, 3.7 Hz, 1H), 7.05 (t, *J* = 7.6 Hz, 2H), 6.99 – 6.92 (m, 1H), 6.89 – 6.81 (m, 4H), 6.48 (d, *J* = 8.3 Hz, 2H), 5.98 (t, *J* = 5.7 Hz, 1H), 4.60 (dd, *J* = 14.8, 6.0 Hz, 1H), 4.44 (dd, *J* = 14.7, 5.4 Hz, 1H), 4.17 (dd, *J* = 9.0, 6.0 Hz, 1H), 3.74 (dd, *J* = 9.6, 7.0 Hz, 1H), 2.82 (s, 6H), 2.75 (ddd, *J* = 11.3, 9.0, 7.0 Hz, 1H), 2.44 (ddd, *J* = 11.4, 9.7, 6.1 Hz, 1H), 1.21 (d, *J* = 11.4 Hz, 12H).

^{13}C NMR (126 MHz, CDCl$_3$): δ 175.06, 142.25, 138.54, 129.39, 128.67, 127.91, 127.45, 127.43, 127.31, 124.45, 112.33, 83.52, 44.42, 43.78, 40.81, 29.35, 24.83, 24.69. (Signal of carbon directly bonded to boron was not detected because of quadrupolar relaxation)

HRMS (ESI): Calc'd for $C_{32}H_{40}BN_2O_3$[M+H$^+$] 511.31265, found 511.3124

4-((1*S*,2*S*,3*S*)-3-(benzylcarbamoyl)-2-phenyl-2-(4,4,5,5-tetramethyl-1,3,2-dioxaborolan-2-yl)cyclobutyl)phenyl acetate (14)

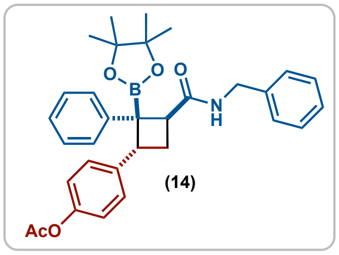

The title compound was prepared according to General Procedure F with alkenylboronate **SI-13** (72.7 mg, 0.20 mmol, 1equiv.) and 4-vinylphenyl acetate (122 µl, 0.80 mmol, 4 equiv.). The yield of the reaction was obtained by NMR analysis of the unpurified reaction mixture with dibromomethane as an internal standard (75% NMR yield, 4:1 dr). The crude mixture was purified by flash chromatography (1-5 % Et_2O in DCM) to provide the product **14** (66.2 mg, 63 % yield as mixture of diastereomers).

Physical State: white solid

^{1}H NMR (500 MHz, $CDCl_3$): δ 7.34 (d, *J* = 4.3 Hz, 3H), 7.31 – 7.26 (m, 1H), 7.04 (t, *J* = 7.6 Hz, 2H), 6.96 (t, *J* = 7.5 Hz, 3H), 6.86 – 6.81 (m, 3H), 6.80 – 6.76 (m, 2H), 6.01 (t, *J* = 5.7 Hz, 1H), 4.58 (dd, *J* = 14.8, 6.0 Hz, 1H), 4.45 (dd, *J* = 14.7, 5.6 Hz, 1H), 4.36 – 4.31 (m, 1H), 3.71 (dd, *J* = 9.6, 6.4 Hz, 1H), 2.72 (ddd, *J* = 11.5, 8.8, 6.4 Hz, 1H), 2.51 (ddd, *J* = 11.5, 9.6, 6.6 Hz, 1H), 2.21 (s, 3H), 1.22 (d, *J* = 11.0 Hz, 12H).

^{13}C NMR (126 MHz, $CDCl_3$): δ 174.79, 173.53(minor diastereoisomer), 169.34, 148.55, 141.21, 139.47, 138.23, 129.44, 128.56, 127.77, 127.50, 127.35, 127.11, 124.69, 120.36, 83.51, 43.87, 43.71, 28.68, 24.73, 24.62, 20.98. (Signal of carbon directly bonded to boron was not detected because of quadrupolar relaxation)

HRMS (ESI): Calc'd for $C_{32}H_{37}BNO_5$[M+H$^+$] 526.2759, found 526.2752

(1*S*,2*S*,3*S*)-*N*-benzyl-3-(4-cyanophenyl)-2-phenyl-2-(4,4,5,5-tetramethyl-1,3,2-dioxaborolan-2-yl)cyclobutane-1-carboxamide (15)

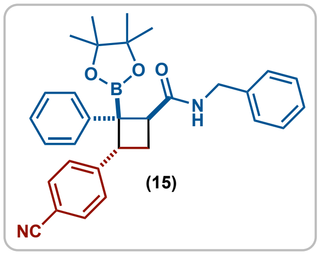

The title compound was prepared according to General Procedure F with alkenylboronate **SI-13** (72.7 mg, 0.20 mmol, 1equiv.) and 4-vinylbenzonitrile (100 µl, 0.80 mmol, 4 equiv.). The yield of the reaction was obtained by NMR analysis of the unpurified reaction mixture with dibromomethane as an internal standard (81% NMR yield, 6:1 dr). The crude mixture was purified by flash chromatography (1-5 % Et_2O in DCM) to provide the product **15** (65.0 mg, 66 % yield as the major diastereomer).

Physical State: off-white solid

^{1}H NMR (500 MHz, CDCl$_3$): δ 7.33 (t, *J* = 4.3 Hz, 6H), 7.29 (dq, *J* = 8.1, 5.3 Hz, 1H), 7.11 – 7.01 (m, 4H), 7.01 – 6.93 (m, 1H), 6.84 – 6.73 (m, 2H), 5.93 (d, *J* = 5.9 Hz, 1H), 4.58 (dd, *J* = 14.8, 5.9 Hz, 1H), 4.51 – 4.40 (m, 2H), 3.66 (dd, *J* = 9.5, 5.8 Hz, 1H), 2.70 (ddd, *J* = 11.6, 8.6, 5.7 Hz, 1H), 2.58 (ddd, *J* = 11.6, 9.5, 7.4 Hz, 1H), 1.24 (d, *J* = 9.7 Hz, 12H).

^{13}C NMR (126 MHz, CDCl$_3$): δ 174.42, 147.57, 140.32, 138.06, 131.23, 129.11, 128.62, 127.80, 127.78, 127.46, 126.96, 125.20, 119.14, 109.25, 83.75, 44.50, 43.79, 43.59, 27.94, 24.80, 24.69. (Signal of carbon directly bonded to boron was not detected because of quadrupolar relaxation)

HRMS: (ESI): Calc'd for $C_{31}H_{34}BN_2O_3$ [MH$^+$] 493.2657, found 493.2662

(1*S*,2*S*,3*S*)-*N*-benzyl-4-methyl-2,3-diphenyl-2-(4,4,5,5-tetramethyl-1,3,2-dioxaborolan-2-yl)cyclobutane-1-carboxamide (16)

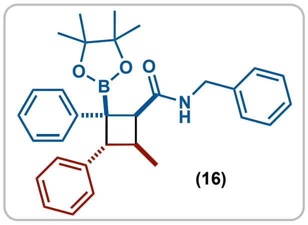

The title compound was prepared according to General Procedure F with alkenylboronate **SI-13** (72.7 mg, 0.20 mmol, 1equiv) and (E)-prop-1-en-1-ylbenzene (104 µl, 0.80 mmol, 4 equiv.). The yield of the reaction was obtained by NMR analysis of the unpurified reaction mixture with dibromomethane as an internal standard (48% NMR yield,4:1 dr). The crude mixture was purified by flash chromatography (1-5 % Et_2O in DCM) to provide the product **16** (27.0 mg, 28 % yield as the major diastereomer).

Physical State: white solid

^{1}H NMR (500 MHz, CDCl$_3$): δ 7.66 – 7.53 (m, 2H), 7.42 – 7.38 (m, 2H), 7.38 – 7.27 (m, 9H), 7.23 – 7.18 (m, 2H), 5.82 (t, *J* = 5.7 Hz, 1H), 4.58 (dd, *J* = 14.9, 5.9 Hz, 1H), 4.47 (dd, *J* = 14.9, 5.5 Hz, 1H), 3.40 (d, *J* = 9.8 Hz, 1H), 3.26 (tq, *J* = 9.9, 6.5 Hz, 1H), 2.79 (d, *J* = 9.8 Hz, 1H), 1.35 (d, *J* = 6.5 Hz, 3H), 0.92 (s, 12H).

^{13}C NMR (126 MHz, CDCl$_3$): δ 173.93, 148.41, 142.34, 138.85, 128.84, 128.22, 128.13, 127.81, 127.72, 127.54, 127.18, 126.50, 125.41, 83.74, 55.38, 51.69, 43.42, 35.25, 24.93, 24.80, 21.35. (Signal of carbon directly bonded to boron was not detected because of quadrupolar relaxation)

HRMS (ESI): Calc'd for $C_{31}H_{36}BNO_3Na$[M+Na$^+$]504.2680, found 504.2691

(1*S*,2*S*,3*S*)-*N*-benzyl-2-phenyl-2-(4,4,5,5-tetramethyl-1,3,2-dioxaborolan-2-yl)-3-(4-(4,4,5,5-tetramethyl-1,3,2-dioxaborolan-2-yl)phenyl)cyclobutane-1-carboxamide (17)

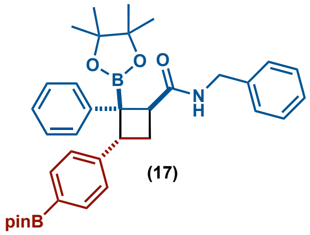

(17)

The title compound was prepared according to General Procedure F with alkenylboronate **SI-13** (72.7 mg, 0.20 mmol, 1equiv) and 4,4,5,5-tetramethyl-2-(4-vinylphenyl)-1,3,2-dioxaborolane (190 µl, 0.80 mmol, 4 equiv.). The yield of the reaction was obtained by NMR analysis of the unpurified reaction mixture with dibromomethane as an internal standard (88% NMR yield, 6:1 dr). The crude mixture was purified by flash chromatography (1-5 % Et_2O in DCM) to provide the product **17** (72.0 mg, 61 % yield as the major diastereomer).

Physical State: white solid

^{1}H NMR (500 MHz, $CDCl_3$): δ 7.73 – 7.61 (m, 2H), 7.57 – 7.44 (m, 2H), 7.30 – 7.26 (m, 6H), 7.26 – 7.19 (m, 3H), 7.15 – 7.09 (m, 1H), 5.71 (t, *J* = 5.7 Hz, 1H), 4.49 (dd, *J* = 14.8, 6.0 Hz, 1H), 4.36 (dd, *J* = 14.8, 5.4 Hz, 1H), 3.83 – 3.72 (m, 1H), 3.11 (dd, *J* = 10.7, 8.5 Hz, 1H), 2.80 (td, *J* = 10.7, 9.2 Hz, 1H), 2.53 (q, *J* = 8.7 Hz, 1H), 1.27 (s, 12H), 0.87 (d, *J* = 16.1 Hz, 12H).

^{13}C NMR (126 MHz, $CDCl_3$): δ 174.09, 148.38, 146.63, 138.77, 134.71, 128.82, 128.09, 127.94, 127.57, 127.13, 126.84, 125.43, 83.81, 83.79, 47.37, 43.45, 43.18, 27.50, 25.01, 24.97, 24.93. (Signal of carbon directly bonded to boron was not detected because of quadrupolar relaxation)

HRMS (ESI): Calc'd for $C_{36}H_{45}B_2NO_5Na[M+Na^+]$ 616.3376, found 616.3386

(1*S*,2*R*,3*R*)-*N*-benzyl-3-methyl-2-phenyl-2-(4,4,5,5-tetramethyl-1,3,2-dioxaborolan-2-yl)-3-vinylcyclobutane-1-carboxamide (18)

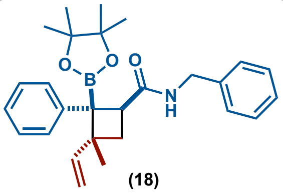

(18)

The title compound was prepared according to General Procedure F with alkenylboronate **SI-13** (72.7 mg, 0.20 mmol, 1 equiv.) and isoprene (80 µl, 0.80 mmol, 4 equiv.). The yield of the reaction was obtained by NMR analysis of the unpurified reaction mixture with dibromomethane as an internal standard (78% yield, 2.9:1 dr, 1.2:1 rr). The crude mixture was purified by flash chromatography (1-5 % Et_2O in DCM) to provide the product **18** (46.6 mg, 54% isolated yield, 2.9:1 dr, 1.2:1 rr). as a mixture of diastereomers and with regioisomers. All of them are marked in the ^{1}H NMR spectra.

Physical State: white solid

^{1}H NMR (500 MHz, $CDCl_3$): δ 7.36 (d, *J* = 4.4 Hz, 5H), 7.33 (d, *J* = 3.9 Hz, 1H), 7.32 – 7.20 (m, 10H), 7.20 – 7.16 (m, 2H), 7.16 – 7.08 (m, 4H), 6.32 – 6.22 (m, 0.32H), 5.92 (s, 1H), 5.75 (dd, *J* = 14.1, 8.5 Hz, 1.32H), 5.62 (dd, *J* = 17.3, 10.7 Hz, 1H), 5.04 – 5.01 (m, 0.35H), 4.95 (dd, *J* = 17.3, 1.3 Hz, 1H), 4.88 –

4.84 (m, 0.36H), 4.82 (dd, J = 10.8, 1.3 Hz, 1H), 4.72 (d, J = 2.6 Hz, 1H), 4.67 (dq, J = 2.9, 1.6 Hz, 1H), 4.64 – 4.54 (m, 2H), 4.38 (ddd, J = 14.6, 8.8, 5.3 Hz, 2H), 3.65 – 3.54 (m, 2H), 3.48 (td, J = 11.5, 8.7 Hz, 1H), 2.77 (t, J = 10.2 Hz, 0.38H), 2.66 – 2.50 (m, 2H), 2.25 (ddd, J = 11.4, 9.4, 4.6 Hz, 1H), 2.17 (dd, J = 9.9, 8.6 Hz, 1H), 1.90 (dd, J = 9.6, 8.7 Hz, 0.37H), 1.47 (s, 3H), 1.38 – 1.31 (m, 3H), 1.21 (d, J = 8.9 Hz, 16H), 1.18 (s, 6H), 1.16 (d, J = 6.1 Hz, 5H), 0.85 (s, 1H).

^{13}C NMR (126 MHz, $CDCl_3$): δ 174.68, 174.50, 173.95, 146.04, 146.00, 145.69, 144.41, 144.28, 143.33, 138.67, 138.65, 138.39, 128.53, 128.04, 127.75, 127.73, 127.66, 127.64, 127.56, 127.52, 127.29, 127.26, 127.17, 127.10, 127.02, 126.99, 126.57, 126.50, 125.04, 124.83, 124.74, 124.62, 113.15, 112.33, 111.03, 110.50, 83.70, 83.59, 83.55, 45.86, 44.74, 44.06, 43.54, 43.19, 43.18, 42.28, 41.85, 33.27, 32.69, 26.26, 26.16, 25.18, 25.13, 24.76, 24.61, 24.52, 22.76, 22.23, 21.65, 20.55. (Signal of carbon directly bonded to boron was not detected because of quadrupolar relaxation)

HRMS (ESI): Calc'd for $C_{27}H_{34}BNO_3Na$[M+Na$^+$] 454.2524, found 454.2525

(1*S*,2*S*,3*S*)-*N*-benzyl-2-phenyl-3-(phenylethynyl)-2-(4,4,5,5-tetramethyl-1,3,2-dioxaborolan-2-yl)cyclobutane-1-carboxamide (19)

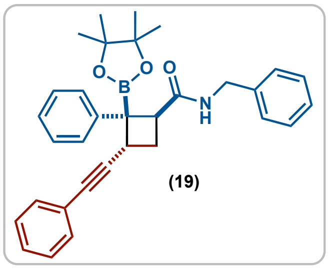

The title compound was prepared according to General Procedure F with alkenylboronate **SI-13** (72.7 mg, 0.20 mmol, 1equiv) and but-3-en-1-yn-1-ylbenzene (190 µl, 0.80 mmol, 4 equiv.). The yield of the reaction was obtained by NMR analysis of the unpurified reaction mixture with dibromomethane as an internal standard (51% NMR yield, 4:1 dr). The crude mixture was purified by flash chromatography (1-5 % Et_2O in DCM) to provide the product **19** (30.0 mg, 29.5 % yield as the major diastereomer).

Physical State: white solid

^{1}H NMR (500 MHz, $CDCl_3$): δ 7.53 – 7.49 (m, 2H), 7.45 – 7.41 (m, 2H), 7.37 (d, J = 4.4 Hz, 3H), 7.34 – 7.27 (m, 7H), 7.17 (tdd, J = 7.2, 5.9, 4.5 Hz, 1H), 5.78 (t, J = 5.8 Hz, 1H), 4.61 (dd, J = 14.8, 6.2 Hz, 1H), 4.42 (dd, J = 14.8, 5.2 Hz, 1H), 3.34 (dd, J = 10.5, 8.3 Hz, 1H), 3.19 (dd, J = 10.8, 8.6 Hz, 1H), 2.81 (td, J = 10.6, 9.4 Hz, 1H), 2.55 (q, J = 8.7 Hz, 1H), 1.22 – 1.14 (m, 12H).

^{13}C NMR (126 MHz, $CDCl_3$): δ 173.90, 148.61, 138.90, 131.96, 129.11, 128.61, 128.36, 128.20, 128.16, 127.89, 126.31, 125.64, 124.12, 91.80, 84.39, 82.96, 47.12, 43.80, 31.62, 29.81, 25.40, 24.98. (Signal of carbon directly bonded to boron was not detected because of quadrupolar relaxation)

HRMS: (ESI): Calc'd for $C_{32}H_{35}BO_3$[MH$^+$] 492.2704, found 492.2713

(1*S*,2*S*,3*S*)-3-(benzo[*d*][1,3]dioxol-5-yl)-*N*-benzyl-2-phenyl-2-(4,4,5,5-tetramethyl-1,3,2-dioxaborolan-2-yl)cyclobutane-1-carboxamide (20)

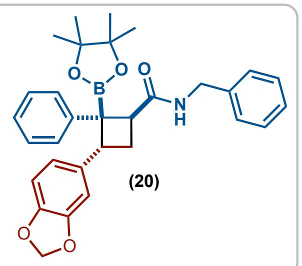

(20)

The title compound was prepared according to General Procedure F with alkenylboronate **SI-13** (72.7 mg, 0.20 mmol, 1equiv) and 5-vinylbenzo[d][1,3]dioxole (99 µl, 0.80 mmol, 4 equiv.). The yield of the reaction was obtained by NMR analysis of the unpurified reaction mixture with dibromomethane as an internal standard (80% NMR yield, 3:1 dr). The crude mixture was purified by flash chromatography (1-5 % Et_2O in DCM) to provide the product **20** (46.0 mg, 45 % yield as the major diastereomer).

Physical State: white solid

^{1}H NMR (500 MHz, $CDCl_3$): δ 7.59 (dt, *J* = 6.8, 1.2 Hz, 2H), 7.40 – 7.27 (m, 7H), 7.23 – 7.15 (m, 1H), 6.90 (d, *J* = 1.8 Hz, 1H), 6.81 (dt, *J* = 8.0, 1.3 Hz, 1H), 6.74 (d, *J* = 8.0 Hz, 1H), 5.91 (s, 2H), 5.77 (q, *J* = 4.3 Hz, 1H), 4.59 (dd, *J* = 14.9, 6.2 Hz, 1H), 4.41 (dd, *J* = 14.8, 5.3 Hz, 1H), 3.76 (dd, *J* = 10.7, 8.4 Hz, 1H), 3.14 (dd, *J* = 10.7, 8.5 Hz, 1H), 2.80 (td, *J* = 10.7, 9.2 Hz, 1H), 2.55 (q, *J* = 8.7 Hz, 1H), 0.99 (s, 12H).

^{13}C NMR (126 MHz, $CDCl_3$): δ 174.32, 148.53, 147.75, 146.27, 139.05, 137.53, 129.08, 128.36, 128.17, 127.82, 127.34, 125.68, 120.83, 108.59, 108.21, 101.17, 84.01, 47.20, 43.68, 43.06, 27.97, 25.19. (Signal of carbon directly bonded to boron was not detected because of quadrupolar relaxation)

HRMS (ESI): Calc'd for $C_{31}H_{34}BNO_5Na[M+Na^+]$ 534.2422, found 534.2429

(5*R*,6*S*,7*S*)-*N*-benzyl-6,7-diphenyl-6-(4,4,5,5-tetramethyl-1,3,2-dioxaborolan-2-yl)-2-oxaspiro[3.3]heptane-5-carboxamide (21)

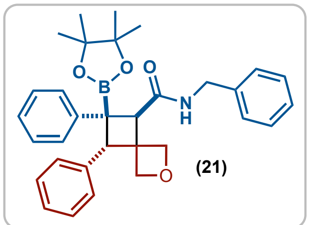

(21)

The title compound was prepared according to General Procedure F with alkenylboronate **SI-13** (72.7 mg, 0.20 mmol, 1equiv) and 3-benzylideneoxetane (99 µl, 0.80 mmol, 4 equiv.). The yield of the reaction was obtained by NMR analysis of the unpurified reaction mixture with dibromomethane as an internal standard (77% NMR yield, 6:1 dr). The crude mixture was purified by flash chromatography (1-5 % Et_2O in DCM) to provide the product **21** (57.0 mg, 56 % yield as the major diastereomer).

Physical State: white solid

^{1}H NMR (500 MHz, $CDCl_3$): δ 7.51 – 7.46 (m, 4H), 7.40 – 7.31 (m, 6H), 7.31 – 7.26 (m, 4H), 7.21 – 7.14 (m, 1H), 6.50 (t, *J* = 5.9 Hz, 1H), 5.06 (d, *J* = 7.5 Hz, 1H), 4.97 (d, *J* = 8.1 Hz, 1H), 4.83 (d, *J* = 8.2

Hz, 1H), 4.69 (dd, J = 14.8, 6.1 Hz, 1H), 4.65 (d, J = 7.5 Hz, 1H), 4.52 (dd, J = 14.8, 5.6 Hz, 1H), 3.85 (s, 1H), 3.15 (s, 1H), 1.10 – 0.95 (m, 12H).

^{13}C NMR (126 MHz, CDCl$_3$): δ 171.06, 148.17, 139.63, 138.41, 128.56, 128.16, 127.88, 127.70, 127.32, 127.25, 126.58, 126.38, 125.35, 83.72, 81.55, 75.63, 57.16, 49.04, 48.55, 43.62, 25.11, 24.76. (The signal of carbon directly bonded to boron was not detected because of quadrupolar relaxation).

HRMS (ESI): Calc'd for $C_{32}H_{37}BNO_4$[M+H$^+$] 510.2810, found 510.2804

(1*S*,2*S*,3*S*)-*N*-benzyl-2-phenyl-3-(pyridin-2-yl)-2-(4,4,5,5-tetramethyl-1,3,2-dioxaborolan-2-yl)cyclobutane-1-carboxamide (22)

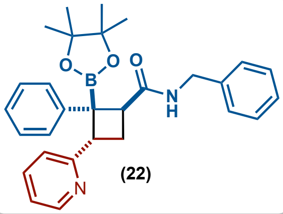

The title compound was prepared according to General Procedure F with alkenylboronate **SI-13** (72.7 mg, 0.20 mmol, 1 equiv.) and 2-vinylpyridine (86.1 µl, 0.80 mmol, 4 equiv.). The yield of the reaction was obtained by NMR analysis of the unpurified reaction mixture with dibromomethane as an internal standard (91% NMR yield, 8:1 dr). The crude mixture was purified by flash chromatography (1-5% Et_2O in DCM) to provide the product **22** (70.3 mg, 75% yield as the major diastereomer).

Physical State: off-white solid

^{1}H NMR (500 MHz, CDCl$_3$): δ 8.19 (ddd, J = 5.0, 1.8, 0.9 Hz, 1H), 7.27 (dd, J = 7.8, 3.1 Hz, 5H), 7.23 – 7.19 (m, 1H), 6.97 (dt, J = 7.8, 1.1 Hz, 1H), 6.91 (t, J = 7.7 Hz, 2H), 6.83 – 6.74 (m, 4H), 5.92 (t, J = 5.8 Hz, 1H), 4.53 (dd, J = 14.8, 6.1 Hz, 1H), 4.34 (dd, J = 14.8, 5.3 Hz, 1H), 4.13 (dd, J = 8.7, 3.7 Hz, 1H), 4.08 – 4.00 (m, 1H), 2.79 (dt, J = 10.9, 8.7 Hz, 1H), 2.61 (ddd, J = 11.0, 9.4, 3.7 Hz, 1H), 1.19 – 1.16 (m, 12H).

^{13}C NMR (126 MHz, CDCl$_3$): δ 174.57, 161.76, 148.63, 142.68, 138.41, 135.06, 128.51, 127.72, 127.51, 127.27, 126.64, 124.26, 124.18, 120.52, 83.63, 45.61, 44.73, 43.57, 26.96, 24.84, 24.56. (The signal of carbon directly bonded to boron was not detected because of quadrupolar relaxation)

HRMS (ESI): Calc'd for $C_{29}H_{34}BN_2O_3Na$[M+Na$^+$] 469.2657, found 469.2662

(1*S*,2*S*,3*S*)-*N*-benzyl-2-phenyl-3-(pyridin-3-yl)-2-(4,4,5,5-tetramethyl-1,3,2-dioxaborolan-2-yl)cyclobutane-1-carboxamide (23)

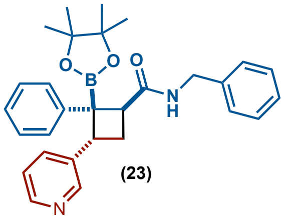

The title compound was prepared according to General Procedure F with alkenylboronate **SI-13** (72.7 mg, 0.20 mmol, 1 equiv.) and 3-vinylpyridine (86 µl, 0.80 mmol, 4 equiv.). The yield of the reaction was obtained by NMR analysis of the unpurified reaction mixture with dibromomethane as an internal standard (88% NMR yield, 8:1 dr). The crude mixture was purified by flash chromatography (1-5% Et_2O in DCM) to provide the product **23** (66.5 mg, 71% yield as the major diastereomer).

Physical State: off-white solid

^{1}H NMR (500 MHz, $CDCl_3$): δ 8.64 (d, *J* = 2.3 Hz, 1H), 8.49 (dd, *J* = 4.8, 1.7 Hz, 1H), 7.76 – 7.69 (m, 1H), 7.61 – 7.56 (m, 2H), 7.40 – 7.30 (m, 7H), 7.27 – 7.20 (m, 2H), 5.79 (t, *J* = 5.8 Hz, 1H), 4.62 (dd, *J* = 14.8, 6.2 Hz, 1H), 4.44 (dd, *J* = 14.8, 5.3 Hz, 1H), 3.87 (dd, *J* = 10.7, 8.3 Hz, 1H), 3.27 (dd, *J* = 10.6, 8.6 Hz, 1H), 2.96 (td, *J* = 10.7, 9.4 Hz, 1H), 2.70 – 2.58 (m, 1H), 0.97 (d, *J* = 7.7 Hz, 12H).

^{13}C NMR (126 MHz, $CDCl_3$): δ 173.72, 149.21, 147.81, 147.71, 138.67, 138.47, 135.23, 128.85, 128.27, 127.92, 127.64, 126.90, 125.67, 122.98, 83.91, 47.29, 43.49, 40.80, 26.74, 24.93, 24.88. (The signal of carbon directly bonded to boron was not detected because of quadrupolar relaxation)

HRMS (ESI): Calc'd for $C_{29}H_{34}BN_2O_3Na[M+Na^+]$ 469.2657, found 469.2660

(1*S*,2*S*,3*S*)-*N*-benzyl-2-phenyl-3-(pyridin-4-yl)-2-(4,4,5,5-tetramethyl-1,3,2-dioxaborolan-2-yl)cyclobutane-1-carboxamide (24)

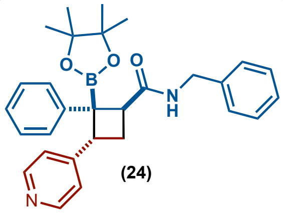

(24)

The title compound was prepared according to General Procedure F with alkenylboronate **SI-13** (72.7 mg, 0.20 mmol, 1 equiv.) and 4-vinylpyridine (86.3 µl, 0.80 mmol, 4 equiv.). The yield of the reaction was obtained by NMR analysis of the unpurified reaction mixture with dibromomethane as an internal standard (93% NMR yield, 5:1 dr). The crude mixture was purified by flash chromatography (1-5% Et_2O in DCM) to provide the product **21** (64.6 mg, 69% yield as the major diastereomer).

Physical State: off-white solid

^{1}H NMR (500 MHz, $CDCl_3$): δ 8.17 – 8.08 (m, 2H), 7.25 (d, *J* = 4.4 Hz, 4H), 7.23 – 7.19 (m, 1H), 6.95 (dd, *J* = 8.2, 7.0 Hz, 2H), 6.89 – 6.85 (m, 1H), 6.84 – 6.82 (m, 2H), 6.76 – 6.72 (m, 2H), 6.38 (t, *J* = 5.6 Hz, 1H), 4.49 (dd, *J* = 14.8, 5.9 Hz, 1H), 4.38 (dd, *J* = 14.7, 5.5 Hz, 1H), 4.26 (dd, *J* = 8.5, 6.8 Hz, 1H), 3.62 (ddd, *J* = 9.5, 6.1, 0.9 Hz, 1H), 2.63 (ddd, *J* = 11.6, 8.6, 6.1 Hz, 1H), 2.45 (ddd, *J* = 11.6, 9.6, 6.8 Hz, 1H), 1.16 (d, *J* = 12.5 Hz, 12H).

^{13}C NMR (126 MHz, $CDCl_3$): δ 174.91, 151.99, 148.50, 140.88, 138.27, 128.79, 127.99, 127.74, 127.62, 127.07, 125.33, 124.08, 83.82, 44.09, 44.05, 43.99, 27.89, 25.04, 24.93. (The signal of carbon directly bonded to boron was not detected because of quadrupolar relaxation)

HRMS (ESI): Calc'd for $C_{29}H_{34}BN_2O_3[M+H^+]$ 469.2657, found 469.2651

(1*S*,2*R*,3*S*)-*N*-benzyl-2-phenyl-2-(4,4,5,5-tetramethyl-1,3,2-dioxaborolan-2-yl)-3-(thiophen-2-yl)cyclobutane-1-carboxamide (22)

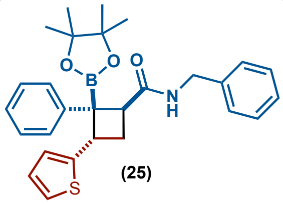

(25)

The title compound was prepared according to General Procedure F with alkenylboronate **SI-13** (72.7 mg, 0.20 mmol, 1 equiv.) and 2-vinylthiophene (83.9 µl, 0.80 mmol, 4 equiv.). The yield of the reaction was obtained by NMR analysis of the unpurified reaction mixture with dibromomethane as an internal standard (30% NMR yield, 7:1 dr). The crude mixture was purified by flash chromatography (1-5% Et_2O in DCM) to provide the product **21** (23.7 mg, 25% yield as the major diastereomer).

Physical State: white solid

^{1}H NMR (500 MHz, $CDCl_3$): δ 7.34 (d, *J* = 4.4 Hz, 4H), 7.31 – 7.26 (m, 1H), 7.15 – 7.03 (m, 2H), 6.97 (m, 1H), 6.93 (dt, *J* = 6.7, 1.5 Hz, 3H), 6.72 (dd, *J* = 5.1, 3.5 Hz, 1H), 6.64 (dt, *J* = 3.5, 0.9 Hz, 1H), 5.98 (t, *J* = 5.8 Hz, 1H), 4.59 (dd, *J* = 14.8, 6.0 Hz, 1H), 4.49 – 4.38 (m, 2H), 3.80 (ddd, *J* = 9.2, 7.9, 1.0 Hz, 1H), 2.97 – 2.86 (m, 1H), 2.41 (ddd, *J* = 11.3, 9.3, 4.9 Hz, 1H), 1.23 (d, *J* = 11.4 Hz, 12H).

^{13}C NMR (126 MHz, $CDCl_3$): δ 174.68, 146.72, 142.31, 138.75, 129.08, 128.29, 128.04, 127.87, 127.27, 126.44, 125.68, 125.37, 123.92, 84.22, 44.58, 44.19, 40.55, 31.57, 25.25, 25.05. (The signal of carbon directly bonded to boron was not detected because of quadrupolar relaxation)

HRMS (ESI): Calc'd for $C_{28}H_{32}BNO_3SNa$[M+Na$^+$] 496.2088, found 496.2095

(1*S*,2*S*,3*S*)-*N*-benzyl-3-(furan-2-yl)-2-phenyl-2-(4,4,5,5-tetramethyl-1,3,2-dioxaborolan-2-yl)cyclobutane-1-carboxamide (23)

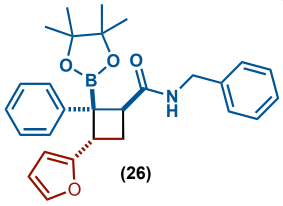

(26)

The title compound was prepared according to General Procedure F with alkenylboronate **SI-13** (72.7 mg, 0.20 mmol, 1 equiv.) and 2-vinylfuran (3.20 ml, 0.25 M in THF, 0.80 mmol, 4 equiv.). The yield of the reaction was obtained by NMR analysis of the unpurified reaction mixture with dibromomethane as an internal standard (46% NMR yield, 3:1 dr). The crude mixture was purified by flash chromatography (1-5% Et_2O in DCM) to provide the product **26** (27.0 mg, 24.7% yield as the major diastereomer).

Physical State: white solid

^{1}H NMR (500 MHz, CDCl$_3$): δ 7.33 (d, *J* = 4.4 Hz, 4H), 7.31 – 7.27 (m, 1H), 7.10 (t, *J* = 7.6 Hz, 2H), 7.03 – 6.98 (m, 2H), 6.97 – 6.92 (m, 2H), 6.06 (dd, *J* = 3.2, 1.8 Hz, 1H), 5.97 (s, 1H), 5.92 (dd, *J* = 3.3, 0.8 Hz, 1H), 4.59 (dd, *J* = 14.7, 6.1 Hz, 1H), 4.42 (dd, *J* = 14.8, 5.4 Hz, 1H), 4.08 (dd, *J* = 9.1, 3.5 Hz, 1H), 3.89 (t, *J* = 9.2 Hz, 1H), 2.86 (dt, *J* = 11.1, 9.2 Hz, 1H), 2.40 (ddd, *J* = 11.1, 9.2, 3.6 Hz, 1H), 1.21 (d, *J* = 12.5 Hz, 12H).

^{13}C NMR (126 MHz, CDCl$_3$): δ 174.62, 156.58, 143.49, 141.43, 138.84, 129.07, 128.28, 127.93, 127.84, 126.58, 125.12, 110.11, 107.33, 84.25, 45.55, 44.12, 38.27, 27.86, 25.23, 24.96. (Signal of carbon directly bonded to boron was not detected because of quadrupolar relaxation)

HRMS (ESI): Calc'd for $C_{28}H_{33}BNO_4$[MH$^+$] 458.2497, found 458.2504

(1*R*,2*S*,4*R*)-*N*-benzyl-1,2-diphenyl-4-(4,4,5,5-tetramethyl-1,3,2-dioxaborolan-2-yl)cyclobutane-1-carboxamide (27)

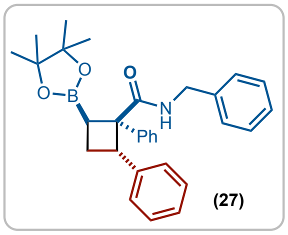

The title compound was prepared according to General Procedure F with alkenylboronate **SI-21** (72.7 mg, 0.20 mmol, 1 equiv.) and phenyl styrene (92.0 µl, 0.80 mmol, 4 equiv.). The yield of the reaction was obtained by NMR analysis of the unpurified reaction mixture with dibromomethanee as an internal standard (>98% NMR yield, 10:1 dr). The crude mixture was purified by flash chromatography (1-5% Et_2O in DCM) to provide the product **27** (87.2 mg, 93% yield).

Physical State: foamy off white solid

^{1}H NMR (500 MHz, CDCl$_3$) δ 7.16 – 7.09 (m, 3H), 7.04 – 6.93 (m, 11H), 6.92 – 6.88 (m, 2H), 5.32 (t, *J* = 5.7 Hz, 1H), 4.51 – 4.42 (m, 2H), 4.16 (dd, *J* = 15.1, 5.1 Hz, 1H), 2.84 (q, *J* = 10.8 Hz, 0H), 2.61 (dd, *J* = 10.1, 6.1 Hz, 1H), 2.50 (ddd, *J* = 10.7, 8.7, 6.0 Hz, 1H), 2.21 (td, *J* = 10.4, 8.0 Hz, 1H).

^{13}C NMR (126 MHz, CDCl$_3$) δ 176.17, 173.38, 141.94, 139.84, 138.75, 129.17, 128.82, 128.61, 128.58, 128.27, 128.07, 127.59, 127.49, 126.92, 126.51, 83.90, 60.96, 47.91, 44.04, 25.45, 25.31, 25.13. (Signal of carbon directly bonded to boron was not detected because of quadrupolar relaxation)

HRMS: Calc'd for $C_{30}H_{34}BNO_3$[MH$^+$] 468.2704, found 468.2694

(1*R*,2*S*,4*R*)-*N*-benzyl-2-(4-methoxyphenyl)-1-phenyl-4-(4,4,5,5-tetramethyl-1,3,2-dioxaborolan-2-yl)cyclobutane-1-carboxamide (28)

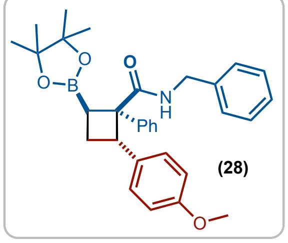

(28)

The title compound was prepared according to General Procedure F with alkenylboronate **SI-21** (72.7 mg, 0.20 mmol, 1 equiv.) and 1-methoxy-4-vinylbenzene (107.0 µl, 0.80 mmol, 4 equiv.). The yield of the reaction was obtained by NMR analysis of the unpurified reaction mixture with dibromomethanee as an internal standard (83% NMR yield). The crude mixture was purified by flash chromatography (1-5% Et_2O in DCM) to provide the product **28** (77.6 mg, 78% yield, 10:1 dr).

Physical State: foamy off-white solid

^{1}H NMR (500 MHz, cdcl$_3$) δ 7.28 – 7.17 (m, 3H), 7.16 – 7.05 (m, 5H), 7.04 – 6.98 (m, 2H), 6.97 – 6.90 (m, 2H), 6.69 – 6.56 (m, 2H), 5.40 (s, 1H), 4.56 (dd, *J* = 15.2, 6.4 Hz, 1H), 4.51 (t, *J* = 8.4 Hz, 1H), 4.29 – 4.17 (m, 1H), 3.73 (s, 3H), 2.67 (dd, *J* = 10.1, 5.8 Hz, 1H), 2.56 (ddd, *J* = 10.6, 8.7, 5.7 Hz, 1H), 2.25 (td, *J* = 10.4, 8.2 Hz, 1H), 1.37 – 1.29 (m, 12H).

^{13}C NMR (126 MHz, CDCl$_3$): δ 175.87, 157.97, 139.45, 138.41, 133.73, 129.77, 128.44, 128.28, 127.94, 127.23, 127.11, 126.53, 113.11, 83.51, 77.28, 77.23, 77.02, 76.77, 60.68, 55.14, 46.85, 43.64, 25.07, 24.95. (Signal of carbon directly bonded to boron was not detected because of quadrupolar relaxation)

HRMS: Calc'd for $C_{31}H_{37}BNO_4$[MH$^+$] 498.2810, found 498.2807

(1*R*,2*S*,4*R*)-*N*-benzyl-3-methyl-1,2-diphenyl-4-(4,4,5,5-tetramethyl-1,3,2-dioxaborolan-2-yl)cyclobutane-1-carboxamide (29)

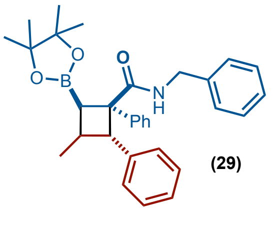

(29)

The title compound was prepared according to General Procedure F with alkenylboronate **SI-21** (72.7 mg, 0.20 mmol, 1 equiv.) and beta styrene (104.0 µl, 0.80 mmol, 4 equiv.). The yield of the reaction was obtained by NMR analysis of the unpurified reaction mixture with dibromomethanee as an internal standard (52% NMR yield, 10:1 dr). The crude mixture was purified by flash chromatography (1-5% Et_2O in DCM) to provide the product **27** (44.3 mg, 46% yield).

Physical State: foamy off-white solid

^{1}H NMR (500 MHz, cdcl$_3$) δ 7.66 – 7.59 (m, 2H), 7.53 – 7.47 (m, 2H), 7.40 – 7.30 (m, 4H), 7.30 – 7.21 (m, 2H), 7.19 – 7.07 (m, 3H), 6.74 – 6.66 (m, 2H), 4.95 (t, *J* = 5.7 Hz, 1H), 4.23 (dd, *J* = 15.2, 5.9 Hz, 1H), 3.96 (dd, *J* = 15.2, 5.5 Hz, 1H), 3.70 (d, *J* = 10.0 Hz, 1H), 3.21 (tq, *J* = 10.0, 6.5 Hz, 1H), 1.58 (d, *J* = 10.1 Hz, 1H), 1.46 – 1.22 (m, 12H), 1.15 (d, *J* = 6.5 Hz, 3H).

^{13}C NMR (126 MHz, CDCl$_3$): δ 173.07, 146.71, 140.13, 138.10, 128.92, 128.74, 128.38, 128.22, 127.04, 126.95, 126.83, 126.77, 82.90, 60.66, 58.54, 43.52, 34.07, 25.31, 24.80, 21.86. (Signal of carbon directly bonded to boron was not detected because of quadrupolar relaxation)

HRMS: Calc'd for $C_{31}H_{37}BNO_3$[MH$^+$] 481.2897, found 481.2895

(1*S*,2*S*,3*S*)-*N*-benzyl-3-phenyl-2-(4,4,5,5-tetramethyl-1,3,2-dioxaborolan-2-yl)-2-(*o*-tolyl)cyclobutane-1-carboxamide (30)

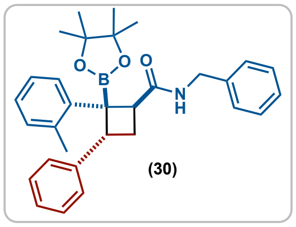

The title compound was prepared according to General Procedure F with alkenylboronate **SI-14** (75.5 mg, 0.20 mmol, 1 equiv.) and phenyl styrene (92.0 µl, 0.80 mmol, 4 equiv.). The yield of the reaction was obtained by NMR analysis of the unpurified reaction mixture with dibromomethanee as an internal standard (42% NMR yield, 5:1 dr). The crude mixture was purified by flash chromatography (1-5% Et_2O in DCM) to provide the product **30** (25.0 mg, 26% yield as the mixture of diastereomers).

Physical State: white solid

^{1}H NMR (500 MHz, CDCl$_3$): δ 7.30 – 7.22 (m, 4H), 7.18 – 7.11 (m, 2H), 7.01 (td, *J* = 7.6, 1.5 Hz, 1H), 6.95 – 6.90 (m, 5H), 6.86 (td, *J* = 7.4, 1.3 Hz, 1H), 6.69 (dd, *J* = 7.6, 1.4 Hz, 1H), 5.92 (t, *J* = 5.8 Hz, 1H), 4.60 (dd, *J* = 14.8, 6.5 Hz, 1H), 4.24 (dd, *J* = 14.8, 5.0 Hz, 1H), 4.06 (t, *J* = 10.2 Hz, 1H), 3.90 (dd, *J* = 9.7, 2.4 Hz, 1H), 3.06 (td, *J* = 10.8, 9.5 Hz, 1H), 2.08 – 2.00 (m, 1H), 1.82 (s, 3H), 1.18 (d, *J* = 13.7 Hz, 12H).

^{13}C NMR (126 MHz, CDCl$_3$): δ 174.26, 143.94, 142.37, 138.85, 135.65, 130.50, 129.07, 128.60, 128.17, 127.94, 127.82, 126.14, 126.11, 125.91, 125.63, 84.37, 46.15, 45.25, 43.94, 30.75, 25.72, 25.39, 20.95. (Signal of carbon directly bonded to boron was not detected because of quadrupolar relaxation)

HRMS: (ESI): Calc'd for $C_{31}H_{36}BNO_3$[M+Na] 504.2680, found 504.2683

(1*S*,2*S*,3*S*)-*N*-benzyl-2-(3-methoxyphenyl)-3-phenyl-2-(4,4,5,5-tetramethyl-1,3,2-dioxaborolan-2-yl)cyclobutane-1-carboxamide (31)

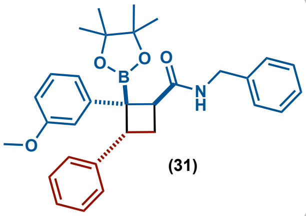

(31)

The title compound was prepared according to General Procedure F with alkenylboronate **SI-15** (78.7 mg, 0.20 mmol, 1equiv) and phenyl styrene (92.0 µl, 0.80 mmol, 4 equiv.). The yield of the reaction was obtained by NMR analysis of the unpurified reaction mixture with dibromomethane as an internal standard (98% NMR yield, 5:1 dr). The crude mixture was purified by flash chromatography (1-5 % Et_2O in DCM) to provide the product **31** (80.6 mg, 81 % yield as the major diastereomer).

Physical State: white solid

^{1}H NMR (500 MHz, $CDCl_3$): δ 7.37 – 7.30 (m, 4H), 7.32 – 7.25 (m, 1H), 7.08 (ddd, *J* = 9.2, 5.7, 1.2 Hz, 2H), 7.02 (td, *J* = 6.4, 1.6 Hz, 3H), 6.97 (t, *J* = 7.9 Hz, 1H), 6.54 – 6.45 (m, 2H), 6.27 (t, *J* = 2.1 Hz, 1H), 5.95 (t, *J* = 5.7 Hz, 1H), 4.60 (dd, *J* = 14.7, 6.0 Hz, 1H), 4.45 (dd, *J* = 14.7, 5.4 Hz, 1H), 4.37 – 4.31 (m, 1H), 3.70 (dd, *J* = 9.6, 6.4 Hz, 1H), 3.49 (s, 3H), 2.71 (ddd, *J* = 11.5, 8.8, 6.4 Hz, 1H), 2.56 (ddd, *J* = 11.5, 9.7, 6.8 Hz, 1H), 1.23 (d, *J* = 8.2 Hz, 12H).

^{13}C NMR (126 MHz, $CDCl_3$): δ 174.88, 158.83, 143.20, 141.94, 138.40, 128.73, 128.70, 128.46, 127.91, 127.61, 127.48, 125.83, 119.41, 113.11, 110.92, 83.61, 54.87, 44.61, 44.23, 43.83, 28.58, 24.81. (Signal of carbon directly bonded to boron was not detected because of quadrupolar relaxation)

HRMS: (ESI): Calc'd for $C_{31}H_{36}BNO_4$[M+Na] 520.2629, found 520.2635

(1*S*,2*S*,3*S*)-*N*-benzyl-2-(4-methoxyphenyl)-3-phenyl-2-(4,4,5,5-tetramethyl-1,3,2-dioxaborolan-2-yl)cyclobutane-1-carboxamide (32)

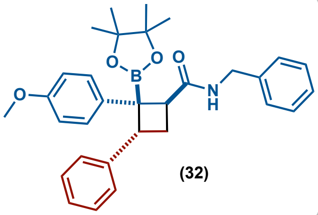

(32)

The title compound was prepared according to General Procedure F with alkenylboronate **SI-16** (78.7 mg, 0.20 mmol, 1equiv) and phenyl styrene (92.0 µl, 0.80 mmol, 4 equiv.). The yield of the reaction was obtained by NMR analysis of the unpurified reaction mixture with dibromomethane as an internal standard (97% NMR yield, 7.8:1 dr). The crude mixture was purified by flash chromatography (1-5 % Et_2O in DCM) to provide the product **32** (78.6 mg, 79% yield as the major diastereomer).

Physical State: white solid

^{1}H NMR (500 MHz, $CDCl_3$): δ 7.35 (d, *J* = 3.5 Hz, 4H), 7.32 – 7.27 (m, 1H), 7.15 – 7.07 (m, 2H), 7.07 – 7.02 (m, 3H), 7.01 – 6.96 (m, 2H), 6.75 – 6.61 (m, 2H), 5.96 (s, 1H), 4.58 (dd, *J* = 14.7, 6.0 Hz, 1H),

4.47 (dd, J = 14.7, 5.4 Hz, 1H), 4.21 (dd, J = 8.9, 5.9 Hz, 1H), 3.75 – 3.68 (m, 1H), 3.66 (s, 3H), 2.78 (ddd, J = 11.4, 8.9, 7.2 Hz, 1H), 2.50 (ddd, J = 11.4, 9.7, 5.9 Hz, 1H), 1.22 (d, J = 12.2 Hz, 12H).

^{13}C NMR (126 MHz, CDCl$_3$): δ 175.37, 157.17, 142.33, 138.82, 134.06, 129.07, 129.04, 128.54, 128.27, 127.96, 127.81, 126.09, 113.39, 83.98, 77.62, 55.40, 44.69, 44.16, 28.81, 25.12, 24.94. (Signal of carbon directly bonded to boron was not detected because of quadrupolar relaxation)

HRMS (ESI): Calc'd for $C_{31}H_{37}BNO_4$[M+H$^+$] 498.2810, found 498.2807

(1*S*,2*S*,3*S*)-*N*-benzyl-3-phenyl-2-(4,4,5,5-tetramethyl-1,3,2-dioxaborolan-2-yl)-2-(4-(trifluoromethyl)phenyl)cyclobutane-1-carboxamide (33)

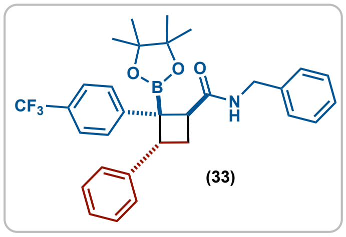

The title compound was prepared according to General Procedure F with alkenylboronate **SI-17** (86.3 mg, 0.20 mmol, 1equiv) and phenyl styrene (92.0 µl, 0.80 mmol, 4 equiv.). The yield of the reaction was obtained by NMR analysis of the unpurified reaction mixture with dibromomethane as an internal standard (98% NMR yield, 9:1 dr). The crude mixture was purified by flash chromatography (1-5 % Et_2O in DCM) to provide the product **33** (95.3 mg, 89% yield as the major diastereomer).

Physical State: white solid

^{1}H NMR (500 MHz, CDCl$_3$): δ 7.34 (s, 6H), 7.30 (ddd, J = 8.5, 3.5, 1.9 Hz, 1H), 7.11 – 7.01 (m, 3H), 7.00 – 6.95 (m, 2H), 6.91 (d, J = 8.1 Hz, 2H), 5.97 (t, J = 5.7 Hz, 1H), 4.59 (dd, J = 14.7, 5.9 Hz, 1H), 4.49 (dd, J = 14.8, 5.5 Hz, 1H), 4.24 (dd, J = 8.9, 5.7 Hz, 1H), 3.77 (dd, J = 9.8, 7.2 Hz, 1H), 2.82 (ddd, J = 11.4, 9.0, 7.2 Hz, 1H), 2.51 (ddd, J = 11.5, 9.9, 5.8 Hz, 1H), 1.23 (d, J = 15.8 Hz, 12H).

^{13}C NMR (126 MHz, CDCl$_3$): δ 175.20, 147.25, 141.99, 138.55, 129.16, 129.00, 128.34, 128.29, 128.26, 128.02, 127.79, 126.95 (q, J = 125 Hz), 126.56, 124.63 (q, J = 15 Hz), 84.10, 45.32, 44.96, 44.33, 29.46, 25.22, 25.12. (Signal of carbon directly bonded to boron was not detected because of quadrupolar relaxation)

^{19}F NMR (471 MHz, CDCl$_3$): δ -62.13.

HRMS (ESI): Calc'd for $C_{31}H_{34}BF_3NO_3$[M+H$^+$] 536.2578, found 536.2571

(1*S*,2*S*,3*S*)-*N*-benzyl-2-(4-bromophenyl)-3-phenyl-2-(4,4,5,5-tetramethyl-1,3,2-dioxaborolan-2-yl)cyclobutane-1-carboxamide (34)

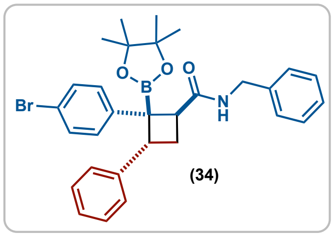

(34)

The title compound was prepared according to General Procedure F with alkenylboronate **SI-18** (88.4 mg, 0.20 mmol, 1equiv) and phenyl styrene (92.0 µl, 0.80 mmol, 4 equiv.). The yield of the reaction was obtained by NMR analysis of the unpurified reaction mixture with dibromomethane as an internal standard (62% NMR yield, 2.5:1 dr). The crude mixture was purified by flash chromatography (1-5 % Et_2O in DCM) to provide the product **34** (44.8 mg, 41% yield as the major diastereomer).

Physical State: white solid

^{1}H NMR (500 MHz, $CDCl_3$): δ 7.35 (d, *J* = 3.5 Hz, 4H), 7.32 – 7.27 (m, 1H), 7.15 – 7.07 (m, 4H), 7.07 – 7.02 (m, 1H), 7.01 – 6.96 (m, 2H), 6.75 – 6.61 (m, 2H), 5.96 (s, 1H), 4.58 (dd, *J* = 14.7, 6.0 Hz, 1H), 4.47 (dd, *J* = 14.7, 5.4 Hz, 1H), 4.21 (dd, *J* = 8.9, 5.9 Hz, 1H), 3.75 – 3.64 (m, 1H), 2.78 (ddd, *J* = 11.4, 8.9, 7.2 Hz, 1H), 2.50 (ddd, *J* = 11.4, 9.7, 5.9 Hz, 1H), 1.22 (d, *J* = 12.2 Hz, 12H).

^{13}C NMR (126 MHz, $CDCl_3$): δ 174.64, 141.59, 141.32, 138.13, 130.30, 128.85, 128.62, 128.55, 127.77, 127.72, 127.45, 125.96, 118.28, 83.5, 44.66, 44.17, 43.74, 28.77, 24.70, 24.61. (Signal of carbon directly bonded to boron was not detected because of quadrupolar relaxation).

HRMS (ESI): Calc'd for $C_{30}H_{34}BBrNO_3$[M+H$^+$] 546.18096, found 546.1803

(1*S*,2*S*,3*S*)-*N*-benzyl-2-(3,5-dimethoxyphenyl)-3-phenyl-2-(4,4,5,5-tetramethyl-1,3,2-dioxaborolan-2-yl)cyclobutane-1-carboxamide (35)

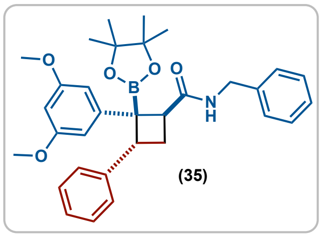

(35)

The title compound was prepared according to General Procedure F with alkenylboronate **SI-19** (84.7 mg, 0.20 mmol, 1 equiv.) and phenyl styrene (92.0 µl, 0.80 mmol, 4 equiv.). The yield of the reaction was obtained by NMR analysis of the unpurified reaction mixture with dibromomethane as an internal standard (55% NMR yield, 4.3:1 dr). The crude mixture was purified by flash chromatography (1-5 % Et_2O in DCM) to provide the product **35** (41.1 mg, 39% yield as the mixture of diastereomer).

Physical State: off white solid

^{1}H NMR (500 MHz, $CDCl_3$) (1:0.45 mixture of diasteromers): δ 7.39 – 7.32 (m, 5H), 7.31 – 7.26 (m, 3H), 7.23 – 7.15 (m, 1H), 7.13 – 6.99 (m, 6H), 6.95 – 6.77 (m, 1H), 6.68 – 6.57 (m, 2H), 6.09 – 5.98 (m, 2H), 5.76 (t, *J* = 5.7 Hz, 0.40H), 4.58 (dd, *J* = 14.8, 5.9 Hz, 1.45H), 4.49 – 4.35 (m, 3H), 3.88 (d, *J* = 3.1

Hz, 3H), 3.76 (s, 3H), 3.66 – 3.60 (m, 1H), 3.41 (s, 3H), 3.11 (dd, *J* = 10.7, 8.5 Hz, 0.45H), 2.93 – 2.79 (m, 0.59H), 2.69 – 2.54 (m, 2.55H), 1.22 (d, *J* = 5.9 Hz, 12H), 0.96 – 0.87 (m, 5H).

^{13}C NMR (126 MHz, CDCl$_3$) (mixture of diasteromers): δ 175.12, 174.16, 148.48, 147.77, 146.76, 146.25, 143.33, 141.90, 140.81, 138.55, 138.41, 133.85, 128.80, 128.69, 128.02, 127.91, 127.67, 127.49, 127.09, 125.89, 117.98, 117.64, 112.16, 111.93, 110.46, 110.32, 83.57, 83.51, 55.61, 55.25, 47.57, 44.42, 44.13, 43.85, 43.36, 41.87, 28.54, 26.85, 24.91, 24.83, 24.75, 24.59. (Signal of carbon directly bonded to boron was not detected because of quadrupolar relaxation)

HRMS (ESI): Calc'd for $C_{32}H_{39}BNO_5$[M+H$^+$] 528.2915, found 528.2913

(1*S*,2*S*,3*S*)-*N*-benzyl-2-(cyclohex-1-en-1-yl)-3-phenyl-2-(4,4,5,5-tetramethyl-1,3,2-dioxaborolan-2-yl)cyclobutane-1-carboxamide (36)

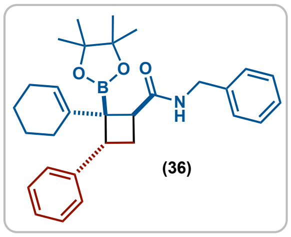

The title compound was prepared according to General Procedure F with alkenylboronate **SI-20** (88.4 mg, 0.20 mmol, 1 equiv.) and phenyl styrene (92.0 µl, 0.80 mmol, 4 equiv.). The yield of the reaction was obtained by NMR analysis of the unpurified reaction mixture with dibromomethane as an internal standard (48% NMR yield, 1.2:1 dr). The crude mixture was purified by flash chromatography (1-5 % Et_2O in DCM) to provide the product **36** (20.0 mg, 21% yield as the major diastereomer).

Physical State: white solid

^{1}H NMR (500 MHz, CDCl$_3$): δ 7.37 – 7.26 (m, 5H), 7.26 – 7.18 (m, 4H), 7.15 (s, 1H), 5.79 (t, *J* = 5.9 Hz, 1H), 5.40 (d, *J* = 3.9 Hz, 1H), 4.67 (dd, *J* = 14.8, 6.7 Hz, 1H), 4.25 (dd, *J* = 14.8, 4.8 Hz, 1H), 3.92 (dd, *J* = 9.2, 4.3 Hz, 1H), 3.58 (t, *J* = 9.1 Hz, 1H), 2.80 (dt, *J* = 11.6, 8.9 Hz, 1H), 2.22 (ddd, *J* = 11.7, 9.5, 4.3 Hz, 1H), 2.01 – 1.92 (m, 1H), 1.89 – 1.79 (m, 1H), 1.67 – 1.53 (m, 3H), 1.29 (d, *J* = 4.0 Hz, 12H), 1.27 – 1.19 (m, 1H), 1.18 – 1.09 (m, 1H), 0.66 (dt, *J* = 10.1, 4.9 Hz, 1H).

^{13}C NMR (126 MHz, CDCl$_3$): δ 174.29, 142.87, 138.51, 138.06, 128.64, 128.51, 127.71, 127.32, 127.23, 125.73, 120.19, 83.62, 43.46, 43.01, 42.59, 27.58, 25.17, 25.14, 24.74, 22.12, 22.06. (Signal of carbon directly bonded to boron was not detected because of quadrupolar relaxation)

HRMS (ESI): Calc'd for $C_{30}H_{39}BNO_3$[MH$^+$] 472.3017, found 472.3017

7. Large Scale Reaction Set up-1

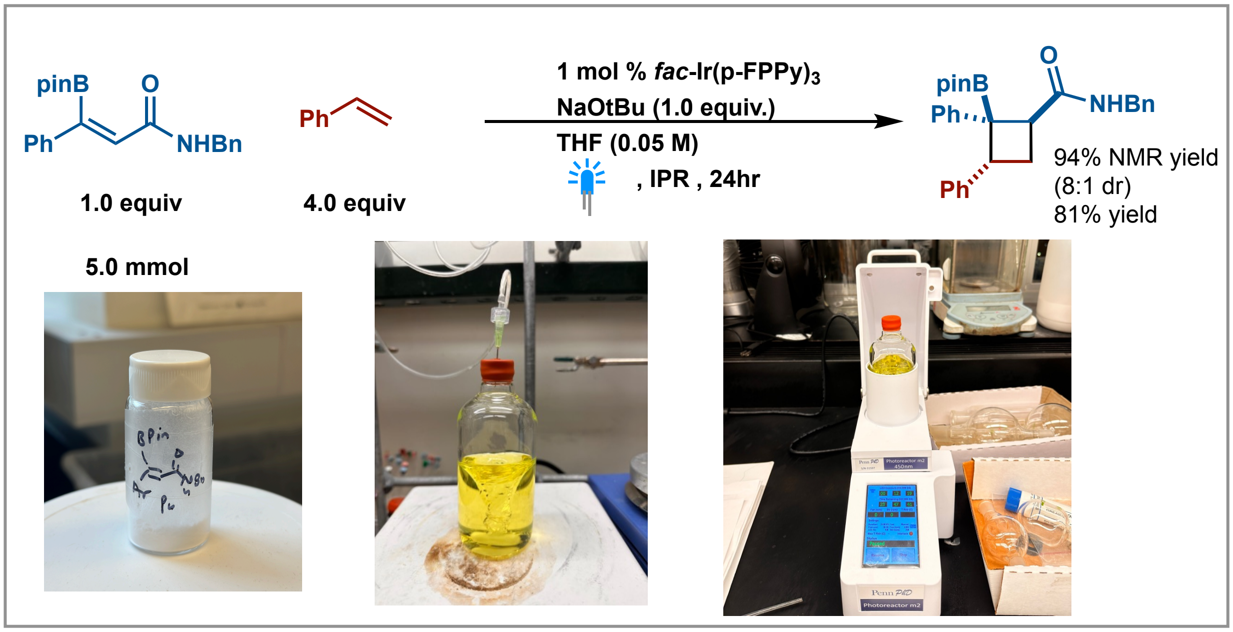

An oven-dried 100 ml glass vial equipped with a magnetic stir bar was charged with (*E*)-*N*-benzyl-3-phenyl-3-(4,4,5,5-tetramethyl-1,3,2-dioxaborolan-2-yl)acrylamide (1.8 gm, 5.00 mmol, 1.0 equiv) and *fac*-Ir(p-Fppy)$_3$ (35.4 mg, 1 mol%, 0.01 equiv). The vial was transferred into a nitrogen-filled glovebox, and sodium *tert*-butoxide (481 mg, 5.0 mmol, 1.0 equiv) was added. The vial was sealed with a septum and removed from the glovebox. Dry THF (100 mL, 0.05 M) was added under nitrogen. The reaction mixture was stirred at room temperature under nitrogen for 30 min.

Styrene (2.30 ml, 20.0 mmol, 4.0 equiv.) was then added dropwise via syringe. The septum was quickly replaced with a screw cap, and the reaction mixture was irradiated with 450 nm light (light source: Integrated Photoreactor, IPR) at room temperature for 24 h with continuous stirring.

Upon completion, the reaction was quenched with saturated aqueous NH_4Cl (75 mL) and extracted with EtOAc (3 × 50 mL). The combined organic layers were washed with brine (40 mL), dried over anhydrous Na_2SO_4, filtered, and concentrated under reduced pressure.

The yield of the reaction was obtained by NMR analysis of the unpurified reaction mixture with dibromomethane as an internal standard (94% NMR yield, 8:1 dr). The crude mixture was purified by flash chromatography (1-5 % Et_2O in DCM) to provide the product (1.9 gm, 81% yield as a mixture of diastereoisomer).

8. Further Transformation:

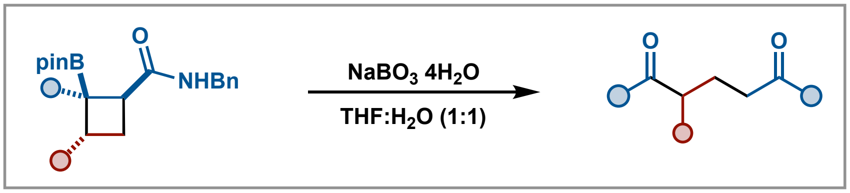

General Procedure G : Oxidation 1

To a 2-dram vial containing the [2+2] cycloaddition pure product (0.1 mmol) was added THF (1.0 ml) and H_2O (1.0 ml) sequentially via syringe. $NaBO_3$. $4H_2O$ (154 mg, 10.0 equiv) was than added to the reaction mixture in one portion under open air. The reaction was stirred at room temperature for 12 hr. The reaction was tracked with checking TLC. Upon completion reaction was quenched with sat. aq. $Na_2S_2O_3$ (1 ml) and further diluted with H_2O (1ml). The organic layer was separated, and the aqueous layer was extracted with ethyl acetate (3 × 1.0 mL). The combined organic layers were dried over Na_2SO_4 filtered, and concentrated under reduced pressure[xii].

CH_2Br_2 was added as an internal standard, and a small aliquot of the crude mixture was analyzed by ^1H NMR spectroscopy to determine the NMR yield. The crude product was purified by silica gel column chromatography.

N-benzyl-5-oxo-4,5-diphenylpentanamide (38)

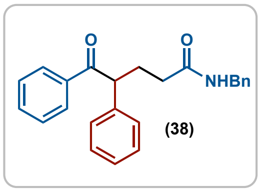

The title compound was prepared according to General Procedure G with compound **09** (47.0 mg, 0.10 mmol, 1equiv.) and The yield of the reaction was obtained by NMR analysis of the unpurified reaction mixture with dibromomethane as an internal standard (>98% NMR yield). The crude mixture was purified by flash chromatography (5-30 % Ethyl acetate in Hexane) to provide the product **38** (33.5 mg, 93% yield).

Physical State: colorless liquid

^{1}H NMR (500 MHz, $CDCl_3$): δ 7.90 – 7.82 (m, 2H), 7.43 – 7.37 (m, 1H), 7.30 (dd, *J* = 8.4, 7.2 Hz, 2H), 7.27 – 7.21 (m, 3H), 7.21 – 7.16 (m, 7H), 7.12 (ddt, *J* = 6.4, 4.8, 3.0 Hz, 1H), 5.61 (s, 1H), 4.68 – 4.60 (m, 1H), 4.37 (dd, *J* = 14.6, 5.8 Hz, 1H), 4.31 (dd, *J* = 14.6, 5.7 Hz, 1H), 2.45 – 2.36 (m, 1H), 2.18 – 2.08 (m, 3H).

^{13}C NMR (126 MHz, $CDCl_3$): δ 199.50, 172.05, 138.78, 138.14, 136.46, 132.86, 128.90, 128.65, 128.62, 128.42, 128.23, 127.74, 127.44, 127.12, 52.21, 43.50, 33.83, 29.41.

HRMS (ESI): Calc'd for $C_{24}H_{24}NO_2[M+H^+]$ 358.18016, found 358.179

N-benzyl-5-(4-methoxyphenyl)-5-oxo-4-phenylpentanamide (39)

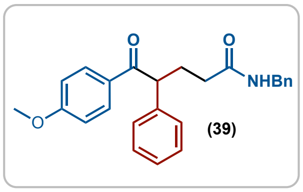

The title compound was prepared according to General Procedure G with compound **32** (50.0 mg, 0.10 mmol, 1 equiv.) and The yield of the reaction was obtained by NMR analysis of the unpurified reaction mixture with dibromomethane as an internal standard (92% NMR yield). The crude mixture was purified by flash chromatography (5-30 % Ethyl acetate in Hexane) to provide the product **39** (32.7 mg, 84% yield).

Physical State: colorless liquid

^{1}H NMR (500 MHz, CDCl$_3$): δ 8.01 – 7.85 (m, 2H), 7.34 – 7.22 (m, 9H), 7.18 (ddd, *J* = 8.7, 5.0, 4.0 Hz, 1H), 6.95 – 6.75 (m, 2H), 5.76 (t, *J* = 5.8 Hz, 1H), 4.69 – 4.63 (m, 1H), 4.43 (dd, *J* = 14.7, 5.8 Hz, 1H), 4.38 (dd, *J* = 14.6, 5.7 Hz, 1H), 3.80 (s, 3H), 2.54 – 2.39 (m, 1H), 2.27 – 2.11 (m, 3H).

^{13}C NMR (126 MHz, CDCl$_3$): δ 197.96, 172.12, 163.24, 139.22, 138.18, 130.94, 129.43, 128.81, 128.57, 128.12, 127.71, 127.37, 126.98, 113.58, 55.28, 51.83, 43.44, 33.90, 29.48.

HRMS (ESI): Calc'd for $C_{25}H_{26}NO_3[M+H^+]$ 388.1907, found 388.1899

General Procedure H: Oxidation 2

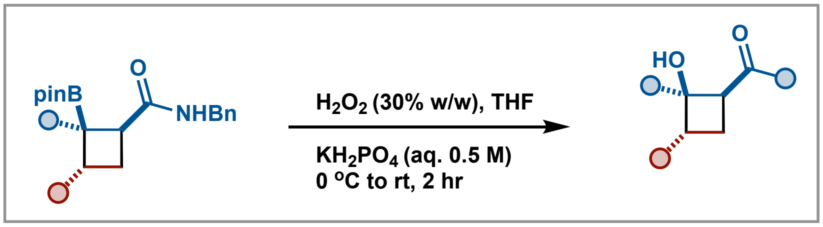

To a 2-dram vial containing the [2+2] cycloaddition pure product (0.1 mmol), THF (0.5 mL), and an aqueous KH_2PO_4 solution (0.5 M, 0.5 mL) were added to the vial with a magnetic stir bar. H_2O_2 (30% w/w in water, 0.25 mL) was added to the vessel dropwise at 0 °C. The reaction was stirred at room temperature for 12 hr. The reaction was tracked with checking TLC. Upon completion reaction mixture further diluted with H_2O (2 ml). The organic layer was separated, and the aqueous layer was extracted with ethyl acetate (3 × 3.0 mL). The combined organic layers were dried over Na_2SO_4 filtered and concentrated under reduced pressure[xiii].

CH_2Br_2 was added as an internal standard, and a small aliquot of the crude mixture was analyzed by ^{1}H NMR spectroscopy to determine the NMR yield. The crude product was purified by silica gel column chromatography.

(1*S*,2*R*,3*R*)-*N*-benzyl-2-hydroxy-2,3-diphenylcyclobutane-1-carboxamide (40)

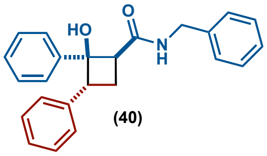

(40)

The title compound was prepared according to General Procedure H with compound **09** (47.0 mg, 0.10 mmol, 1 equiv.) and THF (0.5 mL), and an aqueous KH_2PO_4 solution (0.5 mL, 0.5 M, 3 equiv.). The yield of the reaction was obtained by NMR analysis of the unpurified reaction mixture with dibromomethane as an internal standard (82% NMR yield). The crude mixture was purified by flash chromatography (5-30 % Ethyl acetate in Hexane) to provide the product **40** (26.8mg, 75% yield).

Physical State: white solid

^{1}H NMR (500 MHz, $CDCl_3$): δ 7.41 – 7.28 (m, 5H), 7.27 – 7.22 (m, 2H), 7.17 – 6.99 (m, 6H), 6.88 (dq, *J* = 6.2, 1.1 Hz, 2H), 6.05 (d, *J* = 5.7 Hz, 1H), 5.15 (s, 1H), 4.53 (d, *J* = 2.5 Hz, 1H), 4.52 (d, *J* = 2.5 Hz, 1H), 4.31 (t, *J* = 9.9 Hz, 1H), 3.53 (ddd, *J* = 9.4, 3.9, 1.0 Hz, 1H), 2.53 (ddd, *J* = 11.8, 10.0, 3.9 Hz, 1H), 2.45 (dt, *J* = 11.8, 9.6 Hz, 1H).

^{13}C NMR (126 MHz, $CDCl_3$): δ 174.54, 141.42, 139.45, 138.04, 129.24, 128.23, 128.12, 128.10, 127.69, 127.43, 127.39, 126.45, 126.09, 80.65, 55.56, 48.47, 44.14, 23.12.

HRMS (ESI): Calc'd for $C_{24}H_{24}NO_2$[M+H$^+$] 358.18016, found 358.1791

(1*S*,2*R*,3*R*)-*N*-benzyl-2-hydroxy-2-(4-methoxyphenyl)-3-phenylcyclobutane-1-carboxamide (41)

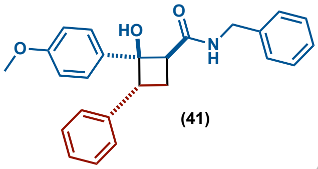

(41)

The title compound was prepared according to General Procedure H with compound **32** (50.0 mg, 0.10 mmol, 1 equiv.) and THF (0.5 mL), and an aqueous KH_2PO_4 solution (1.0 mL, 0.5 M, 5 equiv.). The yield of the reaction was obtained by NMR analysis of the unpurified reaction mixture with dibromomethane as an internal standard (69% NMR yield). The crude mixture was purified by flash chromatography (5-30 % Ethyl acetate in Hexane) to provide the product **41** (24.0 mg, 62% yield).

Physical State: white solid

^{1}H NMR (500 MHz, $CDCl_3$): δ 7.39 – 7.27 (m, 5H), 7.20 – 7.11 (m, 2H), 7.13 – 7.05 (m, 2H), 7.07 – 6.99 (m, 1H), 6.93 – 6.84 (m, 2H), 6.74 – 6.61 (m, 2H), 6.01 (t, *J* = 5.7 Hz, 1H), 5.00 (s, 1H), 4.54 (dd, *J* = 5.8, 1.7 Hz, 2H), 4.28 (t, *J* = 9.8 Hz, 1H), 3.70 (s, 3H), 3.51 (ddd, *J* = 9.4, 4.0, 1.0 Hz, 1H), 2.52 (ddd, *J* = 11.7, 9.9, 3.9 Hz, 1H), 2.41 (dt, *J* = 11.7, 9.6 Hz, 1H).

^{13}C NMR (126 MHz, $CDCl_3$): δ 174.11, 158.46, 139.26, 137.72, 133.35, 128.86, 127.88, 127.86, 127.78, 127.74, 127.40, 126.99, 126.08, 113.08, 80.13, 55.13, 48.24, 43.76, 22.85

HRMS (ESI): Calc'd for $C_{25}H_{26}NO_3[M+H^+]$ 388.1907, found 388.1901

Large Scale Set up-2:

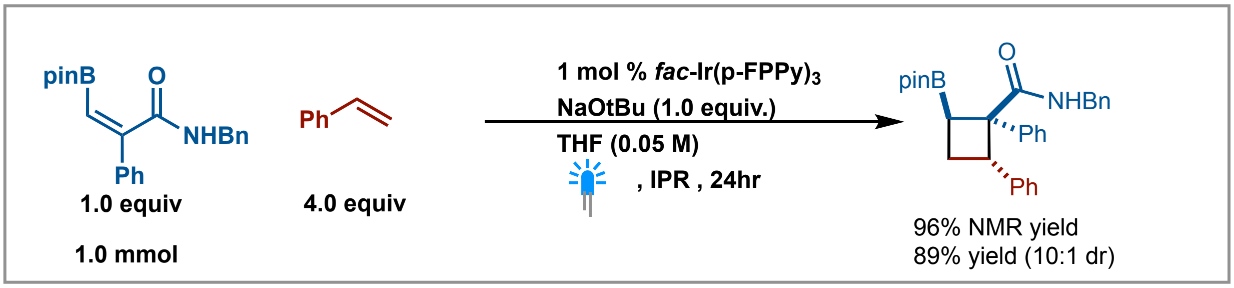

An oven-dried 50 ml glass vial equipped with a magnetic stir bar was charged with (*Z*)-*N*-benzyl-2-phenyl-3-(4,4,5,5-tetramethyl-1,3,2-dioxaborolan-2-yl)acrylamide (363 mg, 1.00 mmol, 1.0 equiv) and *fac*-Ir(p-Fppy)$_3$ (7.0 mg, 1 mol%, 0.01 equiv). The vial was transferred into a nitrogen-filled glovebox, and sodium *tert*-butoxide (96.1 mg, 1.0 mmol, 1.0 equiv) was added. The vial was sealed with a septum and removed from the glovebox. Dry THF (20 mL, 0.05 M) was added under nitrogen. The reaction mixture was stirred at room temperature under nitrogen for 30 min.

Styrene (0.46 ml, 4.0 mmol, 4.0 equiv.) was then added dropwise via syringe. The septum was quickly replaced with a screw cap, and the reaction mixture was irradiated with 450 nm light (light source: Integrated Photoreactor, IPR) at room temperature for 24 h with continuous stirring.

Upon completion, the reaction was quenched with saturated aqueous NH_4Cl (15 mL) and extracted with EtOAc (3 × 10 mL). The combined organic layers were washed with brine (40 mL), dried over anhydrous Na_2SO_4, filtered, and concentrated under reduced pressure.

The yield of the reaction was obtained by NMR analysis of the unpurified reaction mixture with dibromomethane as an internal standard (96% NMR yield). The crude mixture was purified by flash chromatography (1-5 % Et_2O in DCM) to provide the product (380 mg, 89% yield,10:1 dr) as a mixture of diastereoisomer).

Photoredox cross-coupling:

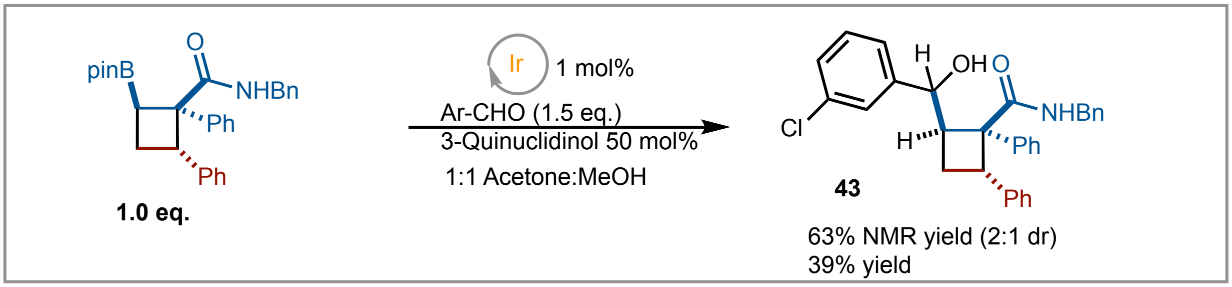

Following procedure is modified from the given literature[xiv]. In a flame-dried one dram vial equipped with a magnetic stir bar were added (2R,4S)-N-benzyl-1,2-diphenyl-4-(4,4,5,5-tetramethyl-1,3,2-dioxaborolan-2-yl)cyclobutane-1-carboxamide (0.10 mmol, 1.0 equiv), 4-chlorobenzaldehyde (21 mg, 0.15 mmol, 1.5 equiv), 3-quinuclidinol (6.36 mg, 0.05 mmol, 50 mol%), and {Ir[dF(CF_3)ppy]$_2$(dtbpy)}PF_6 (2.2 mg, 0.002 mmol, 2 mol%). The vial was evacuated and backfilled with nitrogen three times. A 1:1 mixture of methanol and acetone (2 mL) was added, and the resulting solution was purged with dry nitrogen for 10 min. The reaction mixture was then irradiated with 450 nm light (Integrated Photoreactor, IPR) at room temperature for 12 h with continuous stirring.

Upon completion, the reaction mixture was concentrated under reduced pressure. The reaction yield was determined by ^{1}H NMR analysis of the crude reaction mixture using dibromomethane as an internal standard (63% NMR yield, 2:1 dr). Purification by flash column chromatography (1–5% Et_2O in DCM) afforded the desired product **43** (19 mg, 39% yield).

Physical State: gummy solid

^{1}H NMR (600 MHz, cdcl$_3$): δ 7.33 (d, *J* = 6.3 Hz, 1H), 7.26 – 7.23 (m, 3H), 7.23 – 7.18 (m, 1H), 7.14 – 7.09 (m, 5H), 7.06 (tt, *J* = 7.7, 3.9 Hz, 4H), 6.95 (ddd, *J* = 7.3, 4.3, 2.2 Hz, 5H), 5.24 (s, 1H), 4.70 (t, *J* = 9.4 Hz, 1H), 4.45 (dd, *J* = 14.9, 5.9 Hz, 1H), 4.35 (dd, *J* = 15.0, 5.8 Hz, 1H), 2.76 (ddd, *J* = 11.9, 9.1, 3.0 Hz, 1H), 2.55 (dtd, *J* = 11.1, 9.1, 1.9 Hz, 1H), 2.39 – 2.21 (m, 2H).

^{13}C NMR (126 MHz, CDCl$_3$): δ 176.12, 140.21, 138.45, 137.52, 128.65, 128.61, 128.59, 128.07, 128.05, 127.65, 127.61, 127.33, 127.30, 126.86, 126.18, 77.28, 77.22, 77.02, 76.77, 59.04, 46.41, 43.60, 27.66, 21.49.

Oxidation:

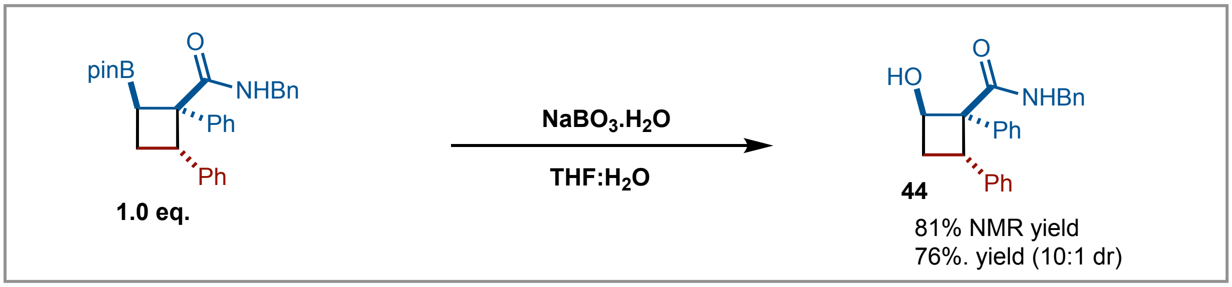

The title compound was prepared according to General Procedure G with compound **27** (50.0 mg, 0.10 mmol, 1 equiv.) and the yield of the reaction was obtained by NMR analysis of the unpurified reaction mixture with dibromomethane as an internal standard (81% NMR yield). The crude mixture was purified by flash chromatography (5-30 % Ethyl acetate in Hexane) to provide the product **44** (27 mg, 76% yield, 10:1 dr).

Physical State: Foamy solid

^{1}H NMR (500 MHz, CDCl$_3$): δ 7.40 – 7.26 (m, 7H), 7.26 – 7.17 (m, 6H), 7.08 – 7.04 (m, 2H), 6.29 (dd, *J* = 7.9, 1.2 Hz, 1H), 5.30 (ddd, *J* = 7.9, 4.7, 0.8 Hz, 1H), 4.89 (d, *J* = 14.7 Hz, 1H), 4.69 (d, *J* = 14.7 Hz, 1H), 3.91 (d, *J* = 6.1 Hz, 1H), 3.86 (ddd, *J* = 6.1, 4.5, 1.3 Hz, 1H).

^{13}C NMR (126 MHz, CDCl$_3$): δ 168.88, 142.12, 139.19, 137.03, 129.11, 128.69, 128.50, 128.21, 128.14, 127.74, 127.44, 127.18, 126.98, 109.21, 77.28, 77.23, 77.03, 76.77, 55.40, 49.61, 46.21.

HRMS (ESI): Calc'd for $C_{24}H_{24}NO_2$[M+H$^+$] 358.1801, found 362.1508 $C_{24}H_{21}NONa$ [M-OH+Na]+

Giese reaction:

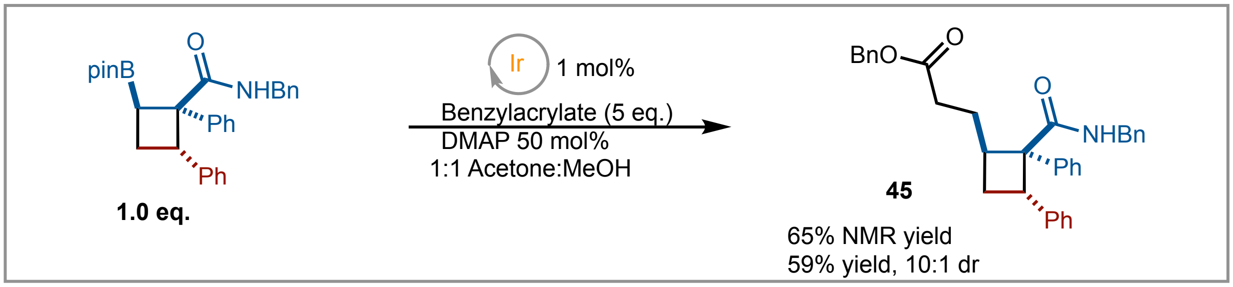

Following procedure is modified from the given literature[xv]. In a flame-dried 1 dram vial equipped with a magnetic stir bar were added (2R,4S)-N-benzyl-1,2-diphenyl-4-(4,4,5,5-tetramethyl-1,3,2-dioxaborolan-2-yl)cyclobutane-1-carboxamide (0.10 mmol, 1.0 equiv), [Ir(dF(CF3)ppy)2(dtbpy)]PF6 (2.24 mg, 2.0 mol%), and DMAP (6.11 mg, 0.05 mmol, 50 mol%). The vial was sealed with a rubber septum, evacuated, and backfilled with argon three times. Benzyl acrylate (66 µL, 0.50 mmol, 5.0 equiv) was then added, followed by degassed acetone/methanol (1:1, 1.0 mL), resulting in a clear yellow solution.
The reaction mixture was irradiated with 450 nm light (Integrated Photoreactor, IPR) at room temperature for 12 h with continuous stirring. Upon completion, the reaction mixture was concentrated under reduced pressure. The yield was determined by ^{1}H NMR analysis of the crude reaction mixture using dibromomethane as an internal standard (65% NMR yield). Purification by flash column chromatography (1–5% Et_2O in DCM) afforded the desired product (30 mg, 59% yield, 10:1 dr).

^{1}H NMR (500 MHz, CDCl$_3$): δ 7.36 – 7.22 (m, 5H), 7.14 (tdd, *J* = 8.7, 7.4, 3.6 Hz, 3H), 7.04 – 6.91 (m, 8H), 6.91 – 6.87 (m, 2H), 6.84 – 6.75 (m, 2H), 5.17 – 5.08 (m, 3H), 4.55 (dd, *J* = 9.3, 5.8 Hz, 1H), 4.38 – 4.26 (m, 2H), 3.25 – 3.15 (m, 1H), 2.43 (ddd, *J* = 16.0, 7.5, 5.7 Hz, 1H), 2.37 – 2.24 (m, 2H), 2.18 (ddd, *J* = 11.3, 9.1, 5.8 Hz, 1H), 2.04 (dtd, *J* = 15.7, 7.8, 4.3 Hz, 1H), 1.83 (dddd, *J* = 13.3, 11.2, 7.3, 5.7 Hz, 1H).

^{13}C NMR (126 MHz, CDCl$_3$): δ 174.33, 173.55, 141.95, 139.94, 138.60, 136.39, 129.20, 129.03, 128.93, 128.72, 128.54, 128.44, 128.13, 127.86, 127.67, 127.45, 127.12, 126.41, 66.74, 61.69, 44.45, 44.22, 39.21, 31.99, 29.28, 27.54.

HRMS (ESI): Calc'd for $C_{34}H_{34}NO_3$[M+H$^+$] 504.2526, found 504.2527

9. Mechanistic investigation

9.1 Photo-Switch Experiment:

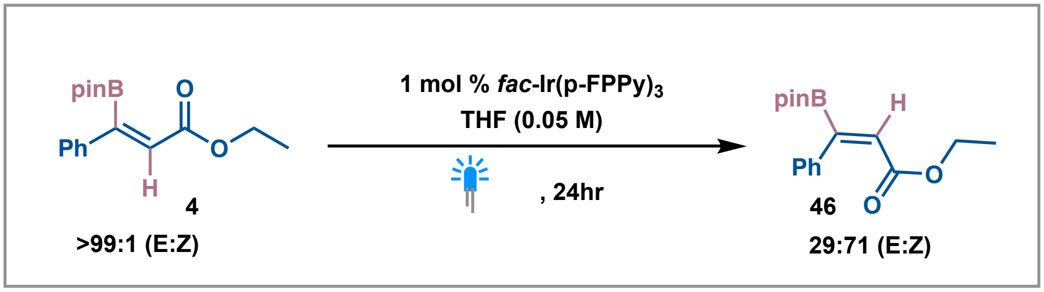

An oven-dried 2-dram vial equipped with a magnetic stir bar was charged with ethyl (*E*)-3-phenyl-3-(4,4,5,5-tetramethyl-1,3,2-dioxaborolan-2-yl)acrylate (0.1 mmol, 1.0 equiv.) and *fac*-Ir(p-Fppy)$_3$ (0.7 mg, 1 mol%, 0.01 equiv.). The vial was sealed with a septum and was evacuated and backfilled with nitrogen (three cycles). Dry THF (2.0 mL, 0.05 M) was added under nitrogen. The reaction mixture was irradiated with 450 nm light (light source: Integrated Photoreactor, IPR) at room temperature for 24 h with continuous stirring. the reaction mixture was concentrated under reduced pressure. E/Z ratio was determined by ^{1}H NMR using CH_2Br_2 as an internal standard.

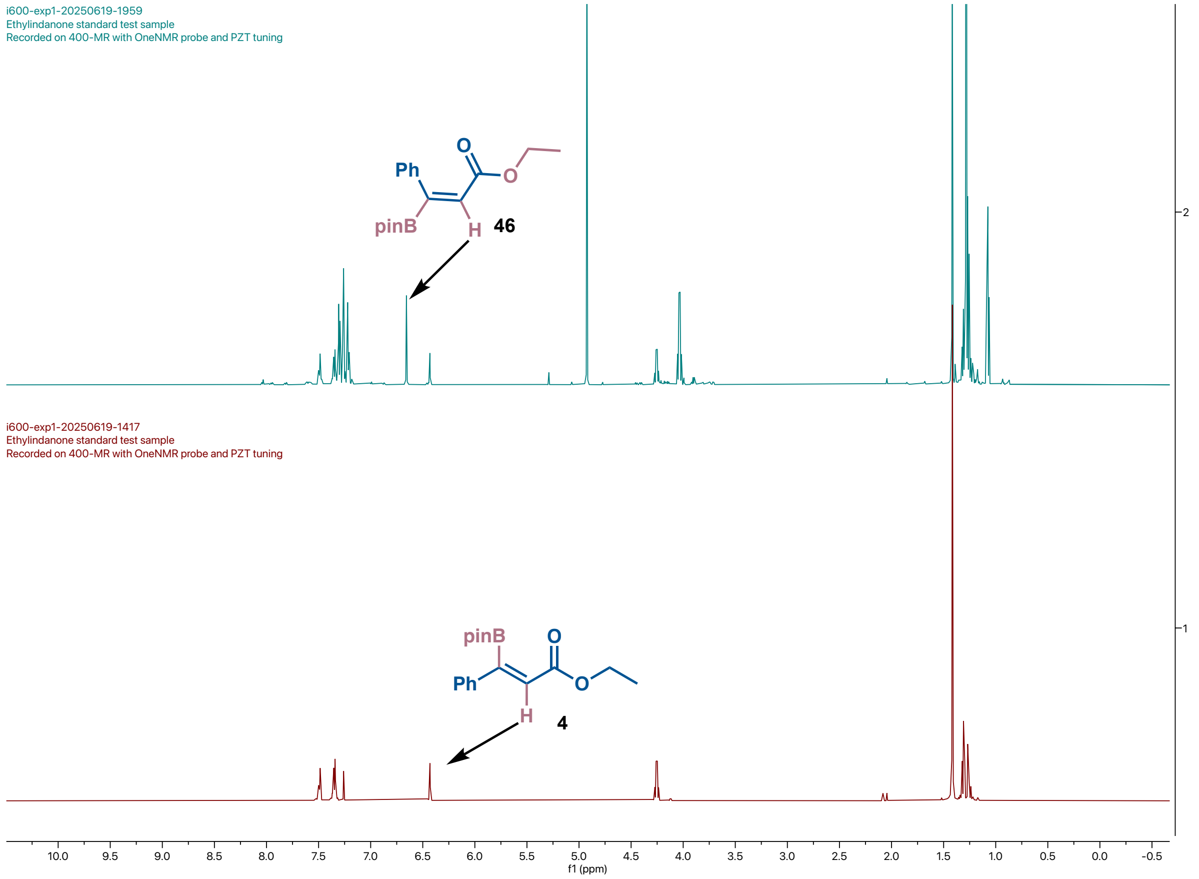

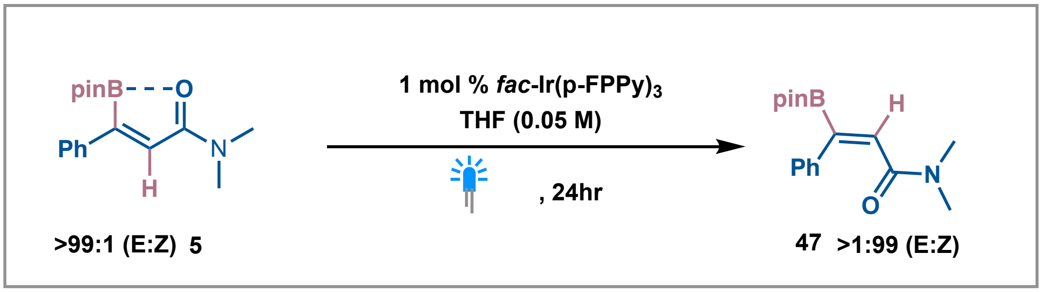

An oven-dried 2-dram vial equipped with a magnetic stir bar was charged with (*E*)-*N*,*N*-dimethyl-3-phenyl-3-(4,4,5,5-tetramethyl-1,3,2-dioxaborolan-2-yl)acrylamide (0.1 mmol, 1.0 equiv.) and *fac*-Ir(p-Fppy)$_3$ (0.7 mg, 1 mol%, 0.01 equiv.). The vial was sealed with a septum and was evacuated and backfilled with nitrogen (three cycles). Dry THF (2.0 mL, 0.05 M) was added under nitrogen. The reaction mixture was irradiated with 450 nm light (light source: Integrated Photoreactor, IPR) at room temperature for 24 h with continuous stirring. the reaction mixture was concentrated under reduced pressure. E/Z ratio was determined by ^{1}H NMR using CH_2Br_2 as an internal standard.

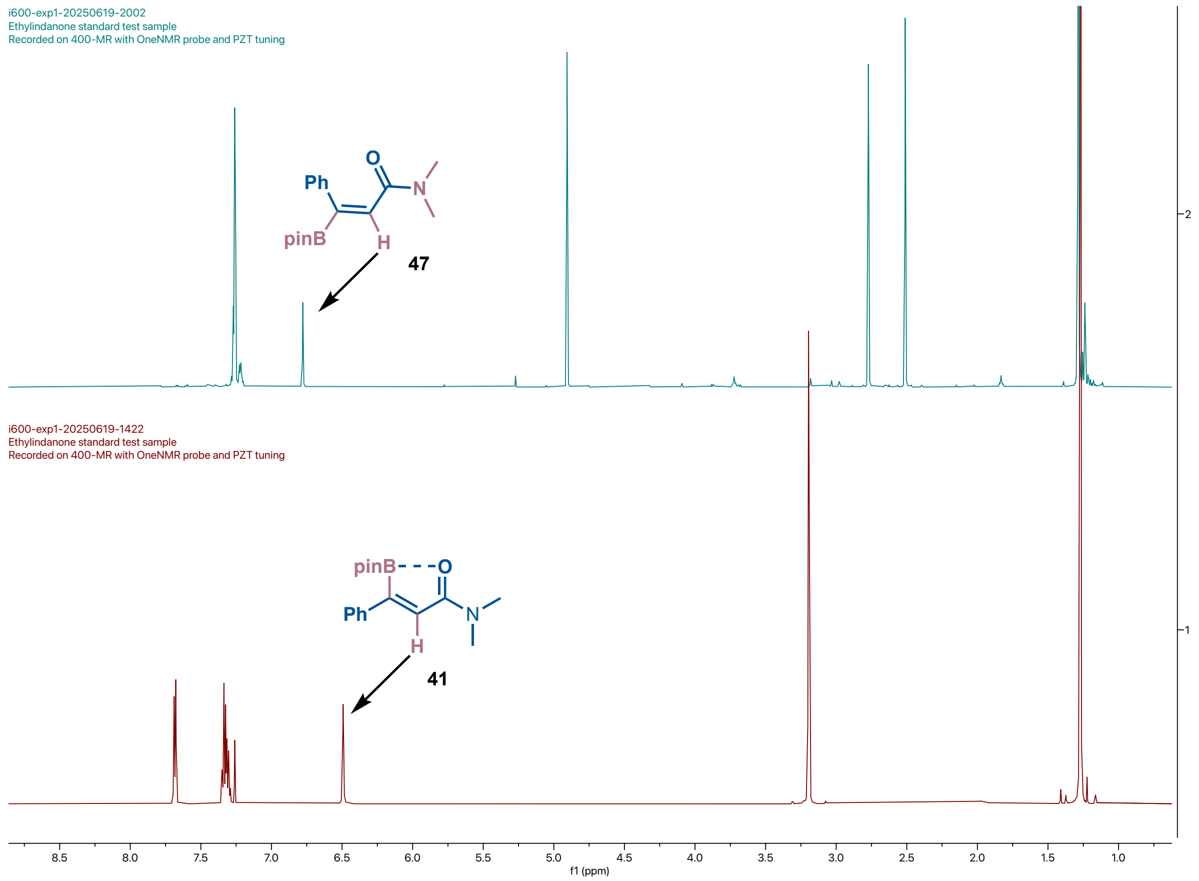

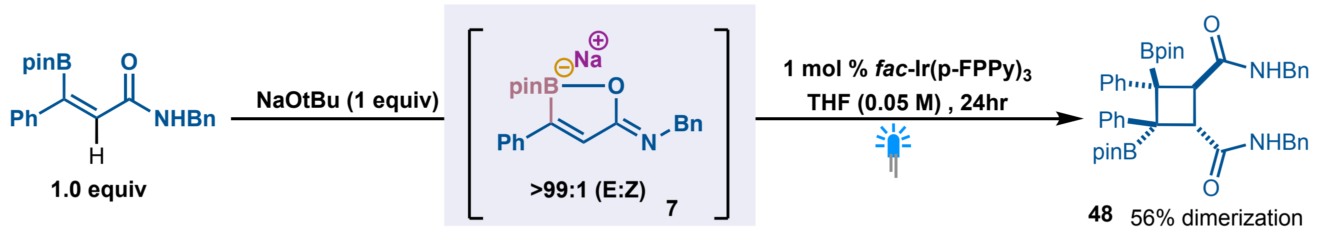

An oven-dried 2-dram vial equipped with a magnetic stir bar was charged with (*E*)-*N*-benzyl-3-phenyl-3-(4,4,5,5-tetramethyl-1,3,2-dioxaborolan-2-yl)acrylamide (36.3 mg, 0.10 mmol, 1.0 equiv.) and *fac*-Ir(p-Fppy)$_3$ (0.7 mg, 1 mol%, 0.01 equiv.). The vial was transferred into a nitrogen-filled glovebox, and sodium *tert*-butoxide (9.6 mg, 0.1 mmol, 1.0 equiv.) was added. The vial was sealed with a septum and removed from the glovebox. Dry THF (2.0 mL, 0.05 M) was added under nitrogen. The reaction mixture was stirred at room temperature under nitrogen for 30 min. The septum was quickly replaced with a screw cap, and the reaction mixture was irradiated with 450 nm light (light source: Integrated Photoreactor, IPR) at room temperature for 24 h with continuous stirring. No isomerization was observed by ^{1}H NMR.

The yield of the dimerization was obtained by NMR analysis of the unpurified reaction mixture with dibromomethane as an internal standard (56% NMR yield). The crude mixture was purified by flash chromatography (1-5 % Et$_2$O in DCM) to provide the product **48** (35.4 mg, 49% yield).

(1*S*,2*S*,3*R*,4*R*)-*N*^{1},*N*^{2}-dibenzyl-3,4-diphenyl-3,4-bis(4,4,5,5-tetramethyl-1,3,2-dioxaborolan-2-yl)cyclobutane-1,2-dicarboxamide (48)

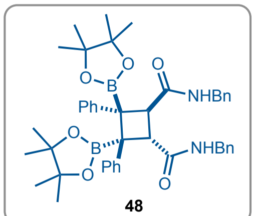

48

^{1}H NMR (500 MHz, CDCl$_3$): δ 7.59 – 7.48 (m, 2H), 7.36 – 7.28 (m, 2H), 7.27 – 7.24 (m, 7H), 7.22 (q, *J* = 3.8 Hz, 3H), 7.18 – 7.07 (m, 4H), 6.98 (td, *J* = 7.3, 1.6 Hz, 1H), 6.94 – 6.88 (m, 3H), 6.85 (dd, *J* = 7.9, 1.5 Hz, 1H), 6.42 (d, *J* = 6.0 Hz, 1H), 6.08 (s, 1H), 4.42 (dd, *J* = 14.7, 6.5 Hz, 1H), 4.34 (dd, *J* = 15.0, 6.9 Hz, 1H), 4.14 (dd, *J* = 14.7, 5.4 Hz, 1H), 3.90 (dd, *J* = 11.7, 5.5 Hz, 1H), 3.51 (d, *J* = 11.7 Hz, 1H), 2.83 (d, *J* = 5.5 Hz, 1H), 1.26 – 1.11 (m, 16H), 1.05 (s, 6H).

^{13}C NMR (126 MHz, CDCl$_3$): δ 175.86, 175.31, 149.14, 140.57, 138.00, 137.77, 131.31, 130.52, 128.70, 128.39, 128.00, 127.62, 127.54, 127.44, 127.23, 125.29, 124.70, 124.64, 82.70, 82.27, 55.69, 45.12, 43.75, 43.31, 24.88, 24.67, 24.64, 24.59. (Signal of carbon directly bonded to boron was not detected because of quadrupolar relaxation)

HRMS (ESI): Calc'd for $C_{44}H_{53}B_2N_2O_3$[M+H$^+$] 727.4084, found 727.4092

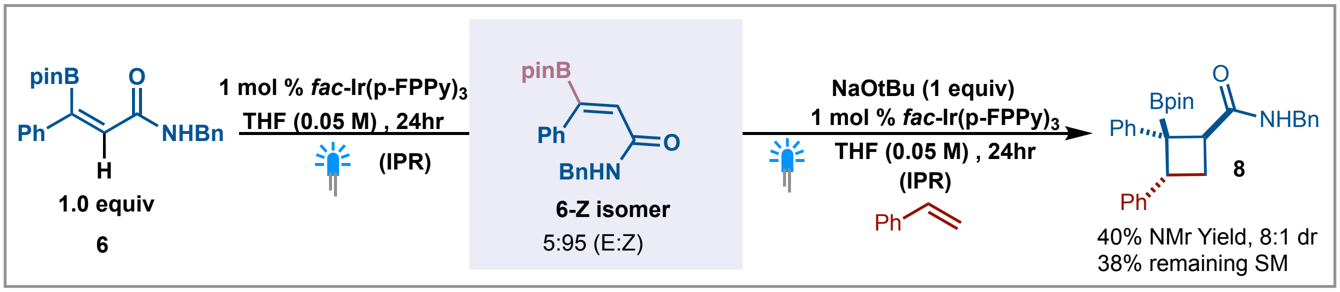

An oven-dried 2-dram vial equipped with a magnetic stir bar was charged with (*E*)-*N*-benzyl-3-phenyl-3-(4,4,5,5-tetramethyl-1,3,2-dioxaborolan-2-yl)acrylamide (72.6 mg, 0.20 mmol, 1.0 equiv.) and *fac*-Ir(p-Fppy)$_3$ (1.4 mg, 1 mol%, 0.01 equiv.). The vial was sealed with a septum and was evacuated and backfilled with nitrogen (three cycles). Dry THF (4.0 mL, 0.05 M) was added under nitrogen. The reaction mixture was irradiated with 450 nm light (light source: Integrated Photoreactor, IPR) at room temperature for 24 h with continuous stirring. the reaction mixture was concentrated under reduced pressure. E/Z ratio was determined by ^{1}H NMR using CH_2Br_2 as an internal standard. The vial containing 6-Z isomer and *fac*-Ir(p-Fppy)$_3$ transferred into a nitrogen-filled glovebox, and sodium *tert*-butoxide (19.2 mg, 0.2 mmol, 1.0 equiv.) was added. The vial was sealed with a septum and removed from the glovebox. Dry THF (4.0 mL, 0.05 M) was added under nitrogen. The reaction mixture was stirred at room temperature under nitrogen for 30 min. Styrene (92.0 µl, 0.800 mmol, 4 equiv.) was then added dropwise via syringe. The septum was quickly replaced with a screw cap, and the reaction mixture was irradiated with 450 nm light (light source: Integrated Photoreactor, IPR) at room temperature for 24 h with continuous stirring. The yield of the product 8 was obtained by NMR analysis of the unpurified reaction mixture with dibromomethane as an internal standard. It appears that photocycloaddition of 6-Z can takes place but is less efficient.

9.2 Boron NMR Experiment:

To investigate the potential coordination between boron and carbonyl 'O', we conducted a series of ^{11}B NMR experiments. **39, 41 and 45** was dissolved in CD_2Cl_2 and analyzed by ^{11}B NMR spectroscopy. The observed chemical shifts for *B* shows the weak coordination between the boron center and the oxygen atom in case of **39 and 41**. In contrast, a distinct coordination event was observed when **41** (*borate complex)* was employed as the boron source. Treatment of compound **SI 13** with NaO^{t}Bu, led to a pronounced downfield shift in the ^{11}B NMR spectrum. This notable change in chemical shift is consistent with strong coordination between the electron-deficient boron center and the oxygen lone pair of the carbonyl, highlighting the distinct reactivity of the Borate-based rigidified system.

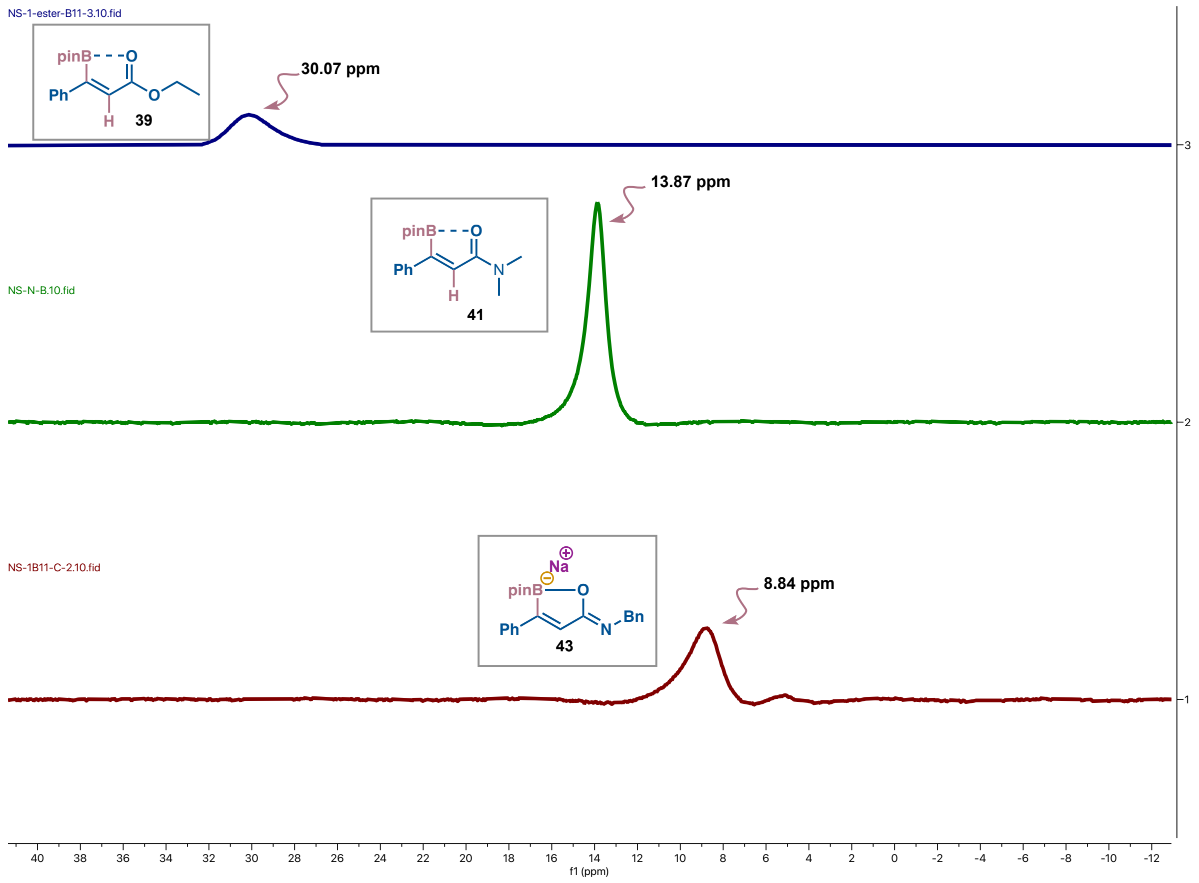

In addition, we provide NMR data for the borate complex to rule out any potential direct coordination of tert-butoxide to boron. Comparison of the ^{1}H NMR spectrum of **SI 13** with that of the intermediate generated by treating **SI 13** with NaOtBu shows the absence of the amide N–H signal in the crude spectrum (taken in CD_2Cl_2), suggesting deprotonation by NaOtBu. Furthermore, HRMS analysis did not reveal any mass corresponding to tert-butoxide coordination to boron. These results support the formation of the conformationally locked borate complex rather than a tert-butoxide-bound species.

HRMS (ESI): Calc'd for $C_{22}H_{26}BNO_3Na[M+Na^+]$ 386.250, found 386.189

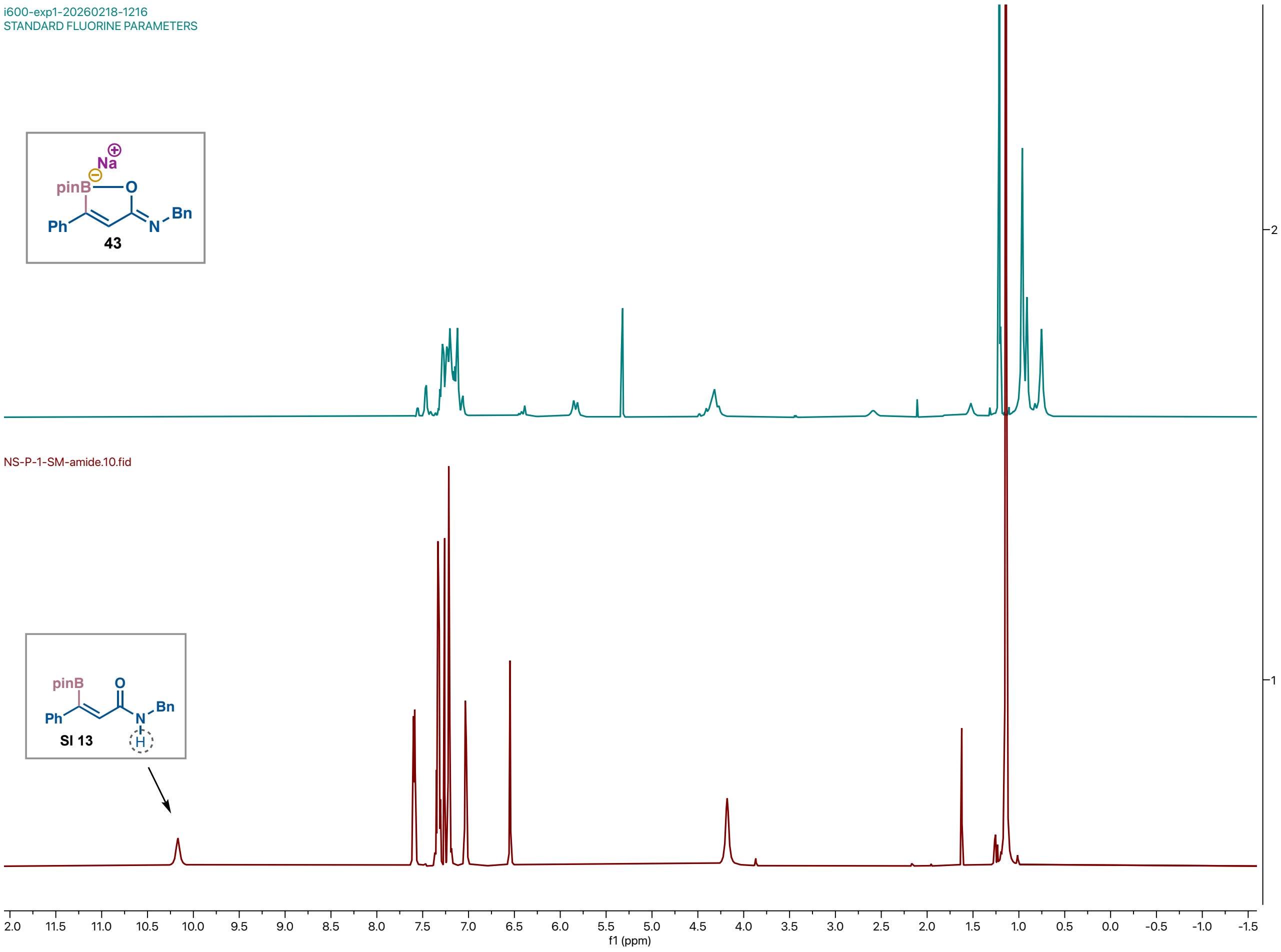

9.3 Stern Volmer Quenching Experiment:

Luminescence quenching experiments were conducted using an Edinburgh Instruments FLS-1000 fluorescence spectrophotometer. All sample preparations took place inside a nitrogen-filled glovebox to eliminate any exposure to moisture or oxygen. Quartz cuvettes with screw caps were used to ensure airtight sealing, and all measurements were carried out at room temperature.

A 0.3 mM solution of *fac*-Ir(p-Fppy)$_3$ in dry tetrahydrofuran (THF) served as the emissive probe. Samples were excited at 420 nm, and emission was monitored at 543 nm. The quencher compound was added in concentrations ranging from 0 to 25 mM, in 5 mM increments. Quenching behavior was analyzed by plotting the ratio I_0/I against quencher concentration, where I_0 corresponds to the emission intensity without quencher and I represents the intensity in its presence. Linear regression was used to interpret the data, and calculations were carried out.

To prepare the solutions, *fac*-Ir(p-Fppy)$_3$ (7.3 mg, 7.2 μmol) was dissolved in 4.0 mL of anhydrous THF, yielding a 1.8 mM stock solution. Separately, compound **SI 13** (, 0.30 mmol) and NaOtBu (28.5 mg, 0.3 mmol) was dissolved in 4.0 ml of dry THF to make 75 mM stock solutions of Borate complex (**7**). Furthermore, Styrene was dissolved in 4.0 mL of dry THF to make 75 mM stock solutions.

For each individual quenching measurement, 0.5 mL of the *fac*-Ir(p-Fppy)$_3$ stock solution was added to a quartz cuvette, followed by a precise volume of the appropriate quencher stock. The total volume was adjusted to 3.0 mL with THF. All cuvettes were sealed within the glovebox before data collection. Luminescence readings were consistently performed at room temperature.

Test	[Ir]-catalyst	Quencher	THF	Final Volume
1	0.5 mL (0.3 mM)	0 mL (0 mM)	2.5 mL	3.0 mL
2	0.5 mL (0.3 mM)	0.2 mL (5 mM)	2.3 mL	3.0 mL
3	0.5 mL (0.3 mM)	0.4 mL (10 mM)	2.1 mL	3.0 mL
4	0.5 mL (0.3 mM)	0.6 mL (15 mM)	1.9 mL	3.0 mL
5	0.5 mL (0.3 mM)	0.8 mL (20 mM)	1.7 mL	3.0 mL
6	0.5 mL (0.3 mM)	1.0 mL (25 mM)	1.5 mL	3.0 mL

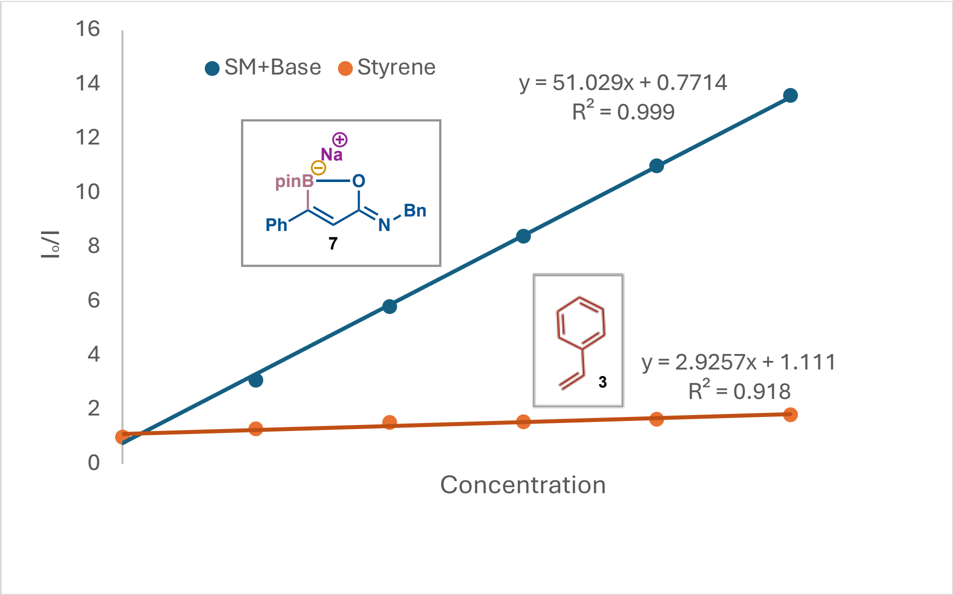

*Figure S1 Stern-Volmer quenching experiments of fac-Ir(p-Fppy)$_3$ by **(SM+Base)** and **(Styrene)***

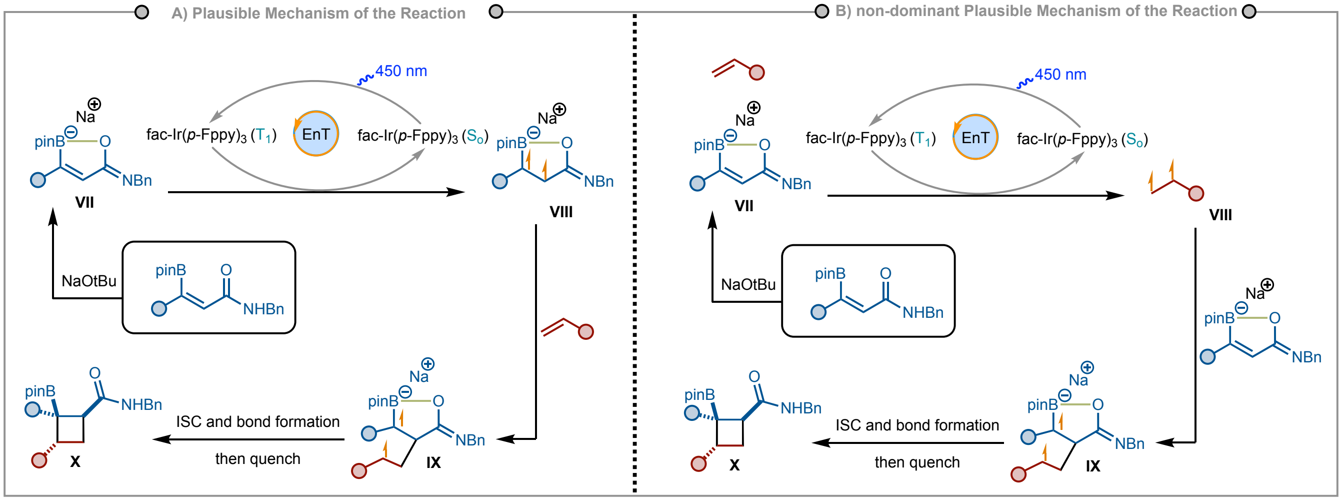

Stern-Volmer experiments suggest that the most plausible mechanism involves quenching of the excited state of the photocatalyst by the cyclic boronate (mechanism A). Although minor quenching by the alkene at high styrene concentrations cannot be fully excluded, this pathway appears to be non-dominant. The less favored mechanistic scenario is depicted accordingly (mechanism B).

10. Computational Details

All structures were optimized with a suite of Gaussian 09 rev. E.01[1] software using the wB97X-D functional and a def2-TZVPP basis set with the SMD solvent model for THF. All structures were checked for the absence of imaginary vibrational modes. Triplet energies were calculated by taking the difference in free energies between the singlet and triplet optimized structures.

VII (singlet)

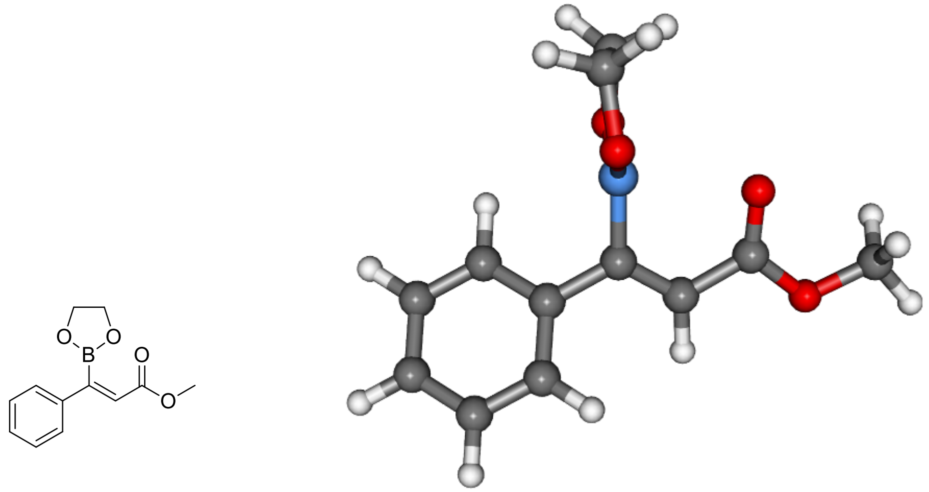

n(min) = 39.3895
wB97XD E(scf) = -791.007393969
Zero-point correction= 0.239909 (Hartree/Particle)
Thermal correction to Energy= 0.255466
Thermal correction to Enthalpy= 0.256410
Thermal correction to Gibbs Free Energy= 0.195006
Sum of electronic and zero-point Energies= -790.767485
Sum of electronic and thermal Energies= -790.751928
Sum of electronic and thermal Enthalpies= -790.750984
Sum of electronic and thermal Free Energies= -790.812388

6 4.231146 -2.208495 -0.139938
8 2.806936 -2.313509 -0.164813
6 2.128418 -1.172284 -0.061563
6 0.673723 -1.390934 -0.096993
6 -0.161732 -0.348657 -0.033928
6 -1.629232 -0.531900 -0.031194
6 -2.457942 0.448735 -0.580095
6 -3.833873 0.284036 -0.604478
6 -4.408216 -0.855448 -0.061726
6 -3.597197 -1.828373 0.507023
6 -2.222250 -1.668852 0.522929
1 -1.603202 -2.424141 0.989369
1 -4.039116 -2.712697 0.947654
1 -5.482918 -0.982800 -0.073802
1 -4.458247 1.050237 -1.045494

1 -2.022412 1.341564 -1.011787
5 0.400649 1.126386 0.058371
8 0.625662 1.913225 -1.036868
6 1.139687 3.160219 -0.558118
6 0.837485 3.153554 0.946352
8 0.506010 1.793233 1.245452
1 1.694723 3.457171 1.545144
1 -0.015625 3.784295 1.200964
1 2.210945 3.197814 -0.760536
1 0.649338 3.979051 -1.082017
1 0.330495 -2.413951 -0.185833
8 2.657936 -0.088390 0.048932
1 4.568686 -1.776294 0.801409
1 4.606248 -3.223032 -0.240514
1 4.585377 -1.595219 -0.967660

VII (triplet)

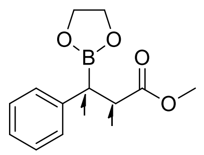

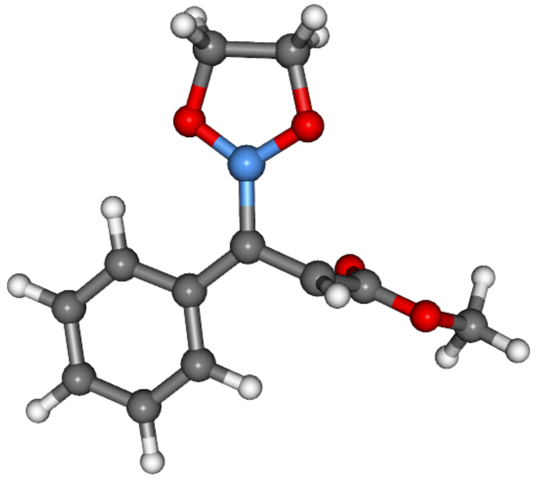

n(min) = 37.5366
UwB97XD E(scf) = -790.931736522
Zero-point correction= 0.237302 (Hartree/Particle)
Thermal correction to Energy= 0.253068
Thermal correction to Enthalpy= 0.254012
Thermal correction to Gibbs Free Energy= 0.191014
Sum of electronic and zero-point Energies= -790.694435
Sum of electronic and thermal Energies= -790.678668
Sum of electronic and thermal Enthalpies= -790.677724
Sum of electronic and thermal Free Energies= -790.740723

6 -3.480553 -3.046012 0.363722
8 -2.631726 -2.289237 -0.494921
6 -1.743922 -1.485138 0.113603
6 -0.918340 -0.775181 -0.841212
6 0.110297 0.168993 -0.408748

6 1.447733 -0.299206 -0.213825
6 2.495434 0.557462 0.195681
6 3.774563 0.074261 0.381086
6 4.063334 -1.269914 0.167145
6 3.050516 -2.133037 -0.238365
6 1.768428 -1.660475 -0.425859
1 0.991115 -2.346173 -0.739974
1 3.265175 -3.180470 -0.406692
1 5.068393 -1.642570 0.315789
1 4.558091 0.750836 0.697506
1 2.287875 1.603205 0.367421
5 -0.398398 1.620630 -0.195624
8 0.335152 2.691513 0.244356
6 -0.505824 3.850002 0.195727
6 -1.915644 3.296501 -0.047346
8 -1.707516 1.940837 -0.451167
1 -2.522715 3.302562 0.859108
1 -2.446285 3.830814 -0.833652
1 -0.423331 4.392038 1.136024
1 -0.167642 4.493562 -0.617501
1 -1.116110 -0.927117 -1.896351
8 -1.655507 -1.383033 1.320187
1 -4.095747 -2.389688 0.979152
1 -4.112320 -3.641437 -0.289975
1 -2.893861 -3.698891 1.009949

VIII (singlet)

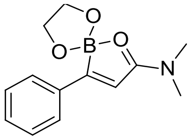

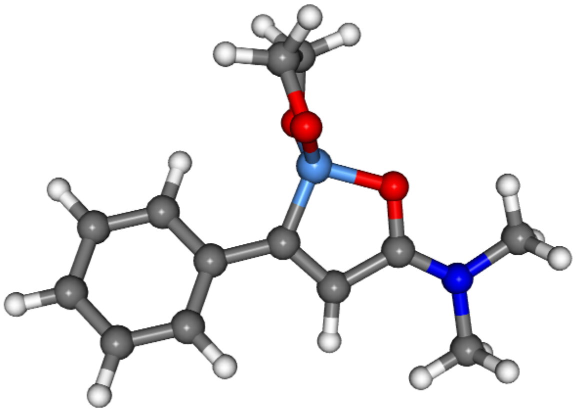

n(min) = 37.9093
wB97XD E(scf) = -810.458409505
Zero-point correction= 0.280818 (Hartree/Particle)
Thermal correction to Energy= 0.297357
Thermal correction to Enthalpy= 0.298301
Thermal correction to Gibbs Free Energy= 0.235544
Sum of electronic and zero-point Energies= -810.177592
Sum of electronic and thermal Energies= -810.161053

Sum of electronic and thermal Enthalpies= -810.160109
Sum of electronic and thermal Free Energies= -810.222865

6 4.427885 -0.444826 -0.026866
7 3.197912 -1.217530 -0.048847
6 2.018467 -0.628453 -0.010485
6 0.726781 -1.312200 -0.057852
6 -0.257070 -0.401970 -0.021213
6 -1.688950 -0.714662 -0.030071
6 -2.599909 0.245851 -0.476175
6 -3.958761 -0.024752 -0.506080
6 -4.433919 -1.253432 -0.071272
6 -3.541459 -2.212054 0.391467
6 -2.183140 -1.946662 0.409261
1 -1.497651 -2.695023 0.786252
1 -3.908525 -3.167236 0.744912
1 -5.496045 -1.461947 -0.086110
1 -4.649994 0.727285 -0.864360
1 -2.226043 1.202314 -0.817461
5 0.386156 1.090334 0.061482
8 0.146065 1.946308 -1.063938
6 0.187981 3.273092 -0.579402
6 -0.255532 3.149911 0.876194
8 0.131117 1.845538 1.254376
1 0.220712 3.890895 1.522432
1 -1.342528 3.263269 0.969476
1 1.206633 3.675252 -0.646497
1 -0.471110 3.908938 -1.173411
1 0.622319 -2.383538 -0.131629
8 1.935974 0.641273 0.072669
6 3.383770 -2.650742 -0.177344
1 3.868317 -2.871878 -1.129498
1 4.024984 -3.002195 0.631285
1 2.440487 -3.180853 -0.129645
1 4.211021 0.605137 0.130425
1 5.066744 -0.809166 0.778097
1 4.952408 -0.569789 -0.975581

VIII (triplet)

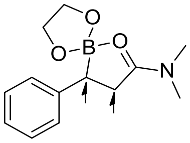

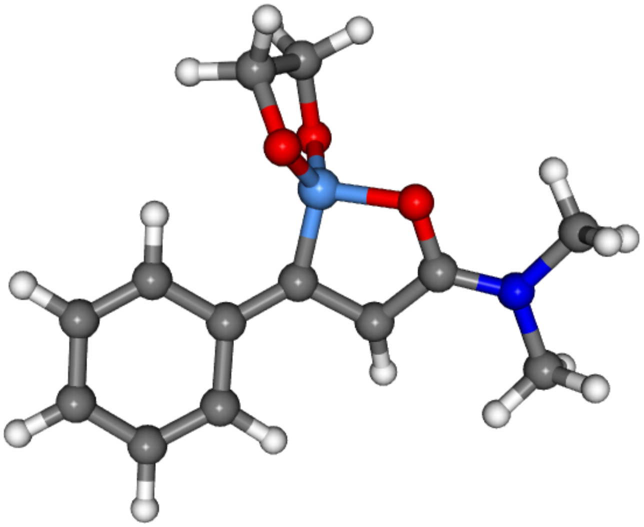

n(min) = 33.7126
UwB97XD E(scf) = -810.363913606
Zero-point correction= 0.276546 (Hartree/Particle)
Thermal correction to Energy= 0.293563
Thermal correction to Enthalpy= 0.294507
Thermal correction to Gibbs Free Energy= 0.230097
Sum of electronic and zero-point Energies= -810.087368
Sum of electronic and thermal Energies= -810.070351
Sum of electronic and thermal Enthalpies= -810.069406
Sum of electronic and thermal Free Energies= -810.133817

6 4.309108 -0.660670 0.432089
7 3.086154 -1.369020 0.106466
6 1.925195 -0.727498 0.037376
6 0.691254 -1.298965 -0.401640
6 -0.371218 -0.290317 -0.248225
6 -1.710638 -0.613006 -0.104669
6 -2.697505 0.412515 0.073202
6 -4.026992 0.106302 0.198488
6 -4.462299 -1.225813 0.161959
6 -3.528825 -2.249144 -0.004618
6 -2.189830 -1.963775 -0.136087
1 -1.483257 -2.771633 -0.272216
1 -3.862072 -3.279131 -0.034886
1 -5.514381 -1.456704 0.262837
1 -4.751029 0.900820 0.329252
1 -2.368198 1.441720 0.115631
5 0.378474 1.131869 -0.048280
8 0.526307 1.951656 -1.217565
6 0.676262 3.278766 -0.757969
6 -0.117621 3.302117 0.543939
8 -0.019710 1.982028 1.035293

1 0.282864 4.011367 1.271600
1 -1.168753 3.558208 0.357557
1 1.734140 3.505397 -0.573236
1 0.297606 3.981514 -1.502293
1 0.621202 -2.234719 -0.934188
8 1.846129 0.514188 0.349634
6 3.212437 -2.796824 -0.113965
1 3.627356 -2.995288 -1.103782
1 3.888111 -3.206407 0.636565
1 2.253869 -3.295484 -0.011982
1 4.180631 0.403008 0.261212
1 4.577229 -0.826316 1.477628
1 5.111445 -1.037667 -0.201731

IX (singlet)

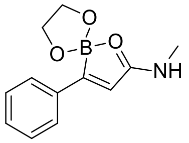

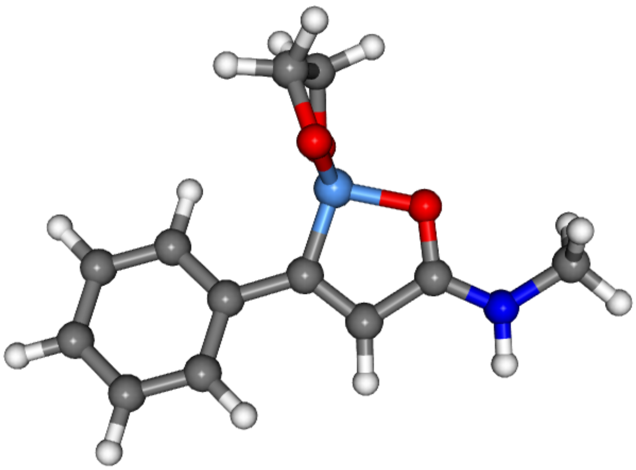

n(min) = 39.1553
wB97XD E(scf) = -771.15181049
Zero-point correction= 0.252217 (Hartree/Particle)
Thermal correction to Energy= 0.267391
Thermal correction to Enthalpy= 0.268335
Thermal correction to Gibbs Free Energy= 0.208736
Sum of electronic and zero-point Energies= -770.899594
Sum of electronic and thermal Energies= -770.884420
Sum of electronic and thermal Enthalpies= -770.883476
Sum of electronic and thermal Free Energies= -770.943075

6 4.453151 -1.587082 -0.037655
7 3.070746 -2.017604 -0.098715
6 2.048741 -1.190409 -0.051990
6 0.662362 -1.634872 -0.101831
6 -0.145469 -0.566451 -0.045269
6 -1.610763 -0.608987 -0.040787
6 -2.334117 0.547106 -0.343009

6 -3.720223 0.536911 -0.350101
6 -4.409245 -0.626364 -0.041527
6 -3.704052 -1.781241 0.273689
6 -2.320282 -1.772644 0.272911
1 -1.783960 -2.675716 0.535543
1 -4.236190 -2.689854 0.524927
1 -5.491800 -0.633998 -0.040001
1 -4.262744 1.441625 -0.592879
1 -1.798211 1.454347 -0.588162
5 0.754868 0.790087 0.041530
8 0.679775 1.674750 -1.079904
6 0.999488 2.964232 -0.594693
6 0.558049 2.931204 0.866694
8 0.654315 1.570540 1.236391
1 1.193029 3.547049 1.507492
1 -0.476886 3.276561 0.976471
1 2.077998 3.145902 -0.676006
1 0.477040 3.723484 -1.179275
1 0.407526 -2.683665 -0.182026
8 2.223708 0.061322 0.043047
1 2.869382 -3.000831 -0.171191
1 4.656573 -1.080032 0.905230
1 5.088252 -2.465136 -0.113266
1 4.682210 -0.908266 -0.858359

IX (triplet)

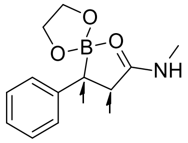

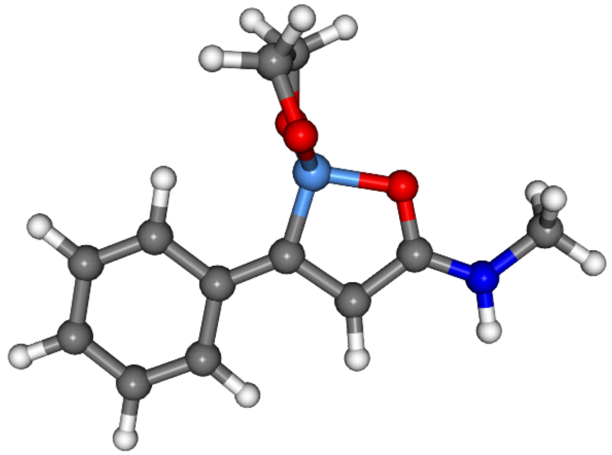

n(min) = 29.7480
UwB97XD E(scf) = -771.056101991
Zero-point correction= 0.247671 (Hartree/Particle)
Thermal correction to Energy= 0.263503
Thermal correction to Enthalpy= 0.264447
Thermal correction to Gibbs Free Energy= 0.202479
Sum of electronic and zero-point Energies= -770.808431

Sum of electronic and thermal Energies= -770.792599
Sum of electronic and thermal Enthalpies= -770.791655
Sum of electronic and thermal Free Energies= -770.853623

6 4.399231 -1.674439 -0.064651
7 3.021732 -2.106567 0.017836
6 1.995953 -1.264208 0.020911
6 0.635391 -1.684683 0.087759
6 -0.229146 -0.490439 0.045096
6 -1.603443 -0.552998 0.013498
6 -2.387652 0.657427 -0.019893
6 -3.753052 0.619602 -0.047166
6 -4.438892 -0.607001 -0.046648
6 -3.714103 -1.801246 -0.018340
6 -2.341559 -1.790816 0.012062
1 -1.806403 -2.730128 0.032725
1 -4.241352 -2.747060 -0.019837
1 -5.520276 -0.626088 -0.069911
1 -4.314645 1.545099 -0.071086
1 -1.867127 1.605586 -0.028918
5 0.730700 0.808916 0.015186
8 0.654467 1.652483 -1.137296
6 1.106119 2.930015 -0.738912
6 0.763267 2.997114 0.748068
8 0.773946 1.651904 1.175776
1 1.485350 3.586720 1.317747
1 -0.231149 3.433617 0.904480
1 2.187869 3.022537 -0.896418
1 0.606722 3.704167 -1.324505
1 0.350955 -2.721995 0.186870
8 2.179974 -0.004617 -0.035771
1 2.817374 -3.090836 0.052841
1 4.643519 -1.004279 0.759608
1 5.038138 -2.551543 -0.012721
1 4.585629 -1.152533 -1.004045

X (singlet)

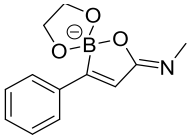

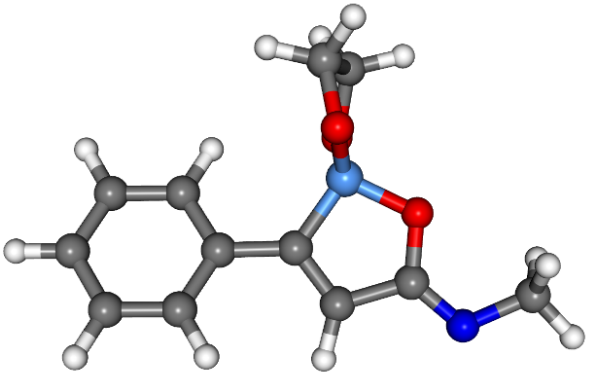

n(min) = 30.6111
wB97XD E(scf) = -770.64967887
Zero-point correction= 0.238013 (Hartree/Particle)
Thermal correction to Energy= 0.252688
Thermal correction to Enthalpy= 0.253633
Thermal correction to Gibbs Free Energy= 0.194926
Sum of electronic and zero-point Energies= -770.411666
Sum of electronic and thermal Energies= -770.396990
Sum of electronic and thermal Enthalpies= -770.396046
Sum of electronic and thermal Free Energies= -770.454753
6 4.401042 -1.584787 -0.042046
7 3.063894 -2.136669 -0.136698
6 2.103071 -1.291468 -0.077761
6 0.682063 -1.677993 -0.141401
6 -0.103651 -0.599559 -0.058989
6 -1.572778 -0.613423 -0.052535
6 -2.280186 0.536786 -0.413559
6 -3.666847 0.555053 -0.413738
6 -4.380985 -0.575064 -0.042127
6 -3.694045 -1.723727 0.330221
6 -2.309058 -1.741372 0.325816
1 -1.787373 -2.638311 0.636034
1 -4.241172 -2.608399 0.631941
1 -5.463630 -0.560213 -0.035534
1 -4.191795 1.457398 -0.702242
1 -1.722693 1.416388 -0.708223
5 0.865790 0.710955 0.050618
8 0.749908 1.646209 -1.066888
6 0.995415 2.931456 -0.559164
6 0.513568 2.855372 0.886948
8 0.732149 1.521369 1.259016
1 1.061243 3.536232 1.546063
1 -0.555114 3.107306 0.953659
1 2.068415 3.170269 -0.595793
1 0.460539 3.683307 -1.146475
1 0.399594 -2.719295 -0.247570

8 2.237526 0.013219 0.053654
1 4.607042 -0.848888 -0.827627
1 4.572768 -1.079466 0.915444
1 5.133659 -2.388440 -0.132114

X (triplet)

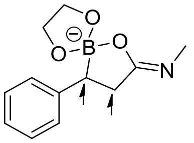

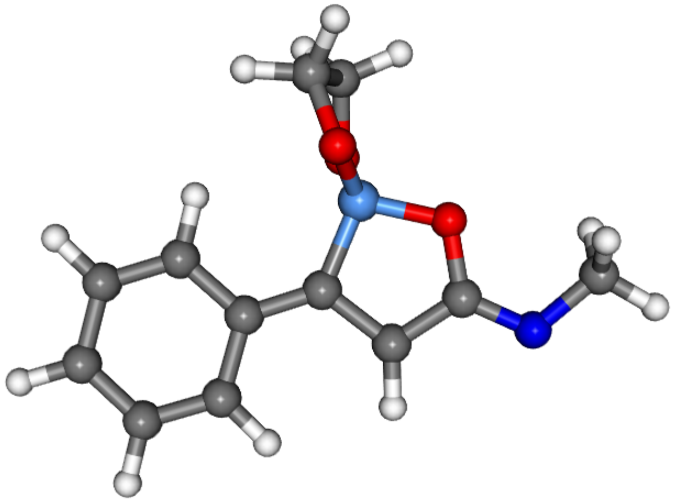

n(min) = 39.7625
UwB97XD E(scf) = -770.557813464
Zero-point correction= 0.233818 (Hartree/Particle)
Thermal correction to Energy= 0.248821
Thermal correction to Enthalpy= 0.249765
Thermal correction to Gibbs Free Energy= 0.190066
Sum of electronic and zero-point Energies= -770.323995
Sum of electronic and thermal Energies= -770.308992
Sum of electronic and thermal Enthalpies= -770.308048
Sum of electronic and thermal Free Energies= -770.367747

6 4.319605 -1.676206 0.122270
7 2.995410 -2.234584 -0.001311
6 2.014995 -1.365061 0.003620
6 0.660188 -1.749822 -0.109596
6 -0.192694 -0.538519 -0.068054
6 -1.571592 -0.554438 -0.039062
6 -2.318880 0.674820 0.011162
6 -3.687046 0.681280 0.051225
6 -4.413801 -0.521022 0.042092
6 -3.723635 -1.734199 -0.012060
6 -2.348915 -1.765114 -0.053539
1 -1.841122 -2.719359 -0.096313
1 -4.277813 -2.665360 -0.022018
1 -5.495299 -0.506670 0.076088

1 -4.216916 1.625514 0.091162
1 -1.769297 1.606397 0.016603
5 0.849919 0.712519 0.000589
8 0.839652 1.573760 -1.180153
6 1.213731 2.856691 -0.754042
6 0.691149 2.938238 0.678632
8 0.733288 1.617525 1.145422
1 1.302363 3.592182 1.308909
1 -0.338440 3.326939 0.694953
1 2.307075 2.977270 -0.771428
1 0.780317 3.618293 -1.408437
1 0.364939 -2.780712 -0.238585
8 2.184255 -0.054630 0.115004
1 4.455438 -1.155665 1.078324
1 5.061249 -2.473256 0.059967
1 4.533847 -0.939209 -0.661075

References

1. Cite this work as: Gaussian 09, Revision E.01, M. J. Frisch, G. W. Trucks, H. B. Schlegel, G. E. Scuseria, M. A. Robb, J. R. Cheeseman, G. Scalmani, V. Barone, B. Mennucci, G. A. Petersson, H. Nakatsuji, M. Caricato, X. Li, H. P. Hratchian, A. F. Izmaylov, J. Bloino, G. Zheng, J. L. Sonnenberg, M. Hada, M. Ehara, K. Toyota, R. Fukuda, J. Hasegawa, M. Ishida, T. Nakajima, Y. Honda, O. Kitao, H. Nakai, T. Vreven, J. A. Montgomery, Jr., J. E. Peralta, F. Ogliaro, M. Bearpark, J. J. Heyd, E. Brothers, K. N. Kudin, V. N. Staroverov, T. Keith, R. Kobayashi, J. Normand, K. Raghavachari, A. Rendell, J. C. Burant, S. S. Iyengar, J. Tomasi, M. Cossi, N. Rega, J. M. Millam, M. Klene, J. E. Knox, J. B. Cross, V. Bakken, C. Adamo, J. Jaramillo, R. Gomperts, R. E. Stratmann, O. Yazyev, A. J. Austin, R. Cammi, C. Pomelli, J. W. Ochterski, R. L. Martin, K. Morokuma, V. G. Zakrzewski, G. A. Voth, P. Salvador, J. J. Dannenberg, S. Dapprich, A. D. Daniels, O. Farkas, J. B. Foresman, J. V. Ortiz, J. Cioslowski, and D. J. Fox, Gaussian, Inc., Wallingford CT, 2013.

11. X-ray structure

INDIANA UNIVERSITY DEPARTMENT OF CHEMISTRY

Molecular Structure Center

Report No. 24066
(NS-1-194-2+2)

$C_{31}H_{33}BN_2O_3$

Prepared for
Neetu Sharma and Professor Kevin Brown

by M. Pink, April 1, 2024

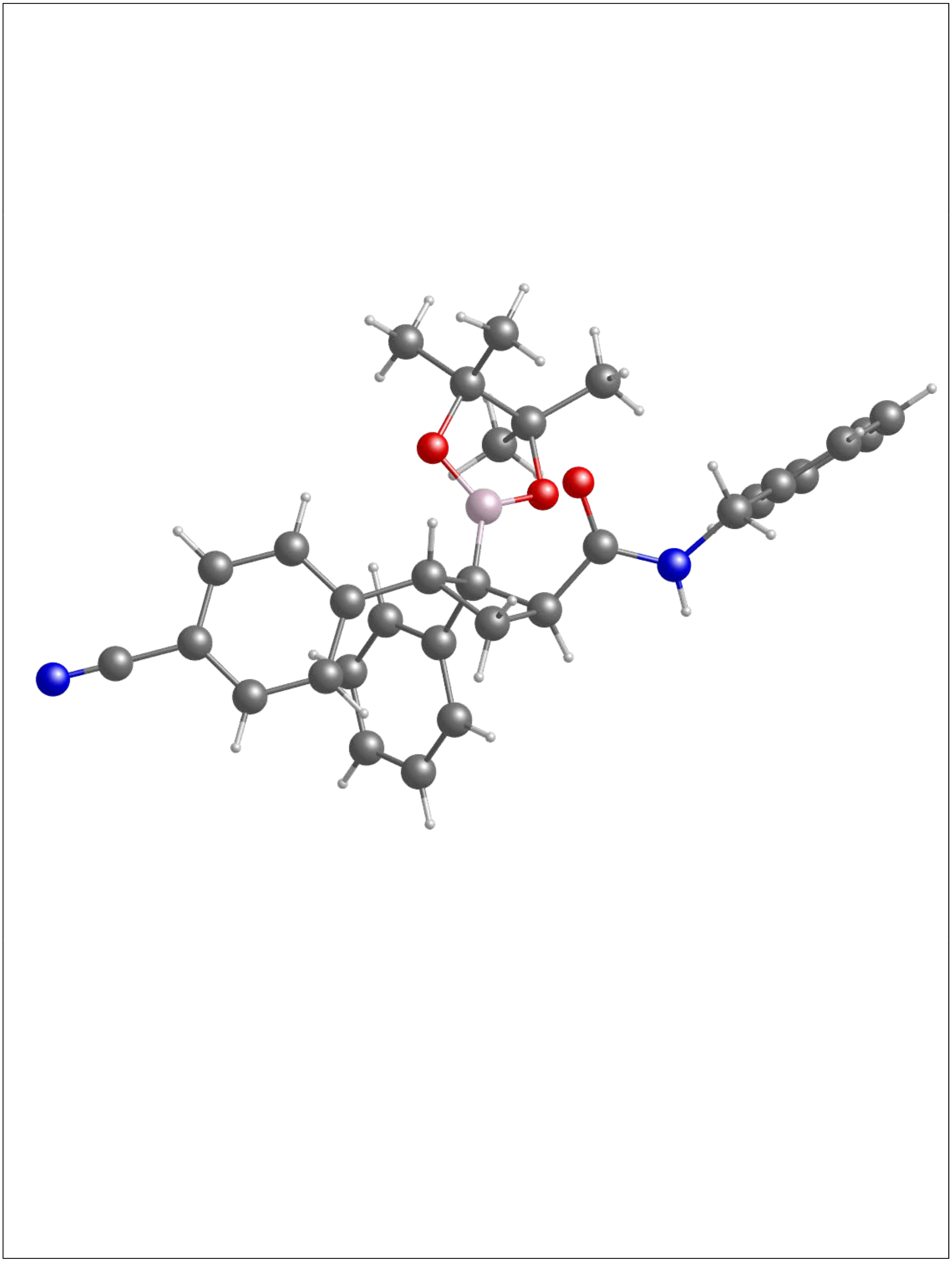

The sample was submitted by Neetu Sharma (research group of Kevin Brown, Department of Chemistry, Indiana University). A colorless crystal (approximate dimensions $0.127 \times 0.107 \times 0.049$ mm^3) was placed onto the tip of a MiTeGen loop and mounted on a Bruker Venture D8 diffractometer equipped with a PhotonIII detector at 173(2) K.

Data collection

The data collection was carried out using Mo Kα radiation (graphite monochromator) with a frame time of 3, 60, and 90 seconds and a detector distance of 4.00 cm. A collection strategy was calculated and complete data to a resolution of 0.84 Å (fourteen sets of frames) were collected with 1º ω and φ scans. A total of 2210 frames were collected. The total exposure time was 22.34 hours. The frames were integrated with the Bruker SAINT software package[1] using a narrow-frame algorithm. The integration of the data using a monoclinic unit cell yielded a total of 89925 reflections to a maximum θ angle of 25.09° (0.84 Å resolution), of which 4791 were independent (average redundancy 18.770, completeness = 99.8%, R_{int} = 10.99%, R_{sig} = 3.86%) and 3733 (77.92%) were greater than $2\sigma(F^2)$. The final cell constants of a = 21.8877(18) Å, b = 14.1739(11) Å, c = 8.6957(6) Å, β = 90.179(3)°, volume = 2697.7(4) Å^3, are based upon the refinement of the XYZ-centroids of 9869 reflections above 20 $\sigma(I)$ with $4.702° < 2\theta < 49.74°$. Data were corrected for absorption effects using the Multi-Scan method (SADABS[2]). The ratio of minimum to maximum apparent transmission was 0.749. The calculated minimum and maximum transmission coefficients (based on crystal size) are 0.9900 and 0.9960. Please refer to Table 1 for additional crystal and refinement information.

Structure solution and refinement

The space group $P2_1/c$ was determined based on intensity statistics and systematic absences. The structure was solved and refined using the SHELX suite of programs.[3,4] An intrinsic-methods solution was calculated, which provided most non-hydrogen atoms from the E-map. Full-matrix least squares / difference Fourier cycles were performed, which located all non-hydrogen atoms. All non-hydrogen atoms were refined with anisotropic displacement parameters. The hydrogen atoms were placed in ideal positions and refined as riding atoms with relative isotropic displacement parameters. The final anisotropic full-matrix least-squares refinement on F^2 with 338 variables converged at R1 = 5.09%, for the observed data and wR2 = 14.81% for all data. The goodness-of-fit was 1.086. The largest peak in the final difference electron density synthesis was 0.192 $e^-/Å^3$ and the largest hole was -0.261 $e^-/Å^3$ with an RMS deviation of 0.055 $e^-/Å^3$. On the basis of the final model, the calculated density was 1.212 g/cm^3 and F(000), 1048 e^-.

1 SAINT V8.40A (2020), Bruker AXS, Madison, WI.
2 L. Krause, R. Herbst-Irmer, G. M. Sheldrick, D. Stalke:
Comparison of silver and molybdenum microfocus X-ray sources for single-crystal structure determination.
J. Appl. Cryst., 48, 3-10 (2015). doi:10.1107/S1600576714022985.
3 G. M. Sheldrick:
SHELXT--Integrated space-group and crystal-structure determination.
Acta Cryst. A71, 3-8 (2015). doi:10.1107/S2053273314026370.
4 G. M. Sheldrick:
Crystal structure refinement with SHELXL.
Acta Cryst. C71, 3-8 (2015). doi:10.1107/S2053229614024218.

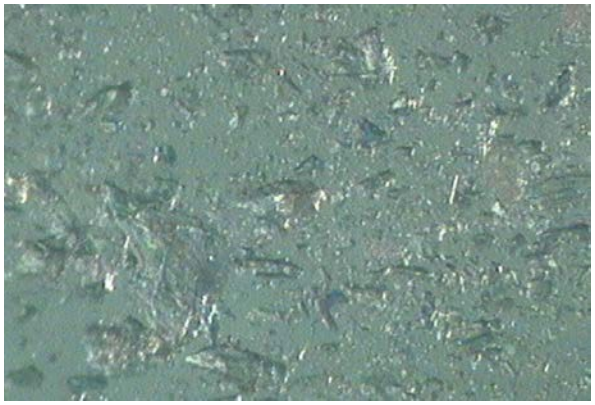

Bulk material.

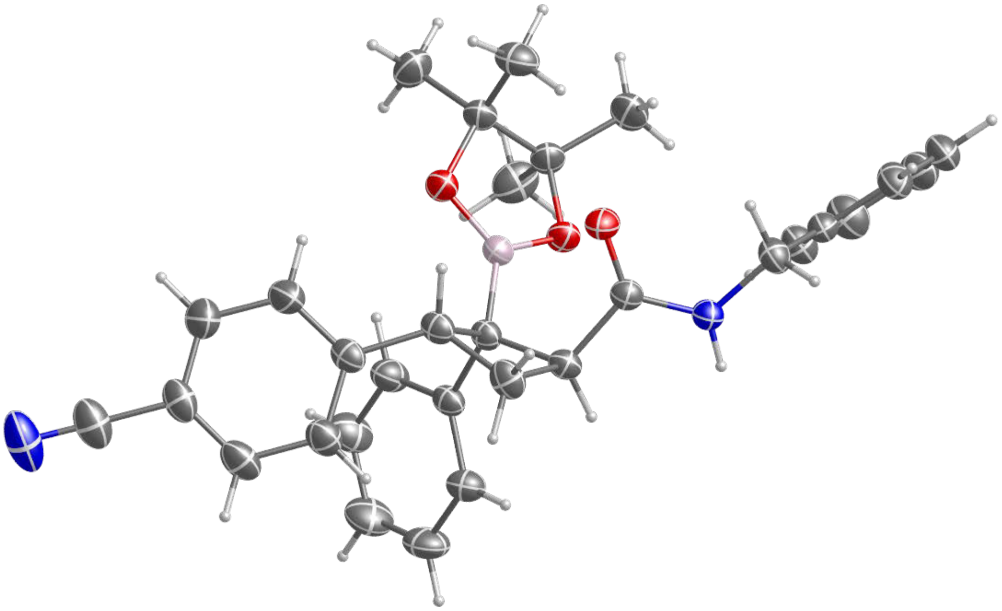

Formula unit.

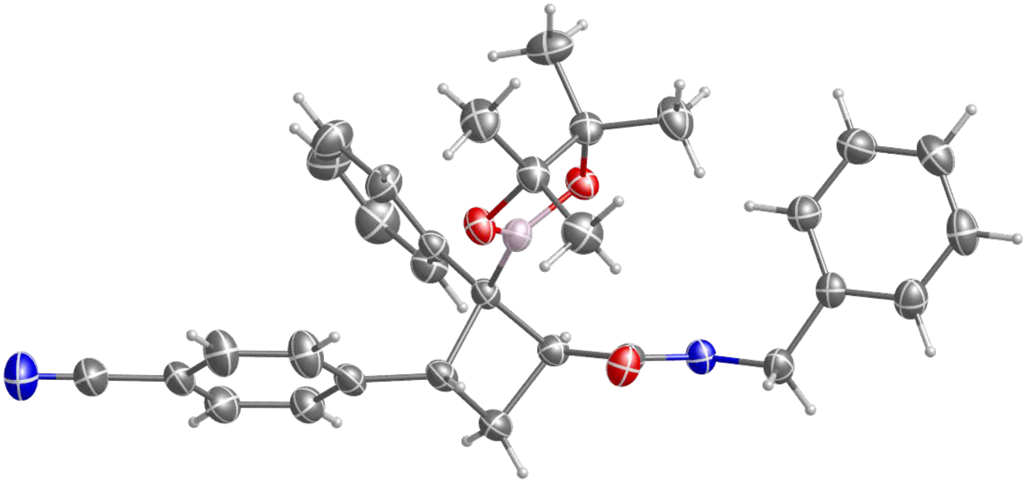

Formula unit.

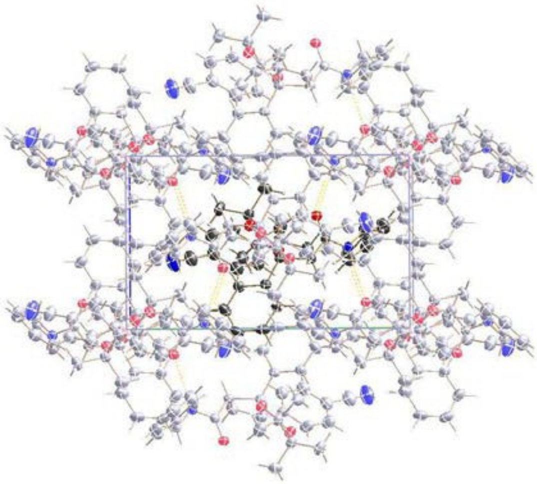

Cell plot, view along *a*.

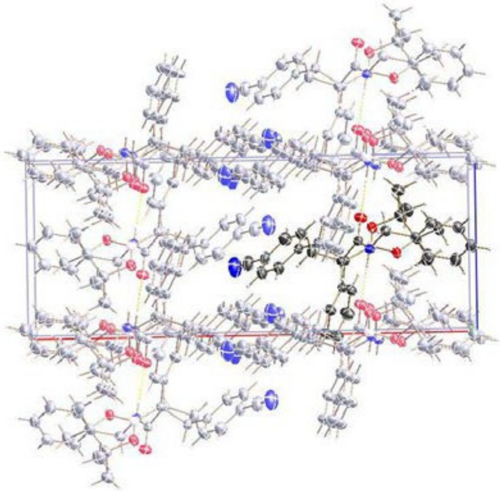

Cell plot, view along *b*.

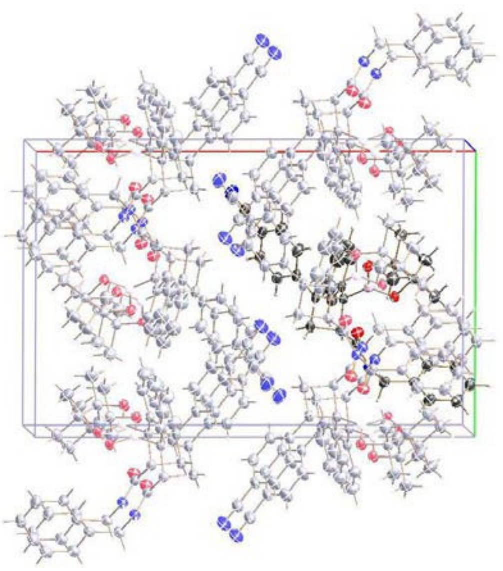

Cell plot, view along *c*.

Table 1. Crystal data and structure refinement for 24066.

Empirical formula	C31 H33 B N2 O3	
Formula weight	492.40	
Crystal color, shape, size	colorless plate, 0.127 × 0.107 × 0.049 mm^3	
Temperature	173(2) K	
Wavelength	0.71073 Å	
Crystal system, space group	Monoclinic, $P2_1/c$	
Unit cell dimensions	a = 21.8877(18) Å	α = 90°.
	b = 14.1739(11) Å	β = 90.179(3)°.
	c = 8.6957(6) Å	γ = 90°.
Volume	2697.7(4) $Å^3$	
Z	4	
Density (calculated)	1.212 Mg/m^3	
Absorption coefficient	0.077 mm^{-1}	
F(000)	1048	

Data collection

Diffractometer	Venture D8, Bruker
Source	Iμ3.0, Incoatec
Detector	Photon III
Theta range for data collection	2.351 to 25.093°.
Index ranges	-26<=h<=26, -16<=k<=16, -10<=l<=10
Reflections collected	89925
Independent reflections	4791 [R_{int} = 0.1099]
Observed Reflections	3733
Completeness to theta = 25.093°	99.8 %

Solution and Refinement

Absorption correction	Semi-empirical from equivalents
Max. and min. transmission	0.7452 and 0.5584
Solution	Intrinsic methods
Refinement method	Full-matrix least-squares on F^2
Weighting scheme	$w = [\sigma^2 Fo^2 + AP^2 + BP]^{-1}$, with<br>$P = (Fo^2 + 2 Fc^2)/3$, A = 0.0768, B = 1.0352
Data / restraints / parameters	4791 / 0 / 338
Goodness-of-fit on F^2	1.086
Final R indices [I>2σ(I)]	R1 = 0.0509, wR2 = 0.1310
R indices (all data)	R1 = 0.0683, wR2 = 0.1481
Largest diff. peak and hole	0.192 and -0.261 $e.Å^{-3}$

Goodness-of-fit = $[\Sigma[w(F_o^2 - F_c^2)^2]/(N_{observns} - N_{params})]^{1/2}$, all data.

$R1 = \Sigma(|F_o| - |F_c|) / \Sigma |F_o|$. $wR2 = [\Sigma[w(F_o^2 - F_c^2)^2] / \Sigma [w(F_o^2)^2]]^{1/2}$.

Table 2. Atomic coordinates ($\times 10^4$) and equivalent isotropic displacement parameters ($Å^2 \times 10^3$) for 24066. U_{eq} is defined as one third of the trace of the orthogonalized U^{ij} tensor.

	x	y	z	U(eq)
O1	2496(1)	6628(1)	6485(2)	36(1)
O2	1716(1)	5276(1)	4472(1)	30(1)
O3	2278(1)	4274(1)	5976(2)	33(1)
N1	2287(1)	7732(1)	4674(2)	29(1)
N2	5445(1)	1596(2)	3889(3)	72(1)
C1	2957(1)	6436(1)	4014(2)	28(1)
C2	2884(1)	5329(1)	4069(2)	26(1)
C3	3489(1)	5308(1)	5102(2)	30(1)
C4	3621(1)	6330(1)	4616(2)	36(1)
C5	2557(1)	6938(1)	5163(2)	27(1)
C6	1907(1)	8311(1)	5660(2)	33(1)
C7	1232(1)	8299(1)	5285(2)	29(1)
C8	864(1)	8947(2)	6039(2)	39(1)
C9	241(1)	8980(2)	5758(2)	44(1)
C10	-20(1)	8371(2)	4706(3)	44(1)
C11	341(1)	7730(2)	3958(3)	48(1)
C12	967(1)	7687(2)	4256(2)	39(1)
C13	1267(1)	4689(1)	5271(2)	32(1)
C14	1656(1)	4229(1)	6585(2)	32(1)
C15	760(1)	5325(2)	5817(3)	46(1)
C16	1031(1)	3982(2)	4102(3)	48(1)
C17	1660(1)	4800(2)	8058(2)	45(1)
C18	1504(1)	3211(2)	6934(3)	47(1)
C19	2964(1)	4828(1)	2540(2)	28(1)
C20	2794(1)	3884(2)	2426(2)	38(1)
C21	2882(1)	3374(2)	1088(3)	49(1)
C22	3142(1)	3800(2)	-175(3)	52(1)
C23	3322(1)	4729(2)	-83(2)	49(1)
C24	3234(1)	5242(2)	1260(2)	36(1)
C25	3943(1)	4533(1)	4825(2)	31(1)
C26	3885(1)	3706(2)	5660(3)	45(1)
C27	4272(1)	2946(2)	5424(3)	47(1)
C28	4728(1)	3016(2)	4332(2)	39(1)
C29	4794(1)	3837(2)	3489(3)	43(1)
C30	4402(1)	4588(2)	3734(2)	38(1)
C31	5129(1)	2222(2)	4076(3)	51(1)
B1	2287(1)	4969(2)	4880(2)	27(1)

Table 3. Bond lengths [Å] and angles [°] for 24066.

O1-C5	1.239(2)	O2-B1	1.368(2)
O2-C13	1.464(2)	O3-B1	1.371(2)
O3-C14	1.465(2)	N1-C5	1.340(2)
N1-C6	1.451(2)	N1-H1	0.8800
N2-C31	1.137(3)	C1-C5	1.508(2)
C1-C4	1.551(3)	C1-C2	1.578(2)
C1-H1A	1.0000	C2-C19	1.518(2)
C2-B1	1.571(3)	C2-C3	1.598(2)
C3-C25	1.502(3)	C3-C4	1.537(3)
C_3-H_3	1.0000	C4-H4A	0.9900
C4-H4B	0.9900	C6-C7	1.510(3)
C6-H6A	0.9900	C6-H6B	0.9900
C7-C12	1.375(3)	C7-C8	1.388(3)
C8-C9	1.386(3)	C8-H8	0.9500
C9-C10	1.379(3)	C9-H9	0.9500
C10-C11	1.370(3)	C10-H10	0.9500
C11-C12	1.394(3)	C11-H11	0.9500
C12-H12	0.9500	C13-C15	1.508(3)
C13-C16	1.517(3)	C13-C14	1.566(3)
C14-C18	1.512(3)	C14-C17	1.515(3)
C15-H15A	0.9800	C15-H15B	0.9800
C15-H15C	0.9800	C16-H16A	0.9800
C16-H16B	0.9800	C16-H16C	0.9800
C17-H17A	0.9800	C17-H17B	0.9800
C17-H17C	0.9800	C18-H18A	0.9800
C18-H18B	0.9800	C18-H18C	0.9800
C19-C24	1.392(3)	C19-C20	1.393(3)
C20-C21	1.383(3)	C20-H20	0.9500
C21-C22	1.378(3)	C21-H21	0.9500
C22-C23	1.377(4)	C22-H22	0.9500
C23-C24	1.389(3)	C23-H23	0.9500
C24-H24	0.9500	C25-C26	1.385(3)
C25-C30	1.387(3)	C26-C27	1.385(3)
C26-H26	0.9500	C27-C28	1.384(3)
C27-H27	0.9500	C28-C29	1.383(3)
C28-C31	1.445(3)	C29-C30	1.385(3)
C29-H29	0.9500	C30-H30	0.9500

B1-O2-C13	108.15(14)	B1-O3-C14	107.30(14)
C5-N1-C6	122.78(15)	C5-N1-H1	118.6
C6-N1-H1	118.6	C5-C1-C4	111.61(15)
C5-C1-C2	112.92(15)	C4-C1-C2	89.33(14)
C5-C1-H1A	113.6	C4-C1-H1A	113.6
C2-C1-H1A	113.6	C19-C2-B1	109.91(15)

C19-C2-C1	115.27(15)	B1-C2-C1	114.89(15)
C19-C2-C3	112.64(14)	B1-C2-C3	115.45(15)
C1-C2-C3	87.23(13)	C25-C3-C4	121.29(16)
C25-C3-C2	118.16(15)	C4-C3-C2	89.09(14)
C25-C3-H3	108.9	C4-C3-H3	108.9
C2-C3-H3	108.9	C3-C4-C1	90.40(14)
C3-C4-H4A	113.6	C1-C4-H4A	113.6
C3-C4-H4B	113.6	C1-C4-H4B	113.6
H4A-C4-H4B	110.8	O1-C5-N1	122.92(17)
O1-C5-C1	120.80(16)	N1-C5-C1	116.27(15)
N1-C6-C7	115.37(16)	N1-C6-H6A	108.4
C7-C6-H6A	108.4	N1-C6-H6B	108.4
C7-C6-H6B	108.4	H6A-C6-H6B	107.5
C12-C7-C8	118.66(19)	C12-C7-C6	123.98(17)
C8-C7-C6	117.36(17)	C9-C8-C7	120.8(2)
C9-C8-H8	119.6	C7-C8-H8	119.6
C10-C9-C8	120.1(2)	C10-C9-H9	120.0
C8-C9-H9	120.0	C11-C10-C9	119.4(2)
C11-C10-H10	120.3	C9-C10-H10	120.3
C10-C11-C12	120.5(2)	C10-C11-H11	119.7
C12-C11-H11	119.7	C7-C12-C11	120.47(19)
C7-C12-H12	119.8	C11-C12-H12	119.8
O2-C13-C15	107.86(15)	O2-C13-C16	106.57(16)
C15-C13-C16	110.89(18)	O2-C13-C14	102.59(14)
C15-C13-C14	114.71(17)	C16-C13-C14	113.41(16)
O3-C14-C18	108.56(16)	O3-C14-C17	106.21(16)
C18-C14-C17	109.95(17)	O3-C14-C13	102.83(14)
C18-C14-C13	115.16(17)	C17-C14-C13	113.41(17)
C13-C15-H15A	109.5	C13-C15-H15B	109.5
H15A-C15-H15B	109.5	C13-C15-H15C	109.5
H15A-C15-H15C	109.5	H15B-C15-H15C	109.5
C13-C16-H16A	109.5	C13-C16-H16B	109.5
H16A-C16-H16B	109.5	C13-C16-H16C	109.5
H16A-C16-H16C	109.5	H16B-C16-H16C	109.5
C14-C17-H17A	109.5	C14-C17-H17B	109.5
H17A-C17-H17B	109.5	C14-C17-H17C	109.5
H17A-C17-H17C	109.5	H17B-C17-H17C	109.5
C14-C18-H18A	109.5	C14-C18-H18B	109.5
H18A-C18-H18B	109.5	C14-C18-H18C	109.5
H18A-C18-H18C	109.5	H18B-C18-H18C	109.5
C24-C19-C20	117.55(17)	C24-C19-C2	123.63(17)
C20-C19-C2	118.70(16)	C21-C20-C19	121.61(19)
C21-C20-H20	119.2	C19-C20-H20	119.2
C22-C21-C20	120.1(2)	C22-C21-H21	120.0

C20-C21-H21	120.0	C23-C22-C21	119.4(2)
C23-C22-H22	120.3	C21-C22-H22	120.3
C22-C23-C24	120.6(2)	C22-C23-H23	119.7
C24-C23-H23	119.7	C23-C24-C19	120.7(2)
C23-C24-H24	119.6	C19-C24-H24	119.6
C26-C25-C30	118.31(19)	C26-C25-C3	118.25(18)
C30-C25-C3	123.38(18)	C27-C26-C25	121.5(2)
C27-C26-H26	119.2	C25-C26-H26	119.2
C28-C27-C26	119.3(2)	C28-C27-H27	120.3
C26-C27-H27	120.3	C29-C28-C27	120.02(19)
C29-C28-C31	120.6(2)	C27-C28-C31	119.4(2)
C28-C29-C30	120.0(2)	C28-C29-H29	120.0
C30-C29-H29	120.0	C29-C30-C25	120.8(2)
C29-C30-H30	119.6	C25-C30-H30	119.6
N2-C31-C28	179.4(3)	O2-B1-O3	113.16(17)
O2-B1-C2	122.71(16)	O3-B1-C2	123.96(17)

Table 4. Anisotropic displacement parameters ($Å^2 \times 10^3$) for 24066. The anisotropic displacement factor exponent takes the form: $-2\pi^2[h^2 a^{*2}U^{11} + ... + 2 h k a^* b^* U^{12}]$

	U^{11}	U^{22}	U^{33}	U^{23}	U^{13}	U^{12}
O1	46(1)	34(1)	27(1)	1(1)	6(1)	4(1)
O2	24(1)	33(1)	34(1)	5(1)	4(1)	-2(1)
O3	28(1)	36(1)	37(1)	7(1)	6(1)	4(1)
N1	31(1)	32(1)	25(1)	1(1)	2(1)	4(1)
N2	51(1)	55(1)	110(2)	-22(1)	3(1)	16(1)
C1	25(1)	31(1)	28(1)	-1(1)	4(1)	-1(1)
C2	22(1)	30(1)	26(1)	-1(1)	0(1)	2(1)
C3	23(1)	39(1)	28(1)	-2(1)	-1(1)	3(1)
C4	25(1)	38(1)	45(1)	-6(1)	-1(1)	0(1)
C5	26(1)	29(1)	27(1)	-1(1)	1(1)	-2(1)
C6	33(1)	34(1)	32(1)	-5(1)	0(1)	6(1)
C7	32(1)	29(1)	26(1)	3(1)	3(1)	4(1)
C8	43(1)	40(1)	34(1)	-4(1)	2(1)	7(1)
C9	42(1)	48(1)	42(1)	3(1)	10(1)	15(1)
C10	31(1)	53(1)	48(1)	8(1)	3(1)	5(1)
C11	37(1)	53(1)	53(1)	-9(1)	-3(1)	-2(1)
C12	34(1)	38(1)	44(1)	-7(1)	4(1)	1(1)
C13	27(1)	30(1)	37(1)	4(1)	6(1)	-4(1)
C14	29(1)	33(1)	36(1)	4(1)	8(1)	0(1)
C15	31(1)	49(1)	58(1)	9(1)	13(1)	5(1)
C16	53(1)	44(1)	47(1)	1(1)	-6(1)	-16(1)

C17	44(1)	57(1)	34(1)	-1(1)	8(1)	0(1)
C18	46(1)	38(1)	56(1)	14(1)	9(1)	-2(1)
C19	22(1)	36(1)	26(1)	-2(1)	1(1)	3(1)
C20	42(1)	37(1)	35(1)	-4(1)	10(1)	-2(1)
C21	57(2)	44(1)	46(1)	-15(1)	10(1)	-5(1)
C22	62(2)	61(2)	33(1)	-15(1)	7(1)	-2(1)
C23	54(1)	65(2)	28(1)	1(1)	10(1)	1(1)
C24	36(1)	43(1)	31(1)	0(1)	6(1)	-1(1)
C25	24(1)	41(1)	28(1)	-4(1)	-3(1)	4(1)
C26	42(1)	53(1)	40(1)	9(1)	10(1)	13(1)
C27	46(1)	48(1)	48(1)	9(1)	3(1)	14(1)
C28	27(1)	43(1)	47(1)	-10(1)	-5(1)	7(1)
C29	29(1)	49(1)	52(1)	-9(1)	8(1)	0(1)
C30	31(1)	40(1)	43(1)	-2(1)	5(1)	1(1)
C31	35(1)	48(1)	69(2)	-14(1)	-2(1)	6(1)
B1	29(1)	27(1)	26(1)	-2(1)	4(1)	2(1)

Table 5. Hydrogen coordinates (× 10^4) and isotropic displacement parameters (Å^2 × 10^3) for 24066.

	x	y	z	U_{eq}
H1	2343	7910	3716	35
H1A	2930	6705	2953	34
H3	3369	5302	6211	36
H4A	3934	6382	3799	43
H4B	3719	6752	5489	43
H6A	2053	8971	5600	40
H6B	1962	8097	6735	40
H8	1041	9373	6757	47
H9	-7	9422	6290	53
H10	-446	8397	4502	53
H11	164	7311	3230	57
H12	1211	7232	3743	47
H15A	463	4955	6406	69
H15B	555	5612	4928	69
H15C	930	5823	6473	69
H16A	733	3563	4594	72
H16B	1372	3608	3708	72
H16C	834	4319	3250	72
H17A	1254	4780	8528	67
H17B	1769	5456	7826	67
H17C	1962	4534	8773	67

H18A	1079	3165	7278	70
H18B	1777	2978	7746	70
H18C	1559	2830	6004	70
H20	2614	3582	3288	46
H21	2762	2731	1040	59
H22	3197	3455	-1101	62
H23	3507	5023	-946	58
H24	3360	5883	1305	44
H26	3573	3658	6413	54
H27	4224	2384	6005	57
H29	5109	3885	2742	52
H30	4449	5149	3147	46

Table 6. Torsion angles [°] for 24066.

C5-C1-C2-C19	147.64(15)	C4-C1-C2-C19	-99.10(16)
C5-C1-C2-B1	18.3(2)	C4-C1-C2-B1	131.54(16)
C5-C1-C2-C3	-98.58(16)	C4-C1-C2-C3	14.68(14)
C19-C2-C3-C25	-24.0(2)	B1-C2-C3-C25	103.35(19)
C1-C2-C3-C25	-140.31(17)	C19-C2-C3-C4	101.47(16)
B1-C2-C3-C4	-131.16(16)	C1-C2-C3-C4	-14.82(14)
C25-C3-C4-C1	137.94(17)	C2-C3-C4-C1	15.07(14)
C5-C1-C4-C3	99.21(16)	C2-C1-C4-C3	-15.26(14)
C6-N1-C5-O1	1.1(3)	C6-N1-C5-C1	-177.75(16)
C4-C1-C5-O1	-57.2(2)	C2-C1-C5-O1	41.6(2)
C4-C1-C5-N1	121.67(17)	C2-C1-C5-N1	-139.48(16)
C5-N1-C6-C7	-109.7(2)	N1-C6-C7-C12	9.7(3)
N1-C6-C7-C8	-170.50(17)	C12-C7-C8-C9	-0.2(3)
C6-C7-C8-C9	180.00(19)	C7-C8-C9-C10	-0.7(3)
C8-C9-C10-C11	0.7(3)	C9-C10-C11-C12	0.2(3)
C8-C7-C12-C11	1.1(3)	C6-C7-C12-C11	-179.1(2)
C10-C11-C12-C7	-1.1(3)	B1-O2-C13-C15	139.22(17)
B1-O2-C13-C16	-101.66(18)	B1-O2-C13-C14	17.76(18)
B1-O3-C14-C18	144.27(17)	B1-O3-C14-C17	-97.55(18)
B1-O3-C14-C13	21.83(19)	O2-C13-C14-O3	-23.69(17)
C15-C13-C14-O3	-140.35(17)	C16-C13-C14-O3	90.84(19)
O2-C13-C14-C18	-141.57(17)	C15-C13-C14-C18	101.8(2)
C16-C13-C14-C18	-27.0(2)	O2-C13-C14-C17	90.54(18)
C15-C13-C14-C17	-26.1(2)	C16-C13-C14-C17	-154.93(18)
B1-C2-C19-C24	147.85(18)	C1-C2-C19-C24	16.1(2)
C3-C2-C19-C24	-81.9(2)	B1-C2-C19-C20	-36.2(2)
C1-C2-C19-C20	-167.95(17)	C3-C2-C19-C20	94.1(2)
C24-C19-C20-C21	-0.8(3)	C2-C19-C20-C21	-177.05(19)

C19-C20-C21-C22	0.0(4)	C20-C21-C22-C23	0.9(4)
C21-C22-C23-C24	-0.9(4)	C22-C23-C24-C19	0.1(3)
C20-C19-C24-C23	0.8(3)	C2-C19-C24-C23	176.81(19)
C4-C3-C25-C26	161.49(19)	C2-C3-C25-C26	-90.8(2)
C4-C3-C25-C30	-21.2(3)	C2-C3-C25-C30	86.5(2)
C30-C25-C26-C27	-0.1(3)	C3-C25-C26-C27	177.4(2)
C25-C26-C27-C28	0.3(4)	C26-C27-C28-C29	-0.1(3)
C26-C27-C28-C31	-179.6(2)	C27-C28-C29-C30	-0.2(3)
C31-C28-C29-C30	179.3(2)	C28-C29-C30-C25	0.3(3)
C26-C25-C30-C29	-0.2(3)	C3-C25-C30-C29	-177.52(19)
C13-O2-B1-O3	-4.7(2)	C13-O2-B1-C2	170.76(16)
C14-O3-B1-O2	-11.8(2)	C14-O3-B1-C2	172.77(17)
C19-C2-B1-O2	-78.8(2)	C1-C2-B1-O2	53.2(2)
C3-C2-B1-O2	152.48(17)	C19-C2-B1-O3	96.2(2)
C1-C2-B1-O3	-131.88(18)	C3-C2-B1-O3	-32.6(2)

Table 7. Hydrogen bonds for 24066 [Å and °].

D-H...A	d(D-H)	d(H...A)	d(D...A)	<(DHA)
N1-H1...O1#1	0.88	2.08	2.955(2)	177.8
C17-H17B...O1	0.98	2.58	3.455(3)	148.5

Symmetry transformations used to generate equivalent atoms: #1 x,-y+3/2,z-1/2

INDIANA UNIVERSITY DEPARTMENT OF CHEMISTRY

Molecular Structure Center

Report No. 26272
(NS-1-889)

$C_{30}H_{34}BNO_3$

Prepared for
Neetu Sharma and Professor Kevin Brown

by M. Pink, May 13, 2026

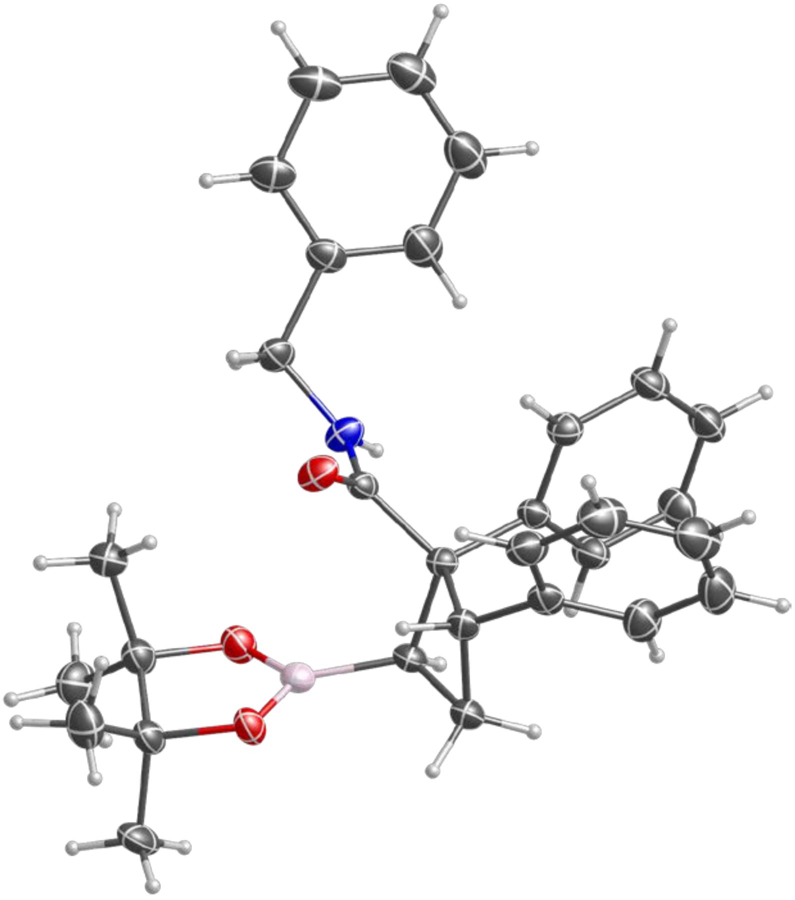

Molecular Structure Center

A colorless, plate-shaped specimen of $C_{30}H_{34}BNO_3$, IUMSC 26272, approximate dimensions 0.04×0.119×0.234 mm^3, was placed on a Kapton mount with inert oil for crystal structure determination. The X-ray intensity data were measured on a Bruker D8 Venture KAPPA diffractometer equipped with a microfocus sealed tube (λ = 1.54178 Å) and a multilayer mirror monochromator. The experiment was performed at 153(2) K using an Oxford Cryostream open-nitrogen-flow temperature control unit.

Data collection

The data collection was performed using 1° ω and φ scans, frame times of 20 and 1 s, and a detector distance of 40 mm. Overall, 2608 frames were collected with a total exposure time of 6.58 hours. The frames were integrated with the SAINT V8.41 package using a narrow-frame algorithm.[1] The integration of the data using a monoclinic unit cell yielded 15916 reflections to a maximum θ angle of 68.41° (0.83 Å resolution), of which 4124 were independent (average redundancy 3.86, completeness = 99.7%, R_{int} = 9.52%, R_{sig} = 7.70%) and 3757 (91.1%) were greater than $2\sigma(F^2)$. The final cell constants of a = 6.4173(7) Å, b = 12.0768(12) Å, c = 16.6136(17) Å, α = 90°, β = 98.849(6)°, γ = 90°, volume = 1272.2(2) $Å^3$, are based upon the refinement of the XYZ-centroids of 8611 reflections above 20 $\sigma(I)$ with $3.66° < 2\theta < 68.21°$. Data were corrected for absorption effects using the Multi-Scan method in SADABS 2016/2. The calculated minimum and maximum transmission coefficients (based on crystal size) are 0.871 and 0.976.[2] Additional crystal and refinement information can be found in the tables.

Structure solution and refinement

The space group *Pn* (7) was determined based on intensity statistics and systematic absences. The structure was solved by SHELXT 2018/2 and refined with full-matrix least squares / difference Fourier cycles using SHELXL-2019/2; Z = 2 for the formula unit $C_{30}H_{34}BNO_3$.[3, 4] Non-hydrogen atoms were refined with anisotropic displacement parameters. The hydrogen atoms were placed in ideal positions and refined as riding atoms with relative isotropic displacement parameters. The final anisotropic full-matrix least-squares refinement on F^2 with 315 variables against 4124 data points and 339 restraints converged at R_1 = 4.55%, for the observed data and wR_2 = 11.74% for all data. The goodness-of-fit on F^2 was 1.07. The largest peak in the final difference electron density synthesis was 0.30 $e^-/Å^3$ and the deepest hole was −0.32 $e^-/Å^3$ with an RMS deviation of 0.092 $e^-/Å^3$. On the basis of the final model, the calculated density was 1.22 g/cm^3 and $F(000)$, 500 e^-. Disorder was refined for a phenyl moiety using restraints and constraints.

1 SAINT V8.41 (2024), Bruker AXS, Madison, WI, USA.
2 L. Krause, R. Herbst-Irmer, G. M. Sheldrick, D. Stalke: Comparison of Ag and Mo microfocus X-ray sources for single-crystal structure determination. *J. Appl. Cryst.,* 48, 3-10 (2015). doi:10.1107/S1600576714022985.
3 G. M. Sheldrick: SHELXT--Integrated space-group and crystal-structure determination. *Acta Cryst*. A71, 3-8 (2015). doi:10.1107/S2053273314026370.
4 G. M. Sheldrick: Crystal structure refinement with SHELXL. *Acta Cryst*. C71, 3-8 (2015). doi:10.1107/S2053229614024218.

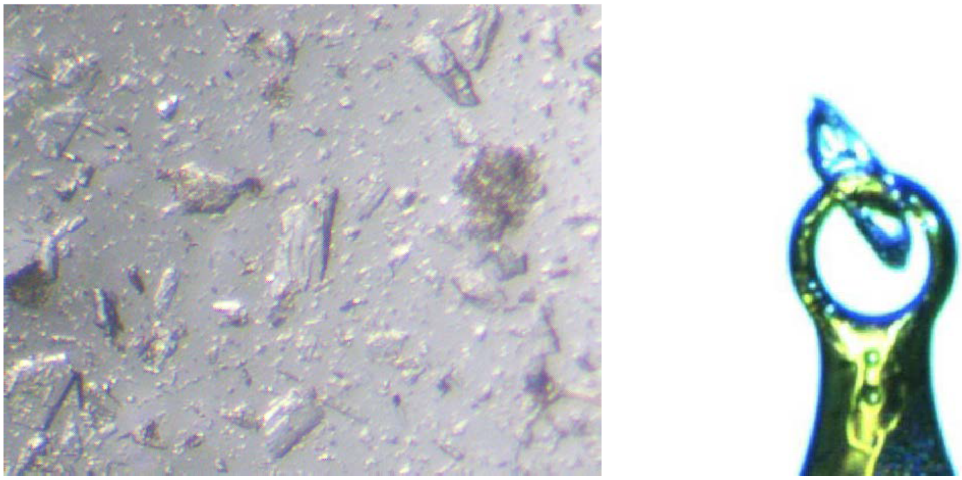

Bulk material and mounted crystal.

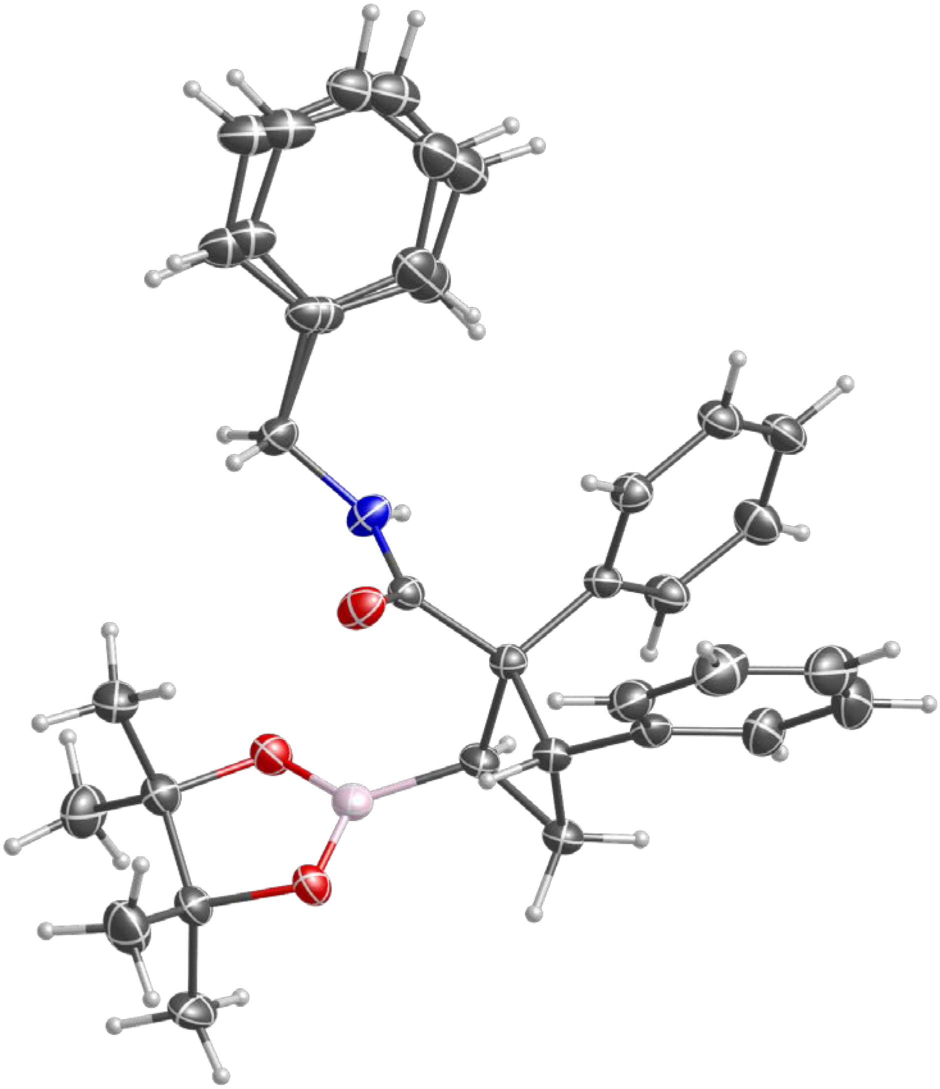

Asymmetric unit, disorder shown.

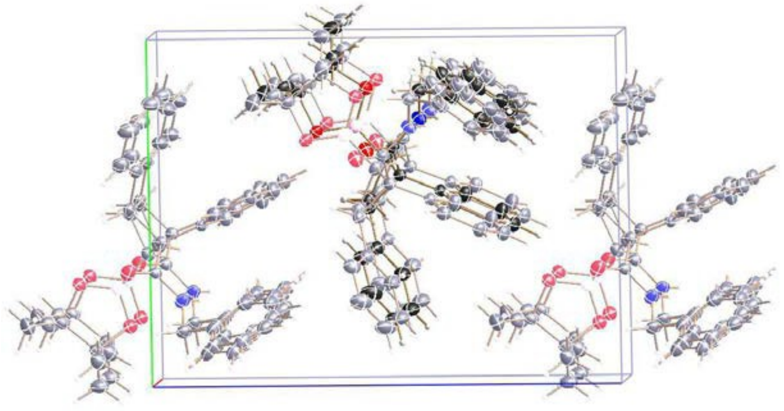

Cell plot, view along *a*.

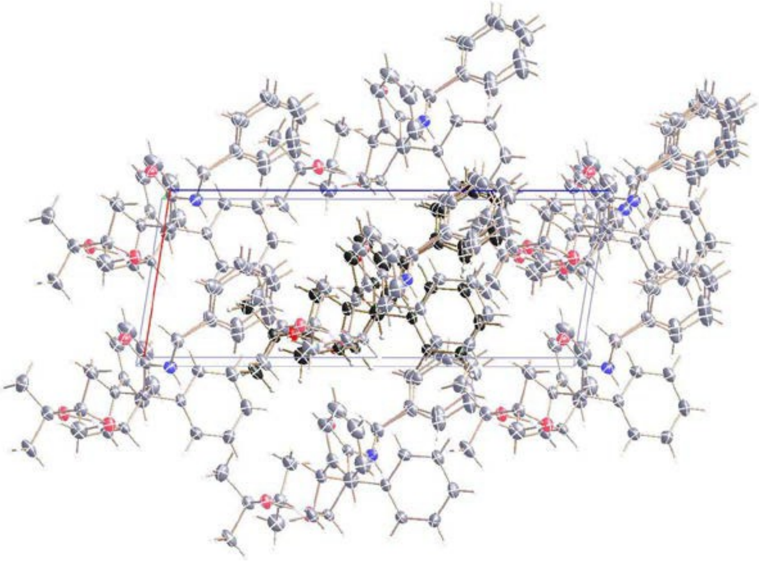

Cell plot, view along *b*.

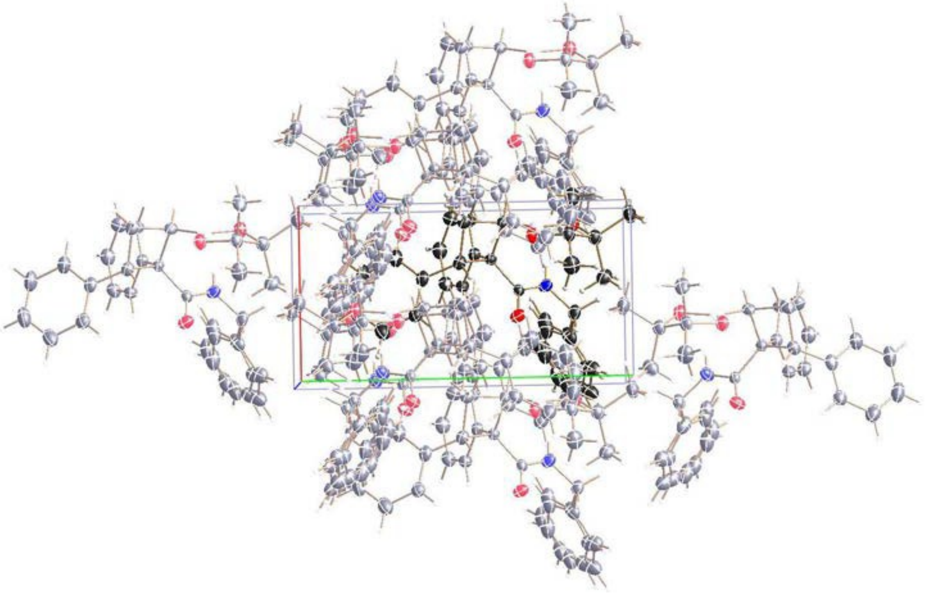

Cell plot, view along *c*.

Table 1. Crystal data and structure refinement for 26272.

Empirical formula	C30 H34 B N O3	
Formula weight	467.39	
Crystal color, shape, size	colorless plate, 0.234 × 0.119 × 0.040 mm^3	
Temperature	153(2) K	
Wavelength	1.54178 Å	
Crystal system, space group	Monoclinic, Pn	
Unit cell dimensions	a = 6.4173(7) Å	α = 90°.
	b = 12.0768(12) Å	β = 98.849(6)°.
	c = 16.6136(17) Å	γ = 90°.
Volume	1272.2(2) $Å^3$	
Z	2	
Density (calculated)	1.220 Mg/m^3	
Absorption coefficient	0.605 mm^{-1}	
F(000)	500	
Data collection		
Diffractometer	Venture D8, Bruker	
Source, detector	Incoatec Iμ3.0, Photon III	
Theta range for data collection	3.660 to 68.407°.	
Index ranges	-7<=h<=7, -14<=k<=14, -19<=l<=20	
Reflections collected	15916	
Independent reflections	4124 [R_{int} = 0.0952]	
Observed Reflections	3757	
Completeness to theta = 67.679°	99.7 %	
Solution and Refinement		
Absorption correction	Semi-empirical from equivalents	
Max. and min. transmission	0.7531 and 0.4793	
Solution	Intrinsic methods	
Refinement method	Full-matrix least-squares on F^2	
Weighting scheme	$w = [\sigma^2 Fo^2 + AP^2 + BP]^{-1}$, with	
	$P = (Fo^2 + 2 Fc^2)/3$, A = 0.0742, B = 0.0390	
Data / restraints / parameters	4124 / 339 / 315	
Goodness-of-fit on F^2	1.070	
Final R indices [I>2σ(I)]	R1 = 0.0455, wR2 = 0.1118	
R indices (all data)	R1 = 0.0564, wR2 = 0.1174	
Absolute structure parameter	0.1(2)	
Largest diff. peak and hole	0.297 and -0.317 $e.Å^{-3}$	

Goodness-of-fit = $[\Sigma[w(F_o^2 - F_c^2)^2]/N_{observns} - N_{params})]^{1/2}$, all data.

$R1 = \Sigma(|F_o| - |F_c|) / \Sigma |F_o|$. $wR2 = [\Sigma[w(F_o^2 - F_c^2)^2] / \Sigma [w(F_o^2)^2]]^{1/2}$.

Table 2. Atomic coordinates (× 10^4) and equivalent isotropic displacement parameters ($Å^2$ × 10^3) for 26272. U_{eq} is defined as one third of the trace of the orthogonalized U^{ij} tensor.

	x	y	z	U(eq)
O1	8232(3)	7104(2)	3414(1)	27(1)
O2	8919(3)	8335(2)	4466(1)	29(1)
O3	3701(3)	6665(2)	4557(1)	30(1)
N1	5453(4)	7531(2)	5663(2)	28(1)
C1	9063(4)	6267(2)	4866(2)	24(1)
C2	7016(4)	5876(2)	5200(2)	21(1)
C3	6815(4)	4930(2)	4540(2)	24(1)
C4	9175(5)	5097(2)	4489(2)	29(1)
C5	8258(5)	8194(2)	3029(2)	27(1)
C6	8073(5)	9004(2)	3756(2)	28(1)
C7	6457(6)	8245(3)	2328(2)	40(1)
C8	10367(6)	8298(3)	2720(2)	38(1)
C9	5813(5)	9267(3)	3848(2)	36(1)
C10	9359(6)	10057(3)	3769(2)	39(1)
C11	5197(4)	6716(2)	5099(2)	20(1)
C12	3886(5)	8384(2)	5720(2)	28(1)
C13	2660(20)	8197(15)	6434(8)	28(1)
C14	3430(20)	7510(13)	7083(8)	37(1)
C15	2280(30)	7360(11)	7719(7)	44(2)
C16	360(20)	7896(13)	7705(7)	42(3)
C17	-410(15)	8583(14)	7056(8)	38(2)
C18	739(18)	8733(14)	6420(8)	34(1)
C13D	2520(50)	8270(30)	6378(18)	28(1)
C14D	3030(40)	7570(30)	7046(19)	37(1)
C15D	1720(40)	7510(20)	7638(13)	44(2)
C16D	-100(40)	8153(19)	7562(13)	42(3)
C17D	-610(30)	8849(19)	6895(15)	38(2)
C18D	700(50)	8910(30)	6303(15)	34(1)
C19	7337(4)	5407(2)	6061(2)	22(1)
C20	9288(4)	5261(2)	6534(2)	28(1)
C21	9467(5)	4758(3)	7296(2)	32(1)
C22	7676(5)	4410(2)	7601(2)	32(1)
C23	5726(5)	4560(2)	7139(2)	30(1)
C24	5552(4)	5044(2)	6374(2)	26(1)
C25	5955(5)	3813(2)	4725(2)	27(1)
C26	7158(6)	3028(3)	5211(2)	35(1)
C27	6256(7)	2030(3)	5402(2)	43(1)
C28	4170(7)	1807(3)	5122(3)	47(1)
C29	2972(6)	2571(3)	4636(3)	45(1)
C30	3871(5)	3569(3)	4435(2)	34(1)
B1	8745(5)	7246(3)	4233(2)	24(1)

Table 3. Bond lengths [Å] and angles [°] for 26272.

O1-B1	1.361(4)	O1-C5	1.464(3)
O2-B1	1.371(4)	O2-C6	1.464(3)
O3-C11	1.213(4)	N1-C11	1.352(3)
N1-C12	1.453(3)	N1-H1N	0.8800
C1-C4	1.552(4)	C1-C2	1.575(3)
C1-B1	1.575(4)	C1-H1	1.0000
C2-C19	1.522(3)	C2-C11	1.536(3)
C2-C3	1.576(3)	C3-C25	1.507(4)
C3-C4	1.543(4)	C3-H3	1.0000
C4-H4A	0.9900	C4-H4B	0.9900
C5-C7	1.510(5)	C5-C8	1.524(4)
C5-C6	1.572(4)	C6-C10	1.514(4)
C6-C9	1.515(4)	C7-H7A	0.9800
C7-H7B	0.9800	C7-H7C	0.9800
C8-H8A	0.9800	C8-H8B	0.9800
C8-H8C	0.9800	C9-H9A	0.9800
C9-H9B	0.9800	C9-H9C	0.9800
C10-H10A	0.9800	C10-H10B	0.9800
C10-H10C	0.9800	C12-C13D	1.510(14)
C12-C13	1.536(6)	C12-H12A	0.9900
C12-H12B	0.9900	C13-C14	1.3900
C13-C18	1.3900	C14-C15	1.3900
C14-H14	0.9500	C15-C16	1.3900
C15-H15	0.9500	C16-C17	1.3900
C16-H16	0.9500	C17-C18	1.3900
C17-H17	0.9500	C18-H18	0.9500
C13D-C14D	1.3900	C13D-C18D	1.3900
C14D-C15D	1.3900	C14D-H14D	0.9500
C15D-C16D	1.3900	C15D-H15D	0.9500
C16D-C17D	1.3900	C16D-H16D	0.9500
C17D-C18D	1.3900	C17D-H17D	0.9500
C18D-H18D	0.9500	C19-C20	1.384(4)
C19-C24	1.400(4)	C20-C21	1.393(4)
C20-H20	0.9500	C21-C22	1.390(4)
C21-H21	0.9500	C22-C23	1.376(5)
C22-H22	0.9500	C23-C24	1.388(4)
C23-H23	0.9500	C24-H24	0.9500
C25-C30	1.381(4)	C25-C26	1.399(4)
C26-C27	1.395(5)	C26-H26	0.9500
C27-C28	1.374(6)	C27-H27	0.9500
C28-C29	1.381(6)	C28-H28	0.9500
C29-C30	1.398(4)	C29-H29	0.9500

C30-H30	0.9500		
B1-O1-C5	107.8(2)	B1-O2-C6	107.3(2)
C11-N1-C12	123.4(2)	C11-N1-H1N	118.3
C12-N1-H1N	118.3	C4-C1-C2	87.86(19)
C4-C1-B1	115.0(2)	C2-C1-B1	115.5(2)
C4-C1-H1	112.1	C2-C1-H1	112.1
B1-C1-H1	112.1	C19-C2-C11	109.8(2)
C19-C2-C1	116.3(2)	C11-C2-C1	115.1(2)
C19-C2-C3	111.7(2)	C11-C2-C3	115.0(2)
C1-C2-C3	87.44(19)	C25-C3-C4	121.5(2)
C25-C3-C2	120.0(2)	C4-C3-C2	88.1(2)
C25-C3-H3	108.5	C4-C3-H3	108.5
C2-C3-H3	108.5	C3-C4-C1	89.43(19)
C3-C4-H4A	113.7	C1-C4-H4A	113.7
C3-C4-H4B	113.7	C1-C4-H4B	113.7
H4A-C4-H4B	111.0	O1-C5-C7	108.3(2)
O1-C5-C8	107.0(2)	C7-C5-C8	110.5(3)
O1-C5-C6	102.5(2)	C7-C5-C6	115.3(3)
C8-C5-C6	112.5(2)	O2-C6-C10	108.5(2)
O2-C6-C9	106.0(2)	C10-C6-C9	110.5(3)
O2-C6-C5	102.3(2)	C10-C6-C5	115.4(2)
C9-C6-C5	113.2(2)	C5-C7-H7A	109.5
C5-C7-H7B	109.5	H7A-C7-H7B	109.5
C5-C7-H7C	109.5	H7A-C7-H7C	109.5
H7B-C7-H7C	109.5	C5-C8-H8A	109.5
C5-C8-H8B	109.5	H8A-C8-H8B	109.5
C5-C8-H8C	109.5	H8A-C8-H8C	109.5
H8B-C8-H8C	109.5	C6-C9-H9A	109.5
C6-C9-H9B	109.5	H9A-C9-H9B	109.5
C6-C9-H9C	109.5	H9A-C9-H9C	109.5
H9B-C9-H9C	109.5	C6-C10-H10A	109.5
C6-C10-H10B	109.5	H10A-C10-H10B	109.5
C6-C10-H10C	109.5	H10A-C10-H10C	109.5
H10B-C10-H10C	109.5	O3-C11-N1	123.4(2)
O3-C11-C2	123.3(2)	N1-C11-C2	113.3(2)
N1-C12-C13D	117.8(12)	N1-C12-C13	112.8(6)
N1-C12-H12A	109.0	C13-C12-H12A	109.0
N1-C12-H12B	109.0	C13-C12-H12B	109.0
H12A-C12-H12B	107.8	C14-C13-C18	120.0
C14-C13-C12	121.5(7)	C18-C13-C12	118.5(7)
C13-C14-C15	120.0	C13-C14-H14	120.0
C15-C14-H14	120.0	C16-C15-C14	120.0
C16-C15-H15	120.0	C14-C15-H15	120.0

C15-C16-C17	120.0	C15-C16-H16	120.0
C17-C16-H16	120.0	C18-C17-C16	120.0
C18-C17-H17	120.0	C16-C17-H17	120.0
C17-C18-C13	120.0	C17-C18-H18	120.0
C13-C18-H18	120.0	C14D-C13D-C18D	120.0
C14D-C13D-C12	122.8(17)	C18D-C13D-C12	117.2(17)
C15D-C14D-C13D	120.0	C15D-C14D-H14D	120.0
C13D-C14D-H14D	120.0	C14D-C15D-C16D	120.0
C14D-C15D-H15D	120.0	C16D-C15D-H15D	120.0
C15D-C16D-C17D	120.0	C15D-C16D-H16D	120.0
C17D-C16D-H16D	120.0	C18D-C17D-C16D	120.0
C18D-C17D-H17D	120.0	C16D-C17D-H17D	120.0
C17D-C18D-C13D	120.0	C17D-C18D-H18D	120.0
C13D-C18D-H18D	120.0	C20-C19-C24	118.0(2)
C20-C19-C2	124.1(2)	C24-C19-C2	117.8(2)
C19-C20-C21	120.9(2)	C19-C20-H20	119.6
C21-C20-H20	119.6	C22-C21-C20	120.4(3)
C22-C21-H21	119.8	C20-C21-H21	119.8
C23-C22-C21	119.3(3)	C23-C22-H22	120.4
C21-C22-H22	120.4	C22-C23-C24	120.3(3)
C22-C23-H23	119.9	C24-C23-H23	119.9
C23-C24-C19	121.2(3)	C23-C24-H24	119.4
C19-C24-H24	119.4	C30-C25-C26	118.3(3)
C30-C25-C3	119.1(3)	C26-C25-C3	122.5(3)
C27-C26-C25	120.4(3)	C27-C26-H26	119.8
C25-C26-H26	119.8	C28-C27-C26	120.6(3)
C28-C27-H27	119.7	C26-C27-H27	119.7
C27-C28-C29	119.5(3)	C27-C28-H28	120.2
C29-C28-H28	120.2	C28-C29-C30	120.2(4)
C28-C29-H29	119.9	C30-C29-H29	119.9
C25-C30-C29	120.9(3)	C25-C30-H30	119.5
C29-C30-H30	119.5	O1-B1-O2	113.6(2)
O1-B1-C1	124.1(3)	O2-B1-C1	122.3(3)

Table 4. Anisotropic displacement parameters ($Å^2 \times 10^3$) for 26272. The anisotropic displacement factor exponent takes the form: $-2\pi^2[h^2 a^{*2}U^{11} + ... + 2 h k a^* b^* U^{12}]$

	U^{11}	U^{22}	U^{33}	U^{23}	U^{13}	U^{12}
O1	35(1)	22(1)	25(1)	4(1)	9(1)	-1(1)
O2	36(1)	26(1)	25(1)	1(1)	8(1)	0(1)
O3	26(1)	35(1)	30(1)	-5(1)	2(1)	6(1)
N1	27(1)	28(1)	29(1)	-5(1)	3(1)	5(1)

C1	23(1)	26(1)	25(1)	4(1)	11(1)	1(1)
C2	20(1)	21(1)	22(1)	0(1)	6(1)	1(1)
C3	30(1)	24(1)	20(1)	0(1)	8(1)	4(1)
C4	33(2)	29(1)	27(1)	5(1)	14(1)	5(1)
C5	33(2)	22(1)	28(1)	5(1)	12(1)	0(1)
C6	33(2)	24(1)	28(1)	3(1)	9(1)	0(1)
C7	49(2)	40(2)	30(1)	6(1)	2(1)	1(1)
C8	47(2)	32(2)	42(2)	5(1)	29(2)	-1(1)
C9	38(2)	36(2)	37(2)	6(1)	17(1)	7(1)
C10	48(2)	23(1)	49(2)	0(1)	14(2)	-3(1)
C11	22(1)	18(1)	22(1)	1(1)	9(1)	-1(1)
C12	35(2)	22(1)	31(1)	-2(1)	12(1)	3(1)
C13	34(2)	25(3)	26(2)	-7(2)	11(2)	-4(2)
C14	55(5)	26(2)	33(2)	-2(1)	14(3)	1(3)
C15	73(7)	29(4)	34(3)	-1(2)	19(4)	-4(3)
C16	61(5)	30(5)	40(3)	-8(3)	25(4)	-13(4)
C17	38(3)	44(6)	37(4)	-13(4)	17(3)	-9(3)
C18	37(2)	37(5)	29(3)	-10(2)	10(2)	-2(2)
C13D	34(2)	25(3)	26(2)	-7(2)	11(2)	-4(2)
C14D	55(5)	26(2)	33(2)	-2(1)	14(3)	1(3)
C15D	73(7)	29(4)	34(3)	-1(2)	19(4)	-4(3)
C16D	61(5)	30(5)	40(3)	-8(3)	25(4)	-13(4)
C17D	38(3)	44(6)	37(4)	-13(4)	17(3)	-9(3)
C18D	37(2)	37(5)	29(3)	-10(2)	10(2)	-2(2)
C19	26(1)	21(1)	20(1)	-1(1)	9(1)	0(1)
C20	26(1)	33(1)	26(1)	1(1)	8(1)	-1(1)
C21	32(2)	38(2)	24(1)	3(1)	1(1)	-1(1)
C22	44(2)	31(2)	23(1)	3(1)	12(1)	-1(1)
C23	35(2)	29(1)	28(1)	2(1)	14(1)	-3(1)
C24	23(1)	26(1)	29(1)	0(1)	7(1)	0(1)
C25	38(2)	23(1)	23(1)	-3(1)	13(1)	3(1)
C26	45(2)	24(1)	36(2)	0(1)	9(1)	2(1)
C27	64(2)	24(1)	43(2)	4(1)	14(2)	5(1)
C28	63(2)	26(2)	57(2)	-2(1)	29(2)	-6(1)
C29	44(2)	33(2)	63(2)	-4(1)	24(2)	-6(1)
C30	35(2)	30(1)	39(2)	-2(1)	14(1)	3(1)
B1	22(1)	26(2)	28(1)	2(1)	9(1)	1(1)

Table 5. Hydrogen coordinates (× 10^4) and isotropic displacement parameters (Å^2 × 10^3) for 26272.

	x	y	z	U_{eq}

H1N	6630	7547	6013	34
H1	10263	6407	5315	29
H3	5968	5218	4027	29
H4A	9496	5106	3925	34
H4B	10117	4580	4834	34
H7A	6330	9000	2110	60
H7B	5142	8036	2521	60
H7C	6730	7731	1900	60
H8A	10467	9030	2474	57
H8B	10470	7724	2311	57
H8C	11519	8207	3176	57
H9A	5179	9735	3393	54
H9B	5790	9660	4363	54
H9C	5010	8577	3847	54
H10A	8918	10474	3265	59
H10B	10858	9869	3812	59
H10C	9134	10510	4237	59
H12A	2878	8402	5205	34
H12B	4596	9113	5787	34
H14	4747	7144	7093	44
H15	2811	6891	8163	53
H16	-424	7794	8139	50
H17	-1724	8949	7046	46
H18	212	9202	5976	41
H14D	4274	7135	7097	44
H15D	2071	7039	8094	53
H16D	-995	8114	7967	50
H17D	-1858	9286	6843	46
H18D	345	9382	5846	41
H20	10522	5507	6337	33
H21	10820	4652	7609	38
H22	7798	4073	8122	38
H23	4492	4332	7345	36
H24	4197	5131	6057	31
H26	8598	3175	5411	42
H27	7091	1500	5729	52
H28	3558	1131	5263	56
H29	1532	2419	4437	54
H30	3038	4086	4095	40

Table 6. Torsion angles [°] for 26272.

C4-C1-C2-C19	93.2(2)	B1-C1-C2-C19	-150.0(2)
C4-C1-C2-C11	-136.3(2)	B1-C1-C2-C11	-19.5(3)
C4-C1-C2-C3	-19.78(19)	B1-C1-C2-C3	97.0(2)
C19-C2-C3-C25	28.3(3)	C11-C2-C3-C25	-97.7(3)
C1-C2-C3-C25	145.7(3)	C19-C2-C3-C4	-97.5(2)
C11-C2-C3-C4	136.5(2)	C1-C2-C3-C4	19.90(19)
C25-C3-C4-C1	-144.7(2)	C2-C3-C4-C1	-20.19(19)
C2-C1-C4-C3	20.21(19)	B1-C1-C4-C3	-97.1(2)
B1-O1-C5-C7	-141.6(2)	B1-O1-C5-C8	99.2(3)
B1-O1-C5-C6	-19.3(3)	B1-O2-C6-C10	-144.4(2)
B1-O2-C6-C9	96.9(3)	B1-O2-C6-C5	-22.0(3)
O1-C5-C6-O2	24.7(3)	C7-C5-C6-O2	142.2(2)
C8-C5-C6-O2	-89.8(3)	O1-C5-C6-C10	142.4(3)
C7-C5-C6-C10	-100.2(3)	C8-C5-C6-C10	27.8(4)
O1-C5-C6-C9	-88.9(3)	C7-C5-C6-C9	28.5(3)
C8-C5-C6-C9	156.5(2)	C12-N1-C11-O3	5.2(4)
C12-N1-C11-C2	-176.3(2)	C19-C2-C11-O3	-126.7(3)
C1-C2-C11-O3	99.7(3)	C3-C2-C11-O3	0.2(3)
C19-C2-C11-N1	54.8(3)	C1-C2-C11-N1	-78.8(3)
C3-C2-C11-N1	-178.3(2)	C11-N1-C12-C13D	102(2)
C11-N1-C12-C13	104.4(9)	N1-C12-C13-C14	19.1(11)
N1-C12-C13-C18	-160.9(6)	C18-C13-C14-C15	0.0
C12-C13-C14-C15	180.0(15)	C13-C14-C15-C16	0.0
C14-C15-C16-C17	0.0	C15-C16-C17-C18	0.0
C16-C17-C18-C13	0.0	C14-C13-C18-C17	0.0
C12-C13-C18-C17	-180.0(15)	N1-C12-C13D-C14D	17(3)
N1-C12-C13D-C18D	-163.8(12)	C18D-C13D-C14D-C15D	0.0
C12-C13D-C14D-C15D	179(3)	C13D-C14D-C15D-C16D	0.0
C14D-C15D-C16D-C17D	0.0	C15D-C16D-C17D-C18D	0.0
C16D-C17D-C18D-C13D	0.0	C14D-C13D-C18D-C17D	0.0
C12-C13D-C18D-C17D	-179(3)	C11-C2-C19-C20	-136.2(3)
C1-C2-C19-C20	-3.2(4)	C3-C2-C19-C20	95.0(3)
C11-C2-C19-C24	47.5(3)	C1-C2-C19-C24	-179.5(2)
C3-C2-C19-C24	-81.3(3)	C24-C19-C20-C21	0.6(4)
C2-C19-C20-C21	-175.7(3)	C19-C20-C21-C22	-1.1(5)
C20-C21-C22-C23	0.5(5)	C21-C22-C23-C24	0.6(4)
C22-C23-C24-C19	-1.1(4)	C20-C19-C24-C23	0.5(4)
C2-C19-C24-C23	177.0(2)	C4-C3-C25-C30	-152.4(3)
C2-C3-C25-C30	99.6(3)	C4-C3-C25-C26	30.5(4)
C2-C3-C25-C26	-77.5(3)	C30-C25-C26-C27	-0.7(4)
C3-C25-C26-C27	176.4(3)	C25-C26-C27-C28	-0.5(5)
C26-C27-C28-C29	1.1(5)	C27-C28-C29-C30	-0.5(5)
C26-C25-C30-C29	1.3(4)	C3-C25-C30-C29	-175.9(3)

C28-C29-C30-C25	-0.7(5)	C5-O1-B1-O2	6.2(3)
C5-O1-B1-C1	-175.2(2)	C6-O2-B1-O1	11.2(3)
C6-O2-B1-C1	-167.5(2)	C4-C1-B1-O1	11.2(4)
C2-C1-B1-O1	-89.0(3)	C4-C1-B1-O2	-170.3(2)
C2-C1-B1-O2	89.5(3)		

Table 7. Hydrogen bonds for 26272 [Å and °].

D-H...A	d(D-H)	d(H...A)	d(D...A)	<(DHA)
C12-H12A^a...O2#1	0.99	2.65	3.530(4)	148.5

Symmetry transformations used to generate equivalent atoms: #1 x-1,y,z

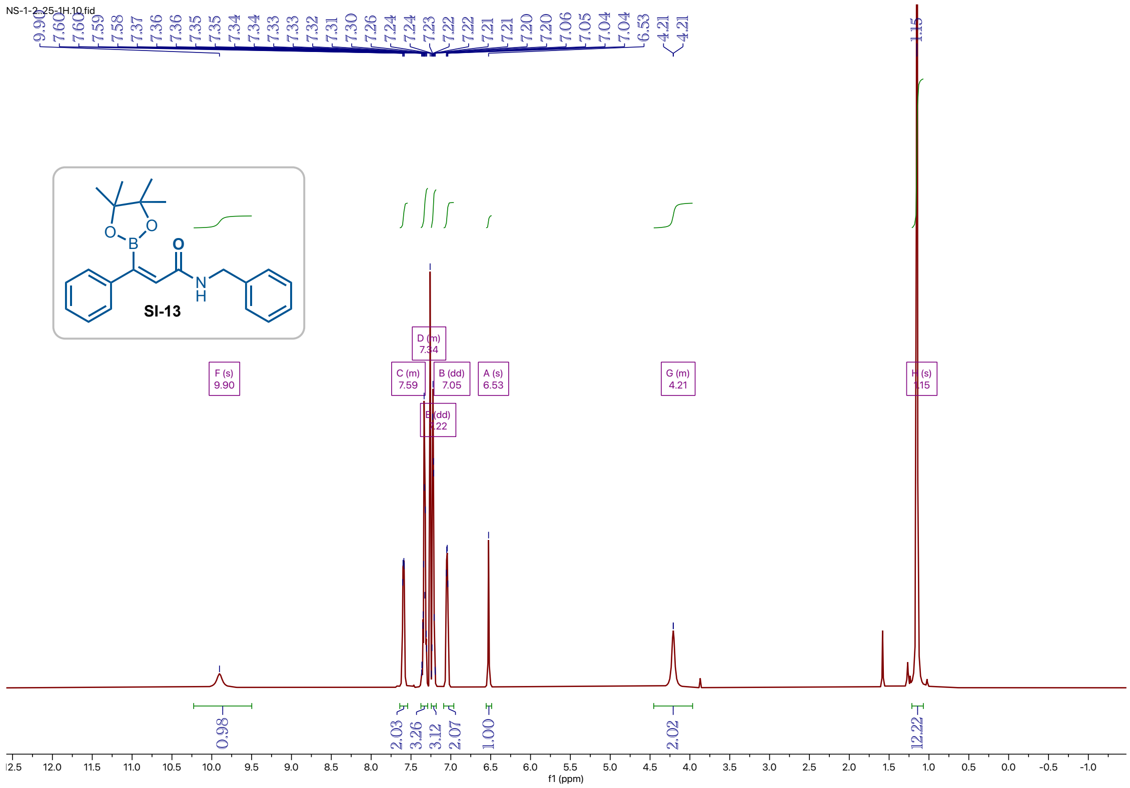

NS-1-2..25-1H.10.fid
9.90 7.60 7.60 7.59 7.58 7.37 7.36 7.36 7.35 7.35 7.34 7.34 7.33 7.33 7.32 7.31 7.30 7.26 7.24 7.24 7.23 7.22 7.22 7.21 7.21 7.20 7.20 7.06 7.05 7.04 7.04 6.53 4.21 4.21
1.15
SI-13
F (s) 9.90
C (m) 7.59
D (m) 7.34
B (dd) 7.05
E (dd) 7.22
A (s) 6.53
G (m) 4.21
H (s) 1.15
0.98 2.03 3.26 3.12 2.07 1.00 2.02 12.22
f1 (ppm)

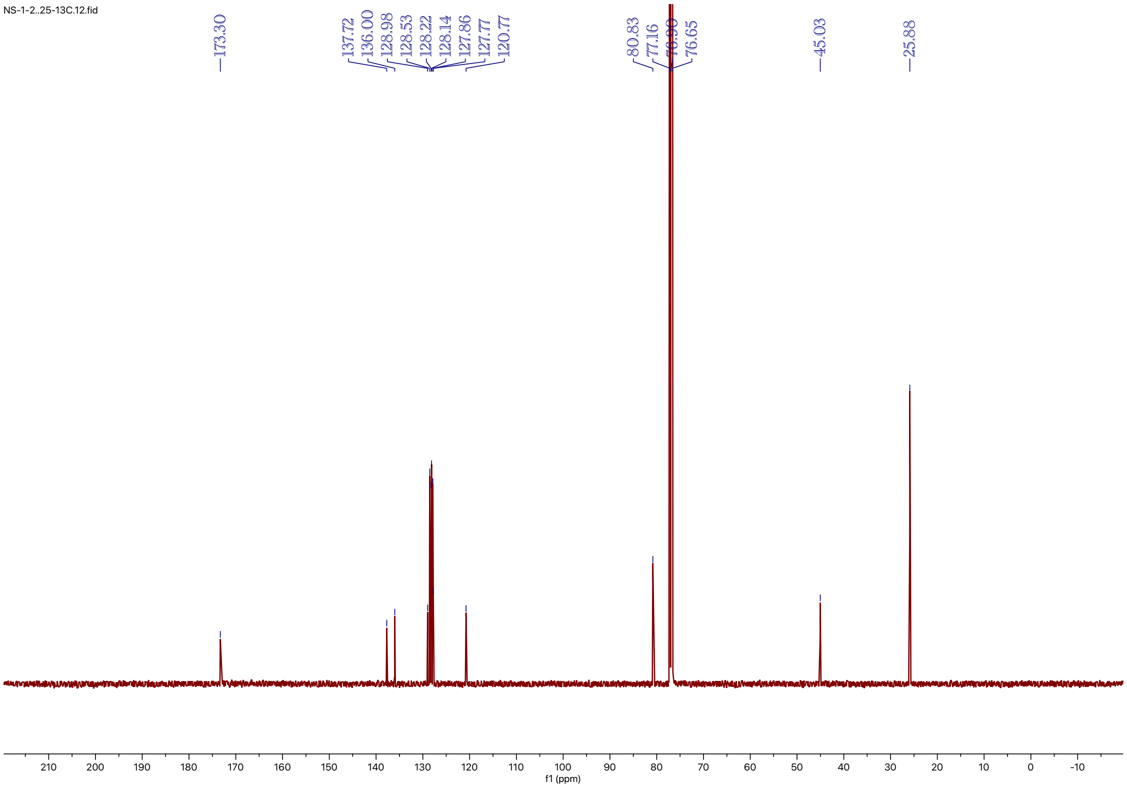

NS-1-2..25-13C.12.fid
173.30
137.72 136.00 128.98 128.53 128.22 128.14 127.86 127.77 120.77
80.83 77.16 76.90 76.65
45.03
25.88
f1 (ppm)

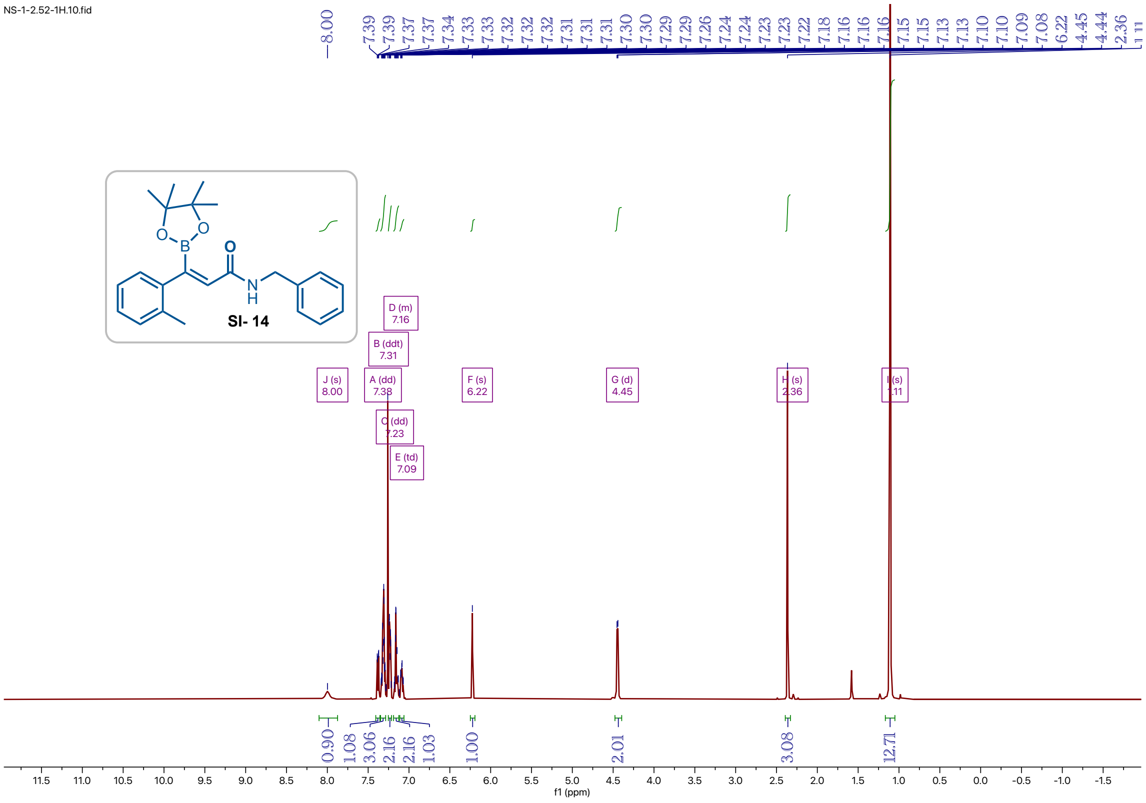
NS-1-2.52-1H.10.fid
8.00
7.39
7.39
7.37
7.37
7.34
7.33
7.33
7.32
7.32
7.31
7.31
7.31
7.30
7.30
7.29
7.29
7.26
7.24
7.24
7.23
7.23
7.22
7.18
7.16
7.16
7.16
7.15
7.15
7.13
7.13
7.10
7.10
7.09
7.08
6.22
4.45
4.44
2.36
1.11
SI- 14
J (s) 8.00
A (dd) 7.38
B (ddt) 7.31
D (m) 7.16
C (dd) 7.23
E (td) 7.09
F (s) 6.22
G (d) 4.45
H (s) 2.36
I (s) 1.11
0.90
1.08
3.06
2.16
2.16
1.03
1.00
2.01
3.08
12.71
f1 (ppm)

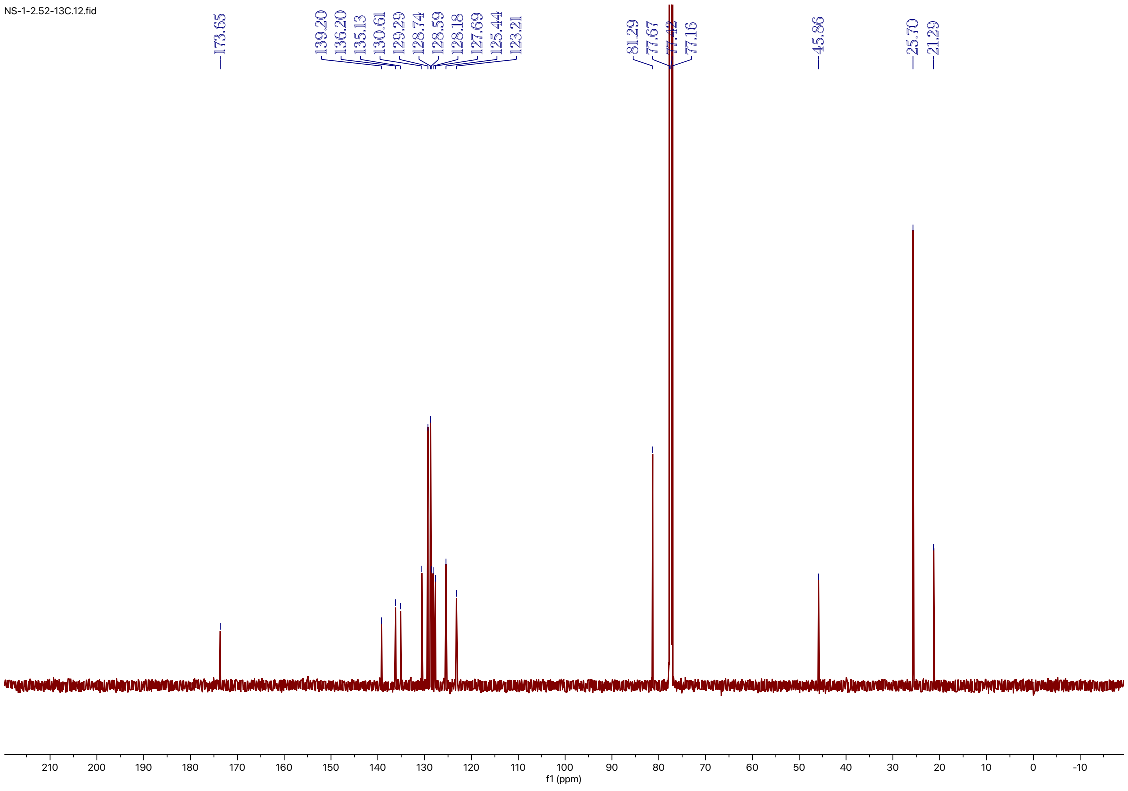
NS-1-2.52-13C.12.fid
173.65
139.20
136.20
135.13
130.61
129.29
128.74
128.59
128.18
127.69
125.44
123.21
81.29
77.67
77.16
45.86
25.70
21.29
f1 (ppm)

NS-1-2.54-1H.10.fid

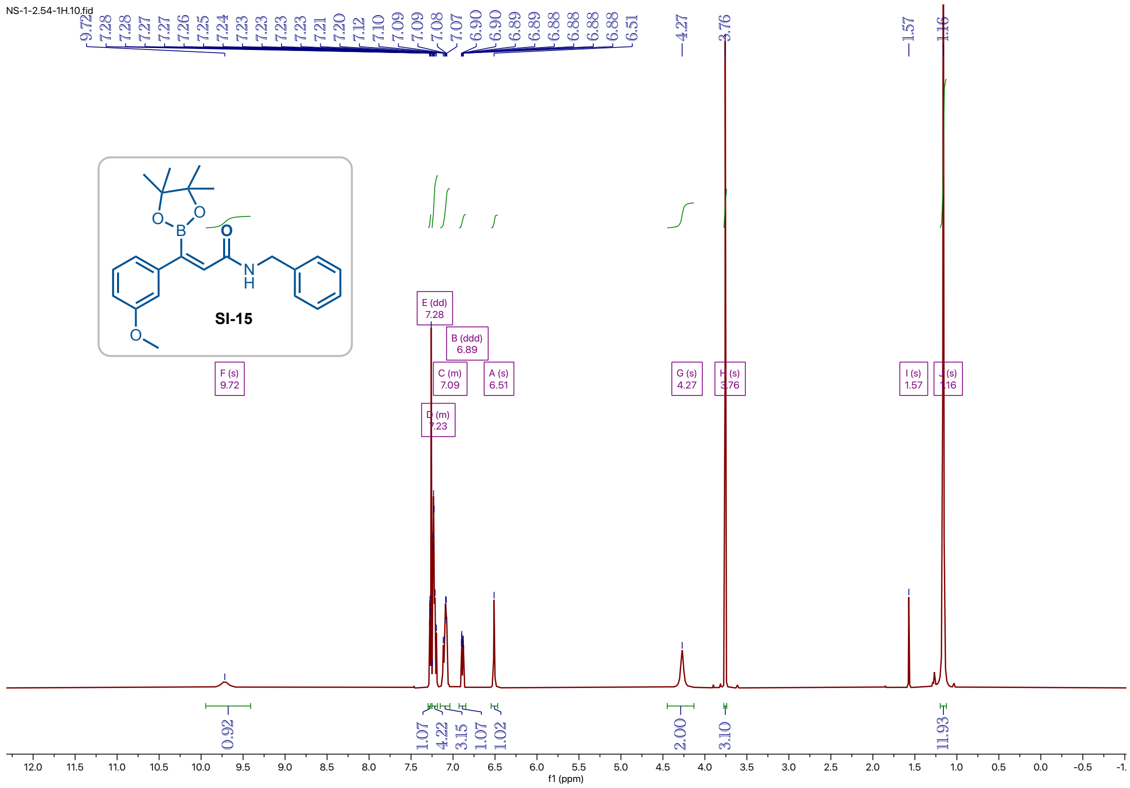

NS-1-2.54-13C.12.fid

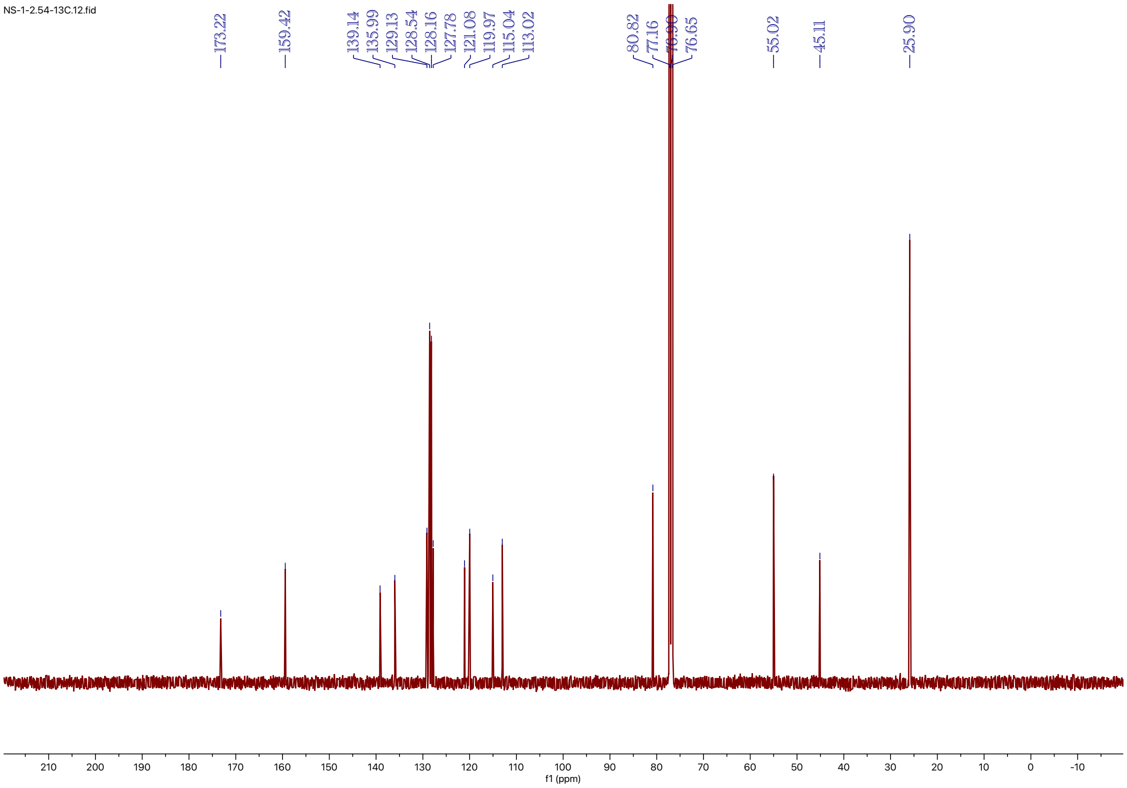

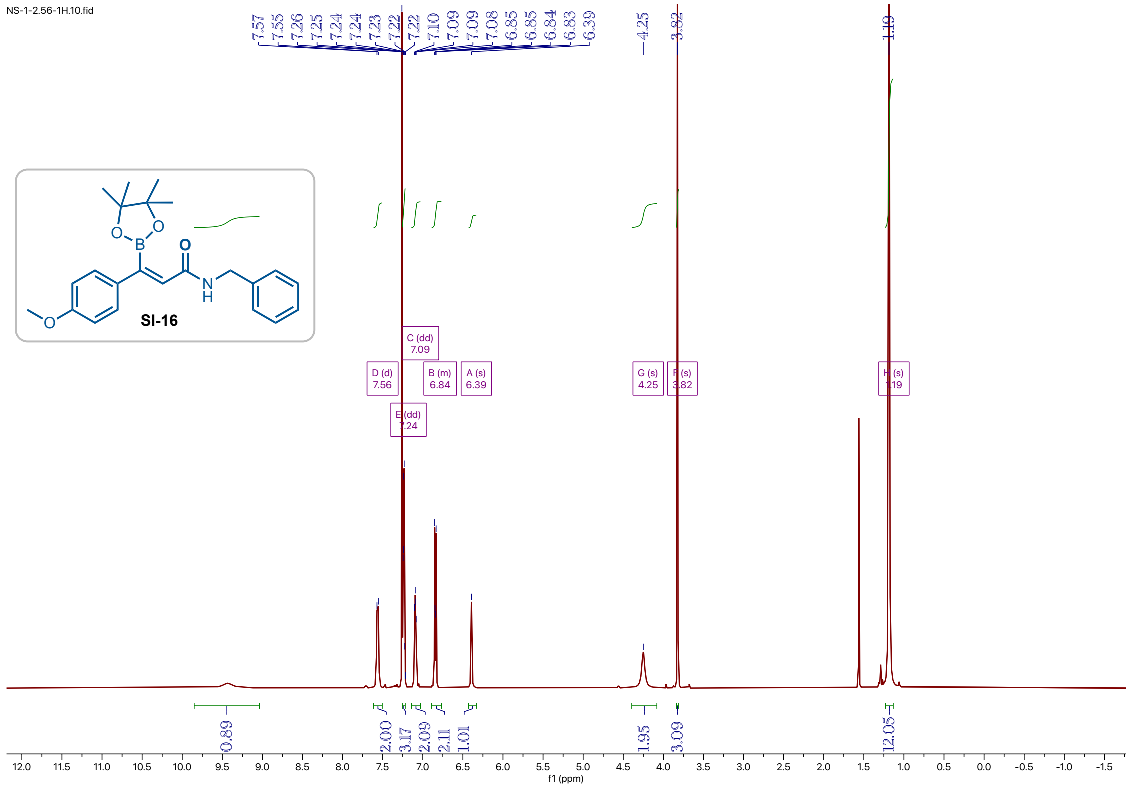
NS-1-2.56-1H.10.fid
SI-16
7.57
7.55
7.26
7.25
7.24
7.24
7.23
7.22
7.22
7.10
7.09
7.09
7.08
6.85
6.85
6.84
6.83
6.39
4.25
3.82
1.19
D (d)
7.56
C (dd)
7.09
B (m)
6.84
A (s)
6.39
E (dd)
7.24
G (s)
4.25
F (s)
3.82
H (s)
1.19
0.89
2.00
3.17
2.09
2.11
1.01
1.95
3.09
12.05
f1 (ppm)

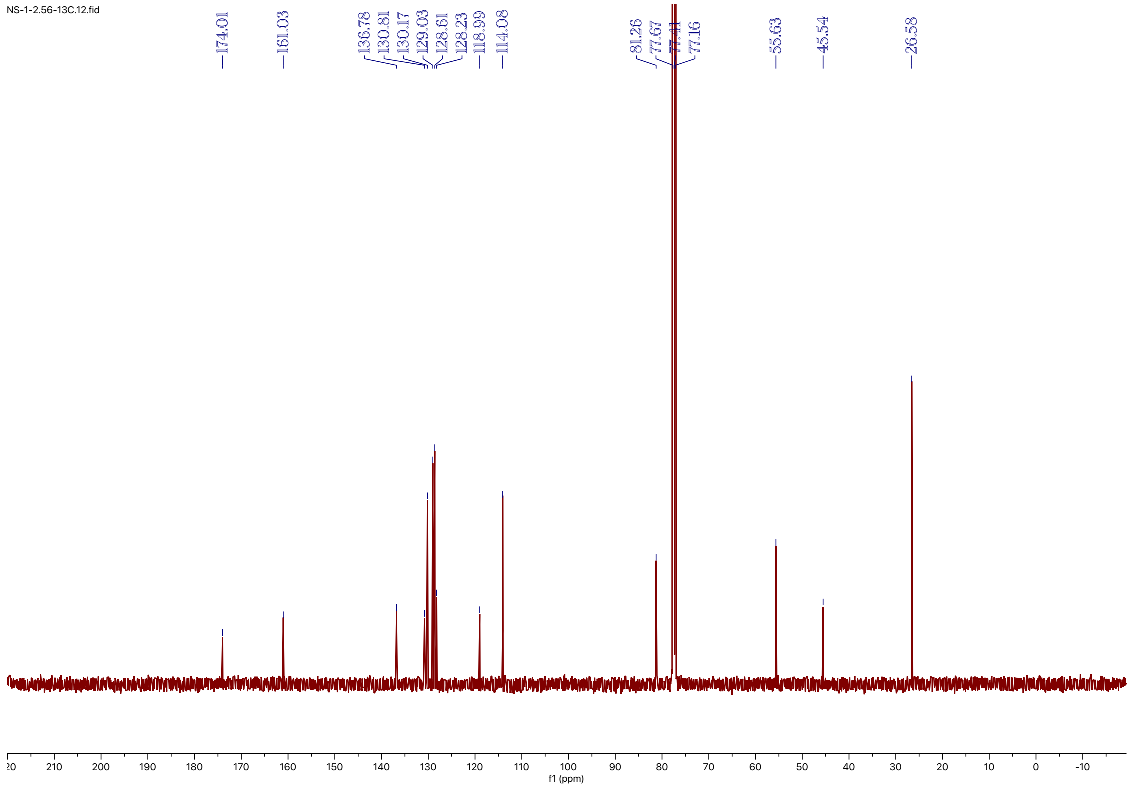
NS-1-2.56-13C.12.fid
174.01
161.03
136.78
130.81
130.17
129.03
128.61
128.23
118.99
114.08
81.26
77.67
77.41
77.16
55.63
45.54
26.58
f1 (ppm)

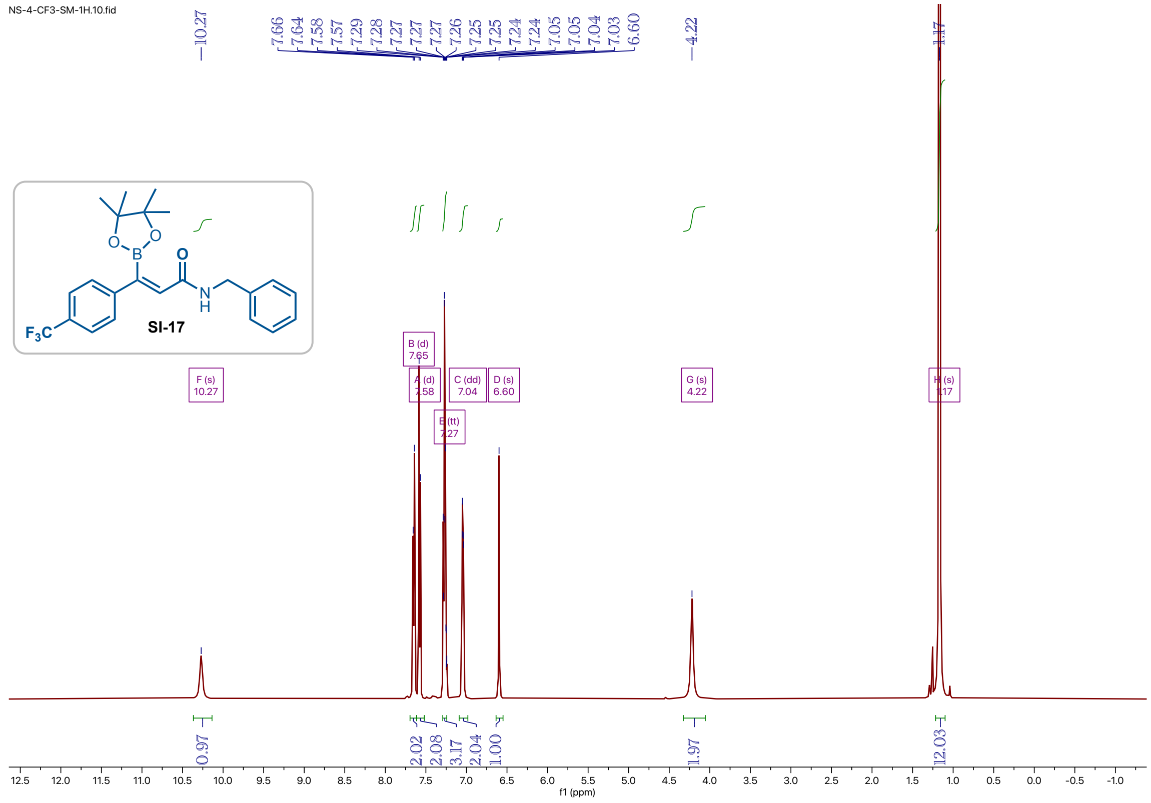
NS-4-CF3-SM-1H.10.fid
SI-17
F (s)
10.27
B (d)
7.65
A (d)
7.58
C (dd)
7.04
D (s)
6.60
E (tt)
7.27
G (s)
4.22
H (s)
1.17
f1 (ppm)

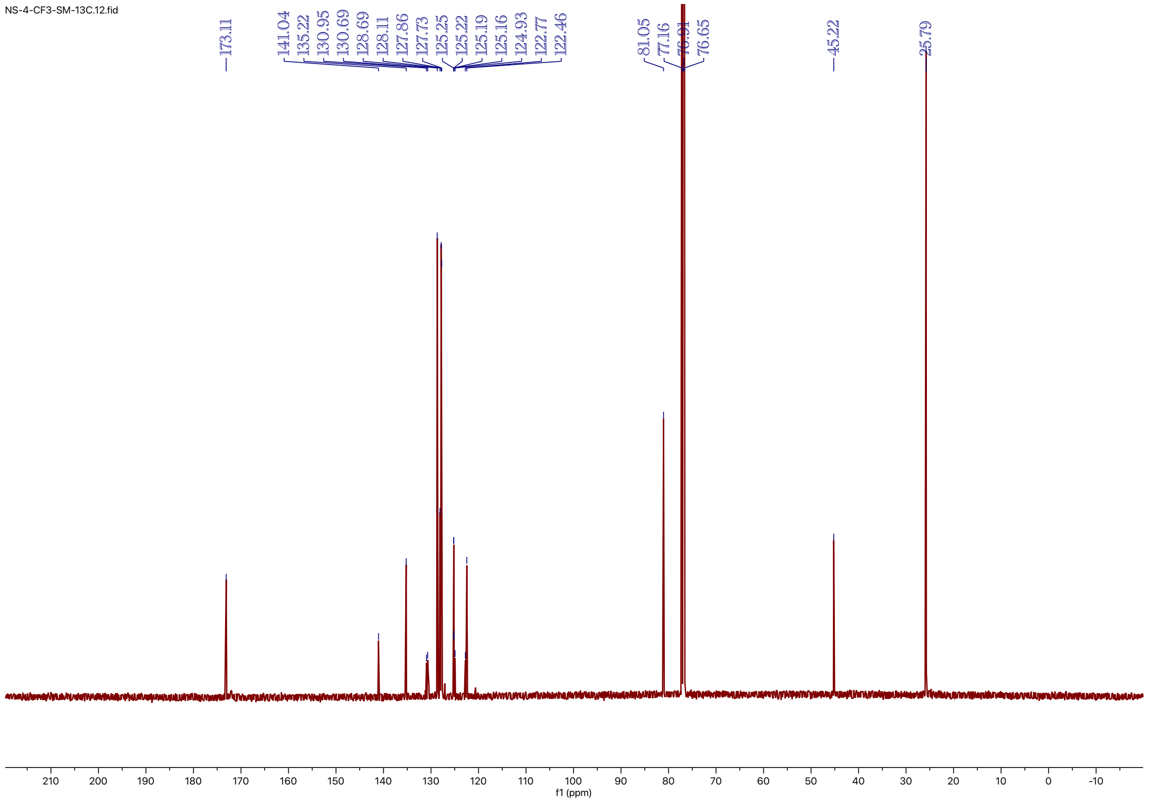
NS-4-CF3-SM-13C.12.fid
f1 (ppm)

NS-1-686-4-cf3-pu-19F.14.fid

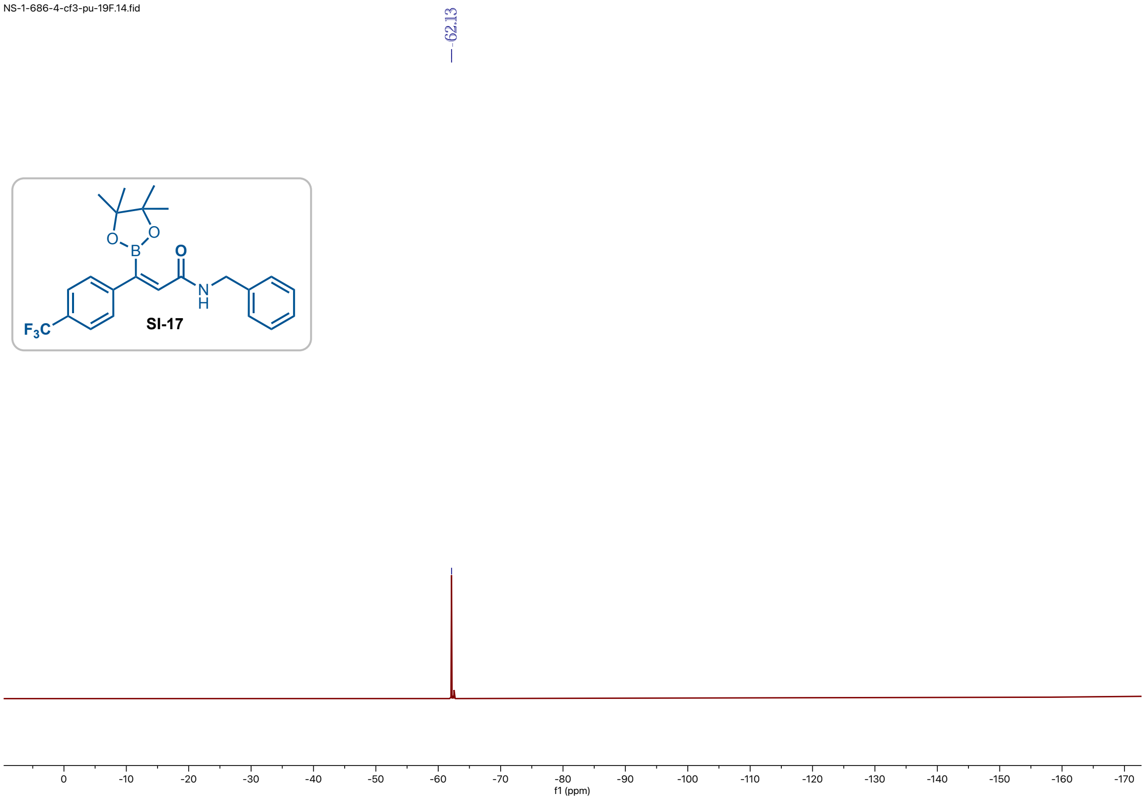

NS-1-4-Br-1H.10.fid

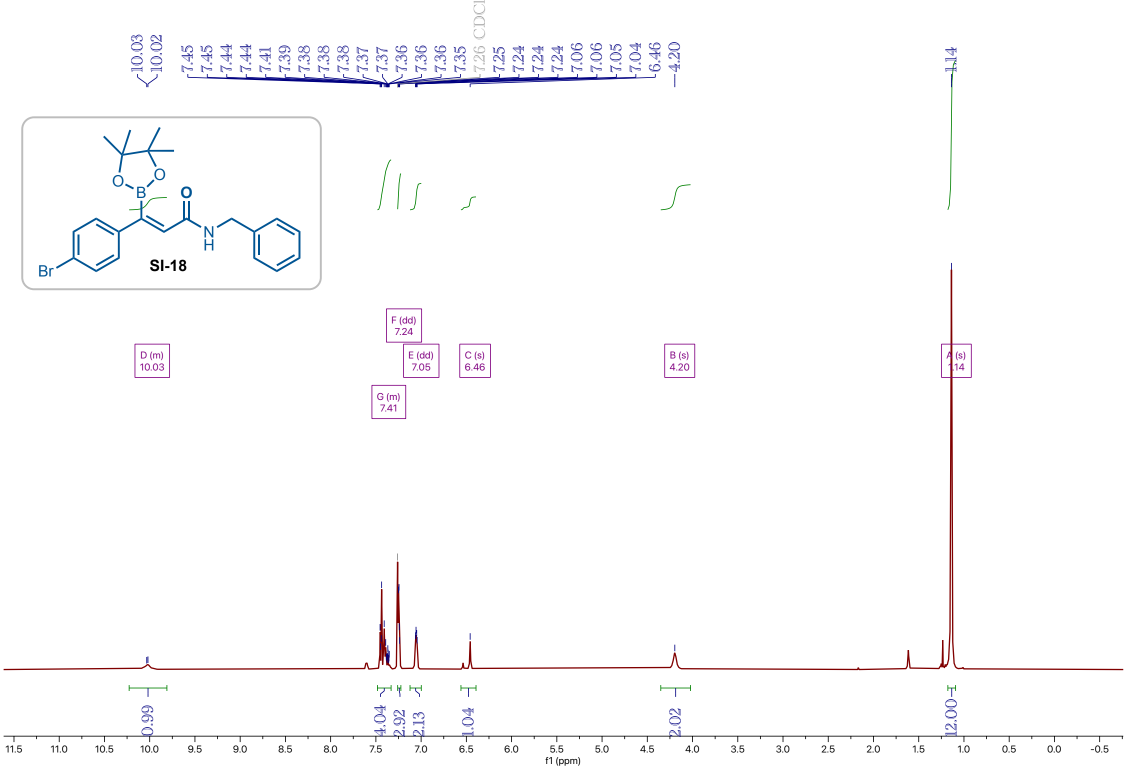

NS-1-4-Br-13C.12.fid

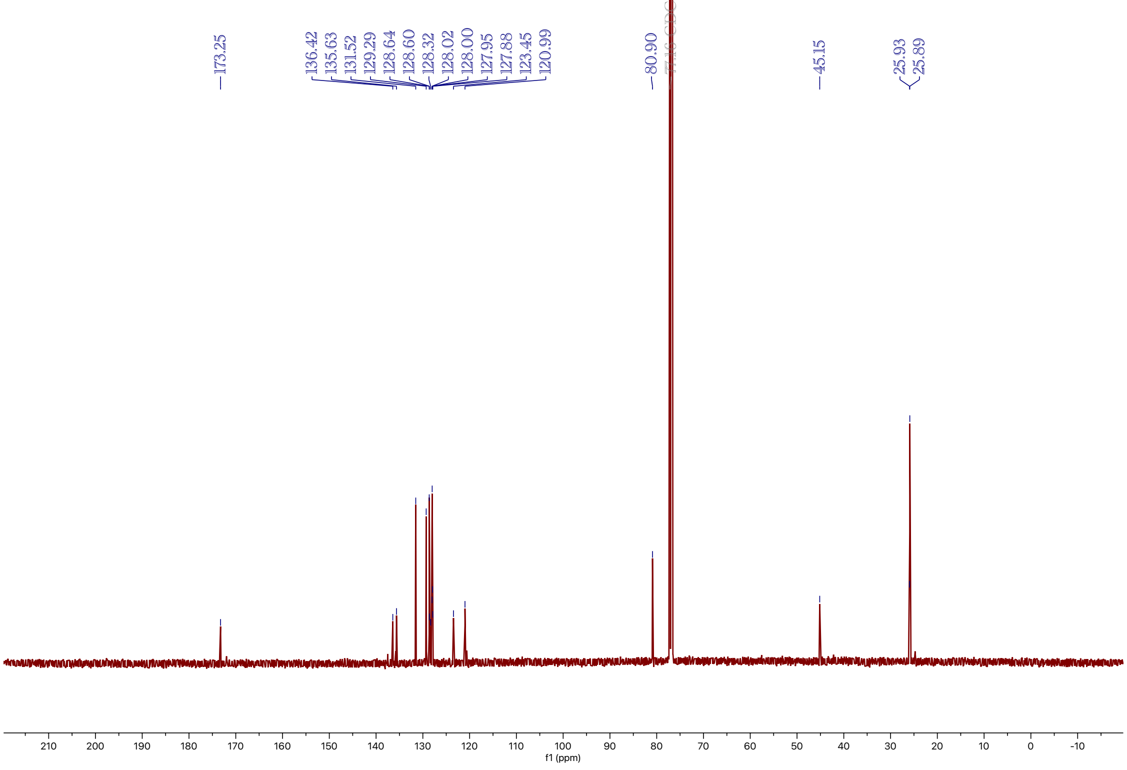

NS-3,5-diOME-SM-1H.10.fid

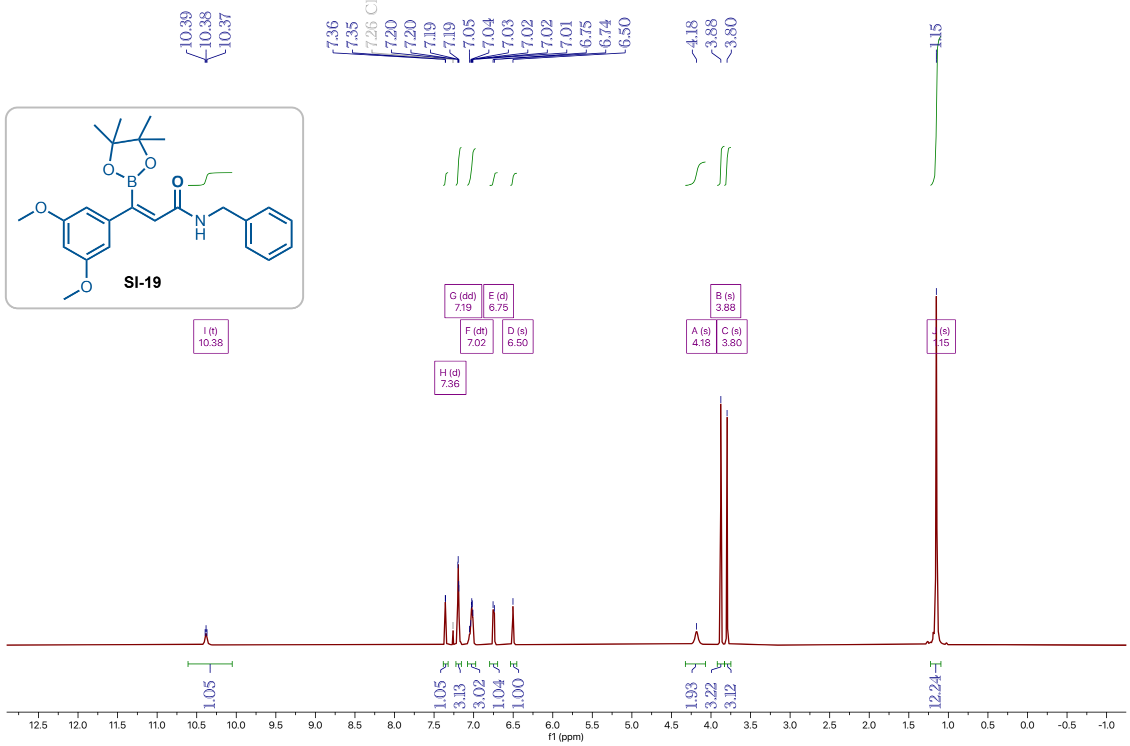

NS-3,5-diOME-SM-13C.12.fid

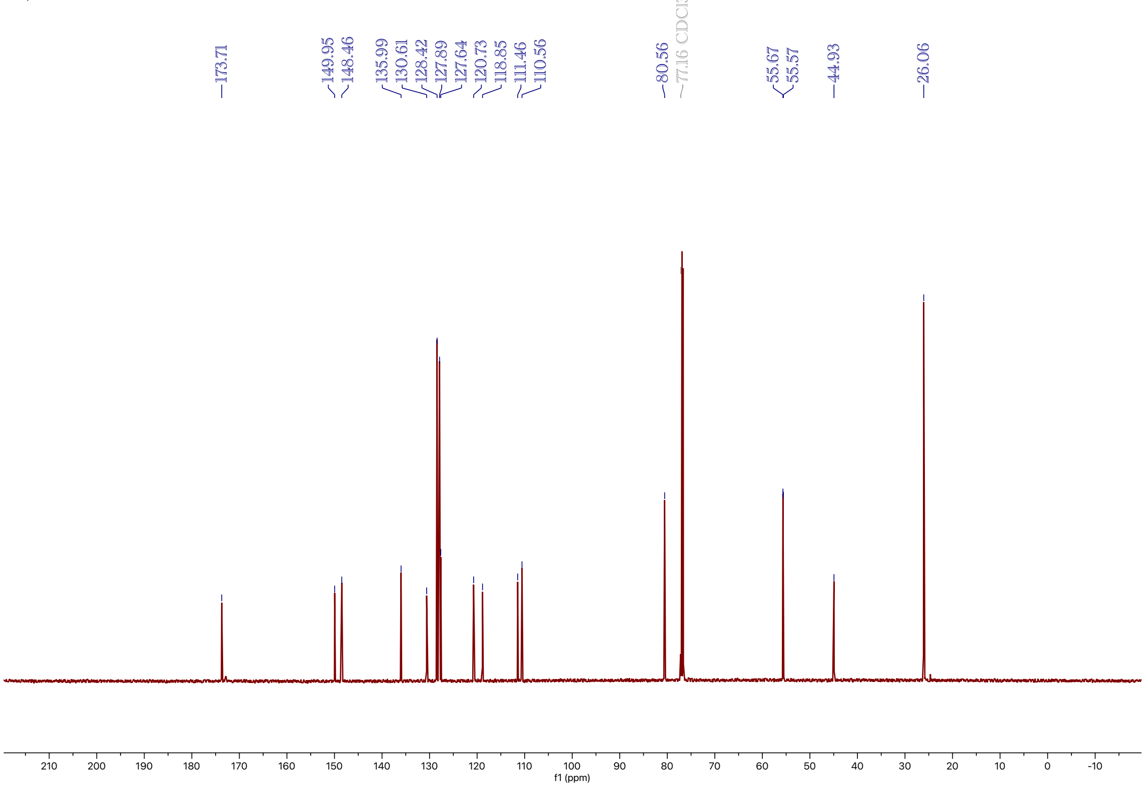

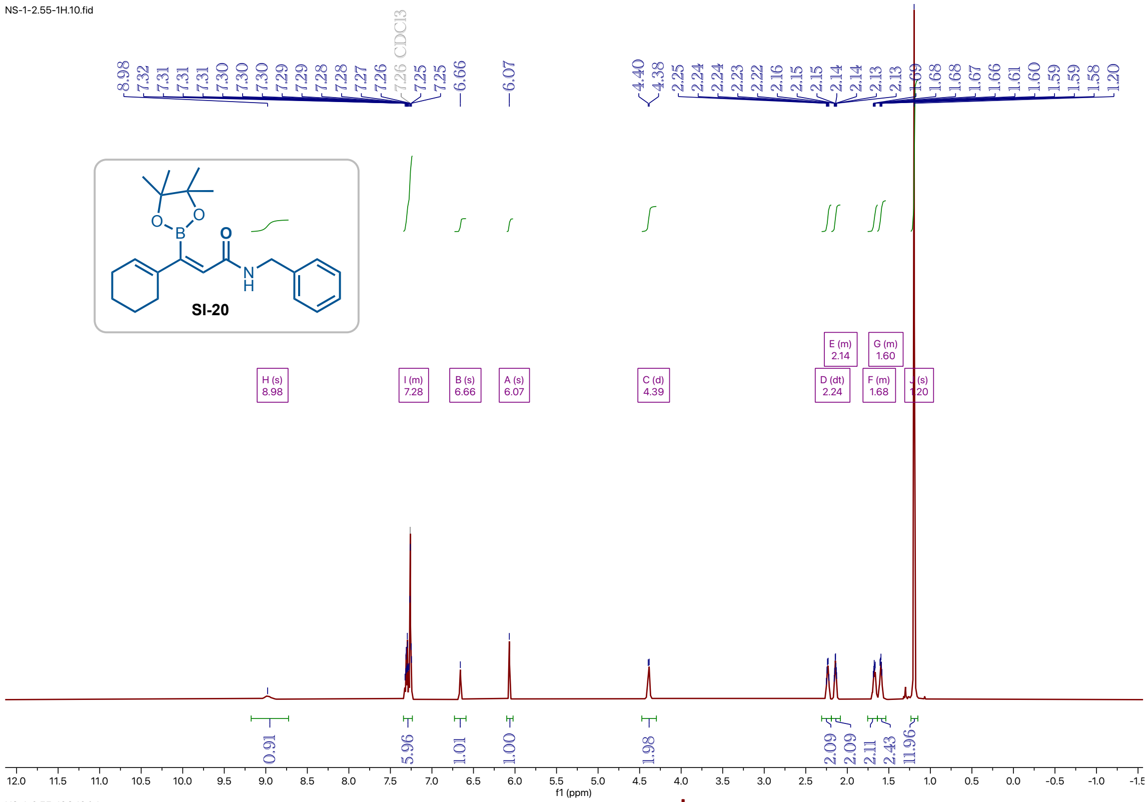
NS-1-2.55-1H.10.fid
8.98 7.32 7.31 7.31 7.31 7.30 7.30 7.30 7.29 7.29 7.28 7.28 7.27 7.26 7.26 CDCl3 7.25 7.25 6.66 6.07
4.40 4.38 2.25 2.24 2.24 2.23 2.22 2.16 2.15 2.15 2.14 2.14 2.13 2.13 1.69 1.68 1.68 1.67 1.66 1.61 1.60 1.59 1.59 1.58 1.20
SI-20
E (m) 2.14
G (m) 1.60
H (s) 8.98
I (m) 7.28
B (s) 6.66
A (s) 6.07
C (d) 4.39
D (dt) 2.24
F (m) 1.68
J (s) 1.20
0.91 5.96 1.01 1.00 1.98 2.09 2.09 2.11 2.43 11.96
f1 (ppm)

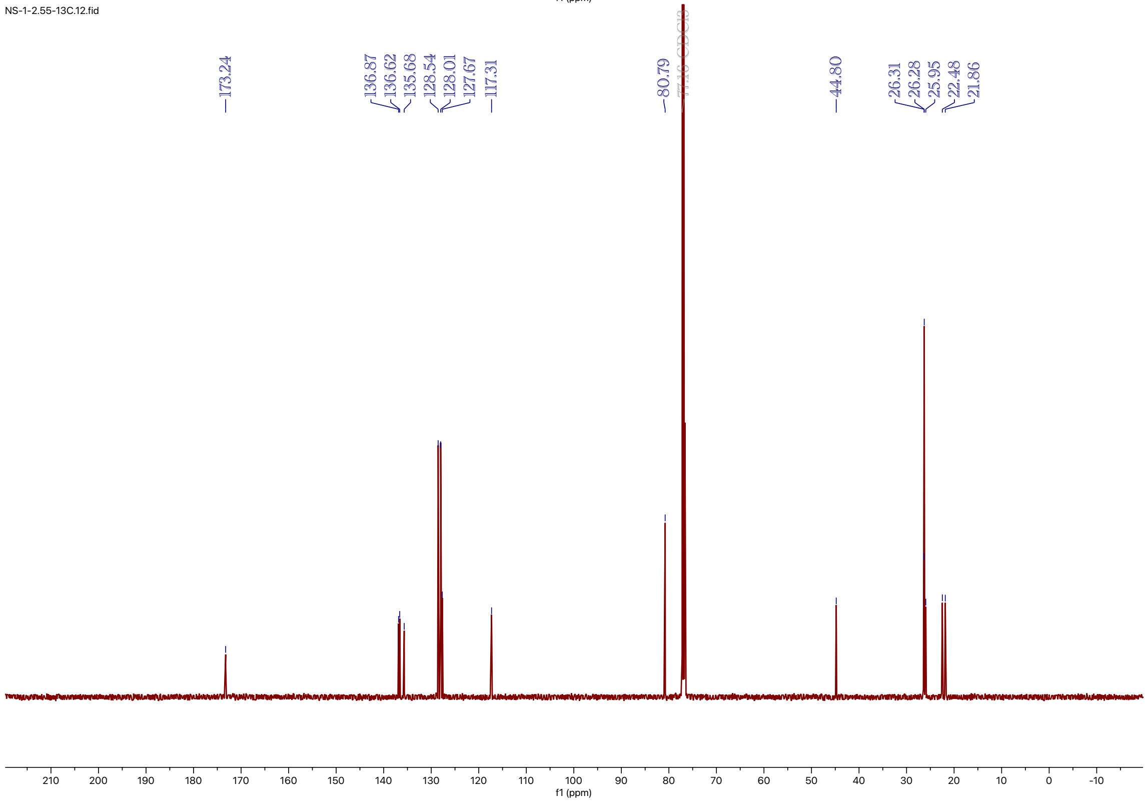
NS-1-2.55-13C.12.fid
173.24
136.87 136.62 135.68 128.54 128.01 127.67 117.31
80.79 77.16 CDCl3
44.80
26.31 26.28 25.95 22.48 21.86
f1 (ppm)

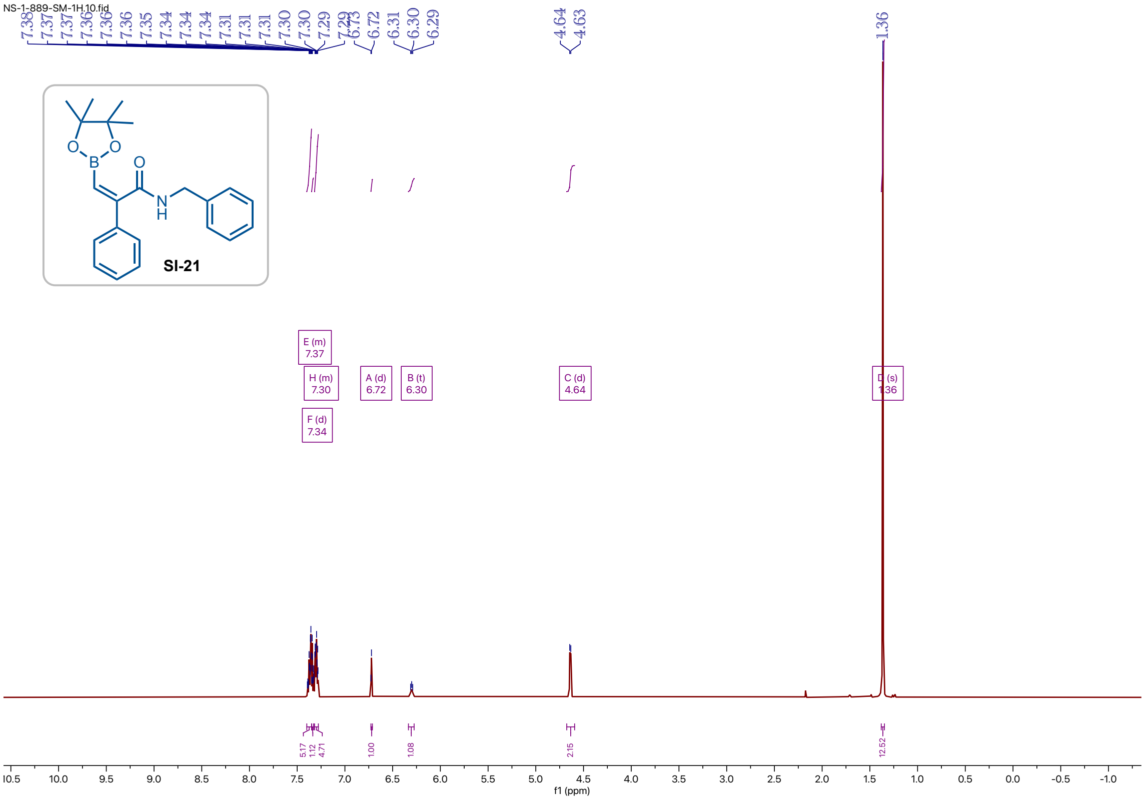
NS-1-889-SM-1H.10.fid
7.38 7.37 7.37 7.36 7.36 7.36 7.35 7.34 7.34 7.34 7.31 7.31 7.31 7.30 7.30 7.29 7.29 6.73 6.72 6.31 6.30 6.29
4.64 4.63
1.36
SI-21
E (m) 7.37
H (m) 7.30
F (d) 7.34
A (d) 6.72
B (t) 6.30
C (d) 4.64
D (s) 1.36
5.17 1.12 4.71 1.00 1.08 2.16 12.52
f1 (ppm)

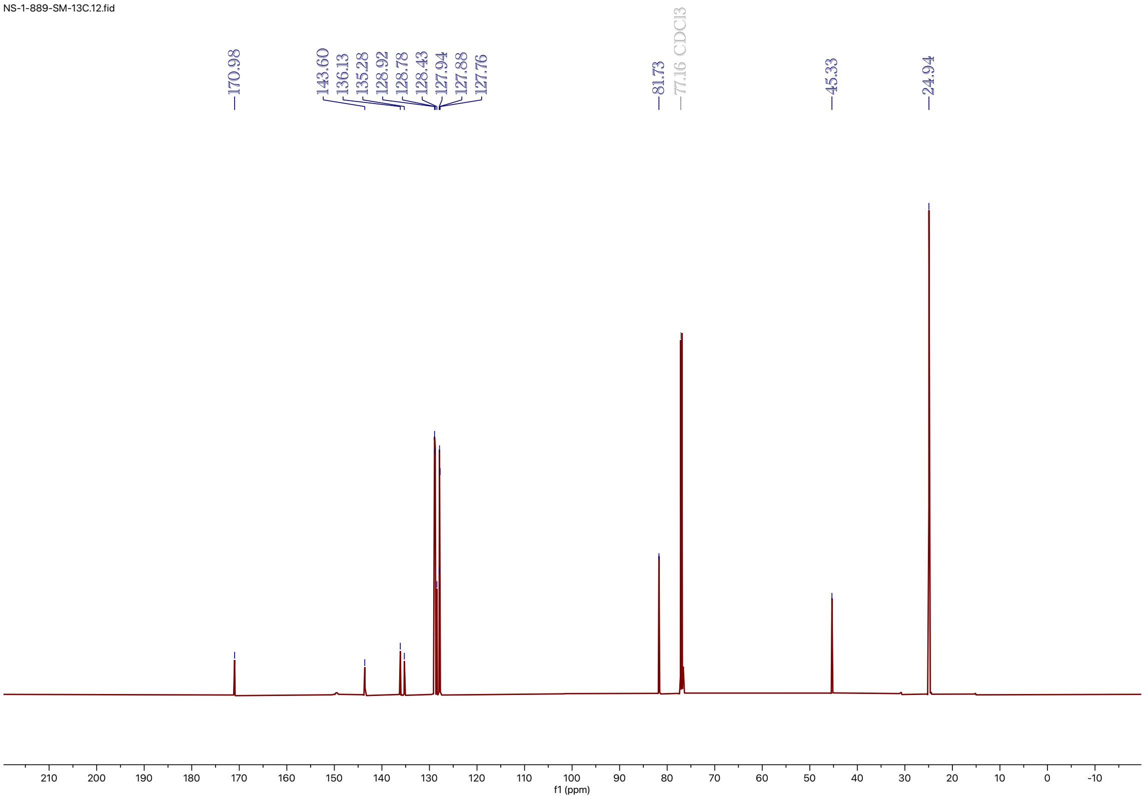
NS-1-889-SM-13C.12.fid
170.98
143.60 136.13 135.28 128.92 128.78 128.43 127.94 127.88 127.76
81.73
77.16 CDCl3
45.33
24.94
f1 (ppm)

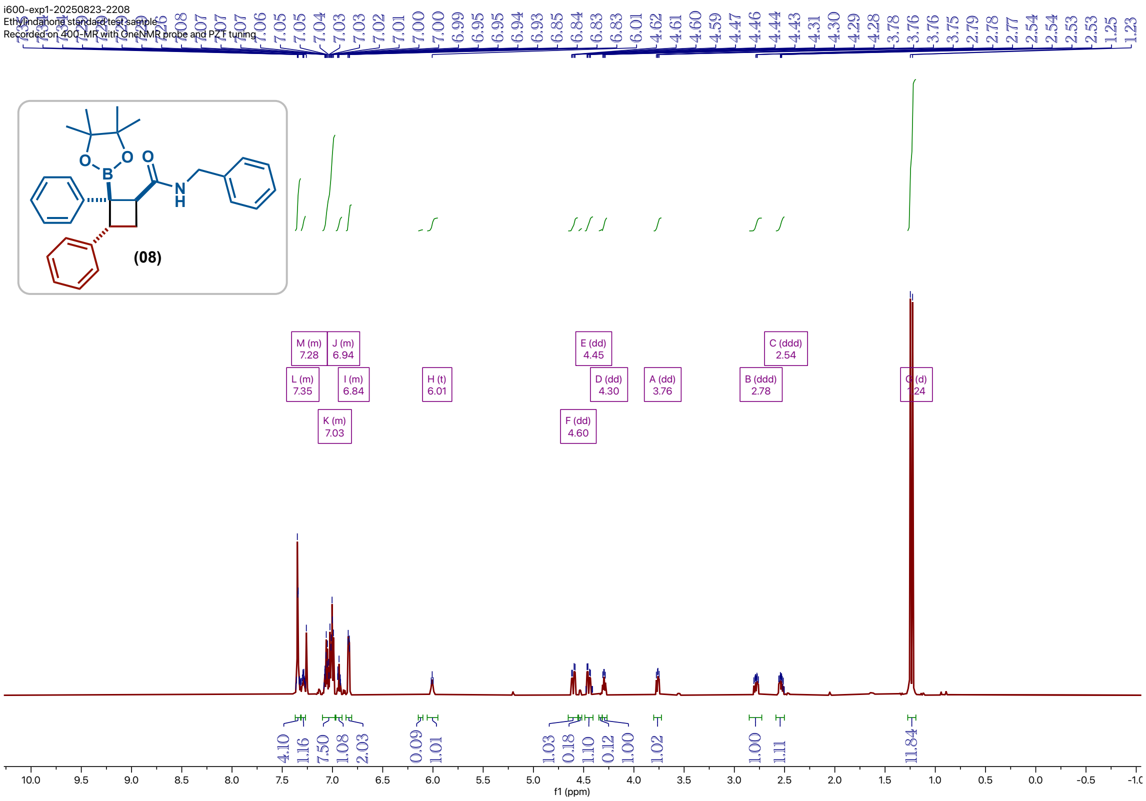
i600-exp1-20250823-2208
(08)
M (m) 7.28
J (m) 6.94
L (m) 7.35
I (m) 6.84
K (m) 7.03
H (t) 6.01
E (dd) 4.45
D (dd) 4.30
F (dd) 4.60
A (dd) 3.76
C (ddd) 2.54
B (ddd) 2.78
G (d) 1.24
4.10
1.16
7.50
1.08
2.03
0.09
1.01
1.03
0.18
1.10
0.12
1.00
1.02
1.00
1.11
11.84
f1 (ppm)

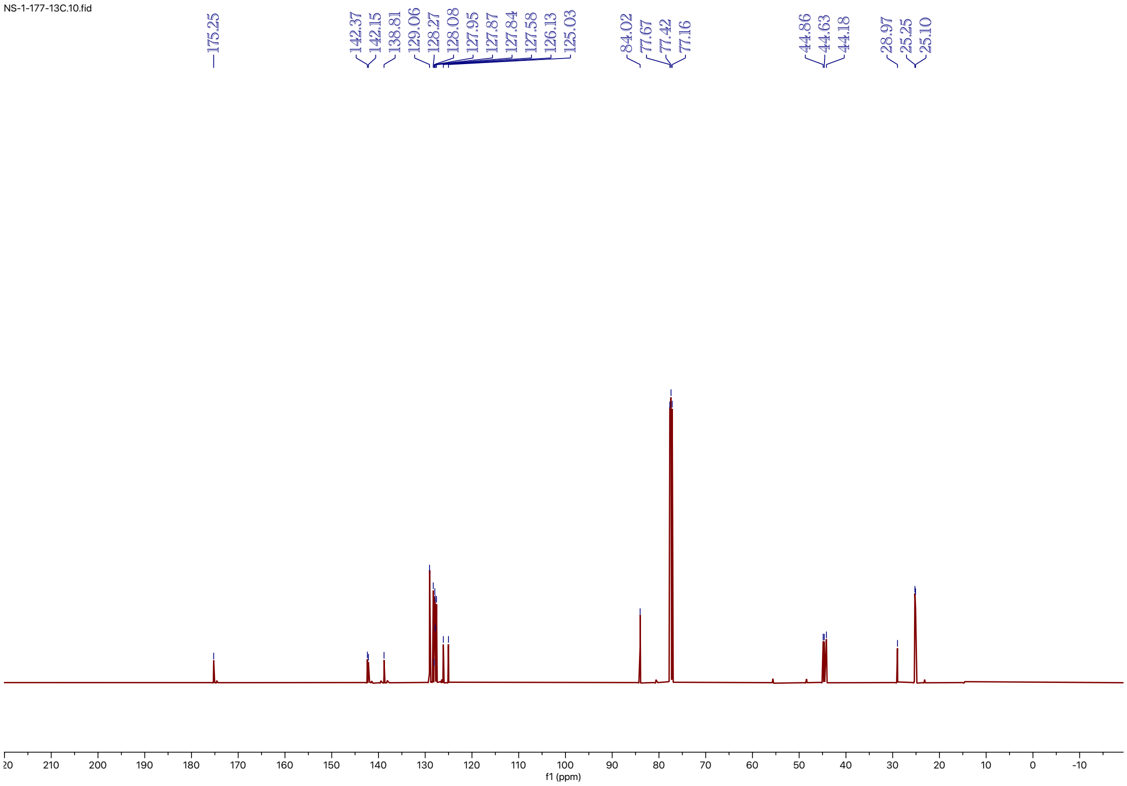
NS-1-177-13C.10.fid
175.25
142.37
142.15
138.81
129.06
128.27
128.08
127.95
127.87
127.84
127.58
126.13
125.03
84.02
77.67
77.42
77.16
44.86
44.63
44.18
28.97
25.25
25.10
f1 (ppm)

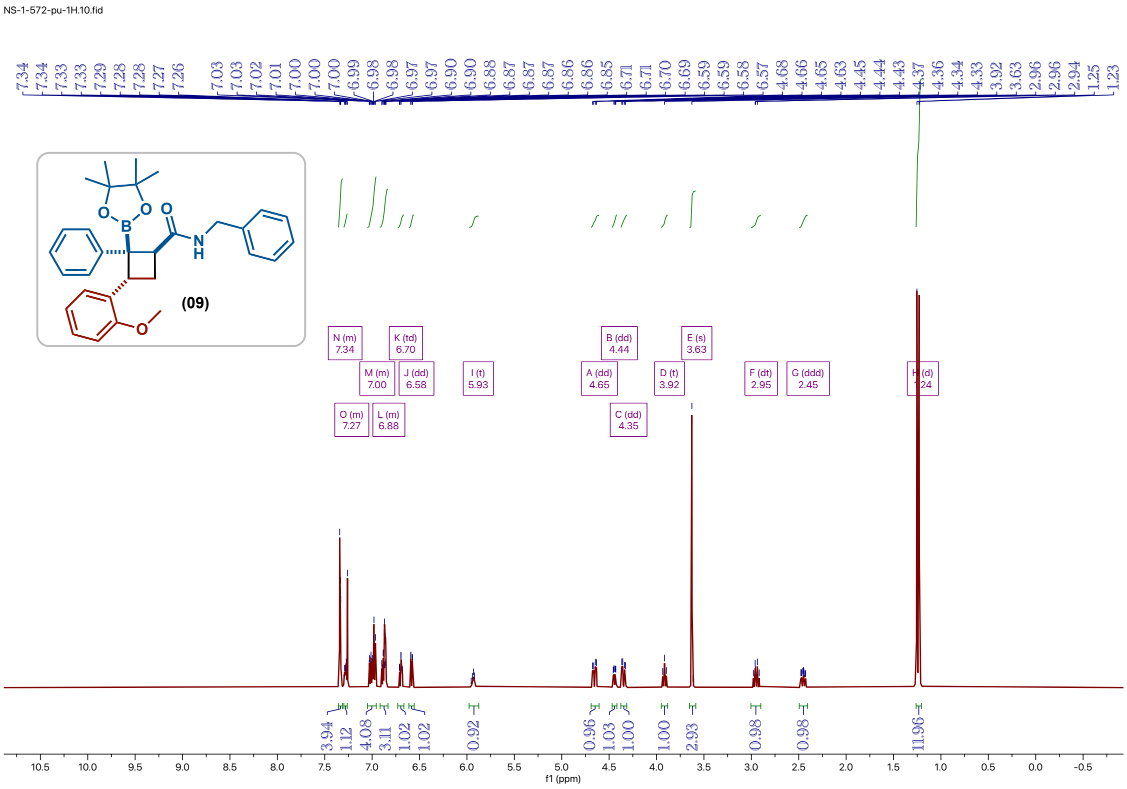

NS-1-572-pu-1H.10.fid
7.34 7.34 7.33 7.33 7.29 7.28 7.28 7.27 7.26 7.03 7.03 7.02 7.01 7.00 7.00 7.00 6.99 6.98 6.98 6.97 6.97 6.90 6.88 6.87 6.87 6.87 6.86 6.86 6.85 6.71 6.71 6.70 6.69 6.59 6.59 6.58 6.57 4.68 4.66 4.65 4.63 4.45 4.44 4.43 4.37 4.36 4.34 4.33 3.92 3.63 2.96 2.96 2.94 1.25 1.23
O
B
O
O
N
H
O
(09)
N (m) 7.34
K (td) 6.70
B (dd) 4.44
E (s) 3.63
M (m) 7.00
J (dd) 6.58
I (t) 5.93
A (dd) 4.65
D (t) 3.92
F (dt) 2.95
G (ddd) 2.45
H (d) 1.24
O (m) 7.27
L (m) 6.88
C (dd) 4.35
3.94 1.12 4.08 3.11 1.02 1.02 0.92 0.96 1.03 1.00 1.00 2.93 0.98 0.98 11.96
10.5 10.0 9.5 9.0 8.5 8.0 7.5 7.0 6.5 6.0 5.5 5.0 4.5 4.0 3.5 3.0 2.5 2.0 1.5 1.0 0.5 0.0 -0.5
f1 (ppm)

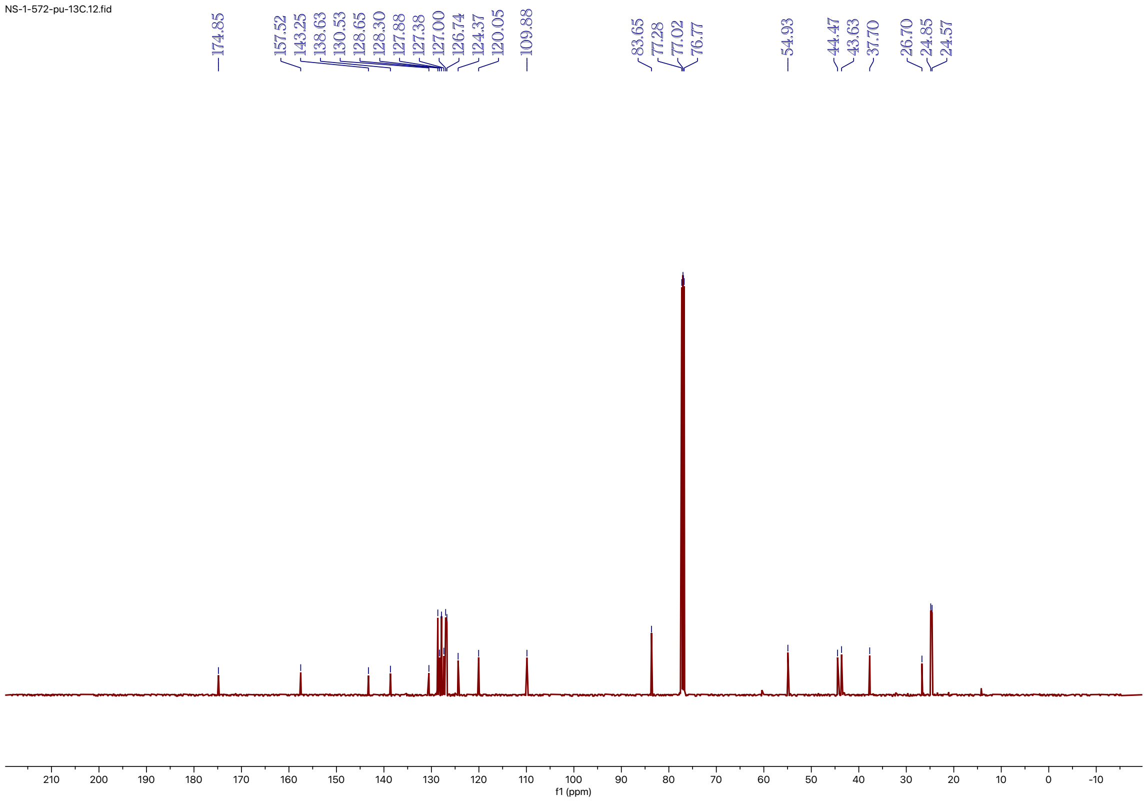

NS-1-572-pu-13C.12.fid
174.85 157.52 143.25 138.63 130.53 128.65 128.30 127.88 127.38 127.00 126.74 124.37 120.05 109.88 83.65 77.28 77.02 76.77 54.93 44.47 43.63 37.70 26.70 24.85 24.57
210 200 190 180 170 160 150 140 130 120 110 100 90 80 70 60 50 40 30 20 10 0 -10
f1 (ppm)

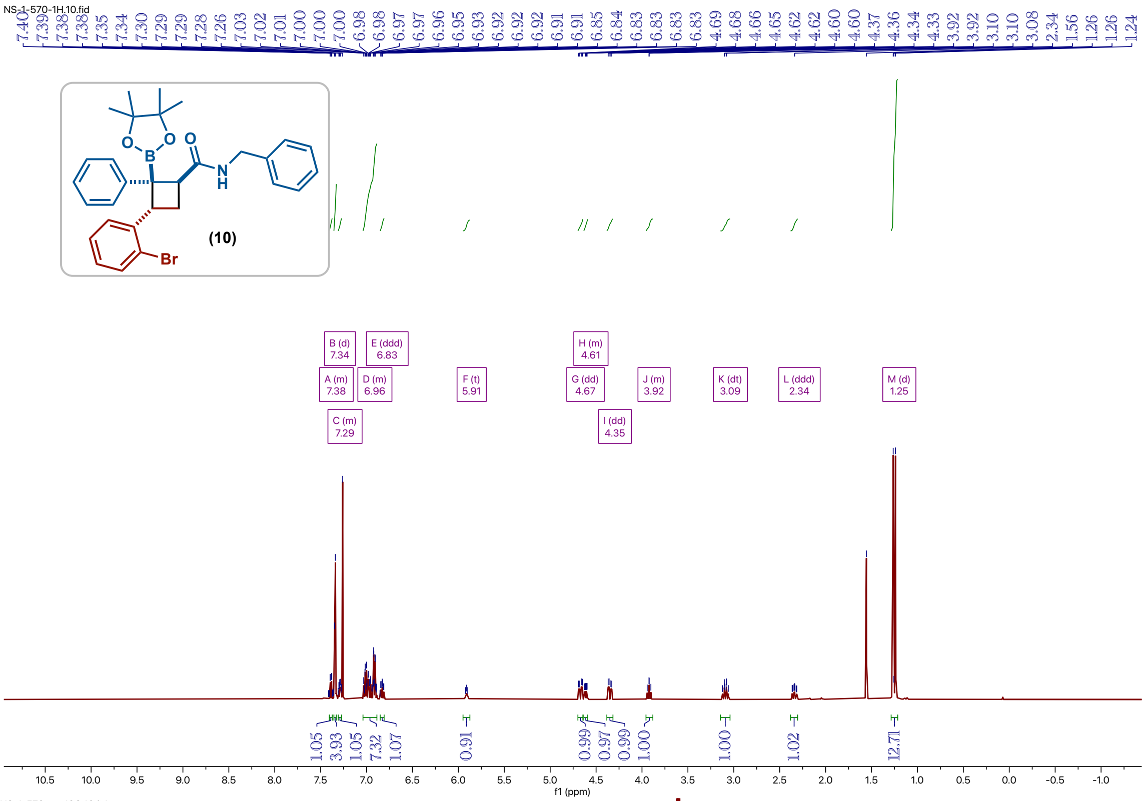
NS-1-570-1H.10.fid
7.40 7.39 7.38 7.38 7.35 7.34 7.30 7.29 7.29 7.28 7.26 7.03 7.02 7.01 7.00 7.00 7.00 6.98 6.98 6.97 6.97 6.96 6.95 6.93 6.92 6.92 6.92 6.91 6.91 6.85 6.84 6.83 6.83 6.83 6.83 4.69 4.68 4.66 4.65 4.62 4.62 4.60 4.60 4.37 4.36 4.34 4.33 3.92 3.92 3.10 3.10 3.08 2.34 1.56 1.26 1.26 1.24
O
B
O
O
N
H
Br
(10)
B (d) 7.34
E (ddd) 6.83
H (m) 4.61
A (m) 7.38
D (m) 6.96
F (t) 5.91
G (dd) 4.67
J (m) 3.92
K (dt) 3.09
L (ddd) 2.34
M (d) 1.25
C (m) 7.29
I (dd) 4.35
1.05 3.93 1.05 7.32 1.07 0.91 0.99 0.97 0.99 1.00 1.00 1.02 12.71
f1 (ppm)

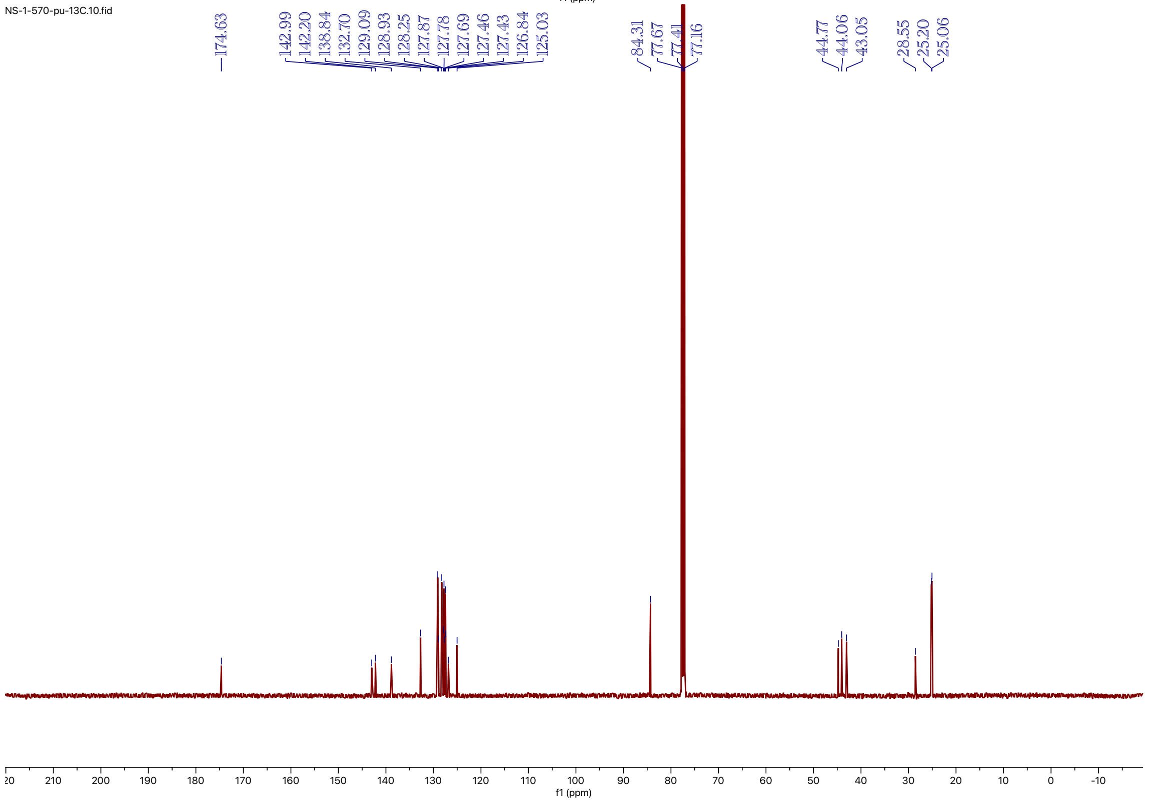
NS-1-570-pu-13C.10.fid
174.63
142.99 142.20 138.84 132.70 129.09 128.93 128.25 127.87 127.78 127.69 127.46 127.43 126.84 125.03
84.31 77.67 77.41 77.16
44.77 44.06 43.05
28.55 25.20 25.06
f1 (ppm)

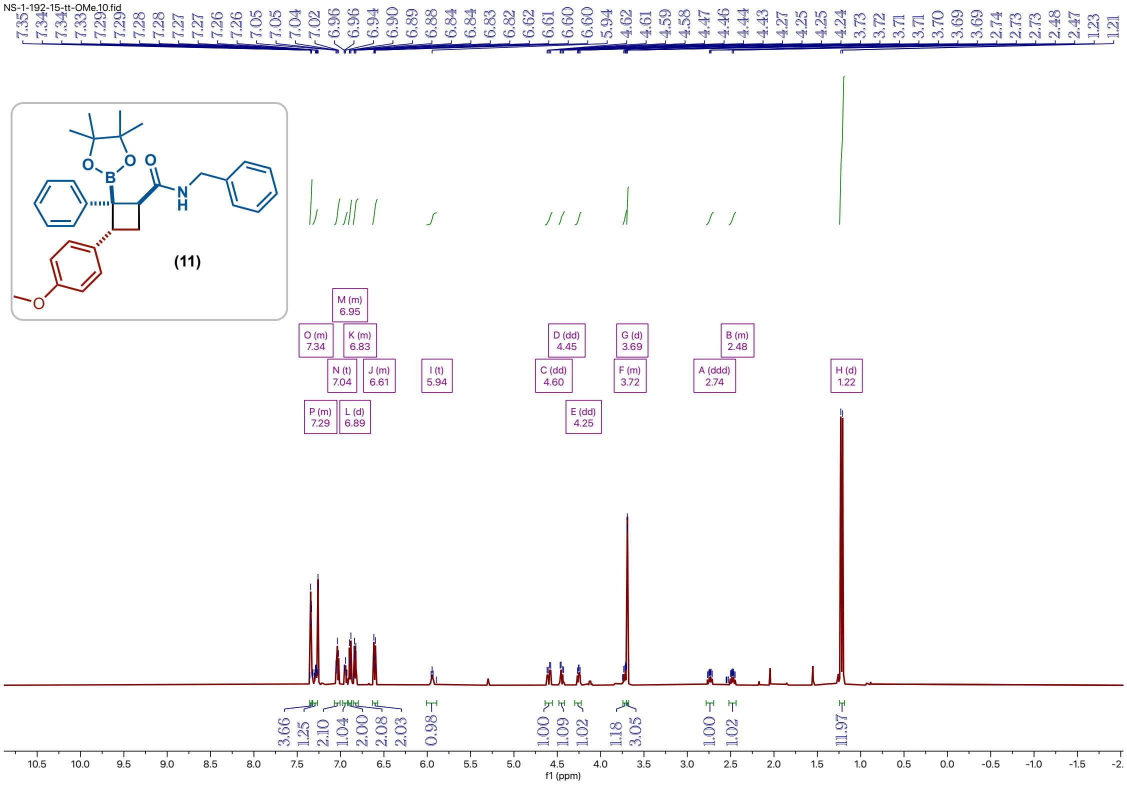
NS-1-192-15-tt-OMe.10.fid
7.35 7.34 7.34 7.33 7.29 7.29 7.28 7.28 7.27 7.27 7.26 7.26 7.05 7.05 7.04 7.02 6.96 6.96 6.94 6.90 6.89 6.88 6.84 6.84 6.83 6.82 6.62 6.61 6.60 6.60 5.94 4.62 4.61 4.59 4.58 4.47 4.46 4.44 4.43 4.27 4.25 4.25 4.24 3.73 3.72 3.71 3.71 3.70 3.69 3.69 2.74 2.73 2.73 2.48 2.47 1.23 1.21
(11)
M (m) 6.95
O (m) 7.34
K (m) 6.83
N (t) 7.04
J (m) 6.61
P (m) 7.29
L (d) 6.89
I (t) 5.94
D (dd) 4.45
C (dd) 4.60
E (dd) 4.25
G (d) 3.69
F (m) 3.72
B (m) 2.48
A (ddd) 2.74
H (d) 1.22
3.66 1.25 2.10 1.04 2.00 2.08 2.03 0.98 1.00 1.09 1.02 1.18 3.05 1.00 1.02 11.97
f1 (ppm)

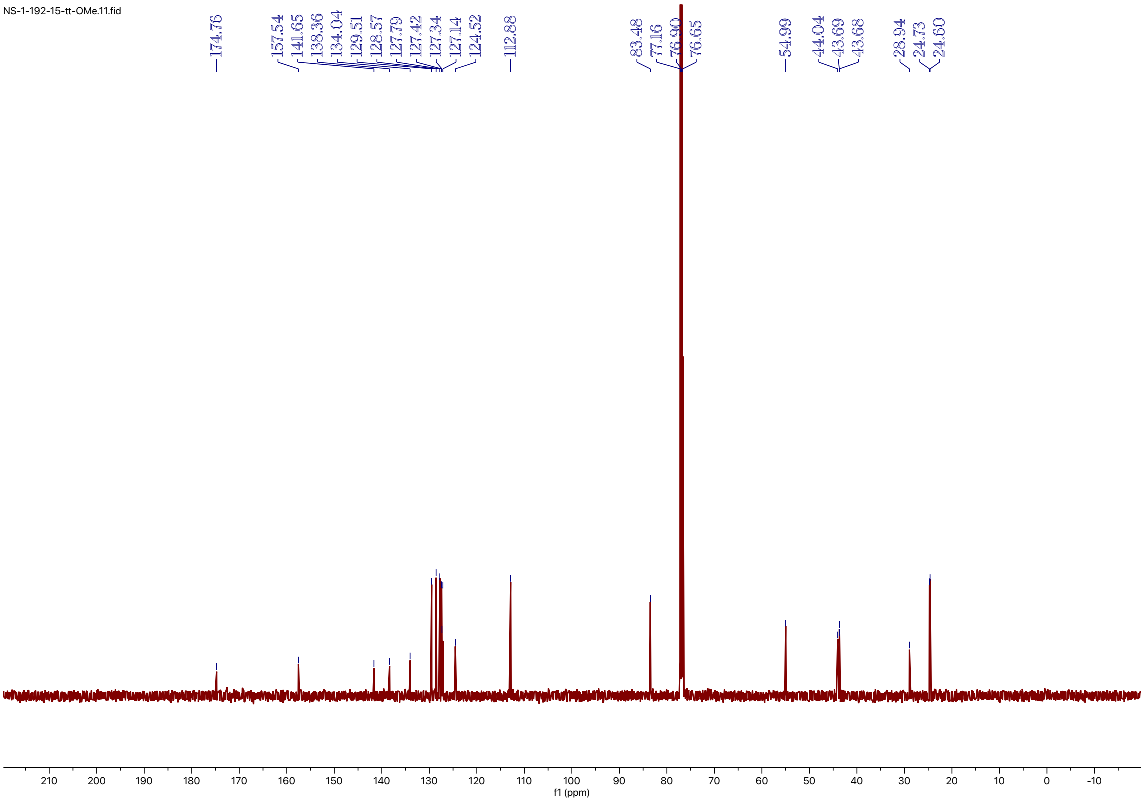
NS-1-192-15-tt-OMe.11.fid
174.76
157.54 141.65 138.36 134.04 129.51 128.57 127.79 127.42 127.34 127.14 124.52
112.88
83.48 77.16 76.90 76.65
54.99
44.04 43.69 43.68
28.94 24.73 24.60
f1 (ppm)

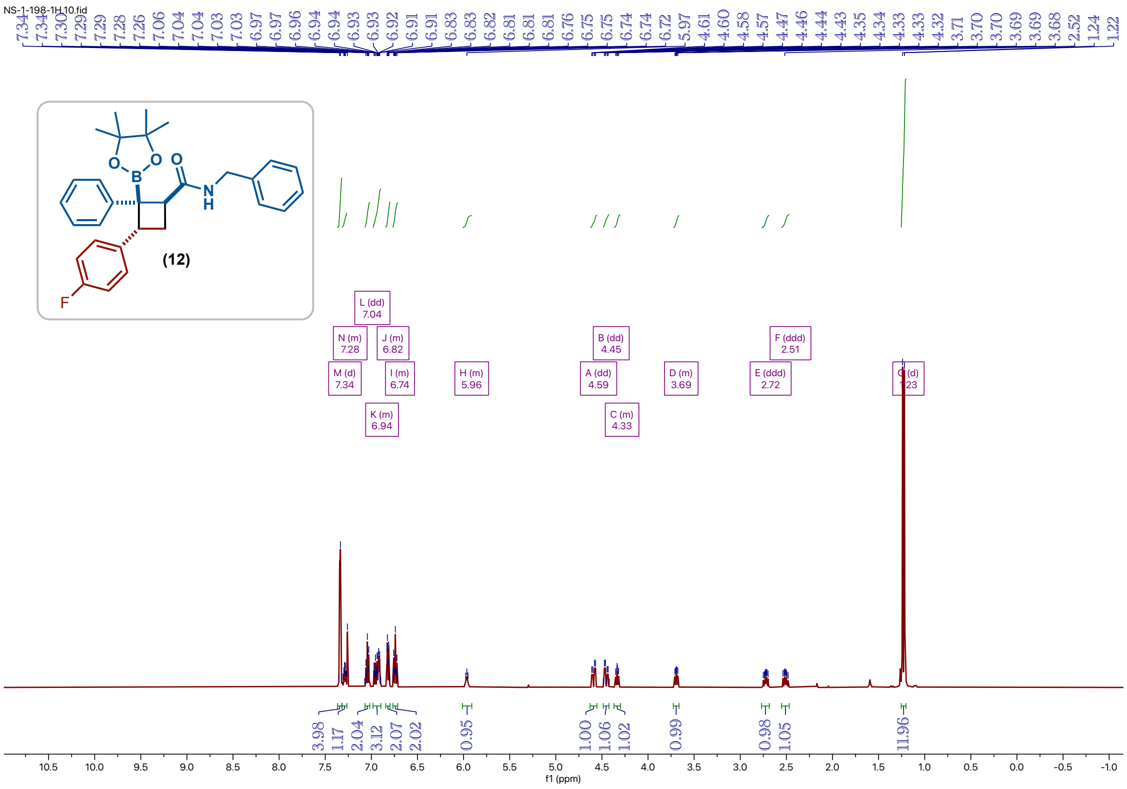
NS-1-198-1H.10.fid
7.34 7.34 7.30 7.29 7.29 7.28 7.26 7.06 7.04 7.04 7.03 7.03 6.97 6.97 6.96 6.94 6.94 6.93 6.93 6.92 6.91 6.91 6.83 6.83 6.82 6.81 6.81 6.81 6.76 6.75 6.75 6.74 6.74 6.72 5.97 4.61 4.60 4.58 4.57 4.47 4.46 4.44 4.43 4.35 4.34 4.33 4.33 4.32 3.71 3.70 3.70 3.69 3.69 3.68 2.52 1.24 1.22
O
B
O
O
N
H
F
(12)
L (dd) 7.04
N (m) 7.28
J (m) 6.82
M (d) 7.34
I (m) 6.74
K (m) 6.94
H (m) 5.96
B (dd) 4.45
A (dd) 4.59
C (m) 4.33
D (m) 3.69
F (ddd) 2.51
E (ddd) 2.72
G (d) 1.23
3.98 1.17 2.04 3.12 2.07 2.02 0.95 1.00 1.06 1.02 0.99 0.98 1.05 11.96
10.5 10.0 9.5 9.0 8.5 8.0 7.5 7.0 6.5 6.0 5.5 5.0 4.5 4.0 3.5 3.0 2.5 2.0 1.5 1.0 0.5 0.0 -0.5 -1.0
f1 (ppm)

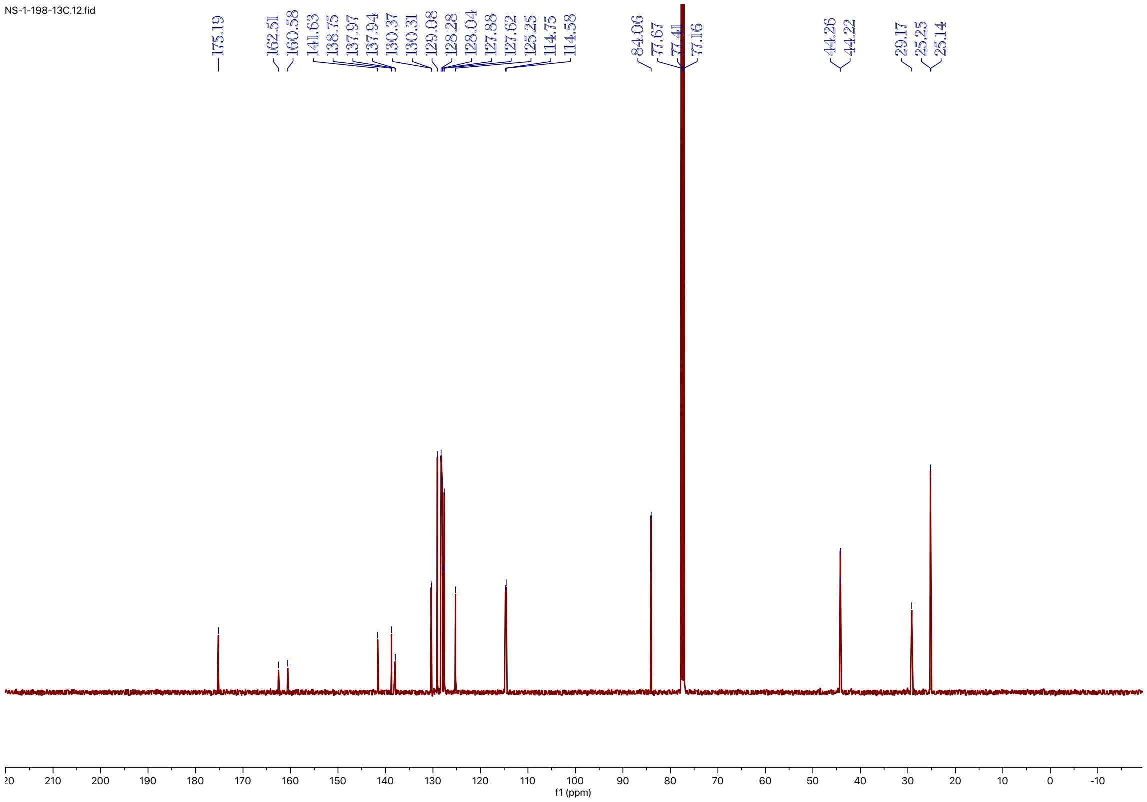
NS-1-198-13C.12.fid
175.19 162.51 160.58 141.63 138.75 137.97 137.94 130.37 130.31 129.08 128.28 128.04 127.88 127.62 125.25 114.75 114.58 84.06 77.67 77.41 77.16 44.26 44.22 29.17 25.25 25.14
210 200 190 180 170 160 150 140 130 120 110 100 90 80 70 60 50 40 30 20 10 0 -10
f1 (ppm)

NS-1-198-19F.14.fid

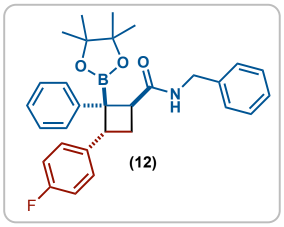

(12)

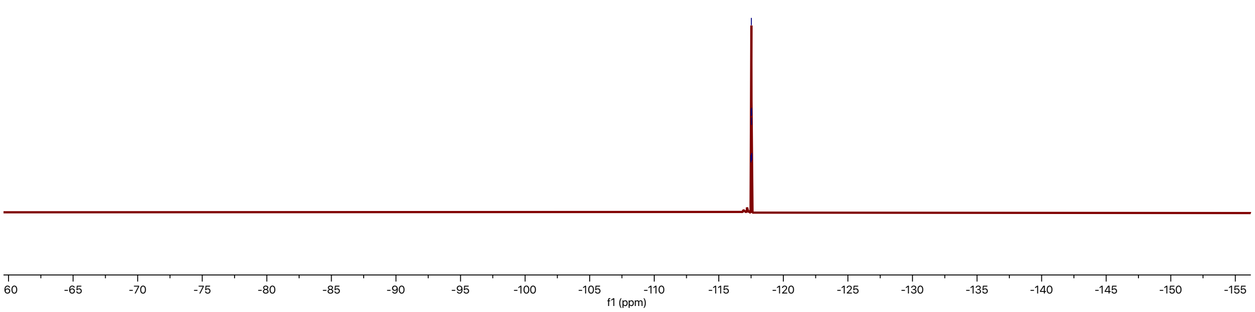

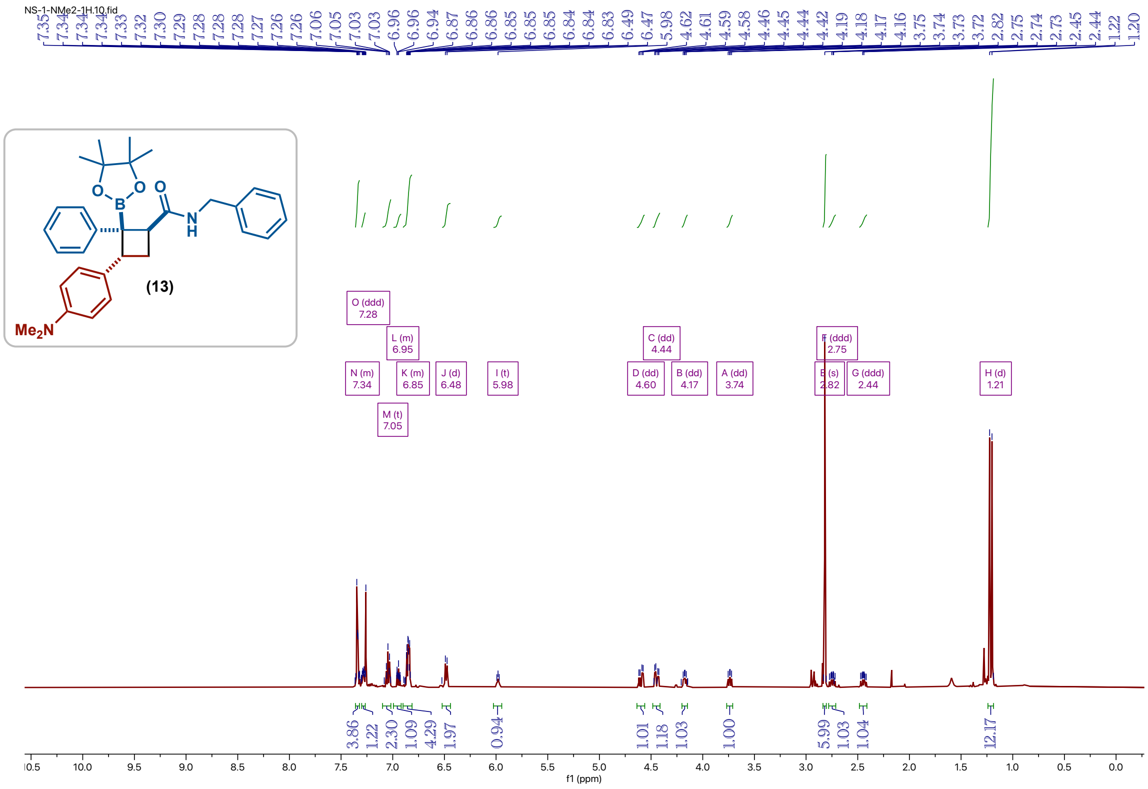
NS-1-NMe2-1H.10.fid
7.35 7.34 7.34 7.34 7.33 7.32 7.30 7.29 7.28 7.28 7.28 7.27 7.26 7.26 7.06 7.05 7.03 7.03 6.96 6.96 6.94 6.87 6.86 6.86 6.85 6.85 6.85 6.84 6.84 6.83 6.49 6.47 5.98 4.62 4.61 4.59 4.58 4.46 4.45 4.44 4.42 4.19 4.18 4.17 4.16 3.75 3.74 3.73 3.72 2.82 2.75 2.74 2.73 2.45 2.44 1.22 1.20
O
B
O
O
N
H
Me2N
(13)
O (ddd) 7.28
L (m) 6.95
N (m) 7.34
K (m) 6.85
J (d) 6.48
I (t) 5.98
M (t) 7.05
C (dd) 4.44
D (dd) 4.60
B (dd) 4.17
A (dd) 3.74
F (ddd) 2.75
E (s) 2.82
G (ddd) 2.44
H (d) 1.21
3.86 1.22 2.30 1.09 4.29 1.97 0.94 1.01 1.18 1.03 1.00 5.99 1.03 1.04 12.17
f1 (ppm)

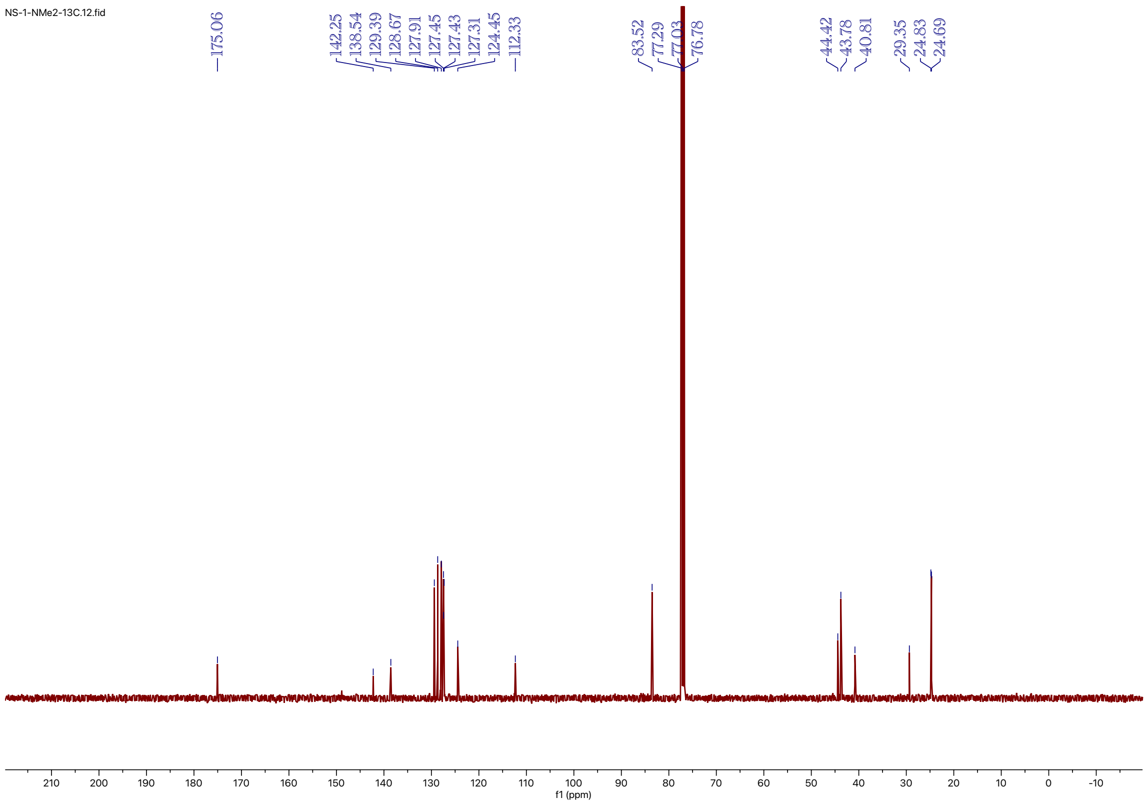
NS-1-NMe2-13C.12.fid
175.06
142.25 138.54 129.39 128.67 127.91 127.45 127.43 127.31 124.45 112.33
83.52 77.29 77.03 76.78
44.42 43.78 40.81 29.35 24.83 24.69
f1 (ppm)

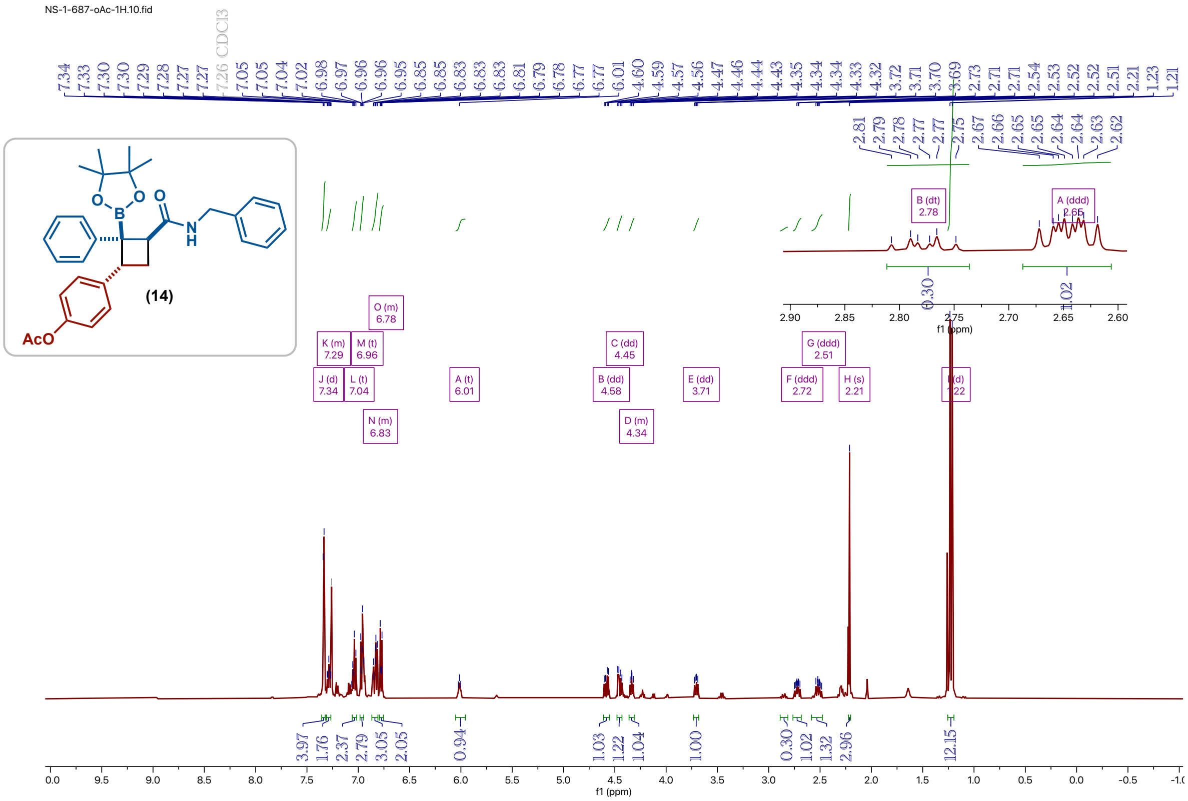

AcO
(14)
NS-1-687-oAc-1H.10.fid
7.34 7.33 7.30 7.30 7.29 7.28 7.27 7.27 7.26 CDCl3 7.05 7.05 7.04 7.02 6.98 6.97 6.96 6.96 6.95 6.85 6.85 6.83 6.83 6.83 6.81 6.79 6.78 6.77 6.77 6.01 4.60 4.59 4.57 4.56 4.47 4.46 4.44 4.43 4.35 4.34 4.34 4.33 4.32 3.72 3.71 3.70 3.69 2.73 2.71 2.71 2.54 2.53 2.52 2.52 2.51 2.21 1.23 1.21
2.81 2.79 2.78 2.77 2.77 2.75 2.67 2.66 2.65 2.65 2.64 2.64 2.63 2.62
B (dt) 2.78
A (ddd) 2.65
0.30
1.02
2.90 2.85 2.80 2.75 2.70 2.65 2.60
f1 (ppm)
O (m) 6.78
K (m) 7.29
M (t) 6.96
C (dd) 4.45
G (ddd) 2.51
J (d) 7.34
L (t) 7.04
A (t) 6.01
B (dd) 4.58
E (dd) 3.71
F (ddd) 2.72
H (s) 2.21
I (d) 1.22
N (m) 6.83
D (m) 4.34
3.97 1.76 2.37 2.79 3.05 2.05 0.94 1.03 1.22 1.04 1.00 0.30 1.02 1.32 2.96 12.15
10.0 9.5 9.0 8.5 8.0 7.5 7.0 6.5 6.0 5.5 5.0 4.5 4.0 3.5 3.0 2.5 2.0 1.5 1.0 0.5 0.0 -0.5 -1.0
f1 (ppm)

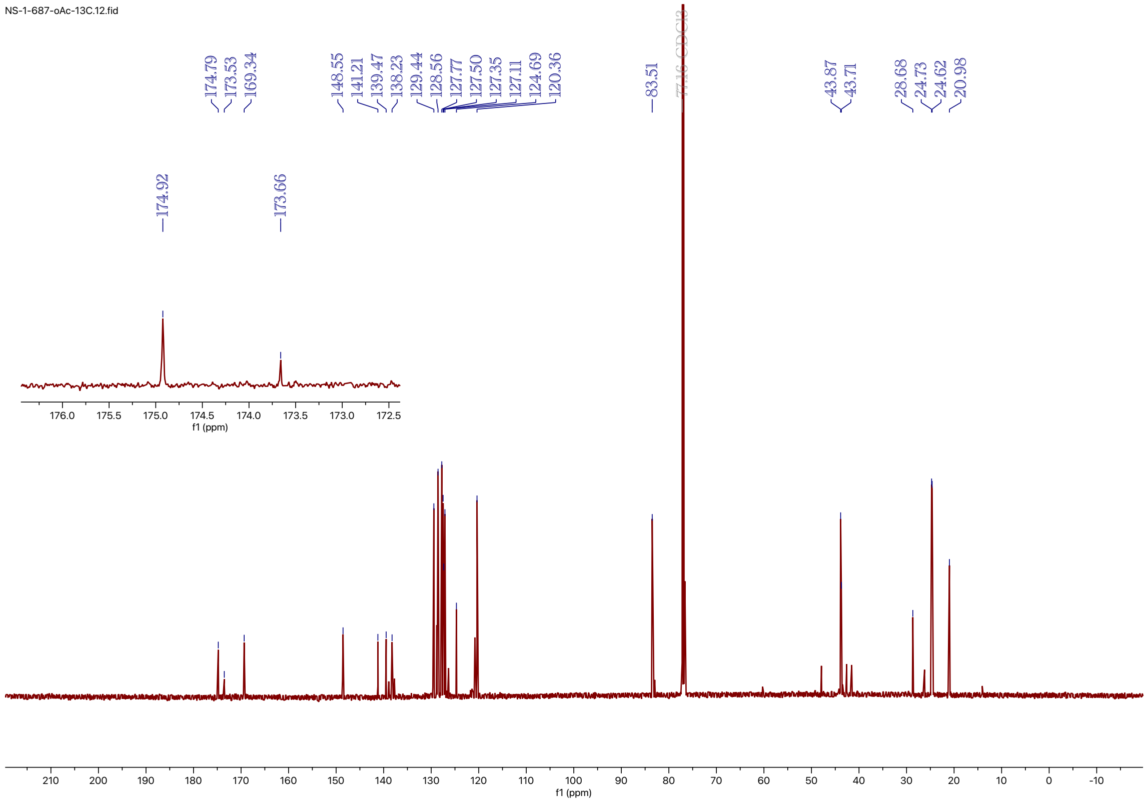

NS-1-687-oAc-13C.12.fid
174.79 173.53 169.34 148.55 141.21 139.47 138.23 129.44 128.56 127.77 127.50 127.35 127.11 124.69 120.36 83.51 77.16 CDCl3 43.87 43.71 28.68 24.73 24.62 20.98
174.92
173.66
176.0 175.5 175.0 174.5 174.0 173.5 173.0 172.5
f1 (ppm)
210 200 190 180 170 160 150 140 130 120 110 100 90 80 70 60 50 40 30 20 10 0 -10
f1 (ppm)

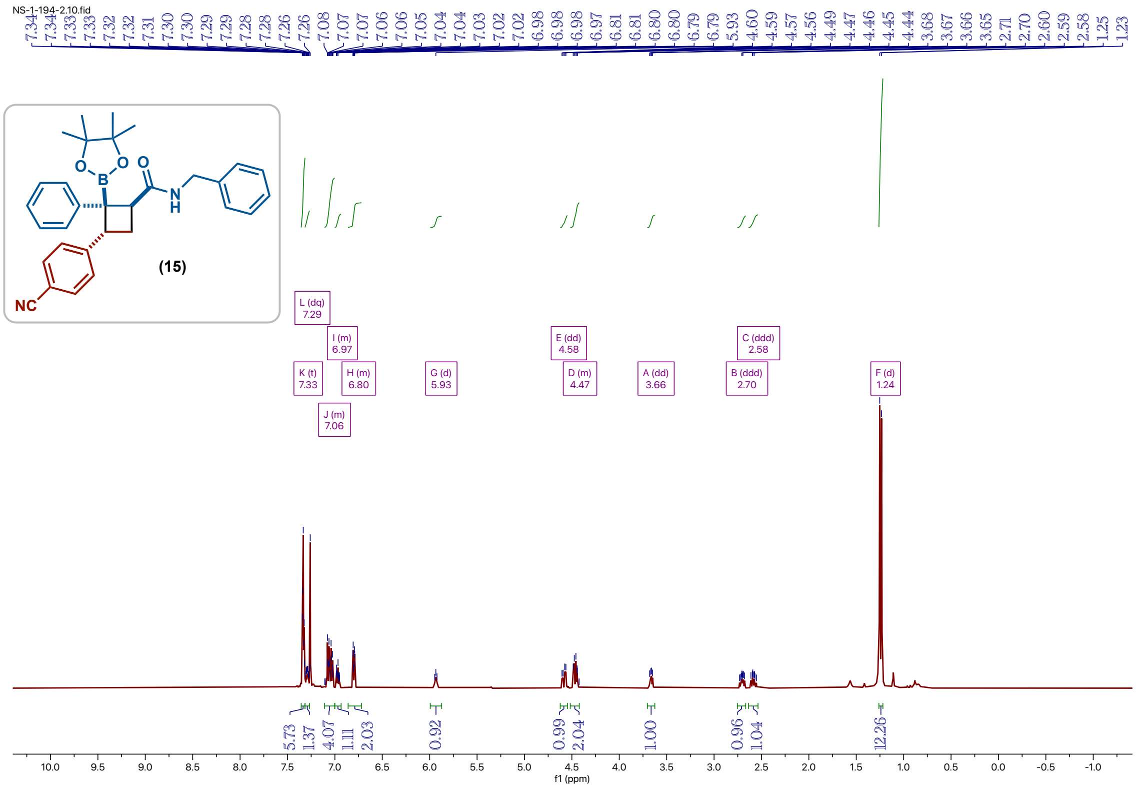
NS-1-194-2.10.fid
7.34 7.34 7.33 7.33 7.32 7.32 7.31 7.30 7.30 7.29 7.29 7.28 7.28 7.26 7.26 7.08 7.07 7.07 7.06 7.06 7.05 7.04 7.04 7.03 7.02 7.02 6.98 6.98 6.98 6.97 6.81 6.81 6.80 6.80 6.79 5.93 4.60 4.59 4.57 4.56 4.49 4.47 4.46 4.45 4.44 3.68 3.67 3.66 3.65 2.71 2.70 2.60 2.59 2.58 1.25 1.23
O
B
O
O
N
H
NC
(15)
L (dq) 7.29
I (m) 6.97
K (t) 7.33
H (m) 6.80
J (m) 7.06
G (d) 5.93
E (dd) 4.58
D (m) 4.47
A (dd) 3.66
C (ddd) 2.58
B (ddd) 2.70
F (d) 1.24
5.73 1.37 4.07 1.11 2.03 0.92 0.99 2.04 1.00 0.96 1.04 12.26
10.0 9.5 9.0 8.5 8.0 7.5 7.0 6.5 6.0 5.5 5.0 4.5 4.0 3.5 3.0 2.5 2.0 1.5 1.0 0.5 0.0 -0.5 -1.0
f1 (ppm)

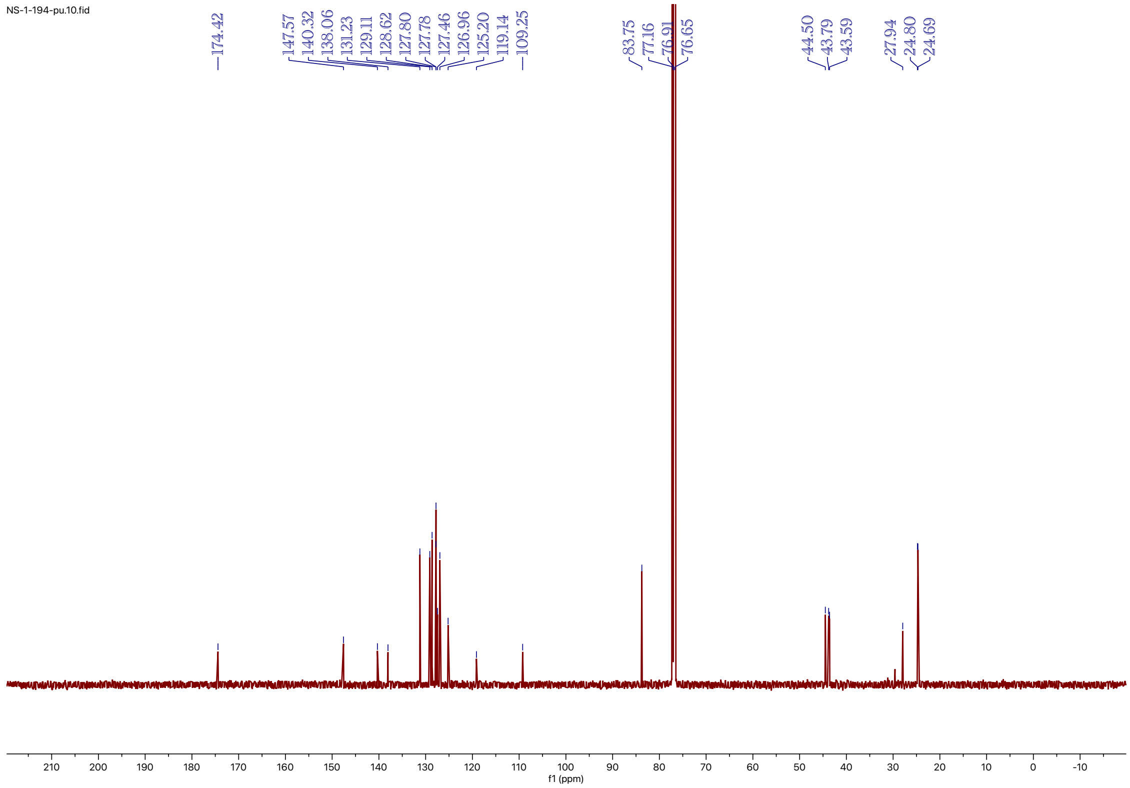
NS-1-194-pu.10.fid
174.42
147.57 140.32 138.06 131.23 129.11 128.62 127.80 127.78 127.46 126.96 125.20 119.14 109.25
83.75 77.16 76.91 76.65
44.50 43.79 43.59
27.94 24.80 24.69
210 200 190 180 170 160 150 140 130 120 110 100 90 80 70 60 50 40 30 20 10 0 -10
f1 (ppm)

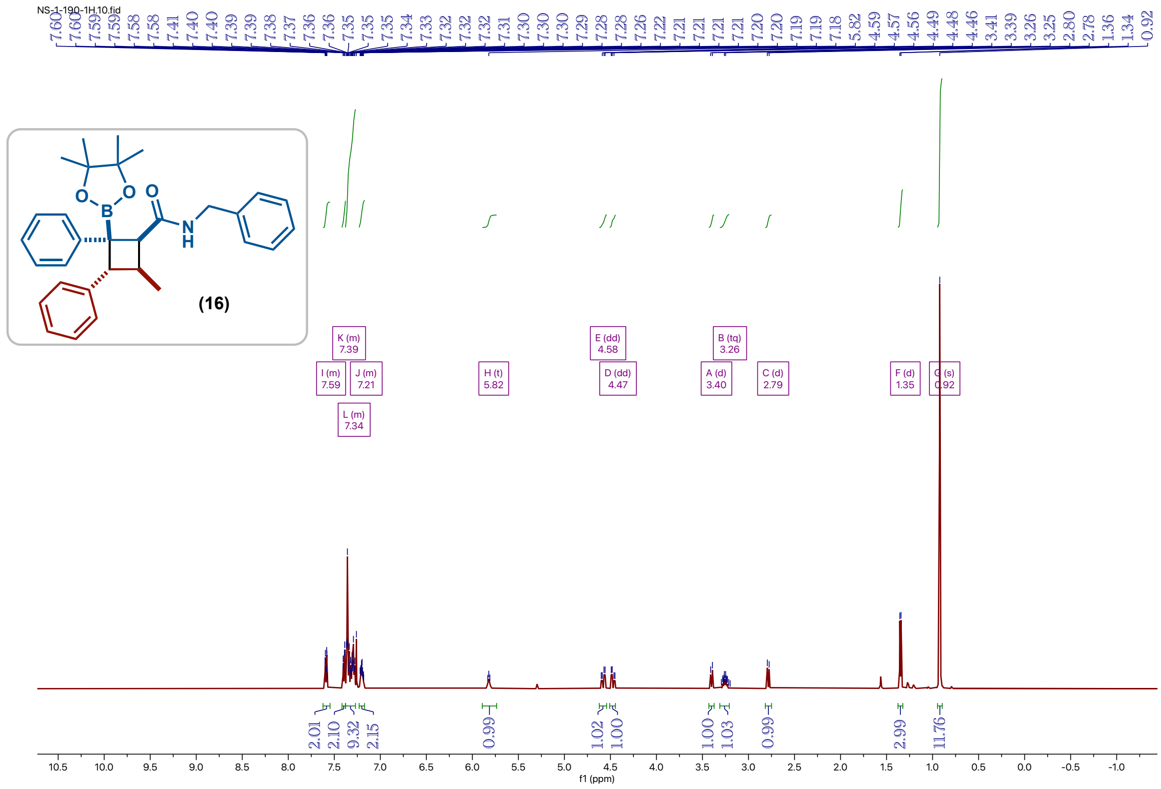
NS-1-190-1H.10.fid
7.60 7.59 7.59 7.59 7.58 7.41 7.40 7.40 7.39 7.39 7.38 7.37 7.36 7.36 7.35 7.35 7.35 7.34 7.33 7.32 7.32 7.32 7.31 7.30 7.30 7.29 7.28 7.28 7.26 7.22 7.21 7.21 7.21 7.21 7.21 7.20 7.20 7.19 7.19 7.18 5.82 4.59 4.57 4.56 4.49 4.48 4.46 3.41 3.39 3.26 3.25 2.80 2.78 1.36 1.34 0.92
(16)
K (m) 7.39
I (m) 7.59
J (m) 7.21
L (m) 7.34
H (t) 5.82
E (dd) 4.58
D (dd) 4.47
B (tq) 3.26
A (d) 3.40
C (d) 2.79
F (d) 1.35
G (s) 0.92
2.01 2.10 9.32 2.15 0.99 1.02 1.00 1.00 1.03 0.99 2.99 11.76
f1 (ppm)

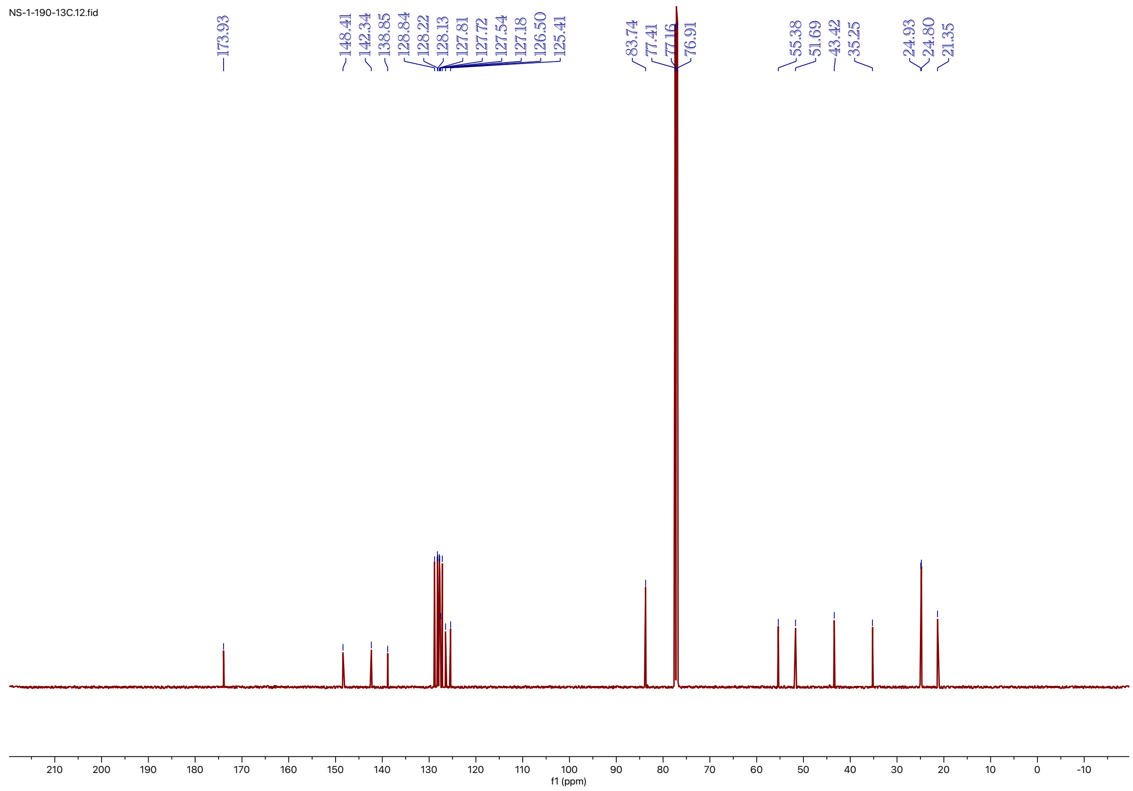
NS-1-190-13C.12.fid
173.93
148.41 142.34 138.85 128.84 128.22 128.13 127.81 127.72 127.54 127.18 126.50 125.41
83.74 77.41 77.16 76.91
55.38 51.69 43.42 35.25
24.93 24.80 21.35
f1 (ppm)

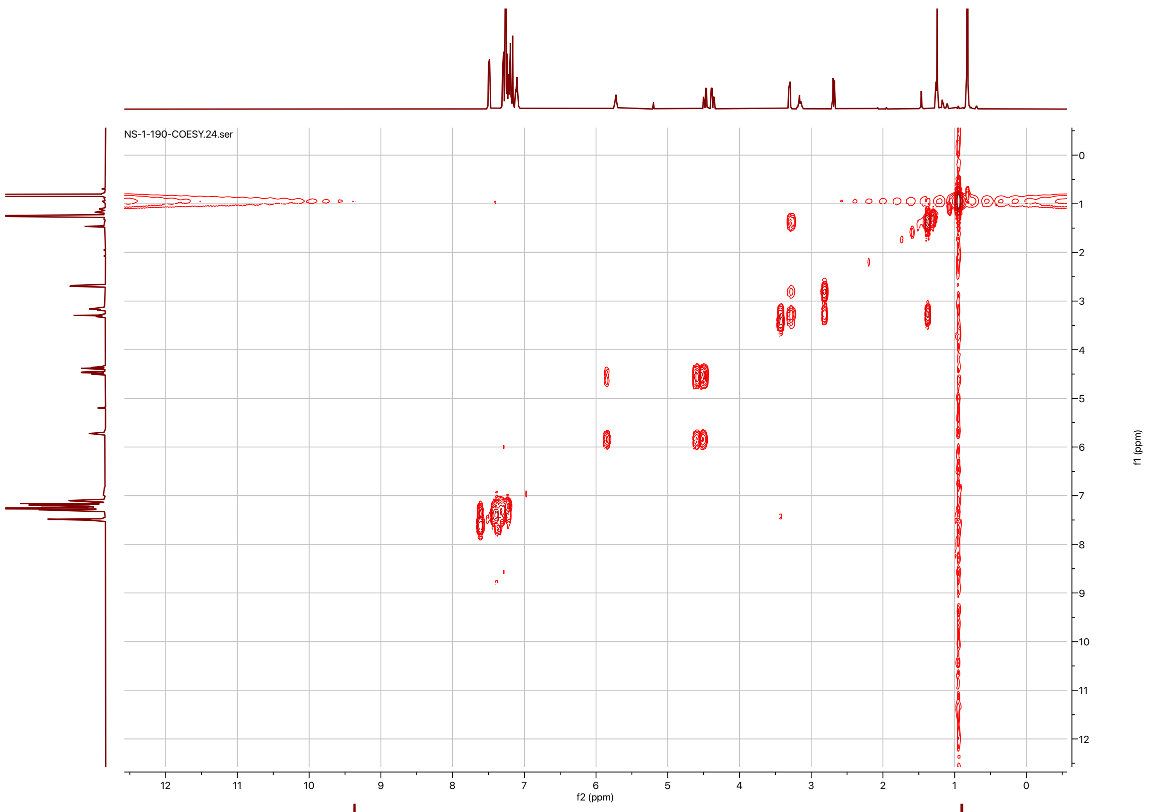

NS-1-190-COESY.24.ser
f2 (ppm)
f1 (ppm)

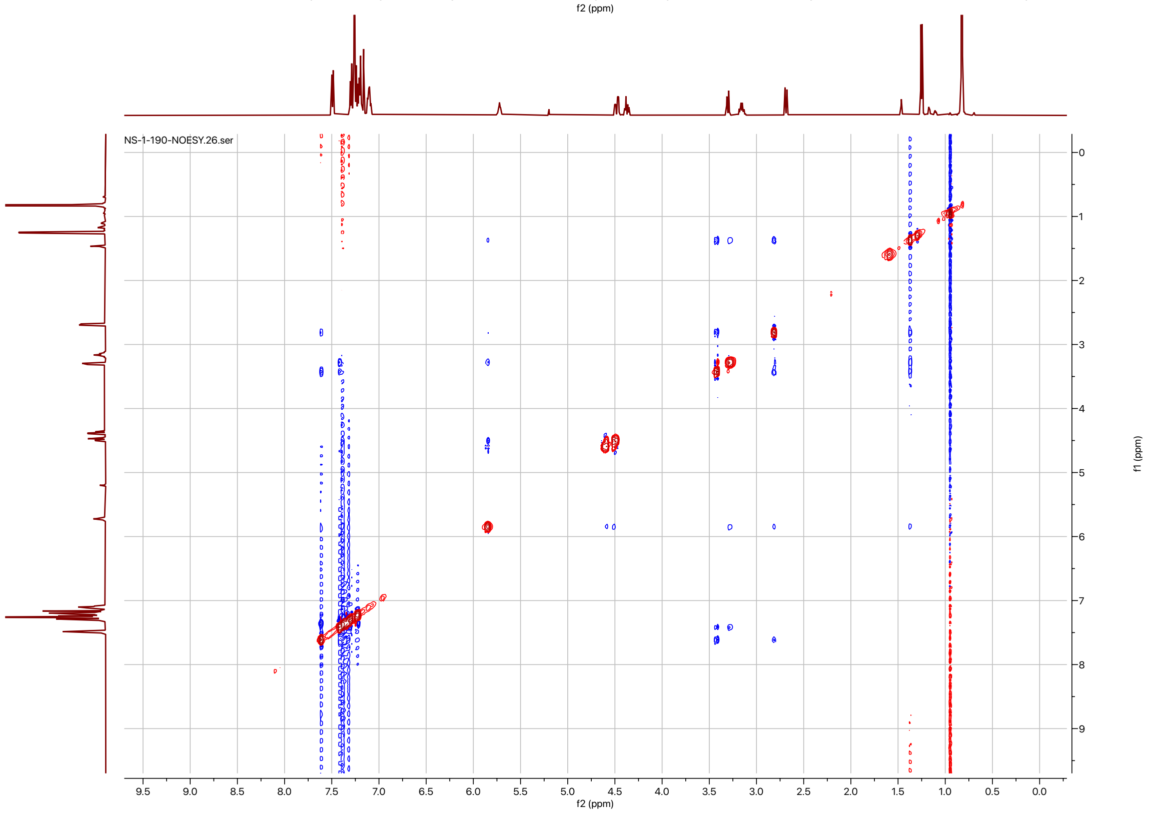

NS-1-190-NOESY.26.ser
f2 (ppm)
f1 (ppm)

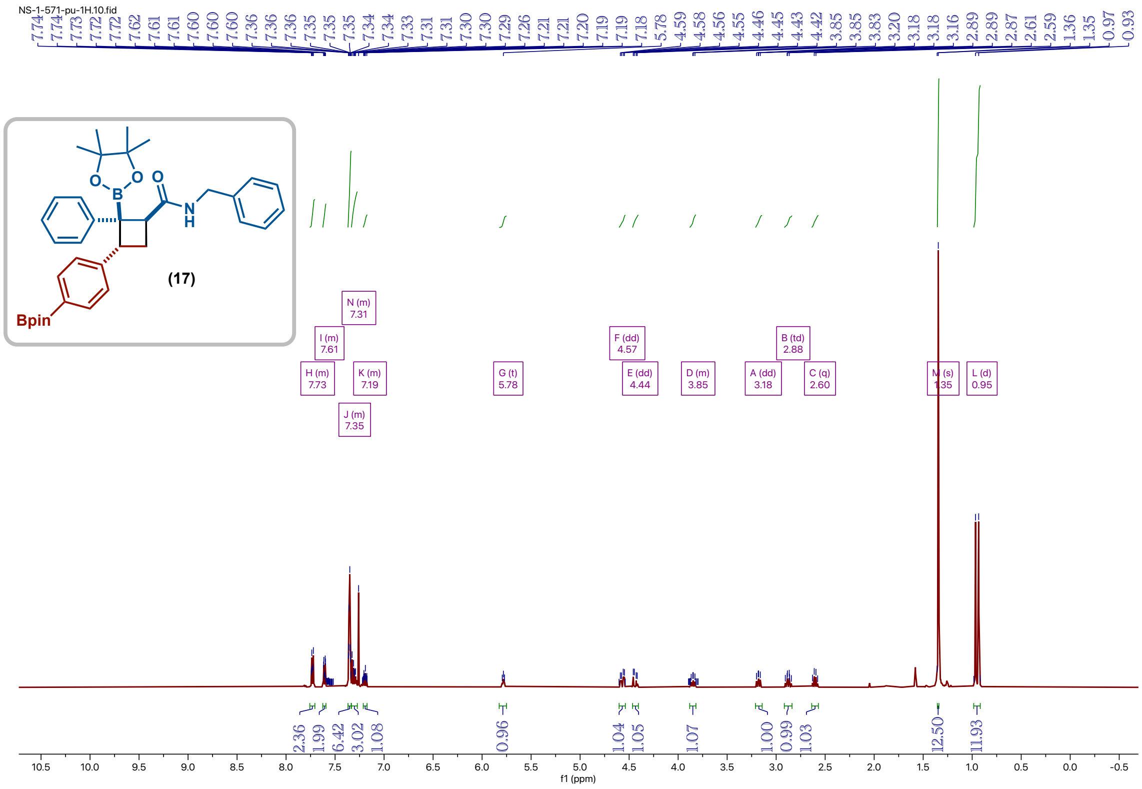
NS-1-571-pu-1H.10.fid
7.74 7.74 7.73 7.72 7.72 7.62 7.61 7.61 7.60 7.60 7.60 7.36 7.36 7.36 7.35 7.35 7.35 7.34 7.34 7.33 7.31 7.31 7.30 7.30 7.29 7.26 7.21 7.21 7.20 7.19 7.19 7.18 5.78 4.59 4.58 4.56 4.55 4.46 4.45 4.43 4.42 3.85 3.85 3.83 3.20 3.18 3.18 3.16 2.89 2.89 2.87 2.61 2.59 1.36 1.35 0.97 0.93
N (m) 7.31
I (m) 7.61
H (m) 7.73
K (m) 7.19
J (m) 7.35
G (t) 5.78
F (dd) 4.57
E (dd) 4.44
D (m) 3.85
A (dd) 3.18
B (td) 2.88
C (q) 2.60
M (s) 1.35
L (d) 0.95
2.36 1.99 6.42 3.02 1.08 0.96 1.04 1.05 1.07 1.00 0.99 1.03 12.50 11.93
f1 (ppm)
O
B
O
O
N
H
Bpin

(17)

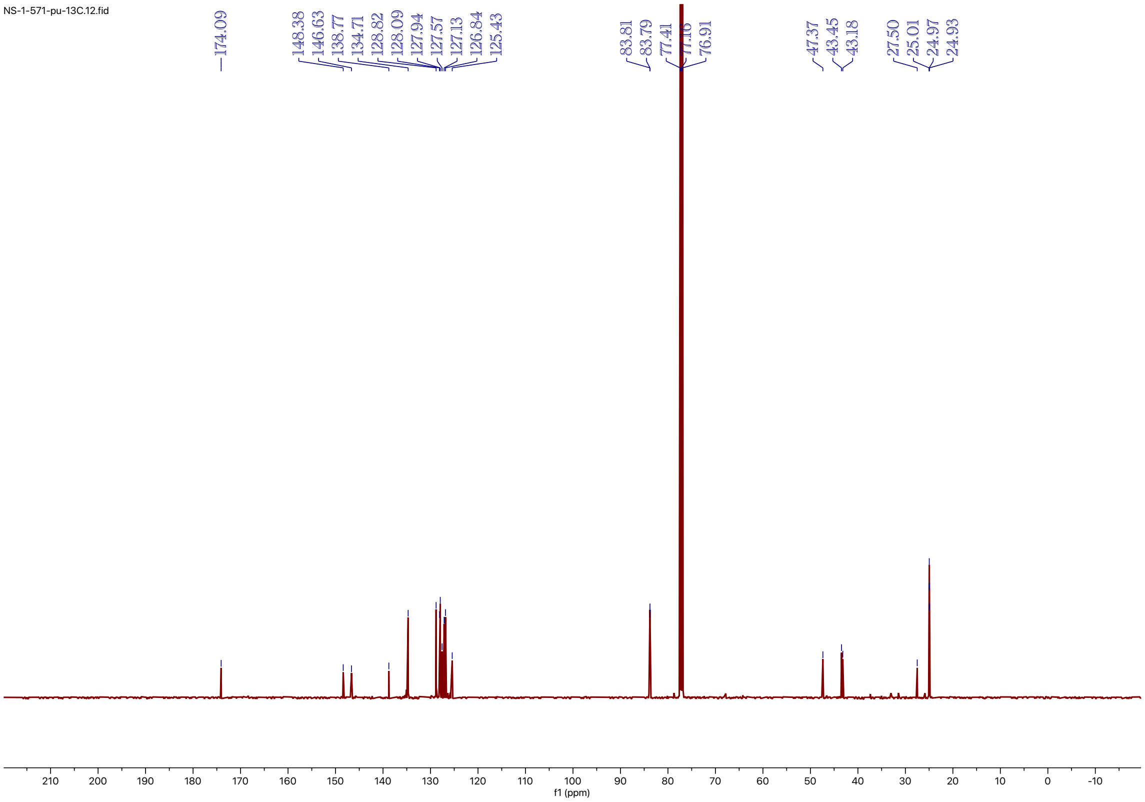
NS-1-571-pu-13C.12.fid
174.09
148.38 146.63 138.77 134.71 128.82 128.09 127.94 127.57 127.13 126.84 125.43
83.81 83.79 77.41 77.16 76.91
47.37 43.45 43.18
27.50 25.01 24.97 24.93
f1 (ppm)

NS-1-228-1H.20.fid

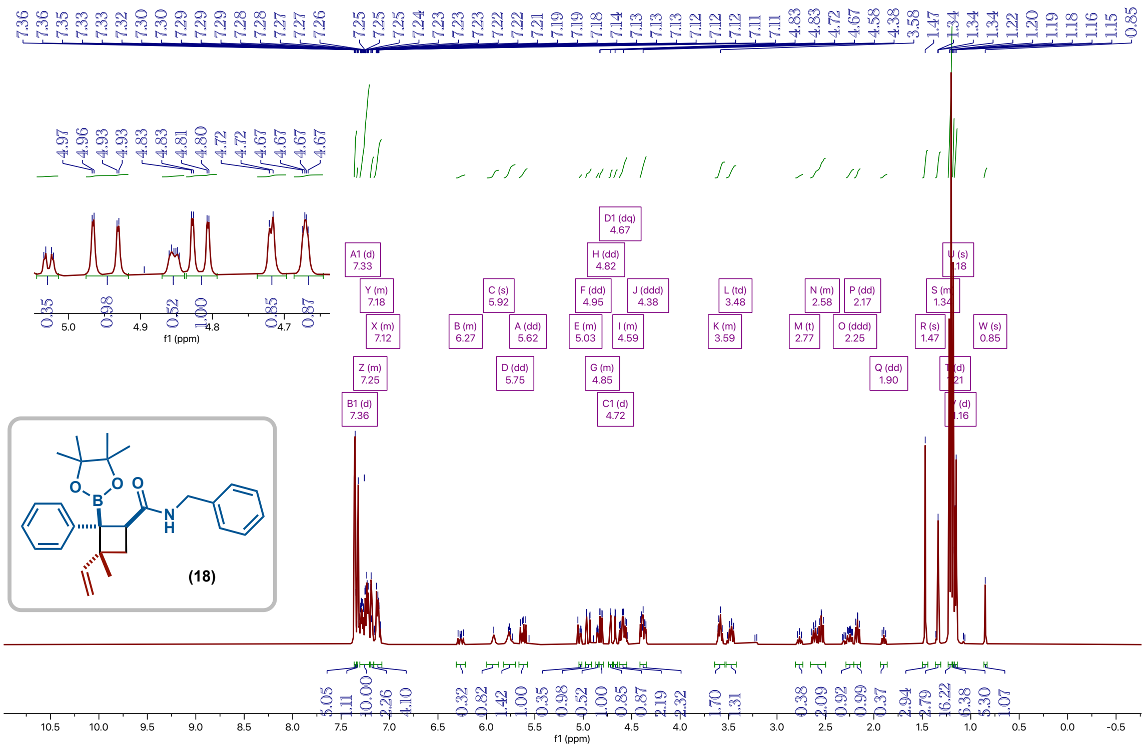

NS-1-228-13C.22.fid

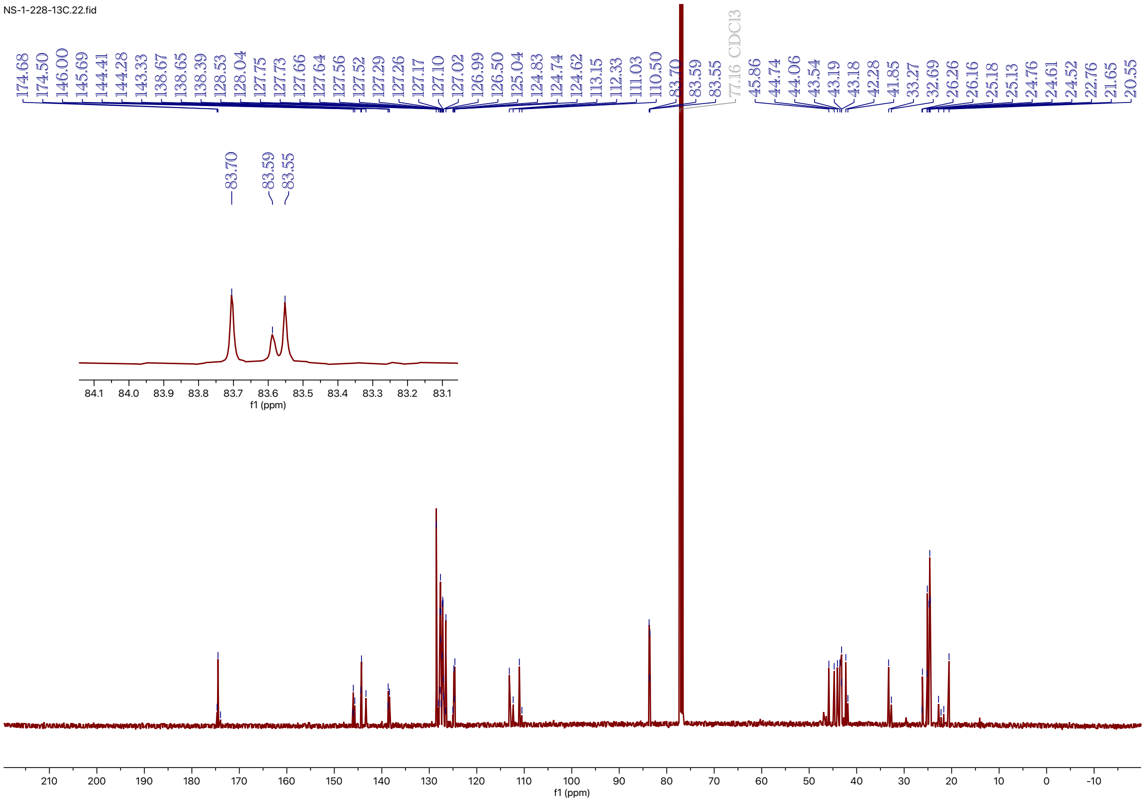

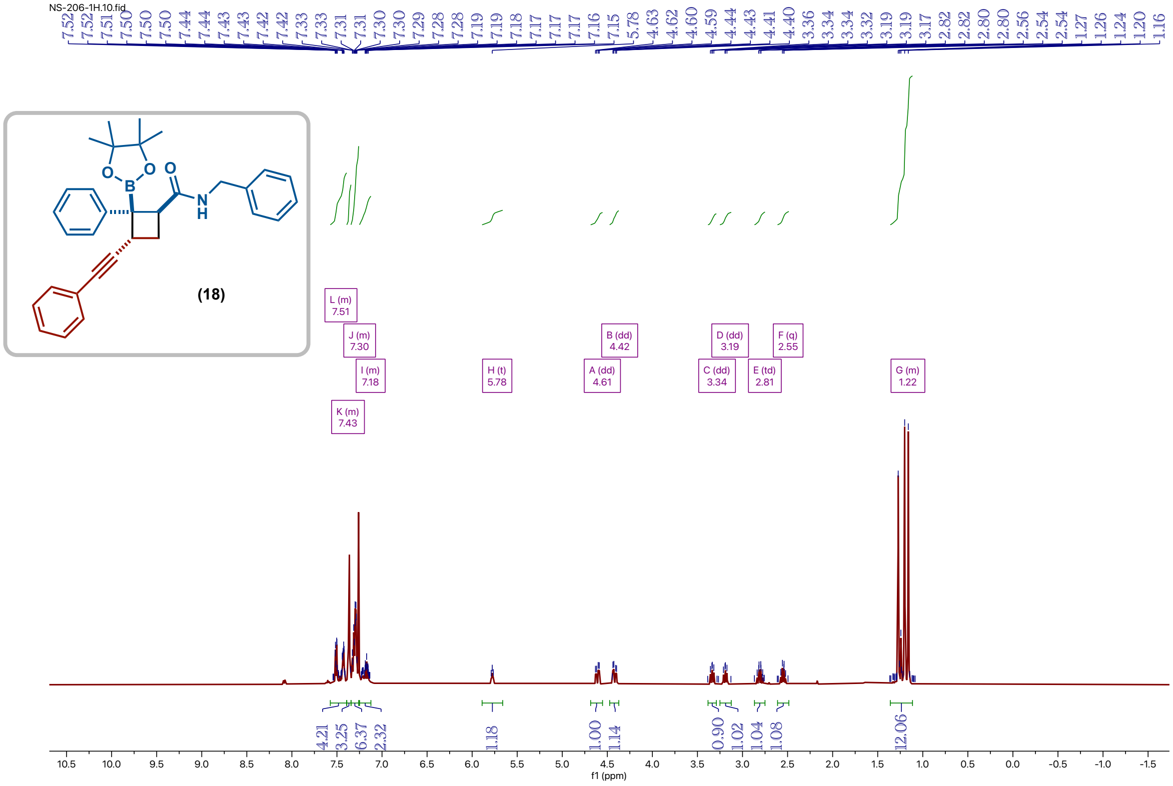
(18)
NS-206-1H.10.fid
7.52 7.52 7.51 7.50 7.50 7.50 7.44 7.44 7.43 7.43 7.42 7.42 7.33 7.33 7.31 7.31 7.30 7.30 7.29 7.28 7.28 7.19 7.19 7.18 7.17 7.17 7.17 7.16 7.15 5.78 4.63 4.62 4.60 4.59 4.44 4.43 4.41 4.40 3.36 3.34 3.34 3.32 3.19 3.19 3.17 2.82 2.82 2.80 2.80 2.56 2.54 2.54 1.27 1.26 1.24 1.20 1.16
L (m) 7.51
J (m) 7.30
I (m) 7.18
K (m) 7.43
H (t) 5.78
B (dd) 4.42
A (dd) 4.61
D (dd) 3.19
C (dd) 3.34
F (q) 2.55
E (td) 2.81
G (m) 1.22
4.21 3.25 6.37 2.32 1.18 1.00 1.14 0.90 1.02 1.04 1.08 12.06
f1 (ppm)

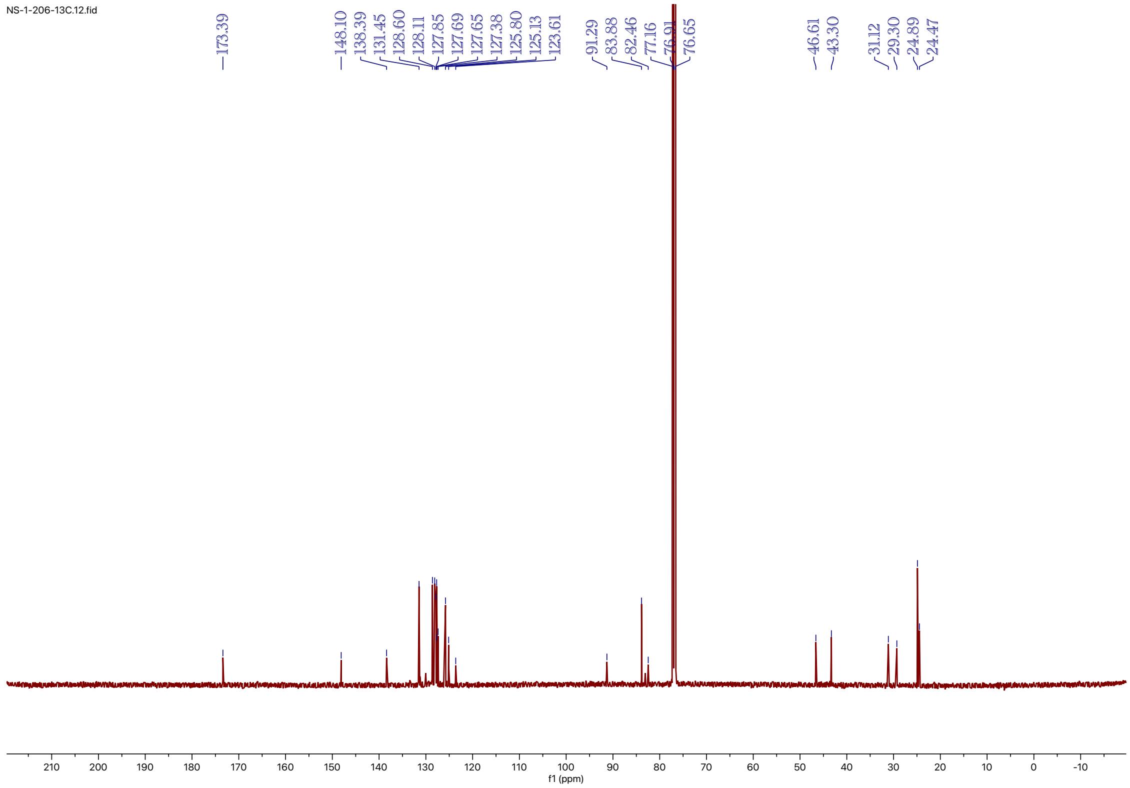
NS-1-206-13C.12.fid
173.39 148.10 138.39 131.45 128.60 128.11 127.85 127.69 127.65 127.38 125.80 125.13 123.61 91.29 83.88 82.46 77.16 76.91 76.65 46.61 43.30 31.12 29.30 24.89 24.47
f1 (ppm)

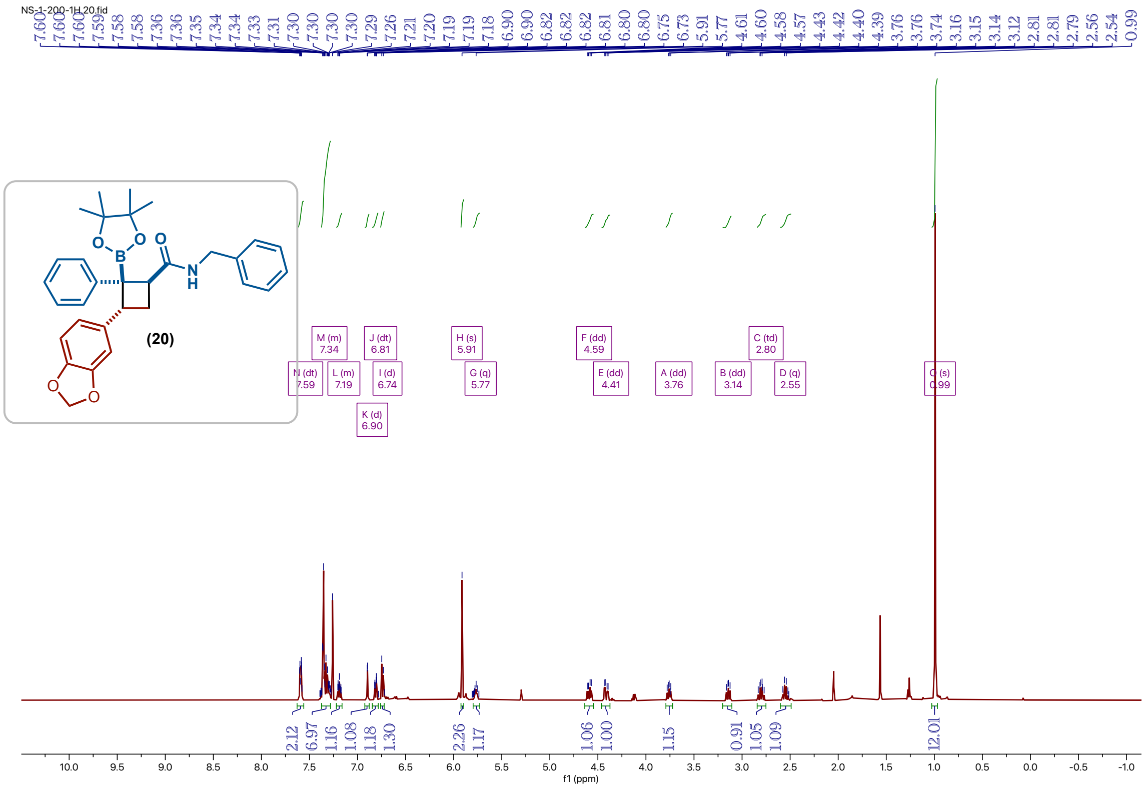
NS-1-200-1H.20.fid
7.60 7.60 7.60 7.59 7.58 7.36 7.36 7.35 7.34 7.34 7.33 7.31 7.30 7.30 7.30 7.30 7.29 7.26 7.21 7.20 7.19 7.19 7.18 6.90 6.90 6.82 6.82 6.82 6.81 6.80 6.80 6.75 6.73 5.91 5.77 4.61 4.60 4.58 4.57 4.43 4.42 4.40 4.39 3.76 3.74 3.16 3.15 3.14 3.12 2.81 2.81 2.79 2.56 2.54 0.99
(20)
M (m) 7.34
J (dt) 6.81
H (s) 5.91
F (dd) 4.59
C (td) 2.80
N (dt) 7.59
L (m) 7.19
I (d) 6.74
G (q) 5.77
E (dd) 4.41
A (dd) 3.76
B (dd) 3.14
D (q) 2.55
O (s) 0.99
K (d) 6.90
2.12 6.97 1.16 1.08 1.18 1.30 2.26 1.17 1.06 1.00 1.15 0.91 1.05 1.09 12.01
f1 (ppm)

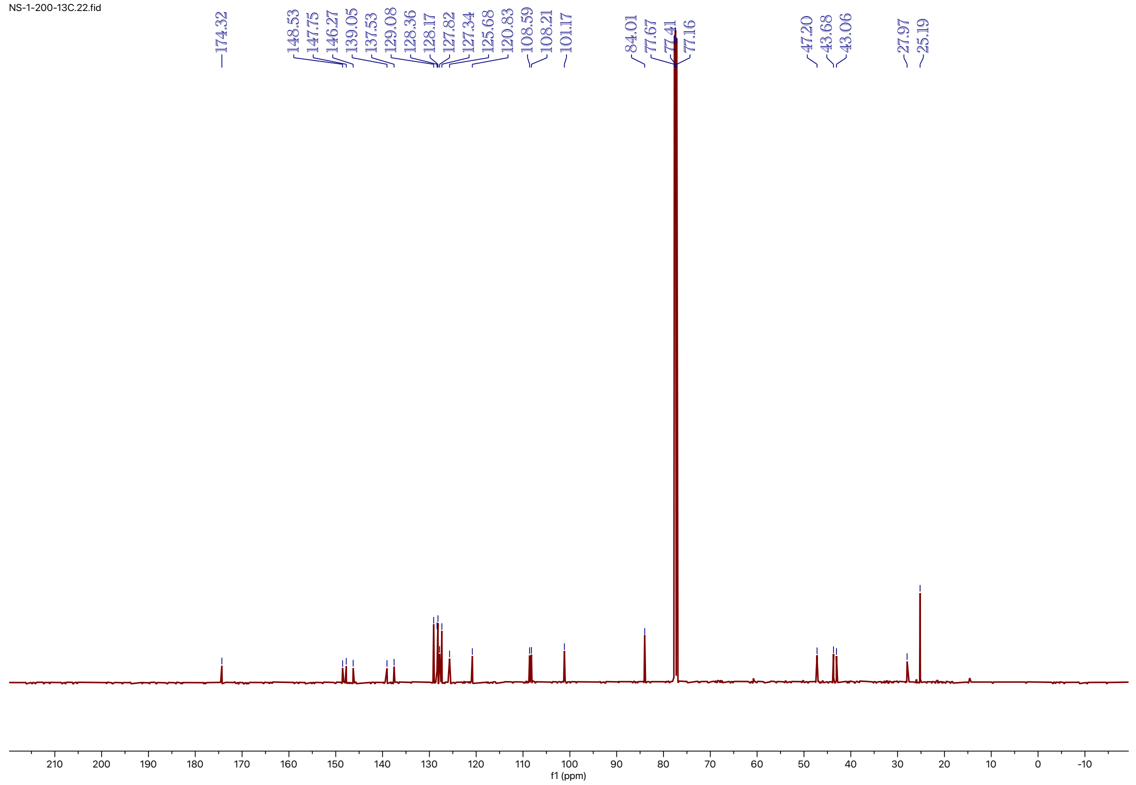
NS-1-200-13C.22.fid
174.32
148.53 147.75 146.27 139.05 137.53 129.08 128.36 128.17 127.82 127.34 125.68 120.83 108.59 108.21 101.17
84.01 77.67 77.41 77.16
47.20 43.68 43.06
27.97 25.19
f1 (ppm)

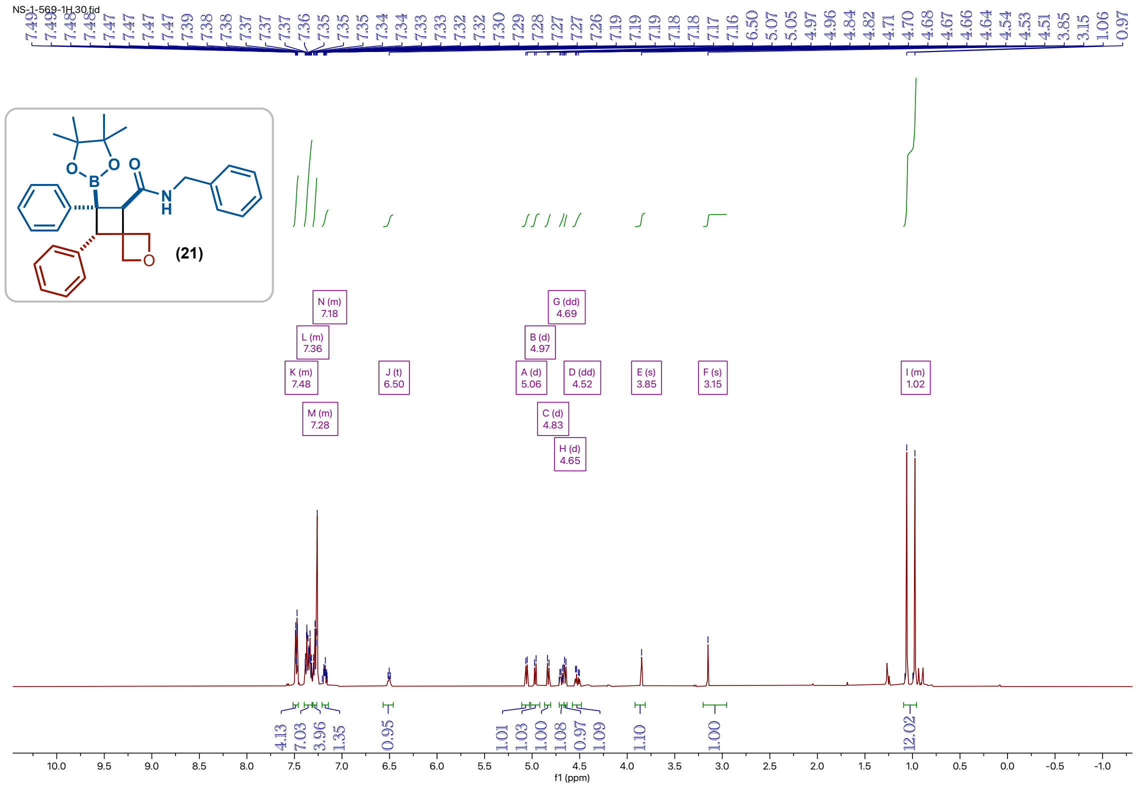
NS-1-569-1H.30.fid
7.49 7.49 7.48 7.48 7.47 7.47 7.47 7.47 7.39 7.38 7.38 7.37 7.37 7.37 7.36 7.35 7.35 7.35 7.34 7.34 7.33 7.33 7.33 7.32 7.32 7.30 7.29 7.28 7.27 7.27 7.26 7.19 7.19 7.19 7.18 7.18 7.17 7.16 6.50 5.07 5.05 4.97 4.96 4.84 4.82 4.71 4.70 4.68 4.67 4.66 4.64 4.54 4.53 4.51 3.85 3.15 1.06 0.97
(21)
O
B
N
H
N (m) 7.18
G (dd) 4.69
L (m) 7.36
B (d) 4.97
K (m) 7.48
J (t) 6.50
A (d) 5.06
D (dd) 4.52
E (s) 3.85
F (s) 3.15
I (m) 1.02
M (m) 7.28
C (d) 4.83
H (d) 4.65
4.13 7.03 3.96 1.35 0.95 1.01 1.03 1.00 1.08 0.97 1.09 1.10 1.00 12.02
10.0 9.5 9.0 8.5 8.0 7.5 7.0 6.5 6.0 5.5 5.0 4.5 4.0 3.5 3.0 2.5 2.0 1.5 1.0 0.5 0.0 -0.5 -1.0
f1 (ppm)

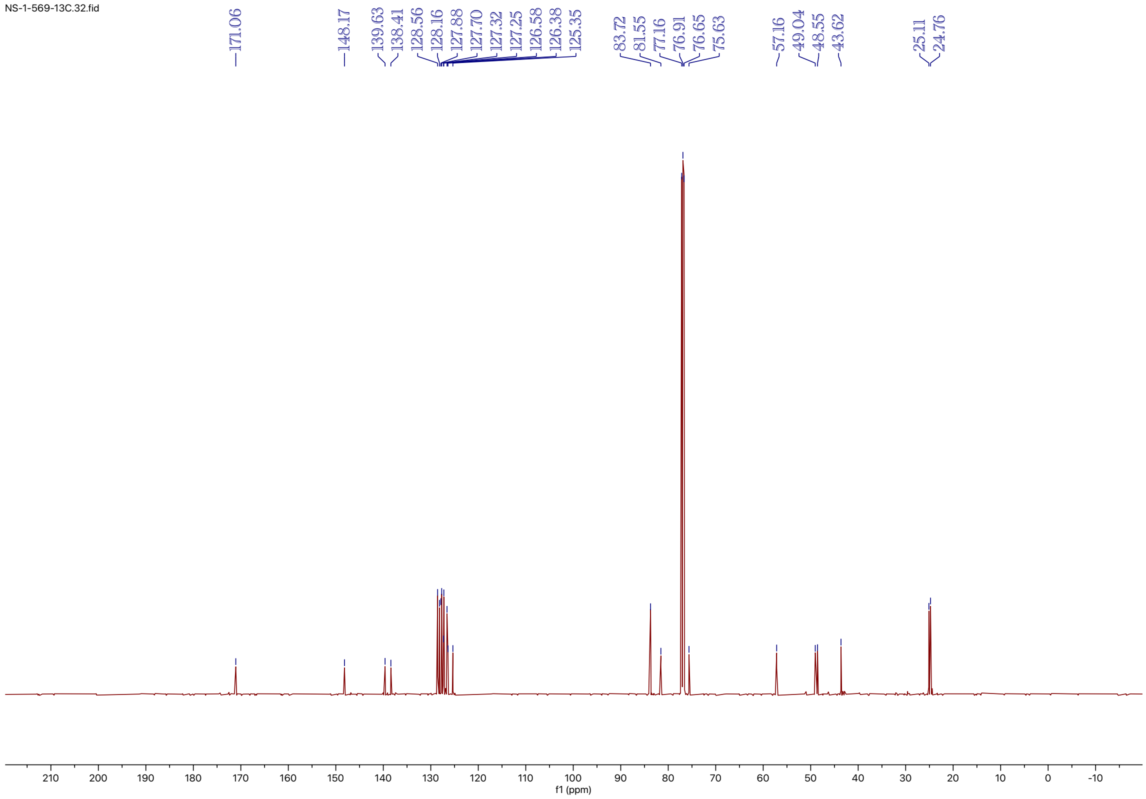
NS-1-569-13C.32.fid
171.06 148.17 139.63 138.41 128.56 128.16 127.88 127.70 127.32 127.25 126.58 126.38 125.35 83.72 81.55 77.16 76.91 76.65 75.63 57.16 49.04 48.55 43.62 25.11 24.76
210 200 190 180 170 160 150 140 130 120 110 100 90 80 70 60 50 40 30 20 10 0 -10
f1 (ppm)

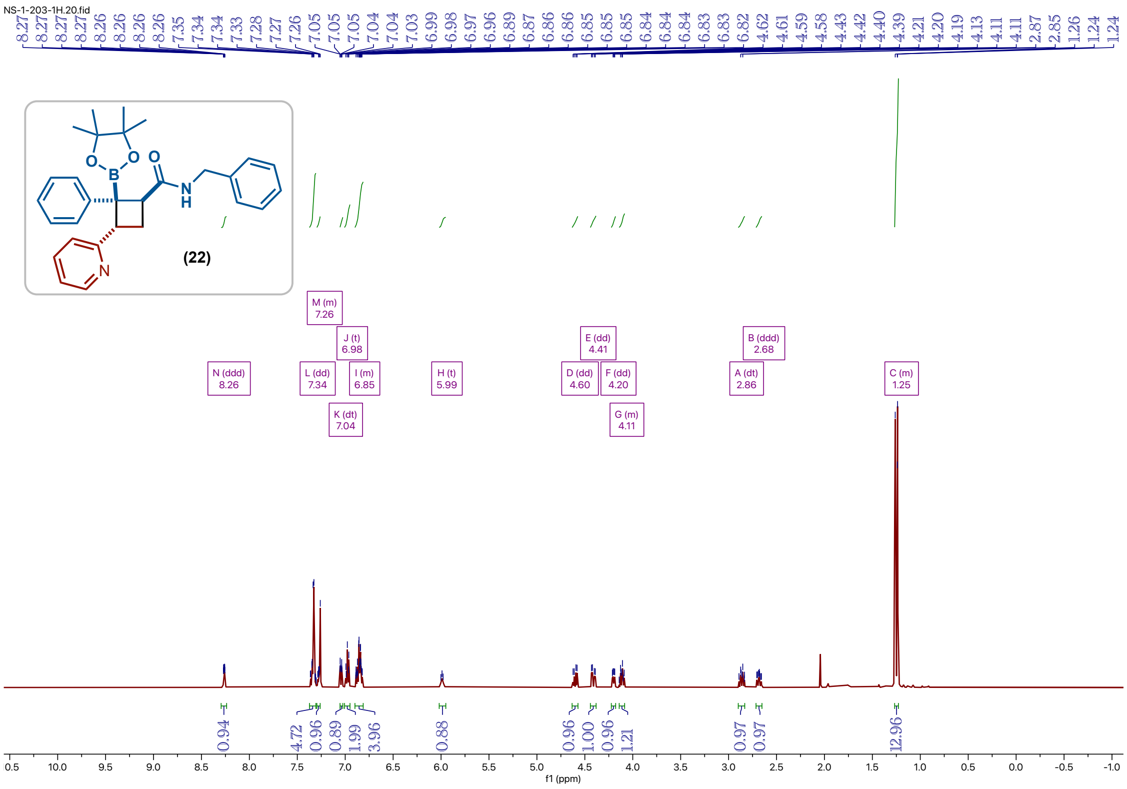
NS-1-203-1H.20.fid
8.27 8.27 8.27 8.27 8.26 8.26 8.26 8.26 7.35 7.34 7.34 7.33 7.28 7.27 7.26 7.05 7.05 7.05 7.04 7.04 7.03 6.99 6.98 6.97 6.96 6.89 6.87 6.86 6.86 6.85 6.85 6.85 6.84 6.84 6.84 6.83 6.83 6.82 4.62 4.61 4.59 4.58 4.43 4.42 4.40 4.39 4.21 4.20 4.19 4.13 4.11 4.11 2.87 2.85 1.26 1.24 1.24
(22)
M (m) 7.26
J (t) 6.98
N (ddd) 8.26
L (dd) 7.34
I (m) 6.85
K (dt) 7.04
H (t) 5.99
E (dd) 4.41
D (dd) 4.60
F (dd) 4.20
G (m) 4.11
B (ddd) 2.68
A (dt) 2.86
C (m) 1.25
0.94 4.72 0.96 0.89 1.99 3.96 0.88 0.96 1.00 0.96 1.21 0.97 0.97 12.96
f1 (ppm)

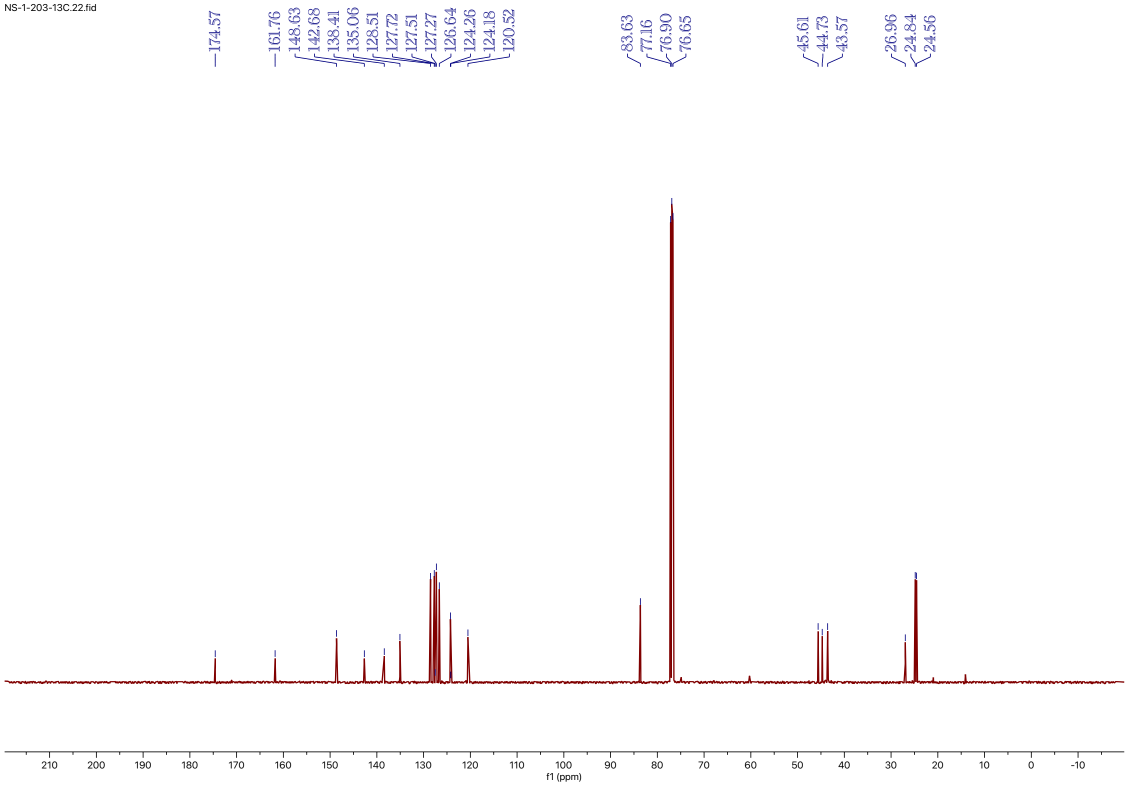
NS-1-203-13C.22.fid
174.57 161.76 148.63 142.68 138.41 135.06 128.51 127.72 127.51 127.27 126.64 124.26 124.18 120.52 83.63 77.16 76.90 76.65 45.61 44.73 43.57 26.96 24.84 24.56
f1 (ppm)

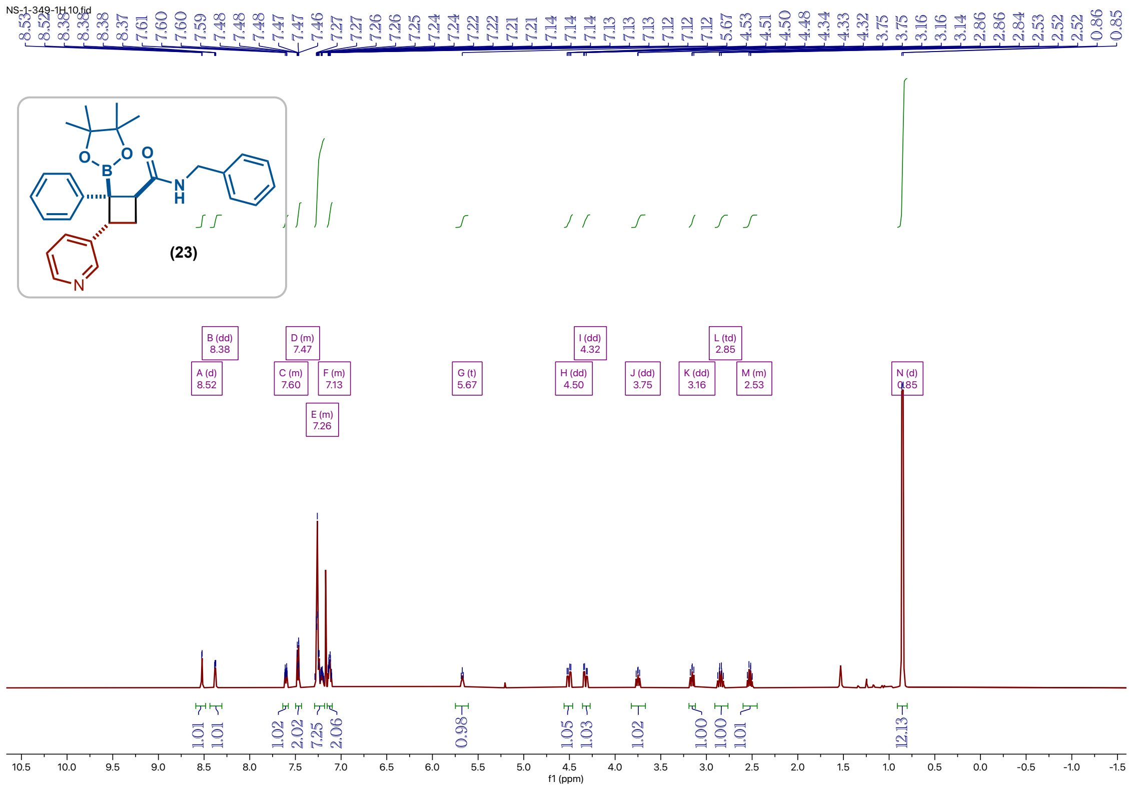
NS-1-349-1H.10.fid
8.53 8.52 8.38 8.38 8.38 8.37 7.61 7.60 7.60 7.59 7.48 7.48 7.48 7.47 7.47 7.46 7.27 7.27 7.26 7.26 7.25 7.24 7.24 7.22 7.22 7.21 7.21 7.14 7.14 7.14 7.13 7.13 7.13 7.12 7.12 7.12 5.67 4.53 4.51 4.50 4.48 4.34 4.33 4.32 3.75 3.75 3.16 3.16 3.14 2.86 2.86 2.84 2.53 2.52 2.52 0.86 0.85
(23)
A (d) 8.52
B (dd) 8.38
C (m) 7.60
D (m) 7.47
E (m) 7.26
F (m) 7.13
G (t) 5.67
H (dd) 4.50
I (dd) 4.32
J (dd) 3.75
K (dd) 3.16
L (td) 2.85
M (m) 2.53
N (d) 0.85
1.01 1.01 1.02 2.02 7.25 2.06 0.98 1.05 1.03 1.02 1.00 1.00 1.01 12.13
f1 (ppm)

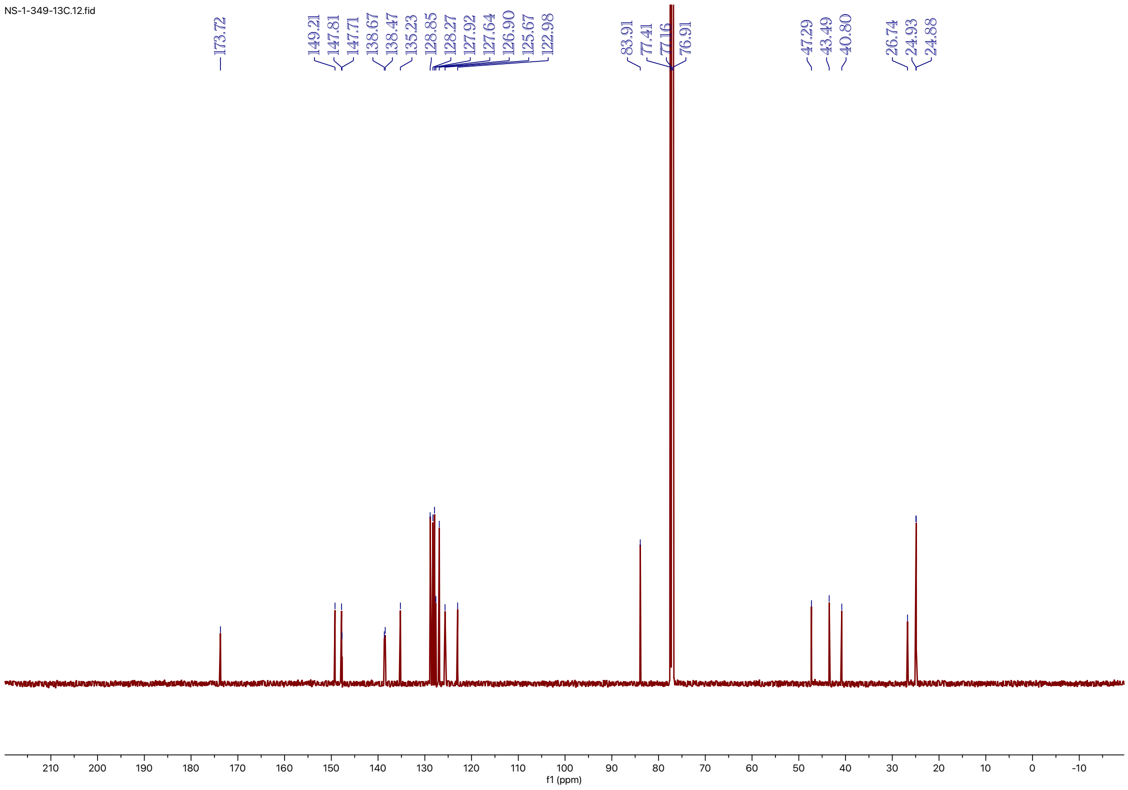
NS-1-349-13C.12.fid
173.72
149.21 147.81 147.71 138.67 138.47 135.23 128.85 128.27 127.92 127.64 126.90 125.67 122.98
83.91 77.41 77.16 76.91
47.29 43.49 40.80
26.74 24.93 24.88
f1 (ppm)

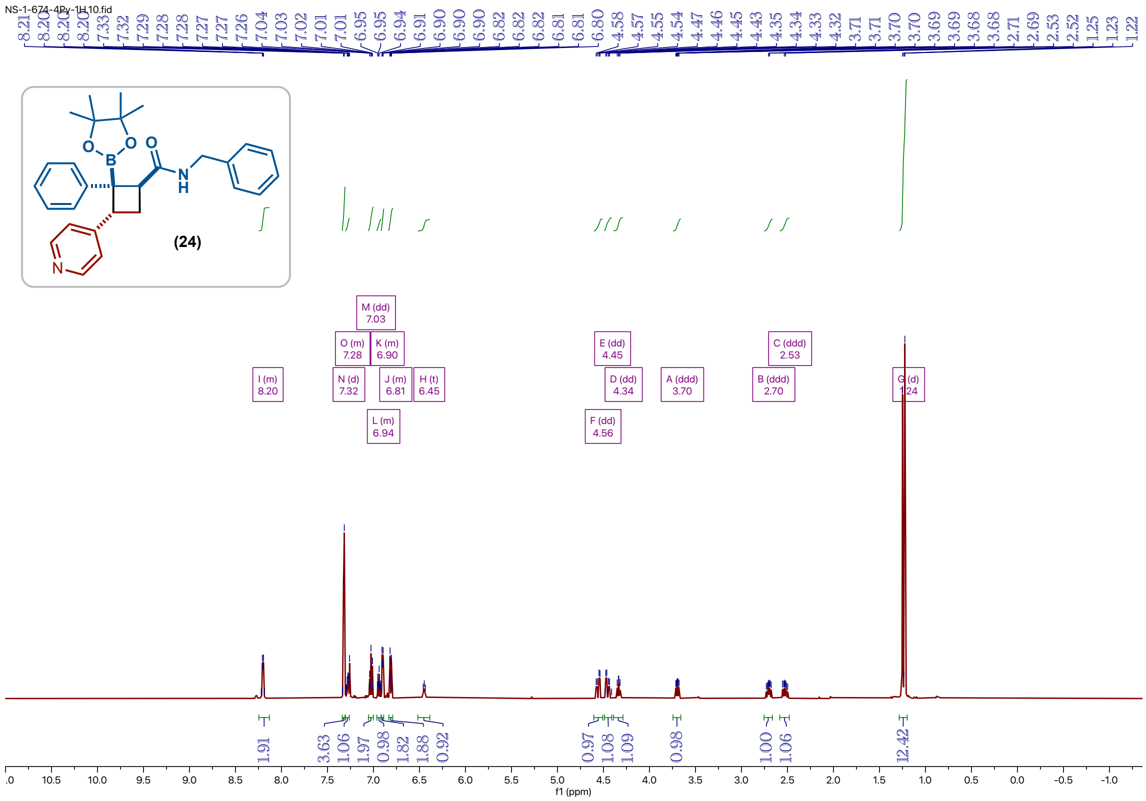
NS-1-674-4Py-1H10.fid
(24)
I (m) 8.20
O (m) 7.28
N (d) 7.32
M (dd) 7.03
K (m) 6.90
J (m) 6.81
L (m) 6.94
H (t) 6.45
E (dd) 4.45
D (dd) 4.34
F (dd) 4.56
A (ddd) 3.70
C (ddd) 2.53
B (ddd) 2.70
G (d) 1.24
f1 (ppm)

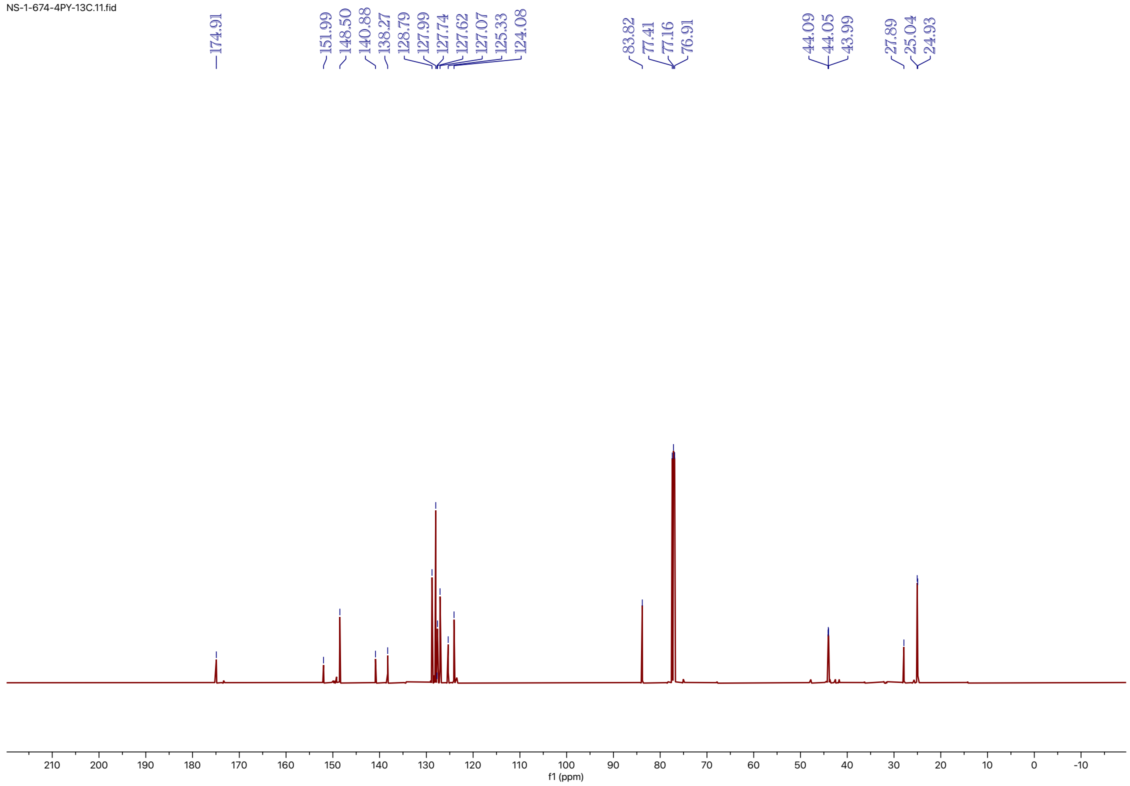
NS-1-674-4PY-13C.11.fid
174.91
151.99
148.50
140.88
138.27
128.79
127.99
127.74
127.62
127.07
125.33
124.08
83.82
77.41
77.16
76.91
44.09
44.05
43.99
27.89
25.04
24.93
f1 (ppm)

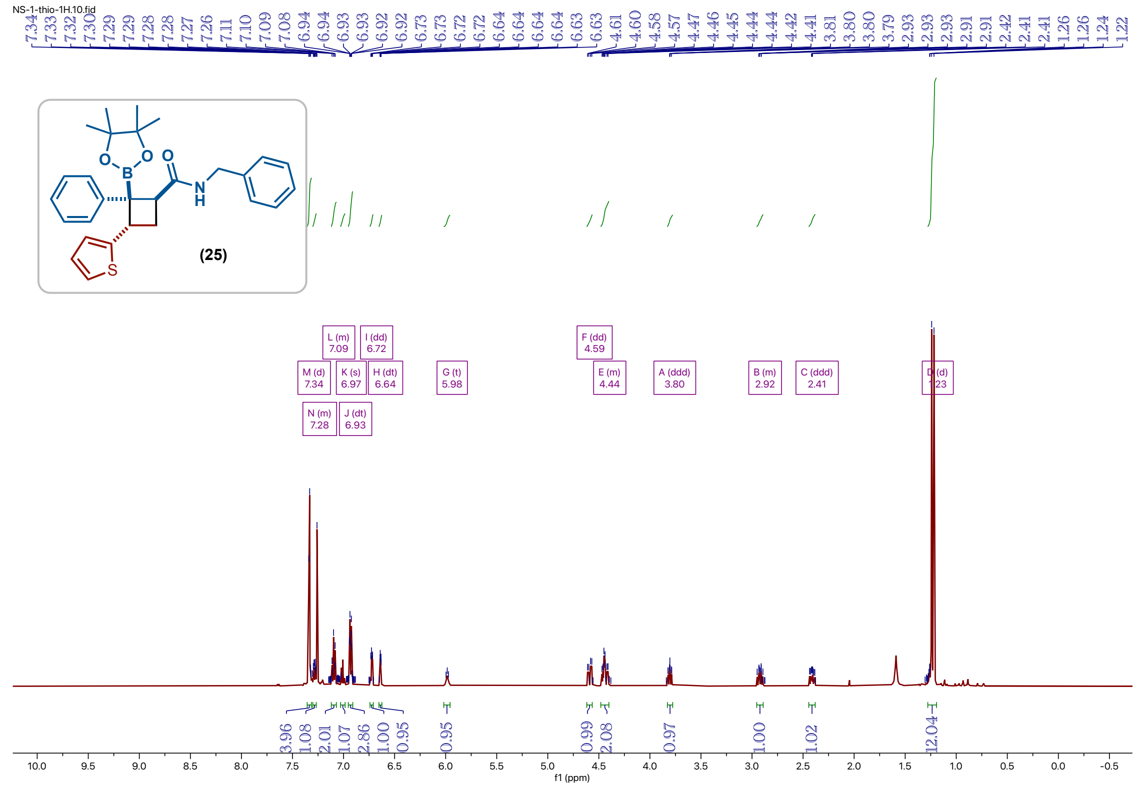
NS-1-thio-1H.10.fid
7.34 7.33 7.32 7.30 7.29 7.29 7.28 7.28 7.27 7.26 7.11 7.10 7.09 7.08 6.94 6.94 6.93 6.93 6.92 6.92 6.73 6.73 6.72 6.72 6.64 6.64 6.64 6.64 6.63 6.63 4.61 4.60 4.58 4.57 4.47 4.46 4.45 4.44 4.44 4.42 4.41 3.81 3.80 3.80 3.79 2.93 2.93 2.91 2.91 2.42 2.41 2.41 1.26 1.26 1.24 1.22
O
B
O
N
H
S
(25)
L (m) 7.09
I (dd) 6.72
F (dd) 4.59
M (d) 7.34
K (s) 6.97
H (dt) 6.64
G (t) 5.98
E (m) 4.44
A (ddd) 3.80
B (m) 2.92
C (ddd) 2.41
D (d) 1.23
N (m) 7.28
J (dt) 6.93
3.96 1.08 2.01 1.07 2.86 1.00 0.95 0.95 0.99 2.08 0.97 1.00 1.02 12.04
10.0 9.5 9.0 8.5 8.0 7.5 7.0 6.5 6.0 5.5 5.0 4.5 4.0 3.5 3.0 2.5 2.0 1.5 1.0 0.5 0.0 -0.5
f1 (ppm)

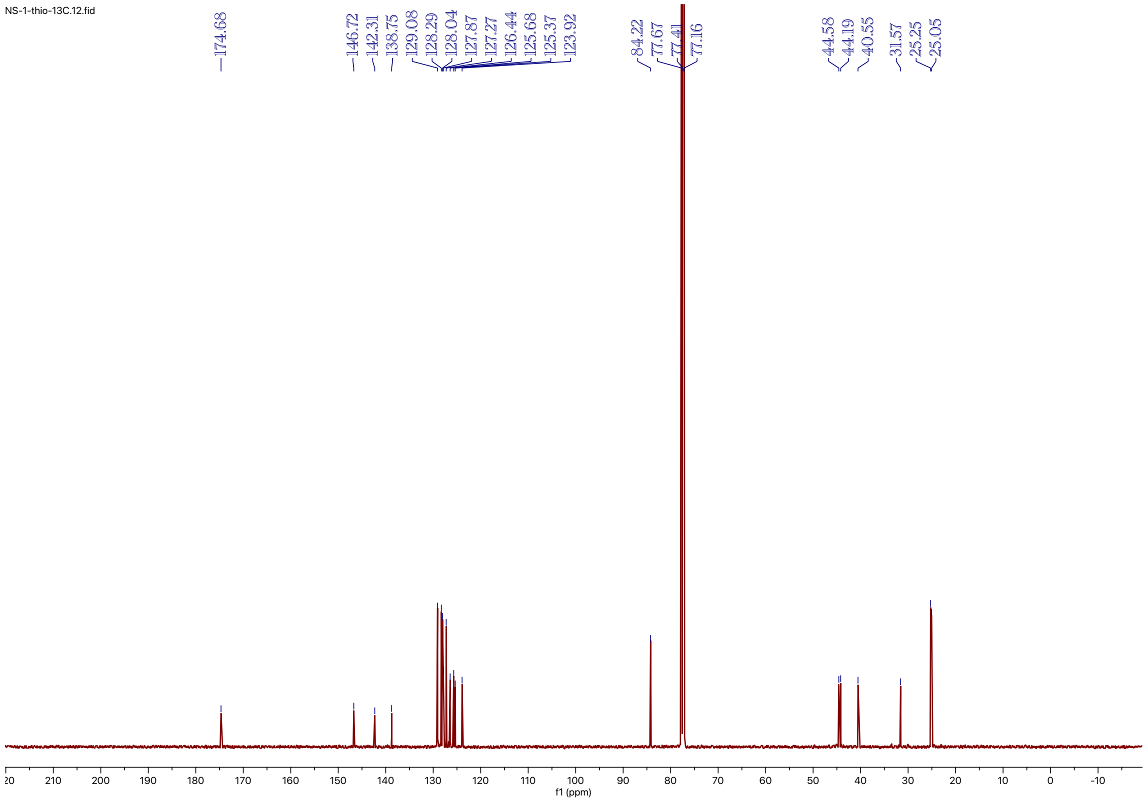
NS-1-thio-13C.12.fid
174.68
146.72 142.31 138.75 129.08 128.29 128.04 127.87 127.27 126.44 125.68 125.37 123.92
84.22 77.67 77.41 77.16
44.58 44.19 40.55 31.57 25.25 25.05
210 200 190 180 170 160 150 140 130 120 110 100 90 80 70 60 50 40 30 20 10 0 -10
f1 (ppm)

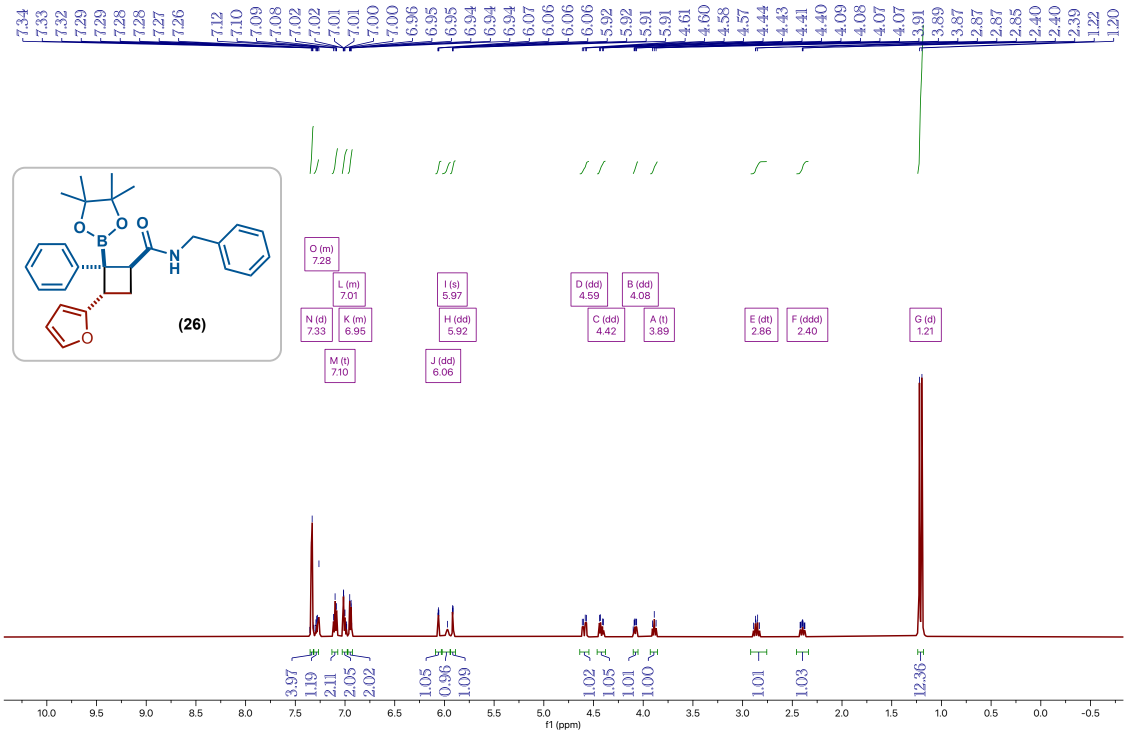
NS-1-278-pu.10.fid
7.34 7.33 7.32 7.29 7.29 7.28 7.28 7.27 7.26 7.12 7.10 7.09 7.08 7.02 7.02 7.01 7.01 7.00 7.00 6.96 6.95 6.95 6.94 6.94 6.94 6.07 6.06 6.06 6.06 5.92 5.92 5.91 5.91 4.61 4.60 4.58 4.57 4.44 4.43 4.41 4.40 4.09 4.08 4.07 4.07 3.91 3.89 3.87 2.87 2.87 2.85 2.40 2.40 2.39 1.22 1.20
(26)
O (m) 7.28
L (m) 7.01
N (d) 7.33
K (m) 6.95
M (t) 7.10
I (s) 5.97
H (dd) 5.92
J (dd) 6.06
D (dd) 4.59
C (dd) 4.42
B (dd) 4.08
A (t) 3.89
E (dt) 2.86
F (ddd) 2.40
G (d) 1.21
3.97 1.19 2.11 2.05 2.02 1.05 0.96 1.09 1.02 1.05 1.01 1.00 1.01 1.03 12.36
f1 (ppm)

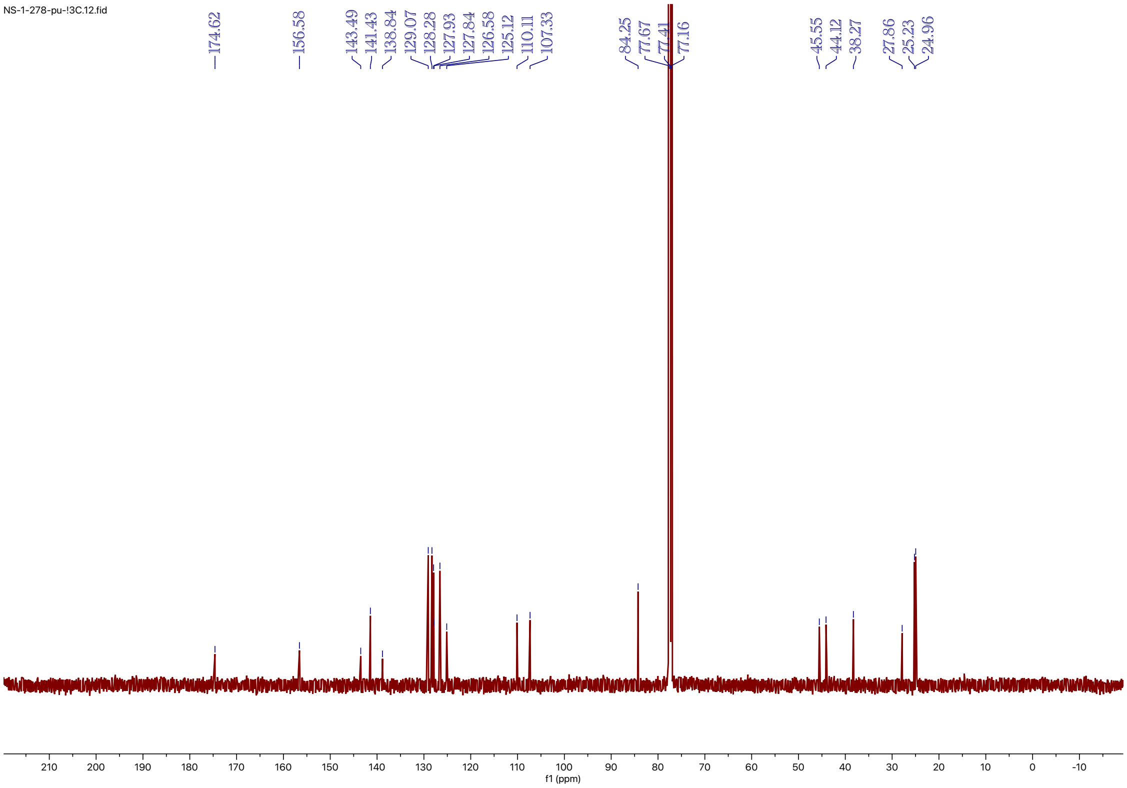
NS-1-278-pu-!3C.12.fid
174.62 156.58 143.49 141.43 138.84 129.07 128.28 127.93 127.84 126.58 125.12 110.11 107.33 84.25 77.67 77.41 77.16 45.55 44.12 38.27 27.86 25.23 24.96
f1 (ppm)

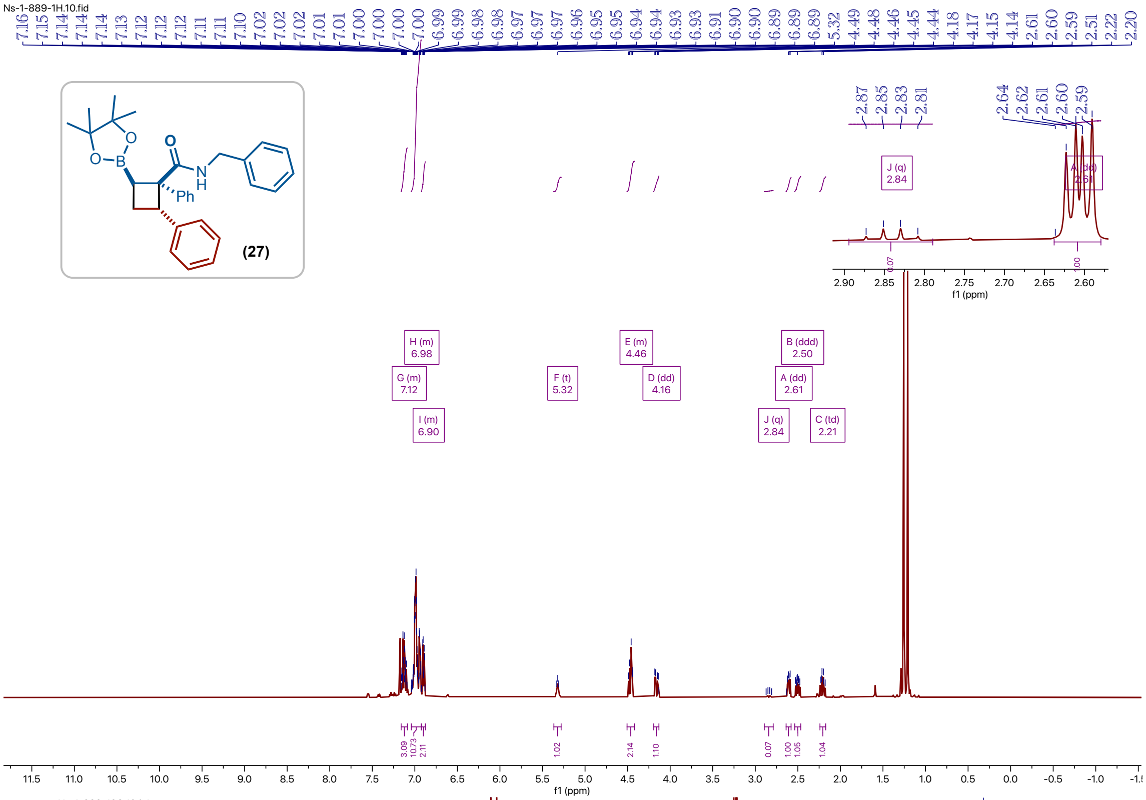
Ns-1-889-1H.10.fid
7.16 7.15 7.14 7.14 7.14 7.13 7.12 7.12 7.12 7.11 7.11 7.10 7.02 7.02 7.02 7.01 7.01 7.00 7.00 7.00 7.00 6.99 6.99 6.98 6.98 6.97 6.97 6.96 6.95 6.95 6.94 6.94 6.93 6.93 6.91 6.90 6.90 6.89 6.89 6.89 5.32 4.49 4.48 4.46 4.45 4.44 4.18 4.17 4.15 4.14 2.61 2.60 2.59 2.51 2.22 2.20
Ph
(27)
2.87 2.85 2.83 2.81
2.64 2.62 2.61 2.60 2.59
J (q) 2.84
A (dd) 2.61
f1 (ppm)
H (m) 6.98
G (m) 7.12
I (m) 6.90
F (t) 5.32
E (m) 4.46
D (dd) 4.16
B (ddd) 2.50
A (dd) 2.61
J (q) 2.84
C (td) 2.21
3.09 10.73 2.11 1.02 2.14 1.10 0.07 1.00 1.05 1.04
f1 (ppm)

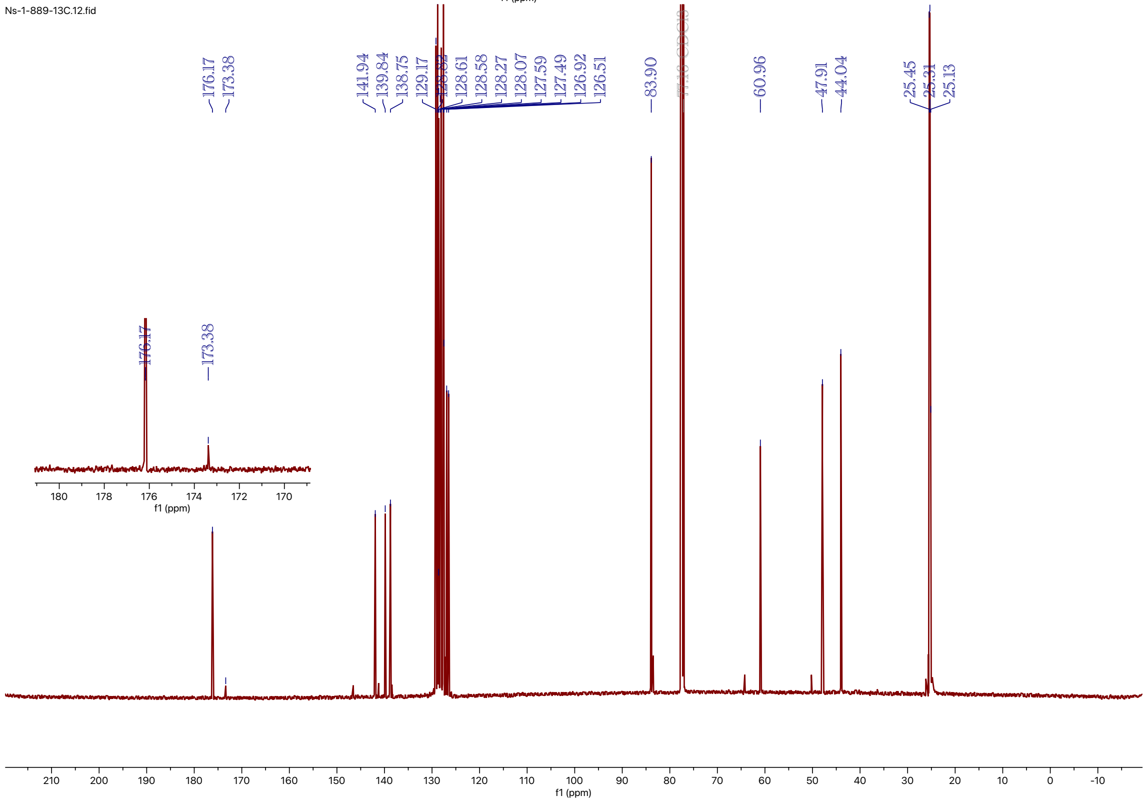
Ns-1-889-13C.12.fid
176.17 173.38
141.94 139.84 138.75 129.17 128.61 128.58 128.27 128.07 127.59 127.49 126.92 126.51
83.90
60.96
47.91 44.04
25.45 25.13
176.17 173.38
f1 (ppm)
f1 (ppm)

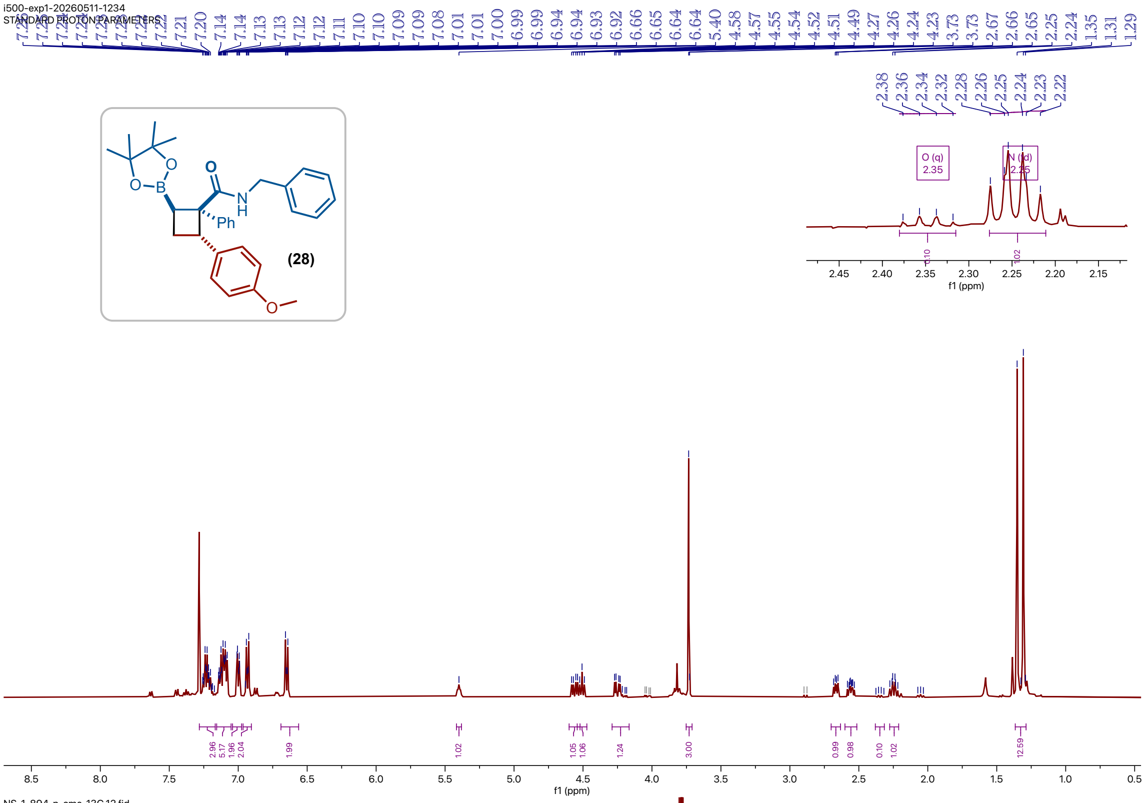
i500-exp1-20260511-1234
STANDARD PROTON PARAMETERS
7.21 7.20 7.14 7.14 7.13 7.13 7.12 7.11 7.10 7.10 7.09 7.09 7.08 7.01 7.01 7.00 6.99 6.99 6.94 6.94 6.93 6.92 6.66 6.65 6.64 6.64 5.40 4.58 4.57 4.55 4.54 4.52 4.51 4.49 4.27 4.26 4.24 4.23 3.73 3.73 2.67 2.66 2.65 2.25 2.24 1.35 1.31 1.29
2.38 2.36 2.34 2.32 2.28 2.26 2.25 2.24 2.23 2.22
O (q)
2.35
N (td)
2.25
0.10
1.02
f1 (ppm)
Ph
(28)
2.96 5.17 1.96 2.04 1.99 1.02 1.05 1.06 1.24 3.00 0.99 0.98 0.10 1.02 12.59
f1 (ppm)

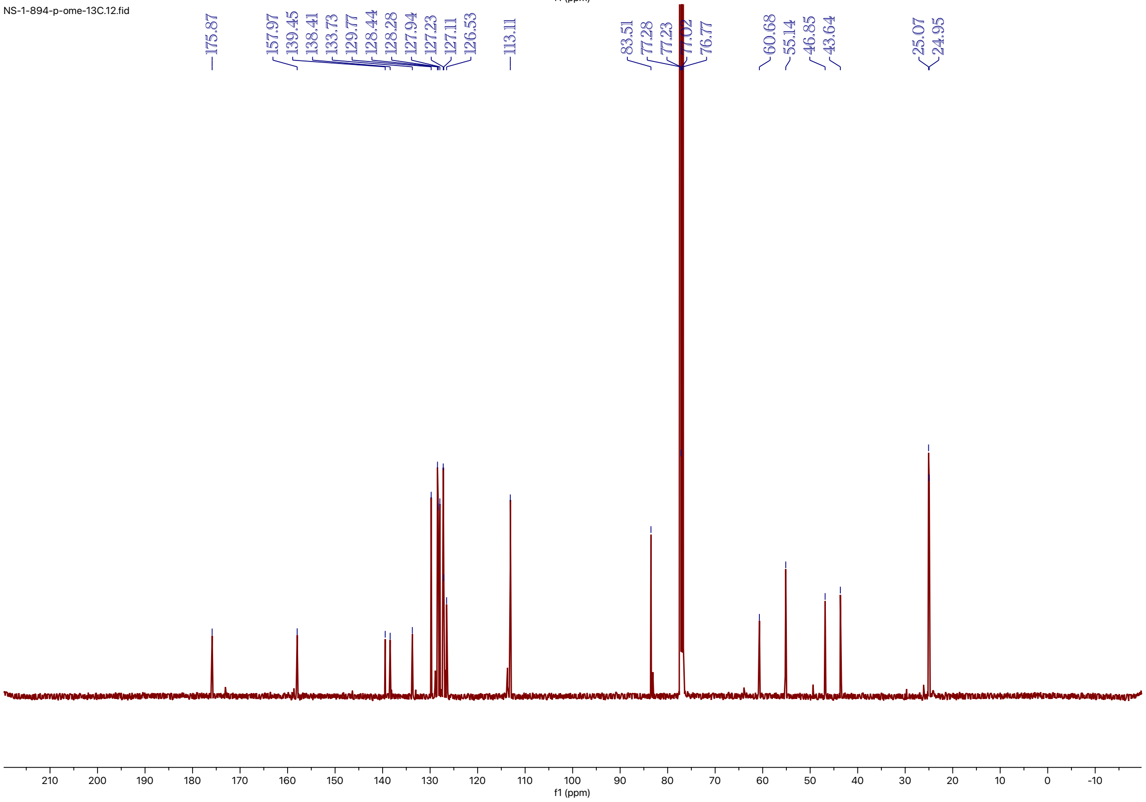
NS-1-894-p-ome-13C.12.fid
175.87
157.97 139.45 138.41 133.73 129.77 128.44 128.28 127.94 127.23 127.11 126.53
113.11
83.51 77.28 77.23 77.02 76.77
60.68 55.14 46.85 43.64
25.07 24.95
f1 (ppm)

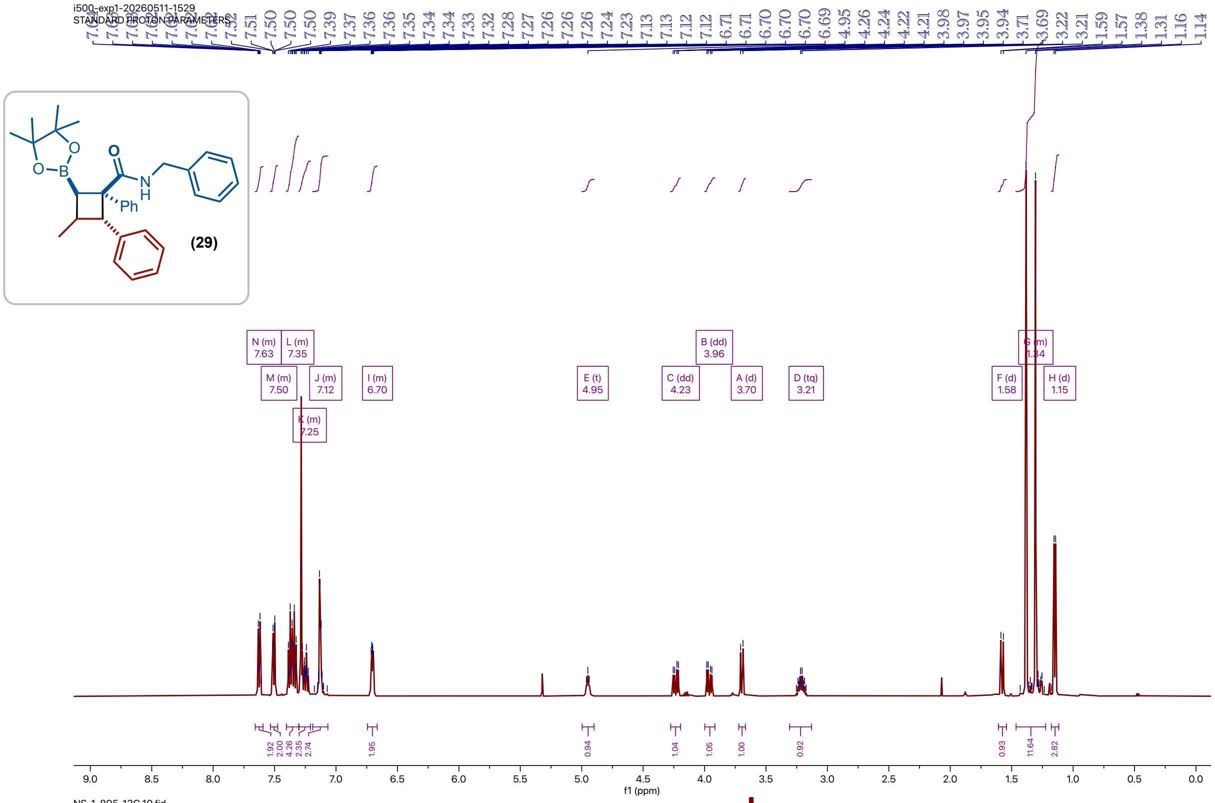

i500-exp1-20260511-1529
STANDARD PROTON PARAMETERS
7.51 7.50 7.50 7.50 7.39 7.37 7.36 7.35 7.34 7.34 7.33 7.32 7.28 7.27 7.26 7.26 7.26 7.24 7.23 7.13 7.13 7.12 7.12 6.71 6.71 6.70 6.70 6.70 6.69 4.95 4.26 4.24 4.22 4.21 3.98 3.97 3.95 3.94 3.71 3.69 3.22 3.21 1.59 1.57 1.38 1.31 1.16 1.14
O
O
B
O
N
H
Ph
(29)
N (m) 7.63
L (m) 7.35
M (m) 7.50
J (m) 7.12
K (m) 7.25
I (m) 6.70
E (t) 4.95
C (dd) 4.23
B (dd) 3.96
A (d) 3.70
D (tq) 3.21
F (d) 1.58
G (m) 1.34
H (d) 1.15
1.92 2.00 4.26 2.15 2.74 1.95 0.94 1.04 1.05 1.00 0.92 0.93 11.64 2.82
f1 (ppm)

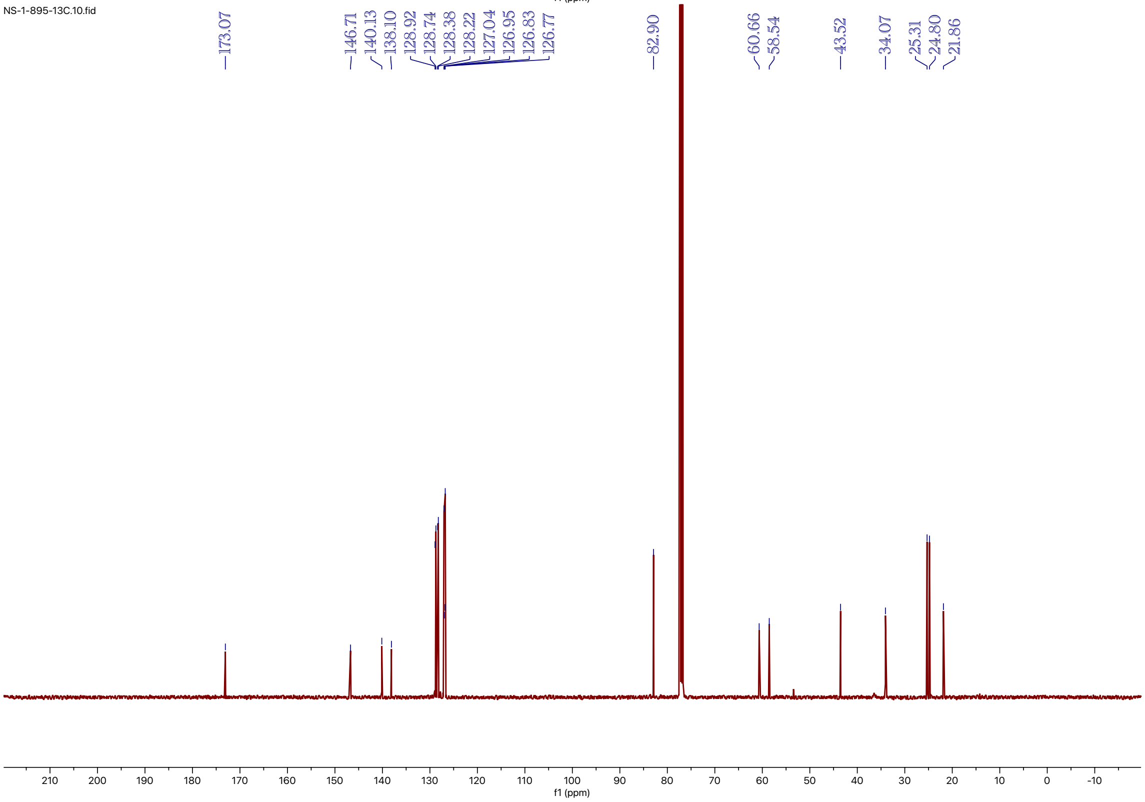

NS-1-895-13C.10.fid
173.07 146.71 140.13 138.10 128.92 128.74 128.38 128.22 127.04 126.95 126.83 126.77 82.90 60.66 58.54 43.52 34.07 25.31 24.80 21.86
f1 (ppm)

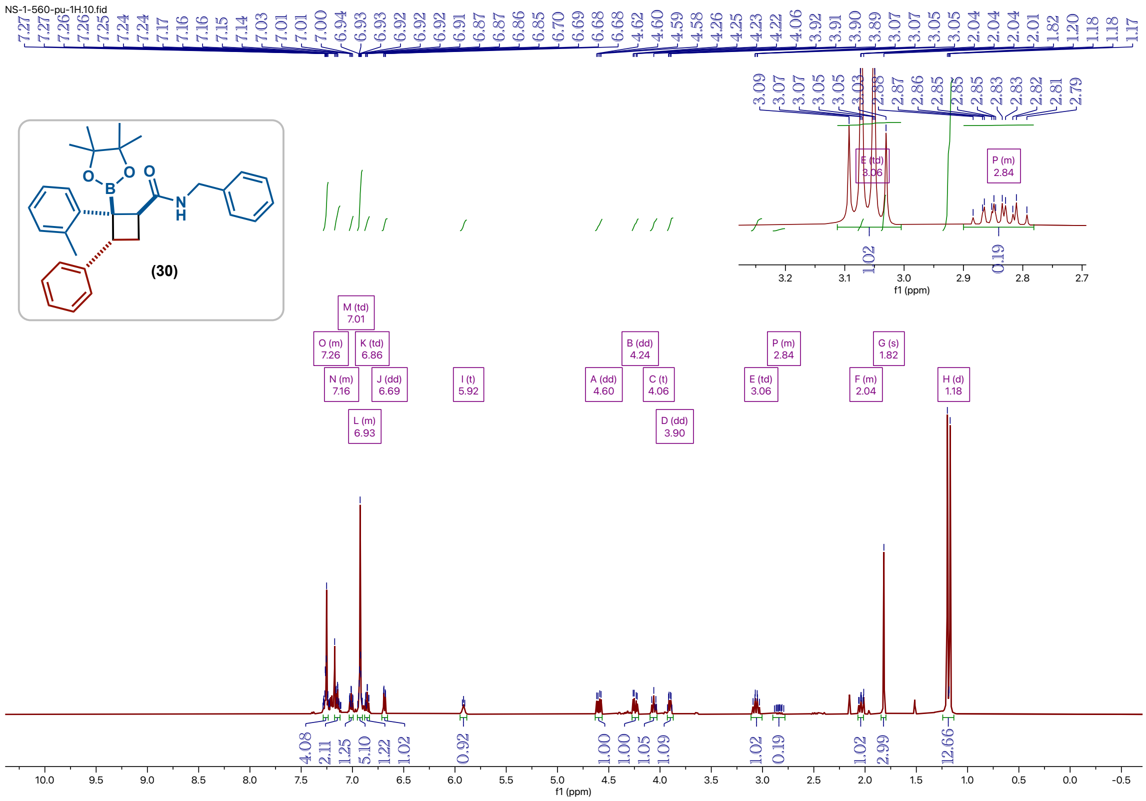
NS-1-560-pu-1H.10.fid
7.27 7.27 7.26 7.26 7.25 7.24 7.24 7.17 7.16 7.16 7.15 7.14 7.03 7.01 7.01 7.00 6.94 6.93 6.93 6.92 6.92 6.92 6.91 6.87 6.87 6.86 6.85 6.70 6.69 6.68 6.68 4.62 4.60 4.59 4.58 4.26 4.25 4.23 4.22 4.06 3.92 3.91 3.90 3.89 3.07 3.07 3.05 3.05 2.04 2.04 2.04 2.01 1.82 1.20 1.18 1.18 1.17
(30)
3.09 3.07 3.07 3.05 3.05 3.03 2.88 2.87 2.86 2.85 2.85 2.85 2.83 2.83 2.82 2.81 2.79
E (td) 3.06
P (m) 2.84
1.02
0.19
f1 (ppm)
M (td) 7.01
O (m) 7.26
K (td) 6.86
N (m) 7.16
J (dd) 6.69
L (m) 6.93
I (t) 5.92
A (dd) 4.60
B (dd) 4.24
C (t) 4.06
D (dd) 3.90
E (td) 3.06
P (m) 2.84
F (m) 2.04
G (s) 1.82
H (d) 1.18
4.08 2.11 1.25 5.10 1.22 1.02 0.92 1.00 1.05 1.09 1.02 0.19 1.02 2.99 12.66
f1 (ppm)

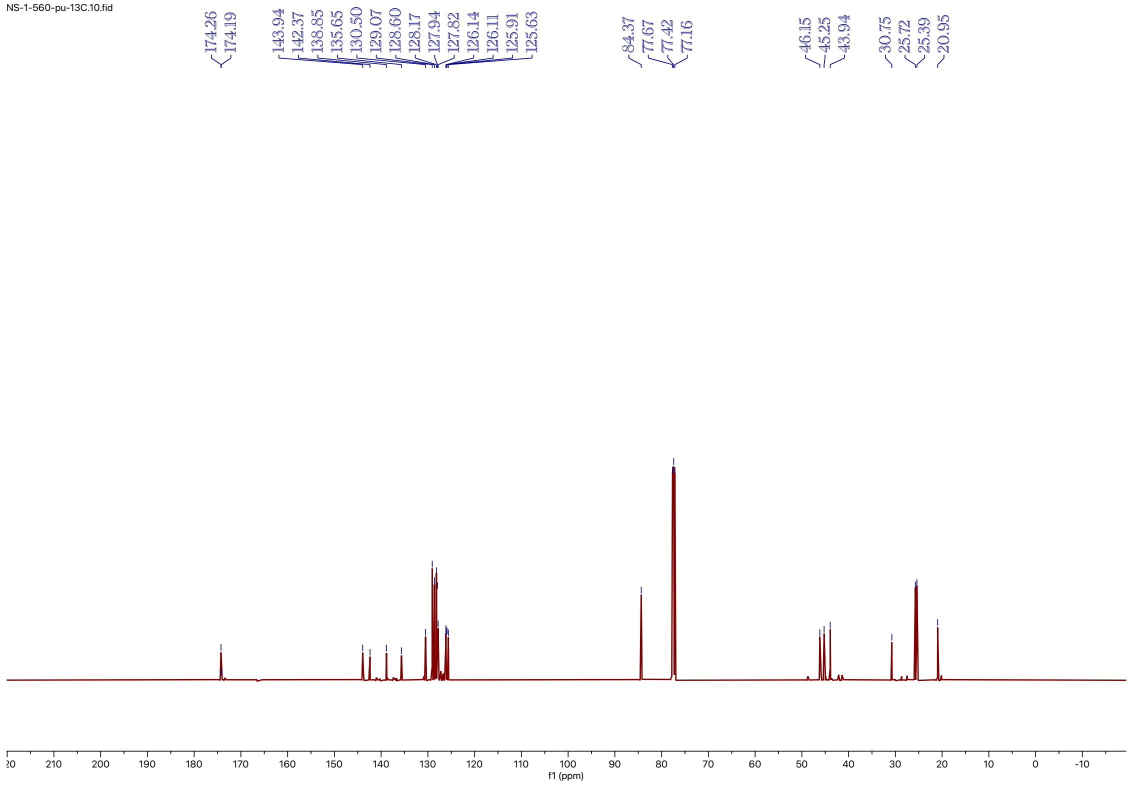
NS-1-560-pu-13C.10.fid
174.26 174.19
143.94 142.37 138.85 135.65 130.50 129.07 128.60 128.17 127.94 127.82 126.14 126.11 125.91 125.63
84.37 77.67 77.42 77.16
46.15 45.25 43.94
30.75 25.72 25.39 20.95
f1 (ppm)

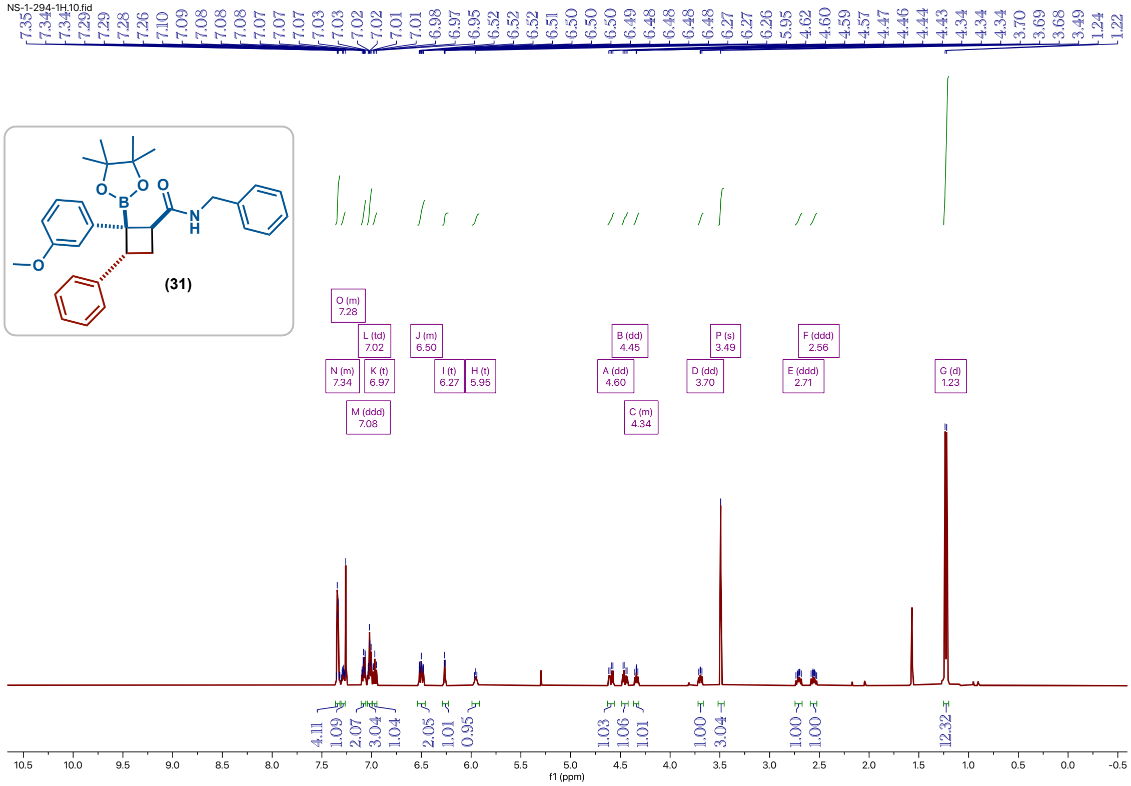
NS-1-294-1H.10.fid
7.35 7.34 7.34 7.29 7.29 7.28 7.26 7.10 7.09 7.08 7.08 7.08 7.07 7.07 7.07 7.03 7.02 7.02 7.01 7.01 6.98 6.97 6.95 6.52 6.52 6.51 6.50 6.50 6.50 6.49 6.48 6.48 6.48 6.48 6.27 6.27 6.26 5.95 4.62 4.60 4.59 4.57 4.47 4.46 4.44 4.43 4.34 4.34 4.34 3.70 3.69 3.68 3.49 1.24 1.22
O
O
B
O
N
H
O
(31)
O (m) 7.28
L (td) 7.02
J (m) 6.50
B (dd) 4.45
P (s) 3.49
F (ddd) 2.56
N (m) 7.34
K (t) 6.97
I (t) 6.27
H (t) 5.95
A (dd) 4.60
D (dd) 3.70
E (ddd) 2.71
G (d) 1.23
M (ddd) 7.08
C (m) 4.34
4.11 1.09 2.07 3.04 1.04 2.05 1.01 0.95 1.03 1.06 1.01 1.00 3.04 1.00 1.00 12.32
10.5 10.0 9.5 9.0 8.5 8.0 7.5 7.0 6.5 6.0 5.5 5.0 4.5 4.0 3.5 3.0 2.5 2.0 1.5 1.0 0.5 0.0 -0.5
f1 (ppm)

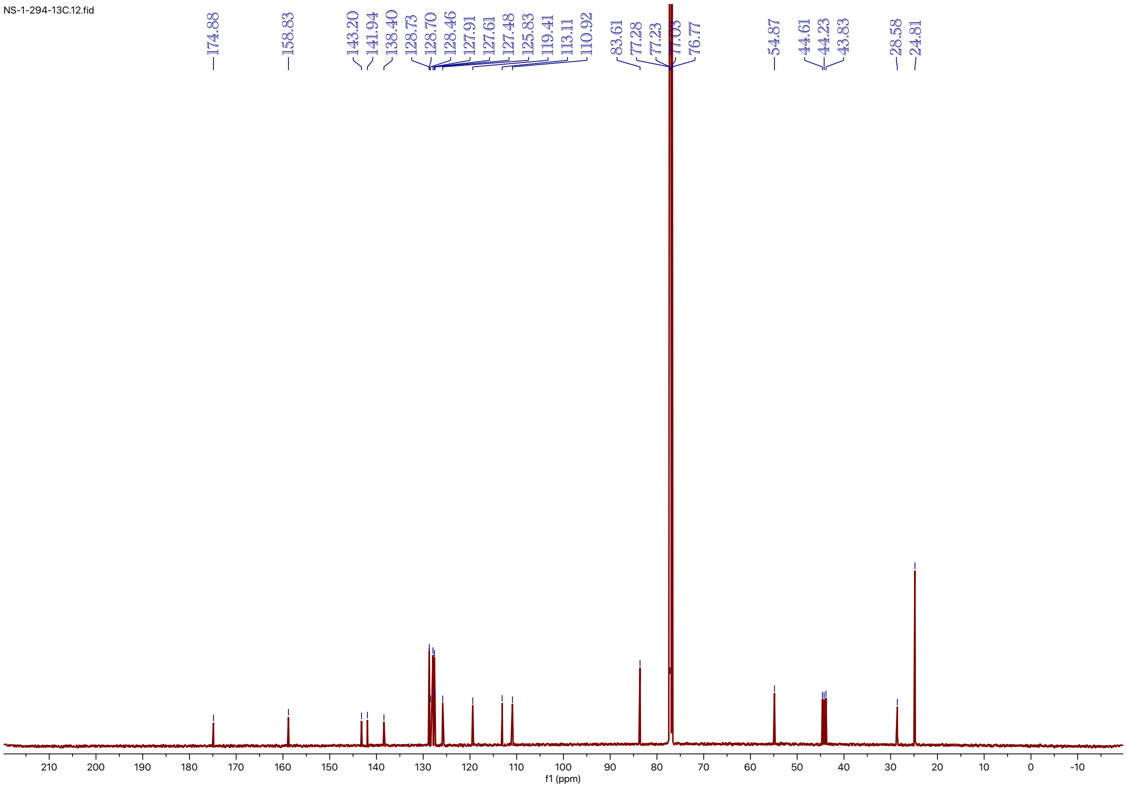
NS-1-294-13C.12.fid
174.88
158.83
143.20 141.94 138.40
128.73 128.70 128.46 127.91 127.61 127.48 125.83 119.41 113.11 110.92
83.61 77.28 77.23 77.03 76.77
54.87
44.61 44.23 43.83
28.58 24.81
210 200 190 180 170 160 150 140 130 120 110 100 90 80 70 60 50 40 30 20 10 0 -10
f1 (ppm)

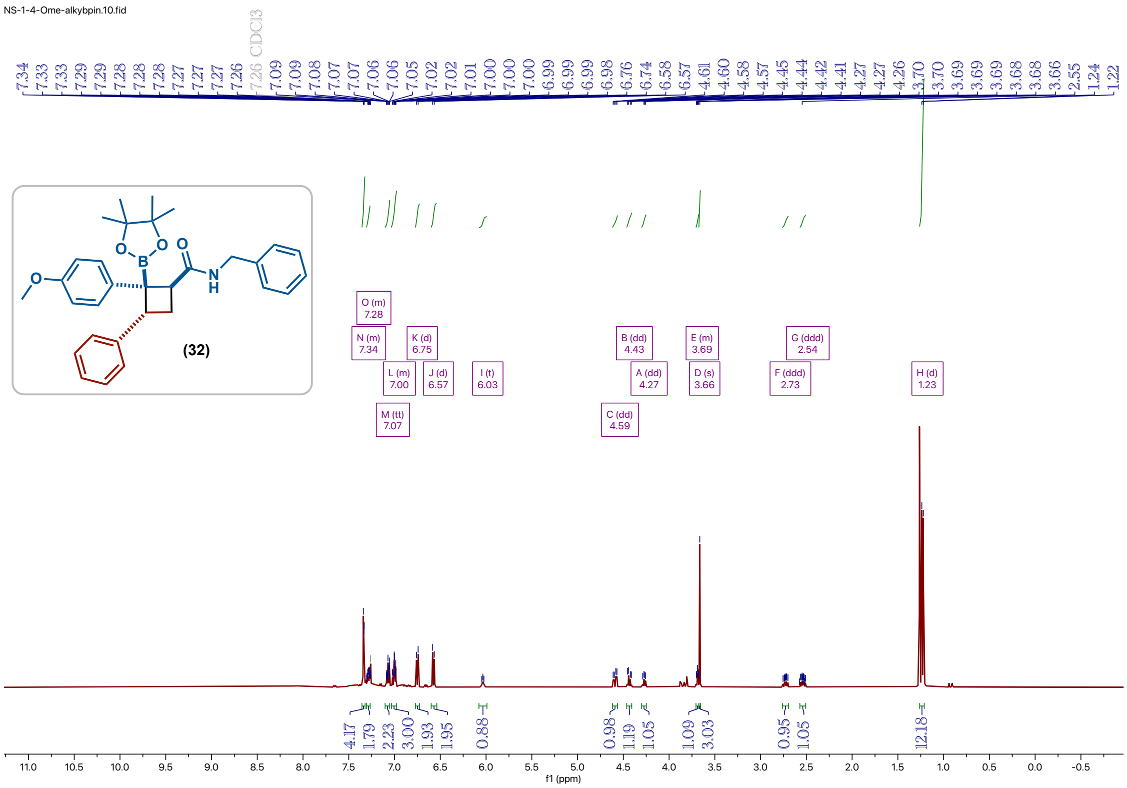
NS-1-4-Ome-alkybpin.10.fid
(32)
f1 (ppm)

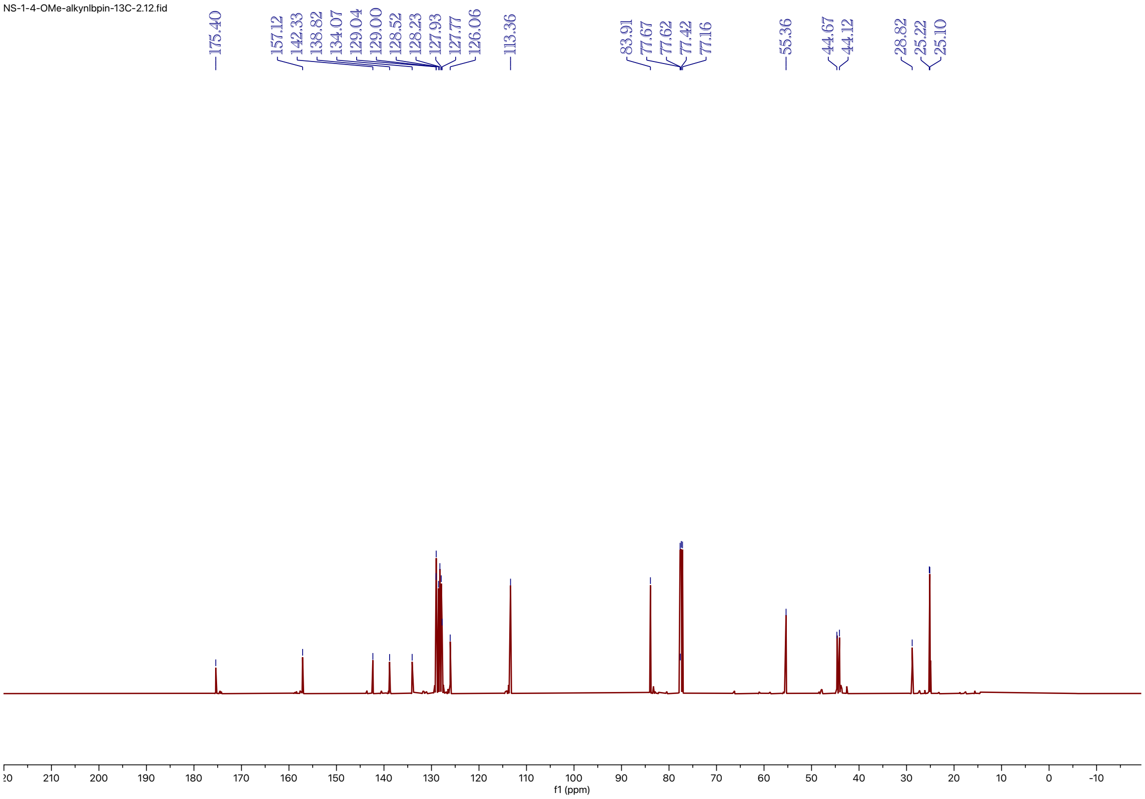
NS-1-4-OMe-alkynlbpin-13C-2.12.fid
f1 (ppm)

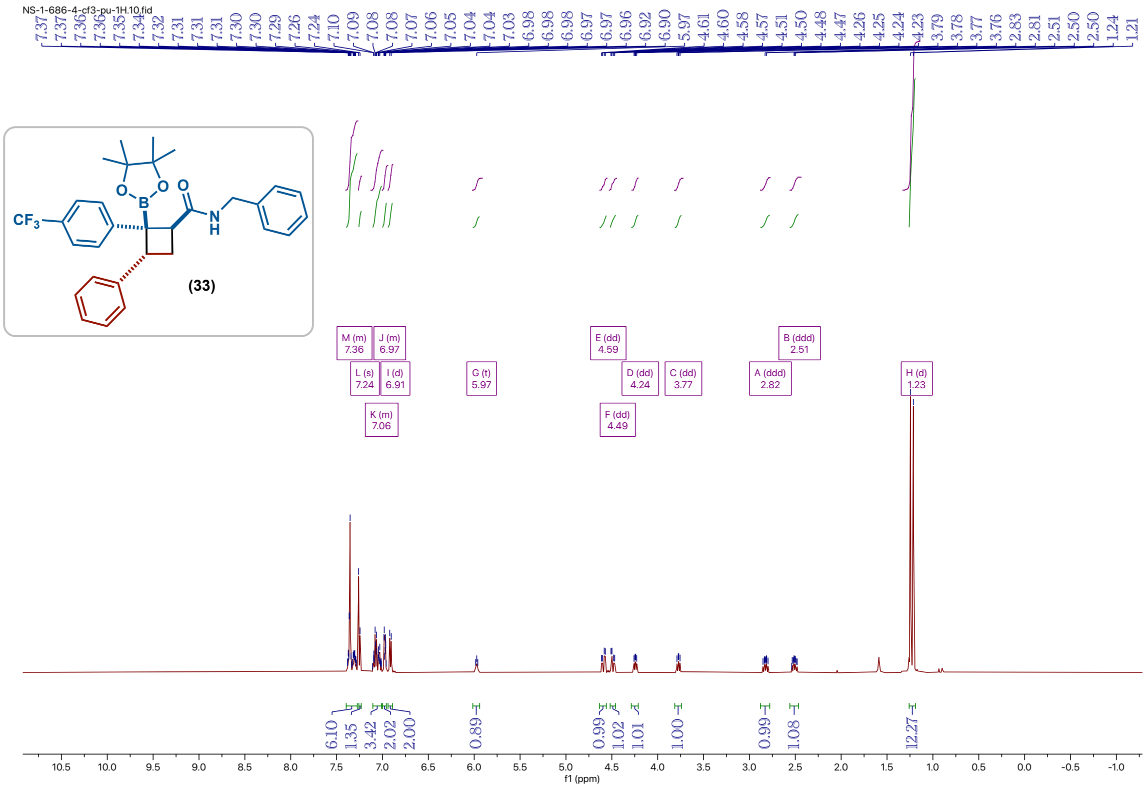

NS-1-686-4-cf3-pu-1H.10.fid
7.37 7.37 7.36 7.36 7.35 7.34 7.32 7.31 7.31 7.30 7.30 7.29 7.26 7.24 7.10 7.09 7.08 7.08 7.07 7.06 7.05 7.04 7.04 7.03 6.98 6.98 6.98 6.97 6.97 6.96 6.92 6.90 5.97 4.61 4.60 4.58 4.57 4.51 4.50 4.48 4.47 4.26 4.25 4.24 4.23 3.79 3.78 3.77 3.76 2.83 2.81 2.51 2.50 2.50 1.24 1.21
CF3
O
B
O
N
H
(33)
M (m) 7.36
J (m) 6.97
L (s) 7.24
I (d) 6.91
K (m) 7.06
G (t) 5.97
E (dd) 4.59
F (dd) 4.49
D (dd) 4.24
C (dd) 3.77
A (ddd) 2.82
B (ddd) 2.51
H (d) 1.23
6.10 1.35 3.42 2.02 2.00 0.89 0.99 1.02 1.01 1.00 0.99 1.08 12.27
10.5 10.0 9.5 9.0 8.5 8.0 7.5 7.0 6.5 6.0 5.5 5.0 4.5 4.0 3.5 3.0 2.5 2.0 1.5 1.0 0.5 0.0 -0.5 -1.0
f1 (ppm)

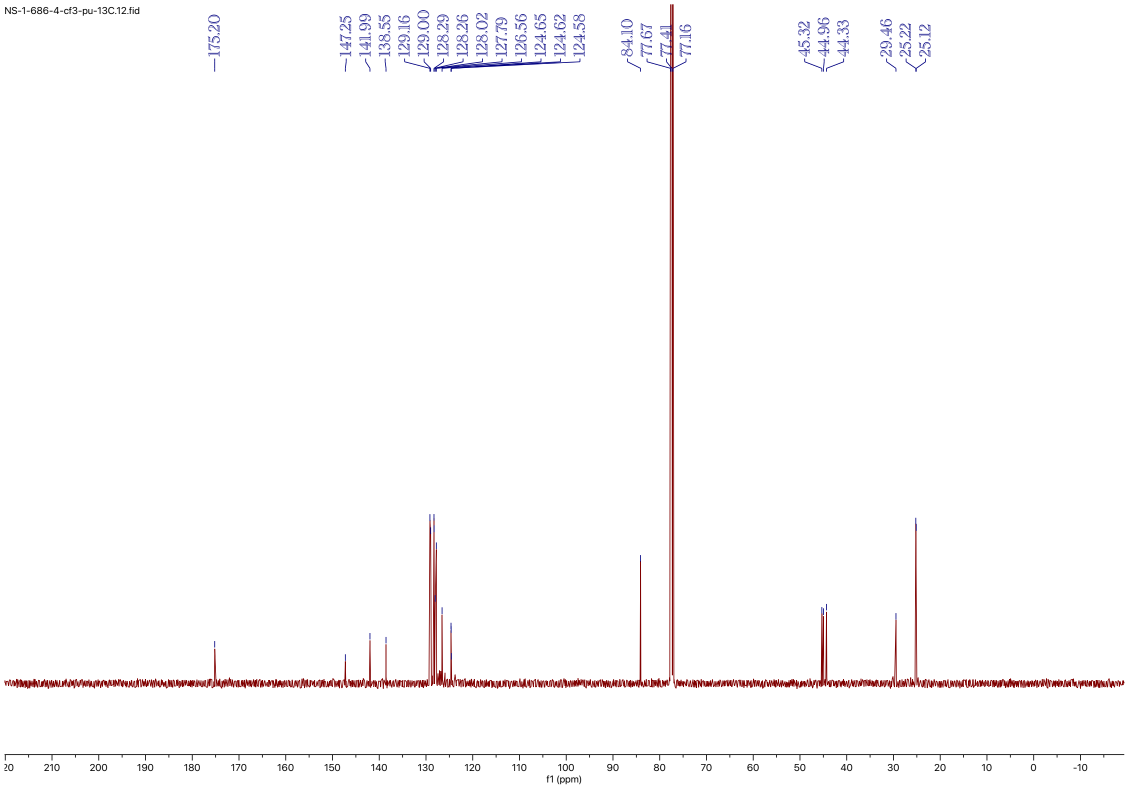

NS-1-686-4-cf3-pu-13C.12.fid
175.20
147.25 141.99 138.55 129.16 129.00 128.29 128.26 128.02 127.79 126.56 124.65 124.62 124.58
84.10 77.67 77.41 77.16
45.32 44.96 44.33
29.46 25.22 25.12
20 210 200 190 180 170 160 150 140 130 120 110 100 90 80 70 60 50 40 30 20 10 0 -10
f1 (ppm)

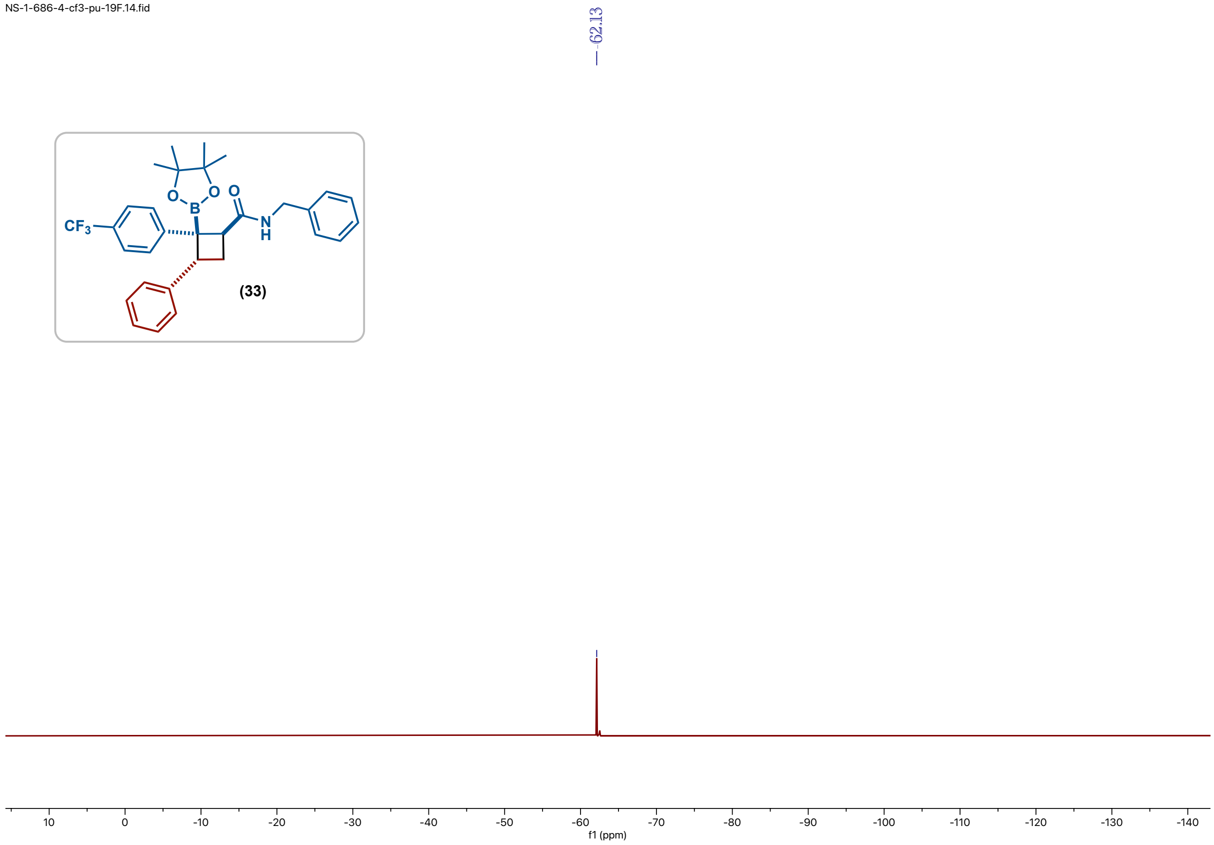
NS-1-686-4-cf3-pu-19F.14.fid
-62.13
CF_3
O
B
O
O
N
H
(33)
10 0 -10 -20 -30 -40 -50 -60 -70 -80 -90 -100 -110 -120 -130 -140
f1 (ppm)

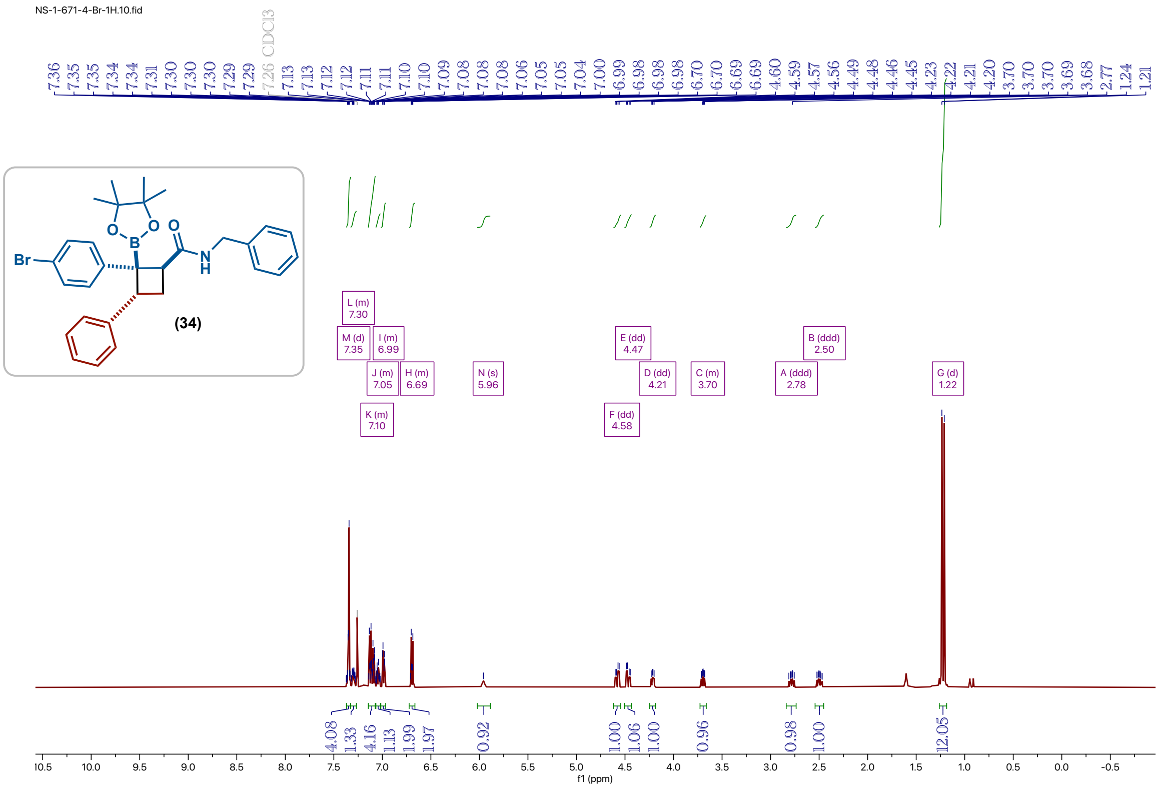
NS-1-671-4-Br-1H.10.fid
7.36 7.35 7.35 7.34 7.34 7.31 7.30 7.30 7.30 7.29 7.29 7.26 CDCl3 7.13 7.13 7.12 7.12 7.11 7.11 7.10 7.10 7.09 7.08 7.08 7.08 7.06 7.05 7.05 7.04 7.00 6.99 6.98 6.98 6.98 6.70 6.70 6.69 6.69 4.60 4.59 4.57 4.56 4.49 4.48 4.46 4.45 4.23 4.22 4.21 4.20 3.70 3.70 3.70 3.69 3.68 2.77 1.24 1.21
Br
O
B
O
O
N
H
(34)
L (m) 7.30
M (d) 7.35
I (m) 6.99
J (m) 7.05
H (m) 6.69
K (m) 7.10
N (s) 5.96
E (dd) 4.47
D (dd) 4.21
F (dd) 4.58
C (m) 3.70
B (ddd) 2.50
A (ddd) 2.78
G (d) 1.22
4.08 1.33 4.16 1.13 1.99 1.97 0.92 1.00 1.06 1.00 0.96 0.98 1.00 12.05
10.5 10.0 9.5 9.0 8.5 8.0 7.5 7.0 6.5 6.0 5.5 5.0 4.5 4.0 3.5 3.0 2.5 2.0 1.5 1.0 0.5 0.0 -0.5
f1 (ppm)

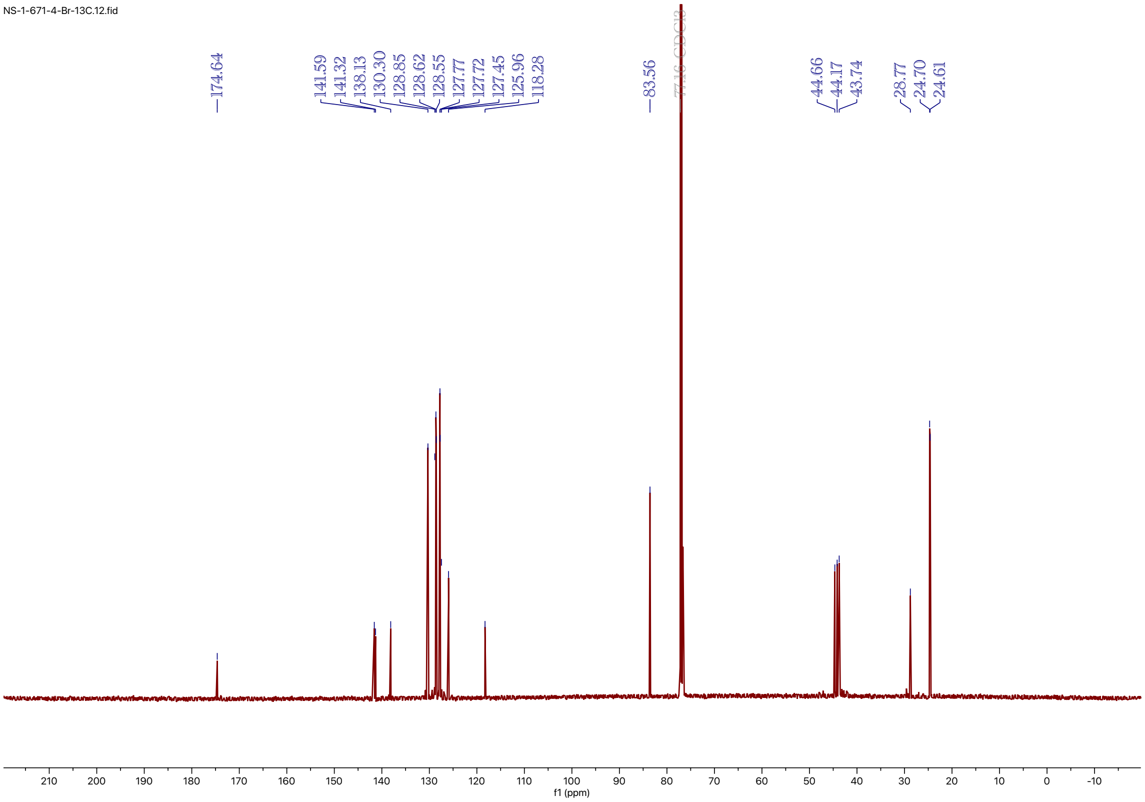
NS-1-671-4-Br-13C.12.fid
174.64
141.59 141.32 138.13 130.30 128.85 128.62 128.55 127.77 127.72 127.45 125.96 118.28
83.56
77.16 CDCl3
44.66 44.17 43.74
28.77 24.70 24.61
210 200 190 180 170 160 150 140 130 120 110 100 90 80 70 60 50 40 30 20 10 0 -10
f1 (ppm)

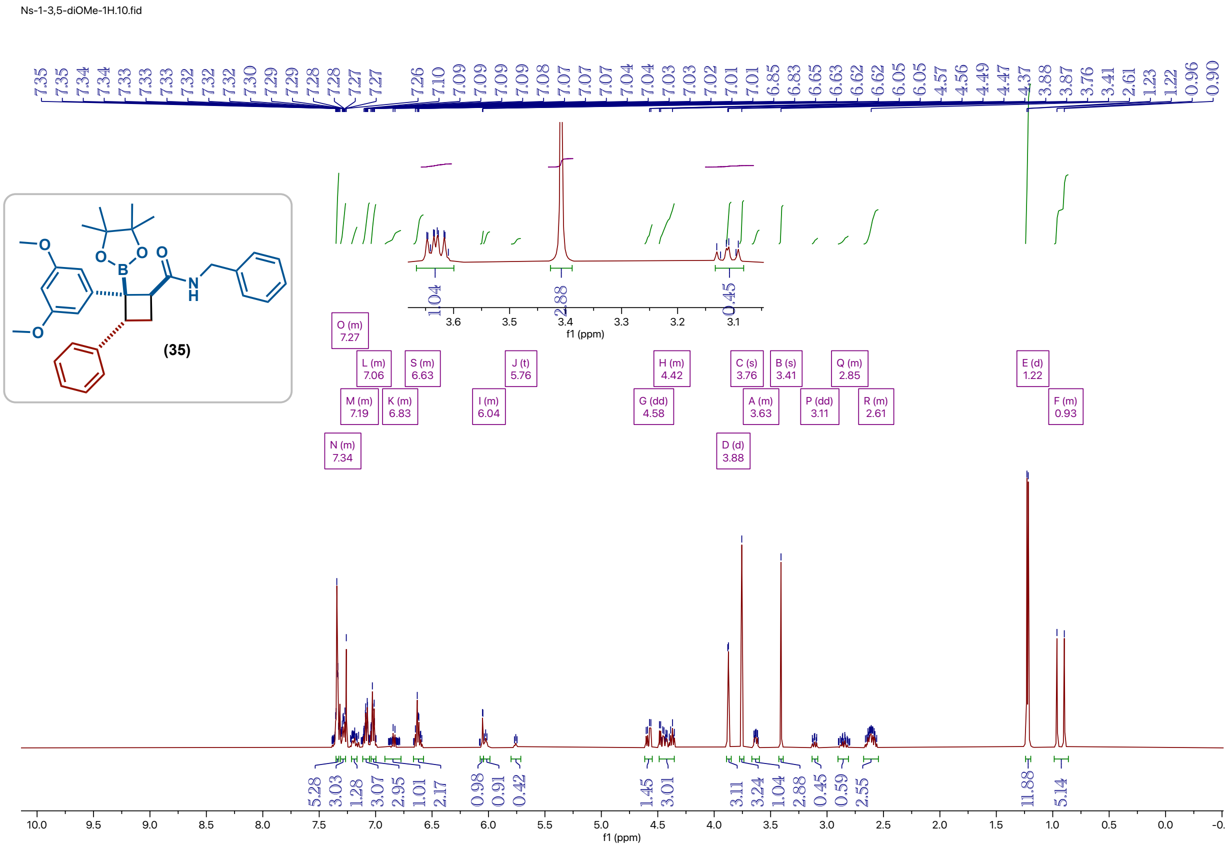
Ns-1-3,5-diOMe-1H.10.fid
(35)
f1 (ppm)

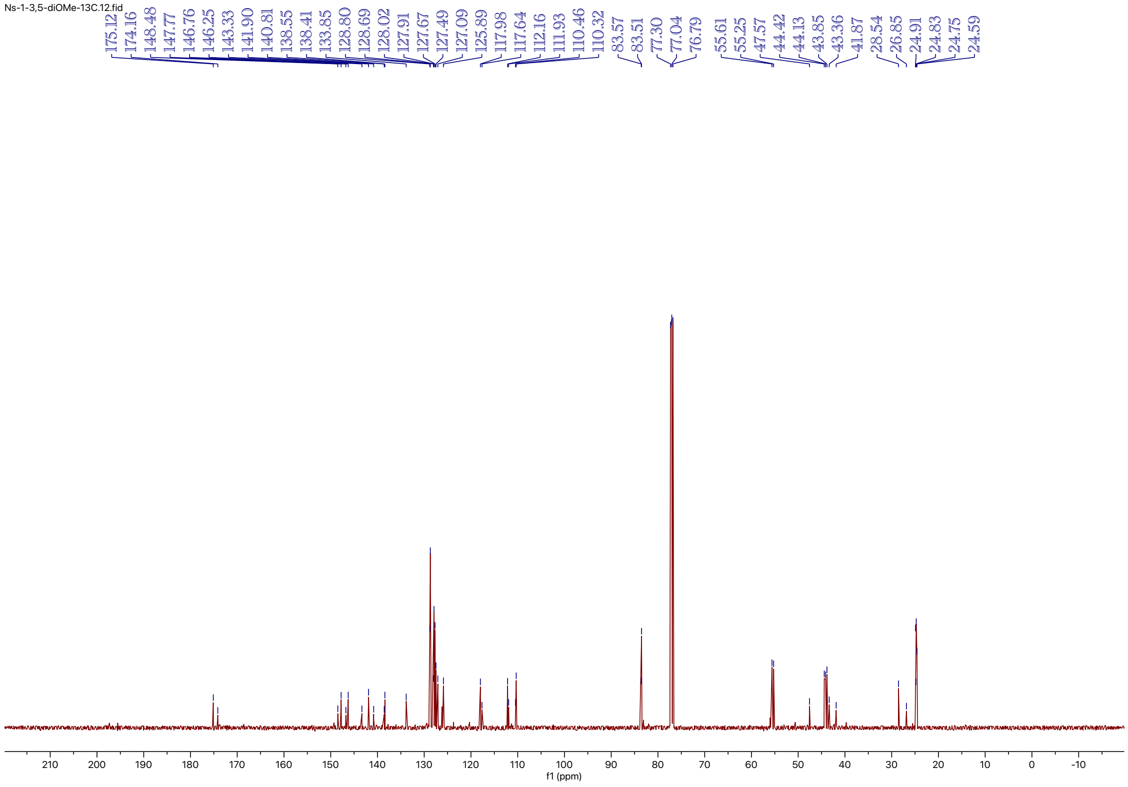
Ns-1-3,5-diOMe-13C.12.fid
f1 (ppm)

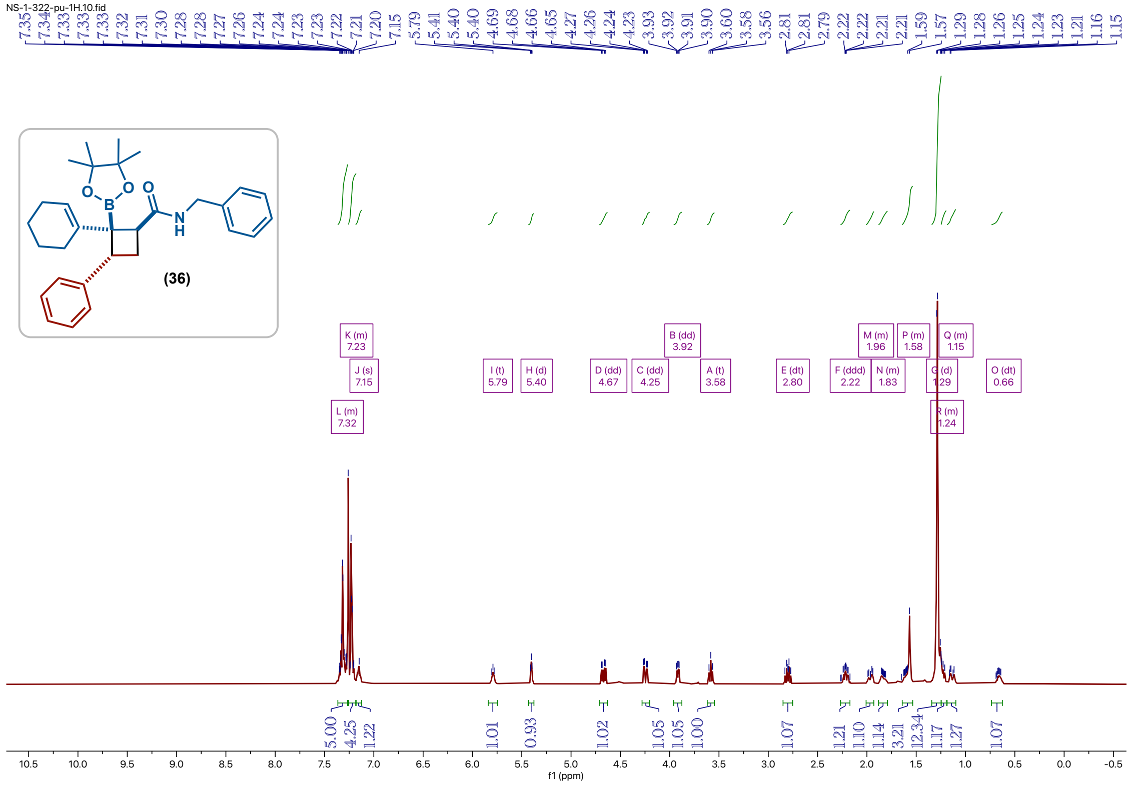
NS-1-322-pu-1H.10.fid
7.35 7.34 7.33 7.33 7.33 7.32 7.31 7.30 7.28 7.28 7.27 7.26 7.24 7.24 7.23 7.23 7.22 7.21 7.20 7.15 5.79 5.41 5.40 5.40 4.69 4.68 4.66 4.65 4.27 4.26 4.24 4.23 3.93 3.92 3.91 3.90 3.60 3.58 3.56 2.81 2.81 2.79 2.22 2.22 2.21 2.21 1.59 1.57 1.29 1.28 1.26 1.25 1.24 1.23 1.21 1.16 1.15
(36)
O
B
N
H
K (m) 7.23
J (s) 7.15
L (m) 7.32
I (t) 5.79
H (d) 5.40
D (dd) 4.67
C (dd) 4.25
B (dd) 3.92
A (t) 3.58
E (dt) 2.80
F (ddd) 2.22
M (m) 1.96
N (m) 1.83
P (m) 1.58
G (d) 1.29
R (m) 1.24
Q (m) 1.15
O (dt) 0.66
5.00 4.25 1.22 1.01 0.93 1.02 1.05 1.05 1.00 1.07 1.21 1.10 1.14 3.21 12.34 1.17 1.27 1.07
f1 (ppm)

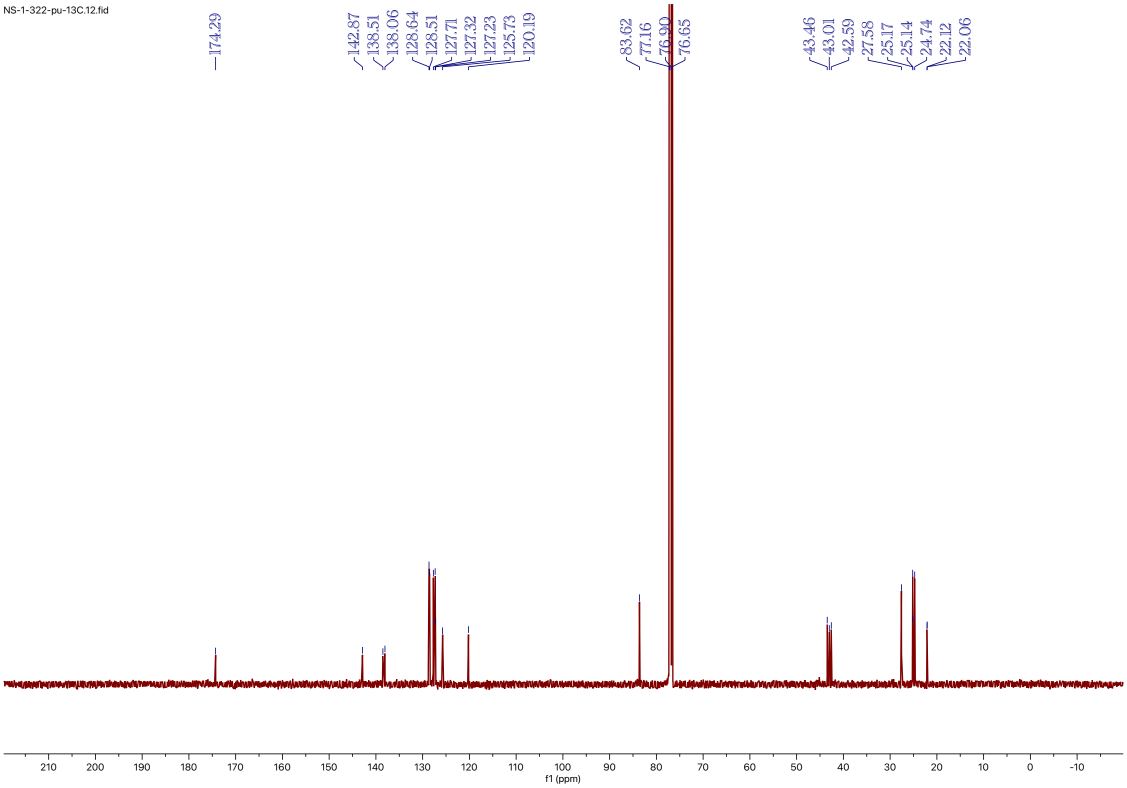
NS-1-322-pu-13C.12.fid
174.29
142.87 138.51 138.06 128.64 128.51 127.71 127.32 127.23 125.73 120.19
83.62 77.16 76.90 76.65
43.46 43.01 42.59 27.58 25.17 25.14 24.74 22.12 22.06
f1 (ppm)

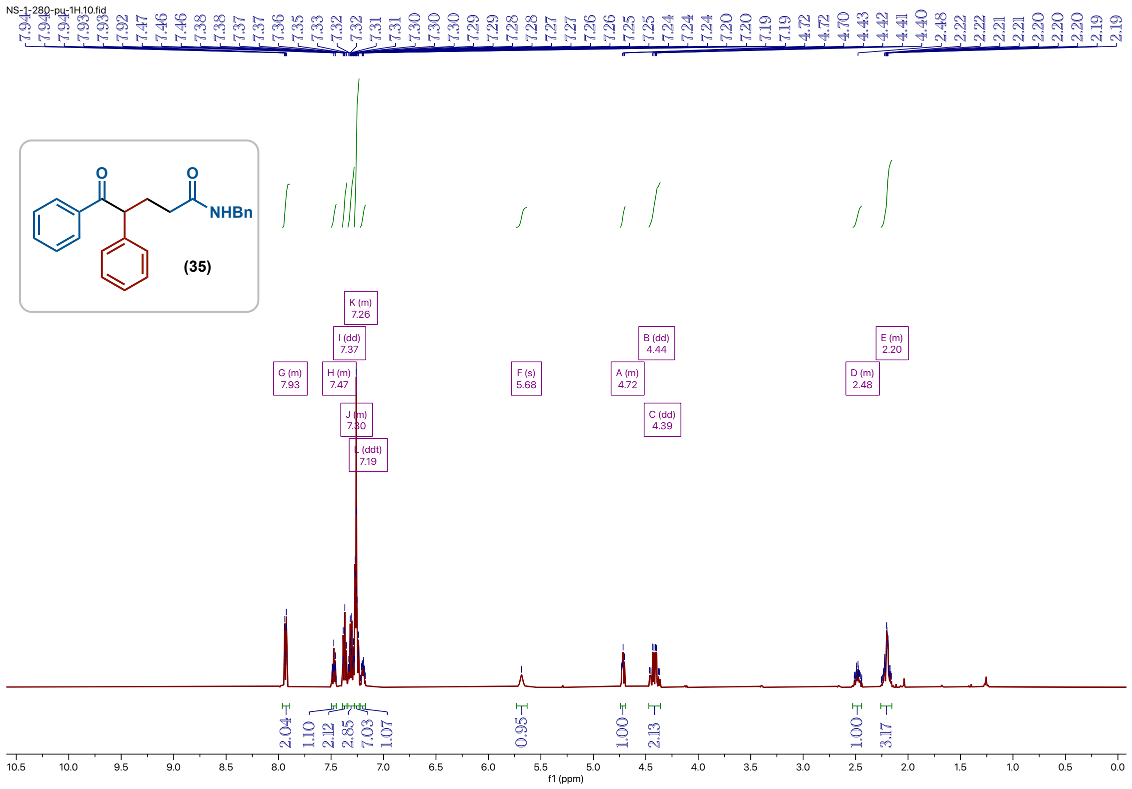

NS-1-280-pu-1H.10.fid
O
O
NHBn
(35)
K (m) 7.26
I (dd) 7.37
B (dd) 4.44
E (m) 2.20
G (m) 7.93
H (m) 7.47
F (s) 5.68
A (m) 4.72
D (m) 2.48
J (m) 7.30
C (dd) 4.39
L (ddt) 7.19
2.04
1.10
2.12
2.85
7.03
1.07
0.95
1.00
2.13
1.00
3.17
f1 (ppm)

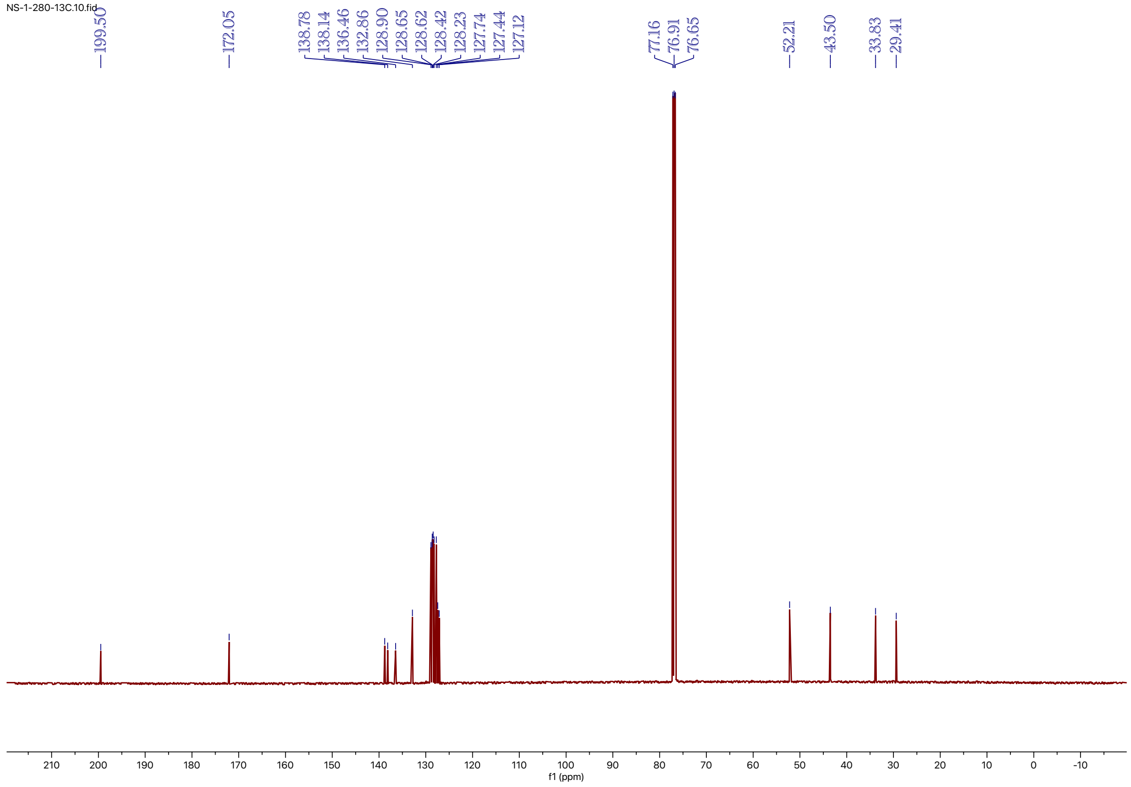

NS-1-280-13C.10.fid
199.50
172.05
138.78
138.14
136.46
132.86
128.90
128.65
128.62
128.42
128.23
127.74
127.44
127.12
77.16
76.91
76.65
52.21
43.50
33.83
29.41
f1 (ppm)

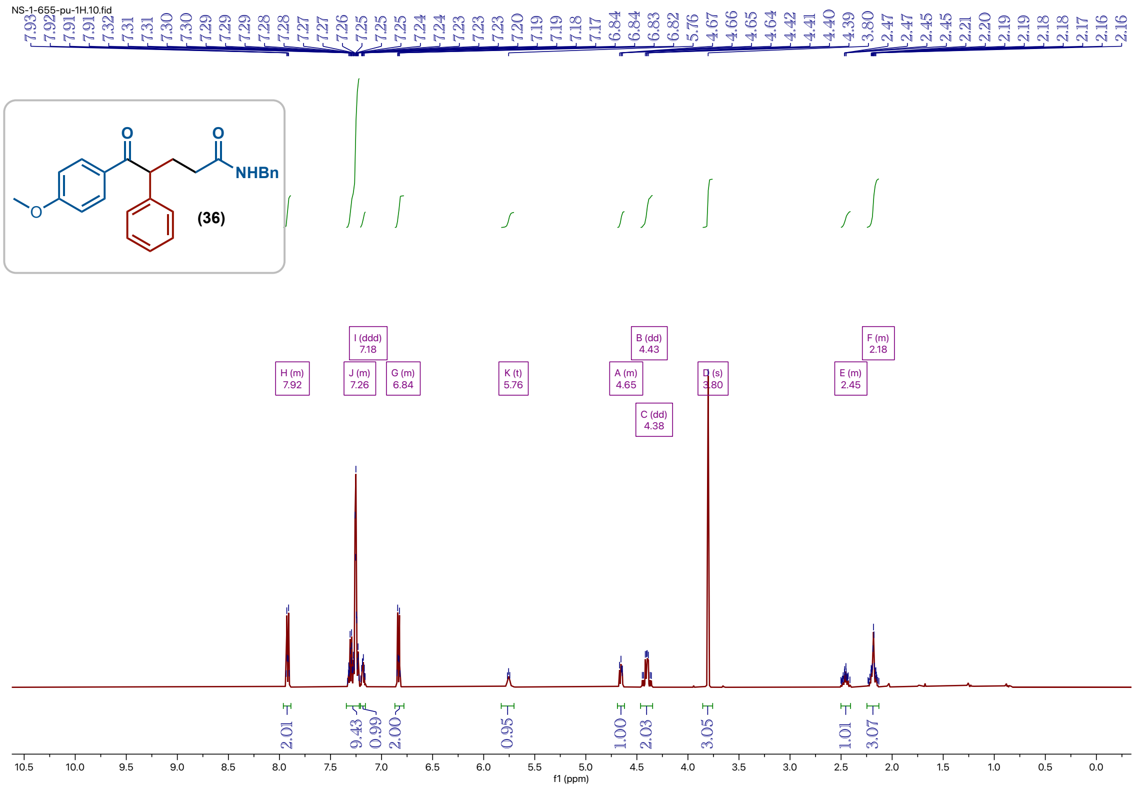

NS-1-655-pu-1H.10.fid
7.93 7.92 7.91 7.91 7.32 7.31 7.31 7.30 7.30 7.29 7.29 7.29 7.29 7.28 7.28 7.27 7.27 7.26 7.25 7.25 7.25 7.24 7.24 7.23 7.23 7.23 7.20 7.19 7.19 7.18 7.17 6.84 6.84 6.83 6.82 5.76 4.67 4.66 4.65 4.64 4.42 4.41 4.40 4.39 3.80 2.47 2.47 2.45 2.45 2.21 2.20 2.19 2.19 2.18 2.18 2.17 2.16 2.16
H (m) 7.92
I (ddd) 7.18
J (m) 7.26
G (m) 6.84
K (t) 5.76
B (dd) 4.43
A (m) 4.65
C (dd) 4.38
D (s) 3.80
F (m) 2.18
E (m) 2.45
2.01
9.43
0.99
2.00
0.95
1.00
2.03
3.05
1.01
3.07
10.5 10.0 9.5 9.0 8.5 8.0 7.5 7.0 6.5 6.0 5.5 5.0 4.5 4.0 3.5 3.0 2.5 2.0 1.5 1.0 0.5 0.0
f1 (ppm)

(36)

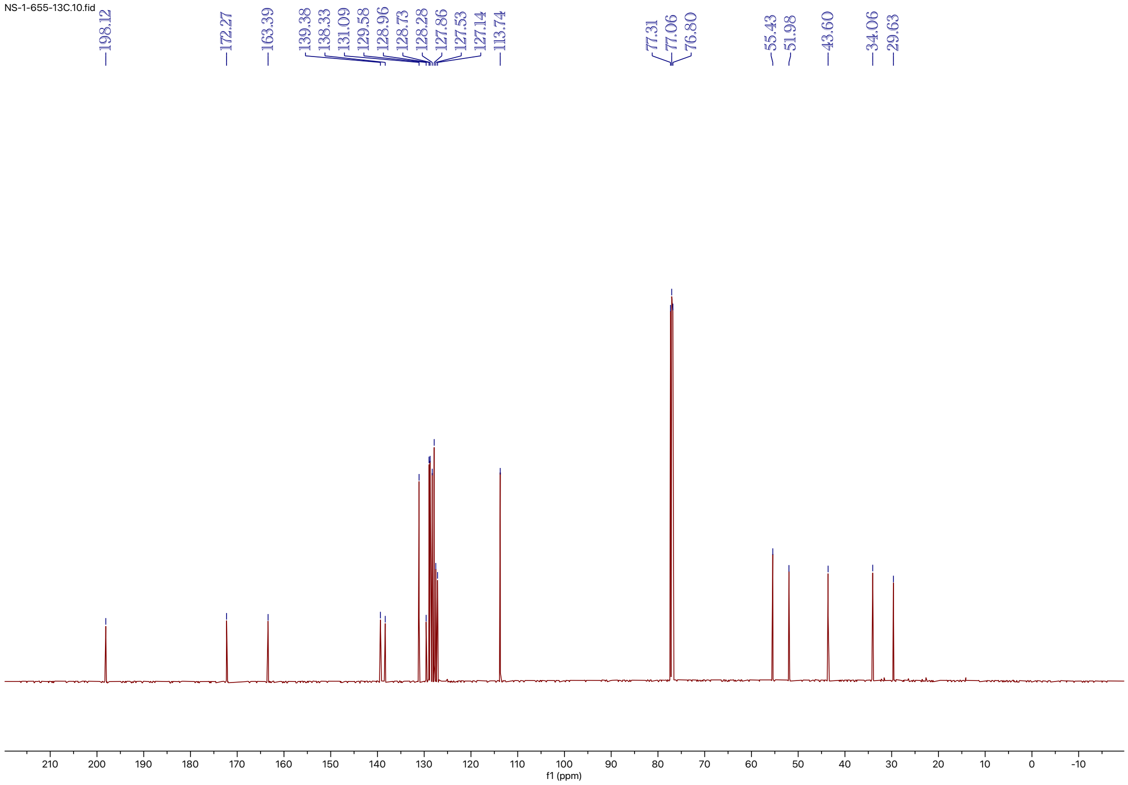

NS-1-655-13C.10.fid
198.12
172.27
163.39
139.38 138.33 131.09 129.58 128.96 128.73 128.28 127.86 127.53 127.14
113.74
77.31 77.06 76.80
55.43
51.98
43.60
34.06
29.63
210 200 190 180 170 160 150 140 130 120 110 100 90 80 70 60 50 40 30 20 10 0 -10
f1 (ppm)

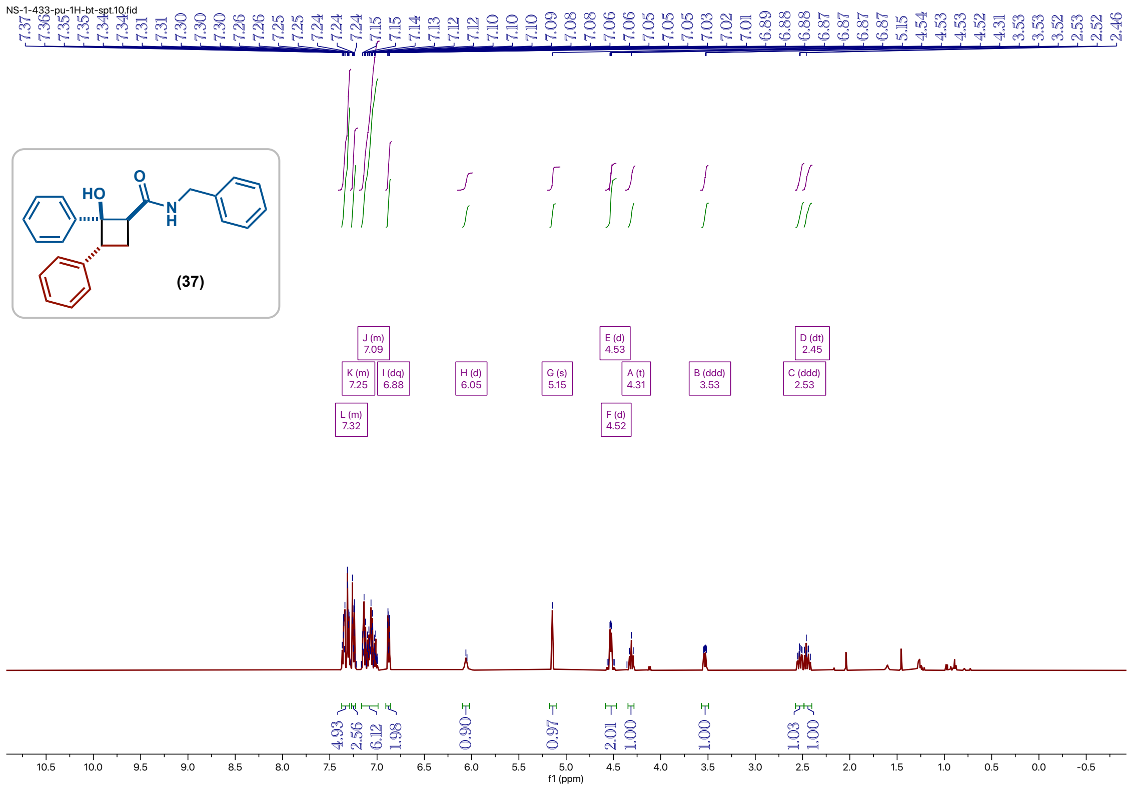
NS-1-433-pu-1H-bt-spt.10.fid
7.37 7.36 7.35 7.35 7.34 7.34 7.31 7.31 7.30 7.30 7.30 7.26 7.26 7.25 7.25 7.24 7.24 7.24 7.15 7.15 7.14 7.13 7.12 7.12 7.10 7.10 7.10 7.09 7.08 7.08 7.06 7.06 7.05 7.05 7.05 7.03 7.02 7.01 6.89 6.88 6.88 6.87 6.87 6.87 6.87 5.15 4.54 4.53 4.53 4.52 4.31 3.53 3.53 3.52 2.53 2.52 2.46
HO
O
N
H
(37)
J (m) 7.09
E (d) 4.53
D (dt) 2.45
K (m) 7.25
I (dq) 6.88
H (d) 6.05
G (s) 5.15
A (t) 4.31
B (ddd) 3.53
C (ddd) 2.53
L (m) 7.32
F (d) 4.52
4.93 2.56 6.12 1.98 0.90 0.97 2.01 1.00 1.00 1.03 1.00
10.5 10.0 9.5 9.0 8.5 8.0 7.5 7.0 6.5 6.0 5.5 5.0 4.5 4.0 3.5 3.0 2.5 2.0 1.5 1.0 0.5 0.0 -0.5
f1 (ppm)

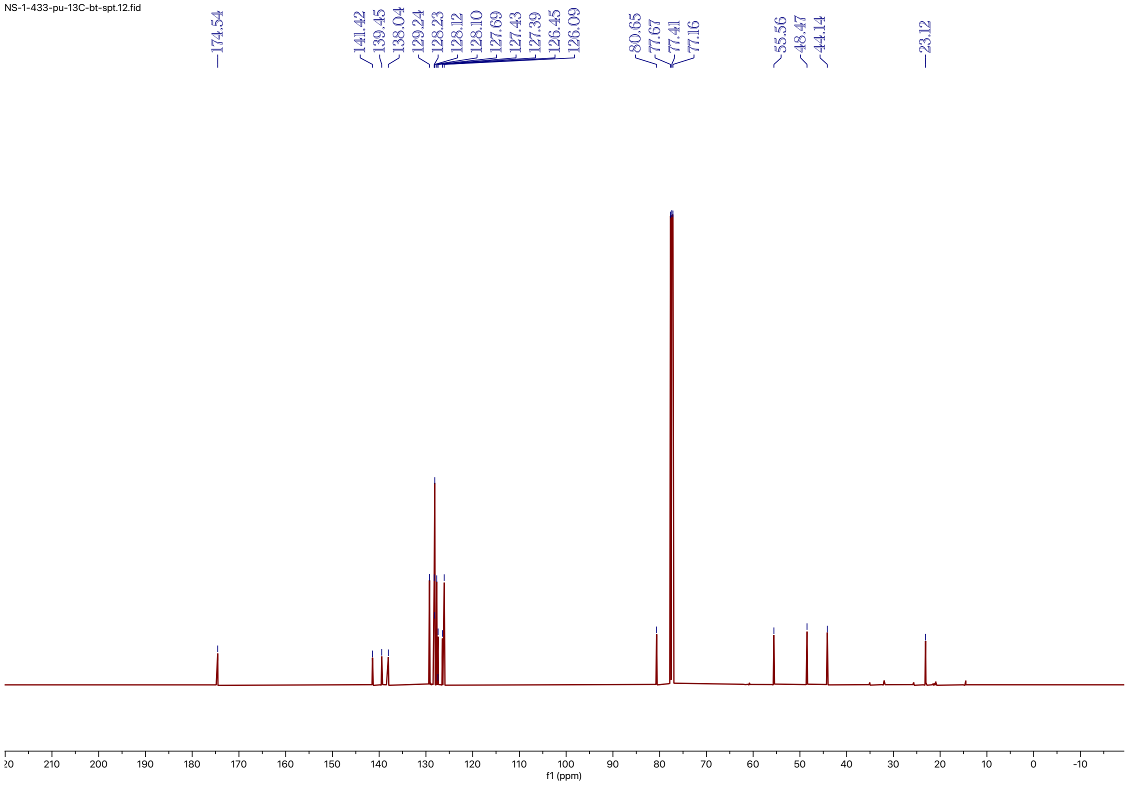
NS-1-433-pu-13C-bt-spt.12.fid
174.54
141.42 139.45 138.04 129.24 128.23 128.12 128.10 127.69 127.43 127.39 126.45 126.09
80.65 77.67 77.41 77.16
55.56 48.47 44.14
23.12
210 200 190 180 170 160 150 140 130 120 110 100 90 80 70 60 50 40 30 20 10 0 -10
f1 (ppm)

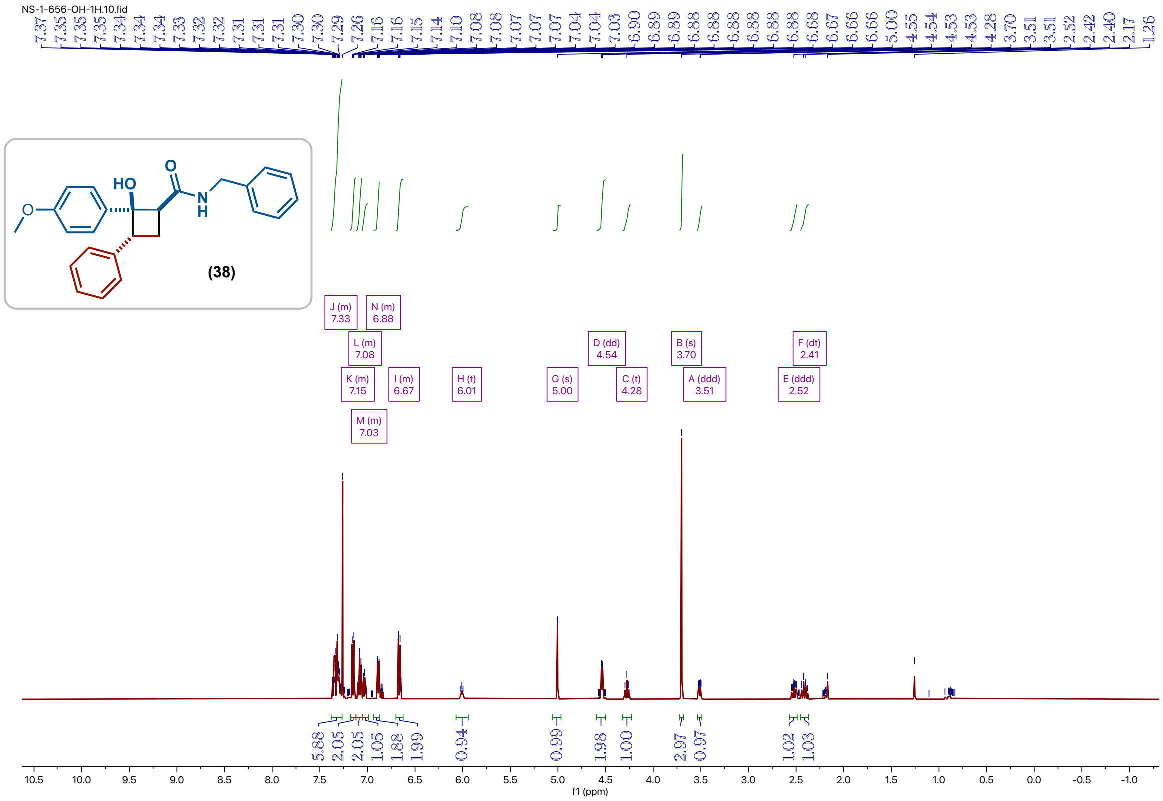
NS-1-656-OH-1H.10.fid
7.37 7.35 7.35 7.35 7.34 7.34 7.34 7.33 7.32 7.32 7.31 7.31 7.31 7.30 7.30 7.29 7.26 7.16 7.16 7.15 7.14 7.10 7.08 7.08 7.07 7.07 7.07 7.04 7.04 7.03 6.90 6.89 6.89 6.88 6.88 6.88 6.88 6.88 6.88 6.68 6.67 6.66 6.66 5.00 4.55 4.54 4.53 4.53 4.28 3.70 3.51 3.51 2.52 2.42 2.40 2.17 1.26
HO
O
N
H
(38)
J (m) 7.33
N (m) 6.88
L (m) 7.08
K (m) 7.15
I (m) 6.67
M (m) 7.03
H (t) 6.01
G (s) 5.00
D (dd) 4.54
C (t) 4.28
B (s) 3.70
A (ddd) 3.51
F (dt) 2.41
E (ddd) 2.52
5.88 2.05 2.05 1.05 1.88 1.99 0.94 0.99 1.98 1.00 2.97 0.97 1.02 1.03
10.5 10.0 9.5 9.0 8.5 8.0 7.5 7.0 6.5 6.0 5.5 5.0 4.5 4.0 3.5 3.0 2.5 2.0 1.5 1.0 0.5 0.0 -0.5 -1.0
f1 (ppm)

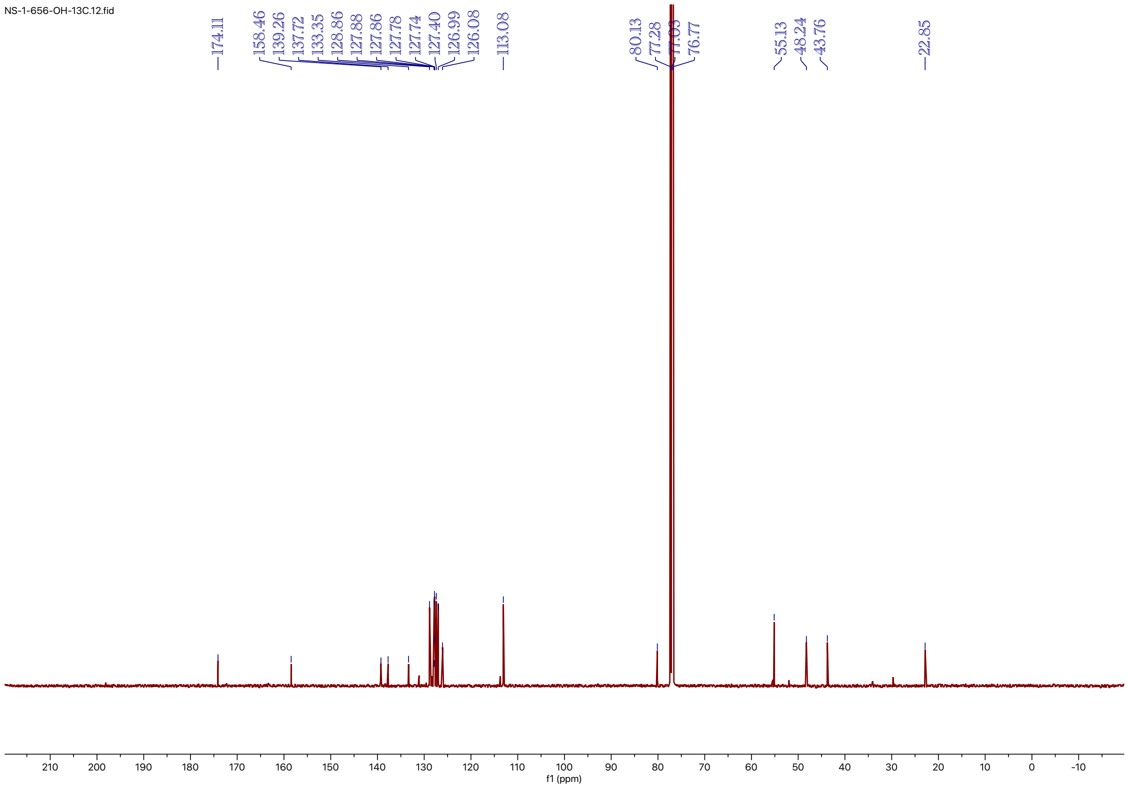
NS-1-656-OH-13C.12.fid
174.11
158.46 139.26 137.72 133.35 128.86 127.88 127.86 127.78 127.74 127.40 126.99 126.08
113.08
80.13 77.28 77.03 76.77
55.13 48.24 43.76
22.85
210 200 190 180 170 160 150 140 130 120 110 100 90 80 70 60 50 40 30 20 10 0 -10
f1 (ppm)

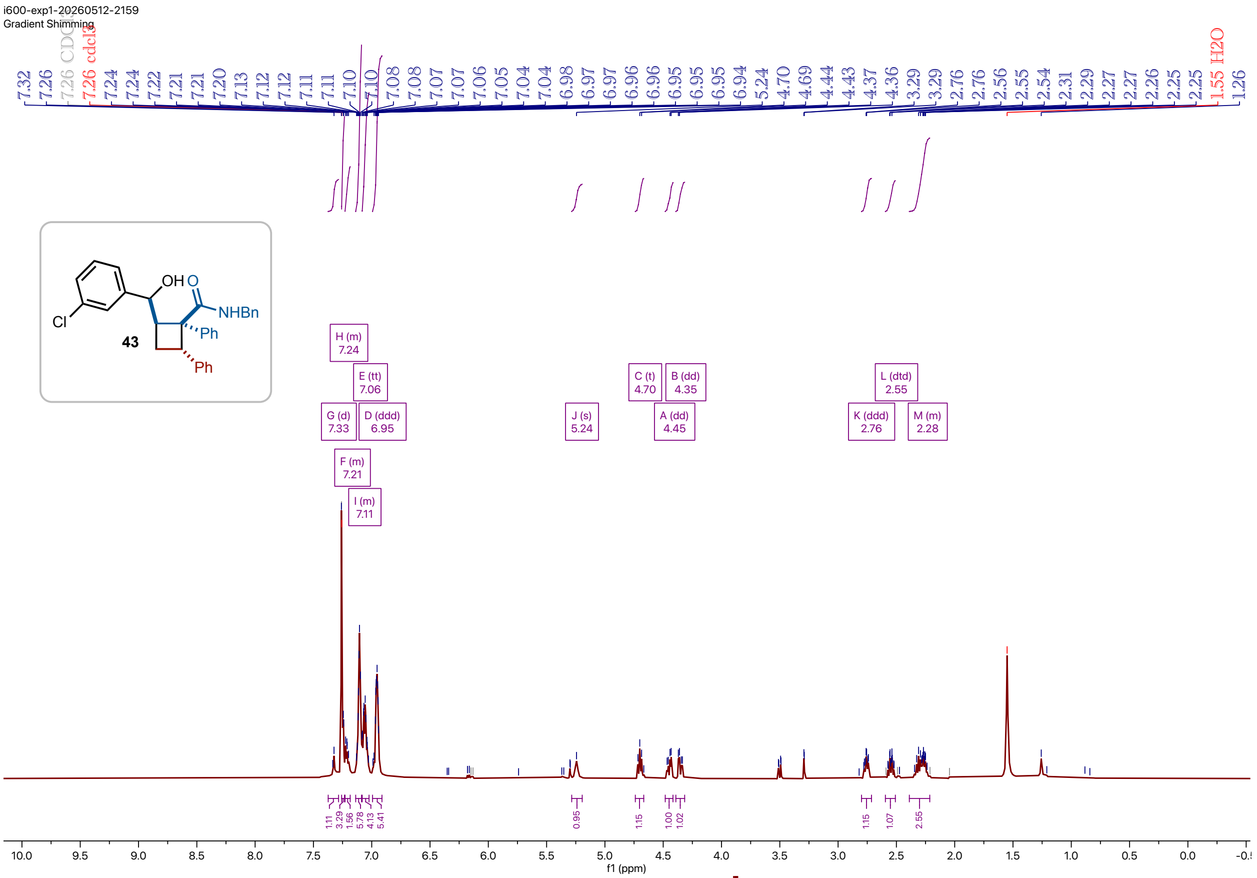
i600-exp1-20260512-2159
Gradient Shimming
7.32 7.26 7.26 CDCl3 7.26 cdcl3 7.24 7.24 7.22 7.21 7.21 7.20 7.13 7.12 7.12 7.11 7.11 7.10 7.10 7.08 7.08 7.07 7.07 7.06 7.05 7.04 7.04 6.98 6.97 6.97 6.96 6.96 6.95 6.95 6.94 5.24 4.70 4.69 4.44 4.43 4.37 4.36 3.29 3.29 2.76 2.76 2.56 2.55 2.54 2.31 2.29 2.27 2.26 2.25 2.25 1.55 H2O 1.26
OH O
NHBn
Cl
43
Ph
Ph
H (m) 7.24
E (tt) 7.06
G (d) 7.33
D (ddd) 6.95
F (m) 7.21
I (m) 7.11
J (s) 5.24
C (t) 4.70
B (dd) 4.35
A (dd) 4.45
L (dtd) 2.55
K (ddd) 2.76
M (m) 2.28
1.11 3.29 1.56 5.78 4.13 5.41 0.95 1.15 1.00 1.02 1.15 1.07 2.55
10.0 9.5 9.0 8.5 8.0 7.5 7.0 6.5 6.0 5.5 5.0 4.5 4.0 3.5 3.0 2.5 2.0 1.5 1.0 0.5 0.0
f1 (ppm)

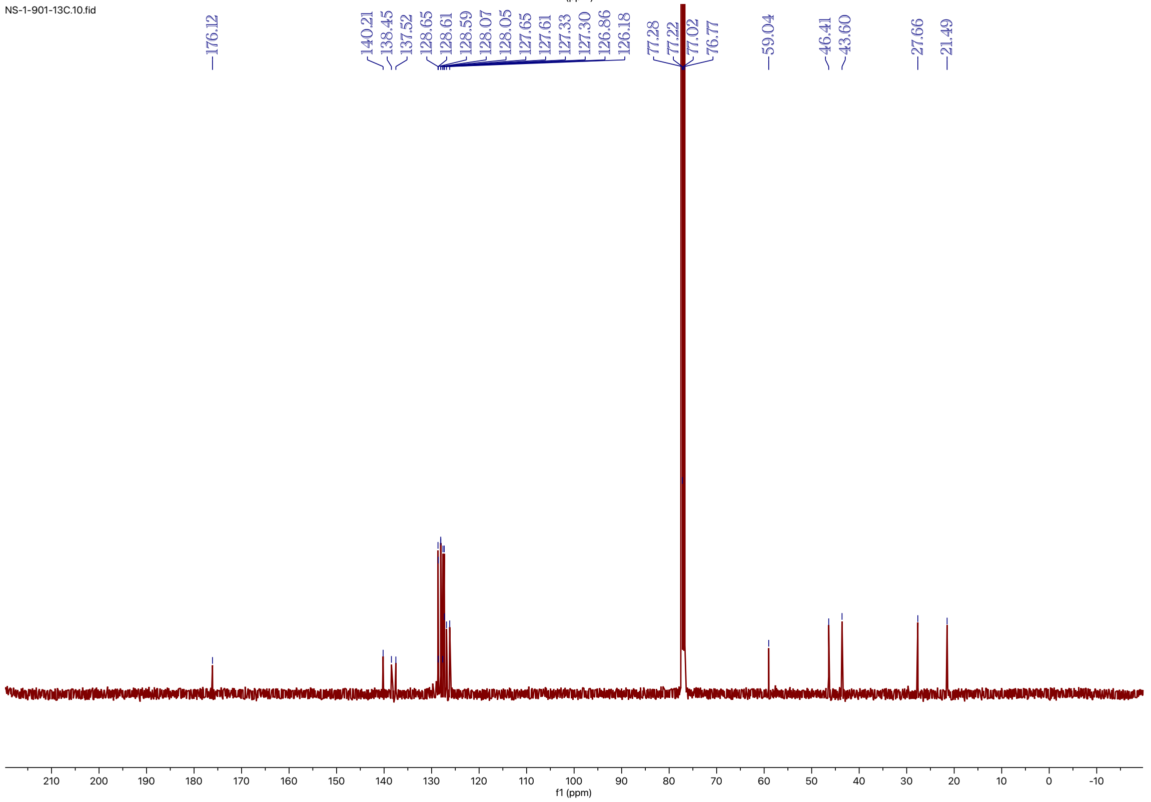
NS-1-901-13C.10.fid
176.12 140.21 138.45 137.52 128.65 128.61 128.59 128.07 128.05 127.65 127.61 127.33 127.30 126.86 126.18 77.28 77.22 77.02 76.77 59.04 46.41 43.60 27.66 21.49
210 200 190 180 170 160 150 140 130 120 110 100 90 80 70 60 50 40 30 20 10 0 -10
f1 (ppm)

COSY

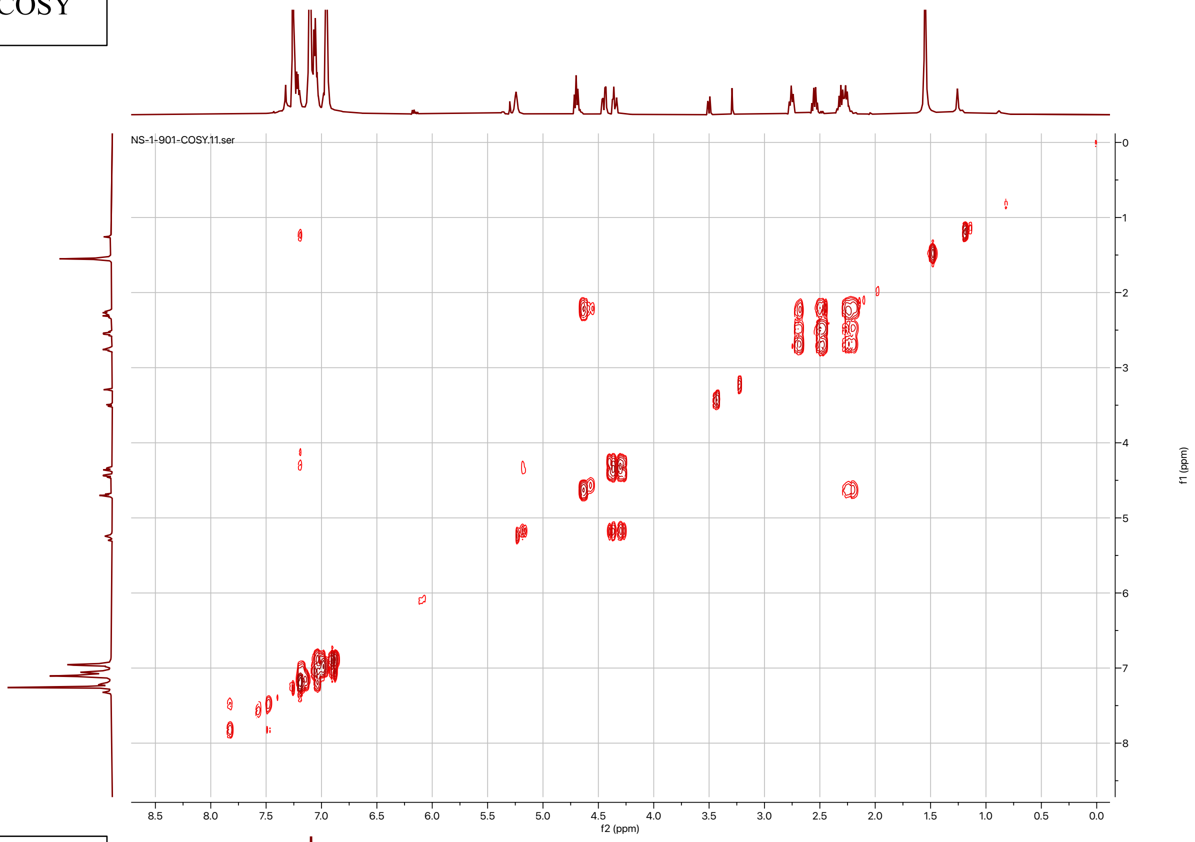
NS-1-901-COSY.11.ser
f2 (ppm)
f1 (ppm)

NOE

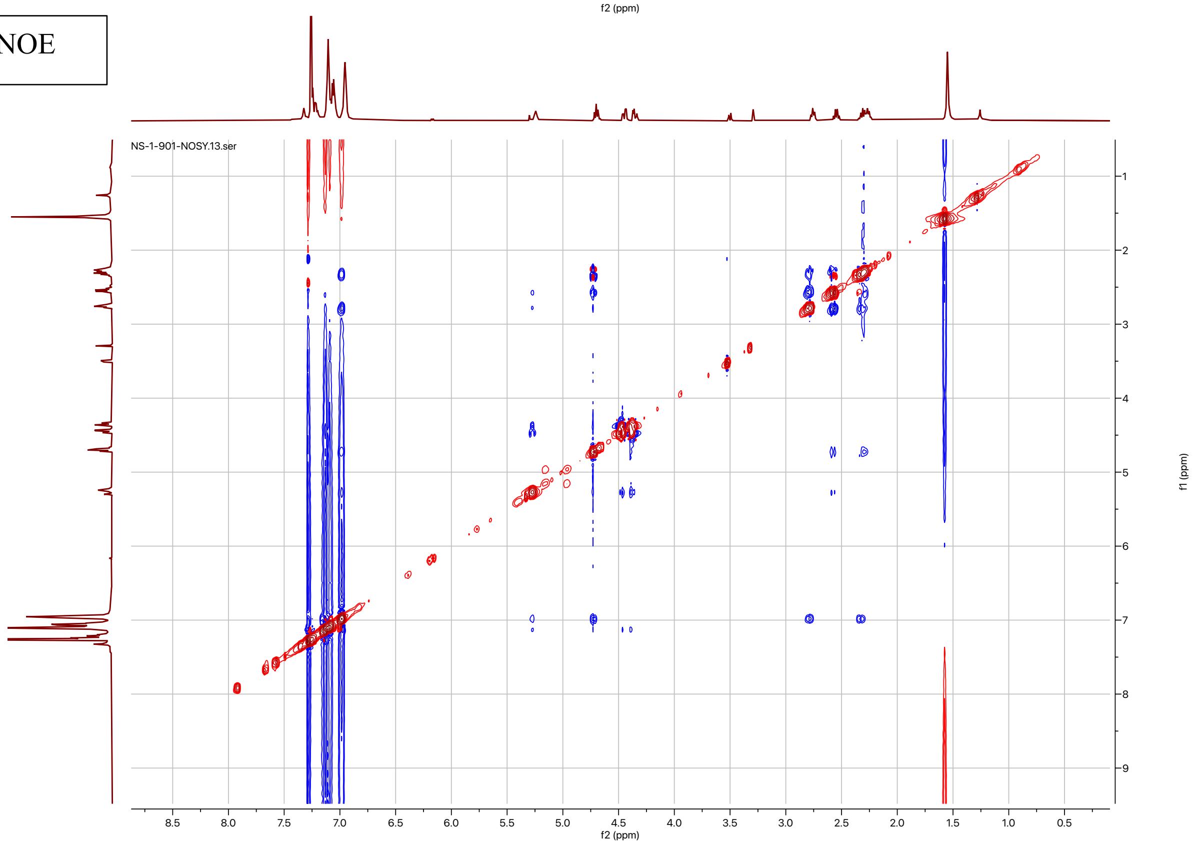
NS-1-901-NOSY.13.ser
f2 (ppm)
f1 (ppm)

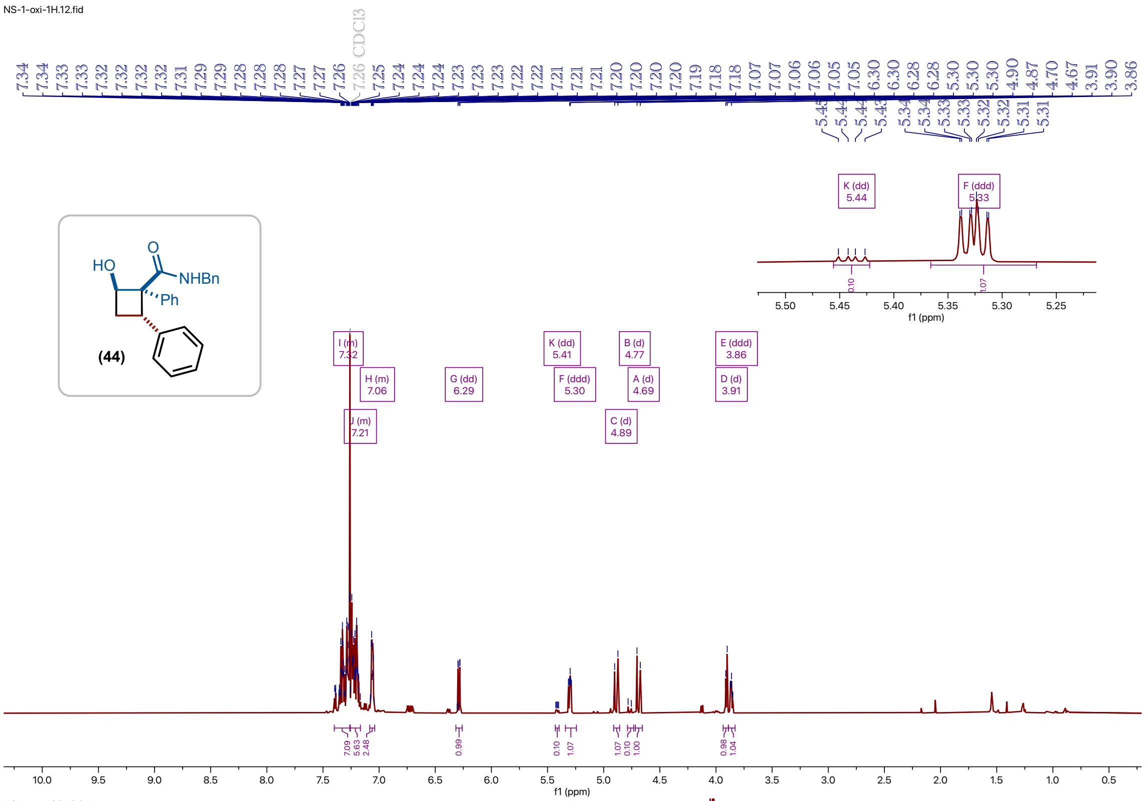
HO
O
NHBn
Ph
(44)
NS-1-oxi-1H.12.fid
CDCl3
f1 (ppm)

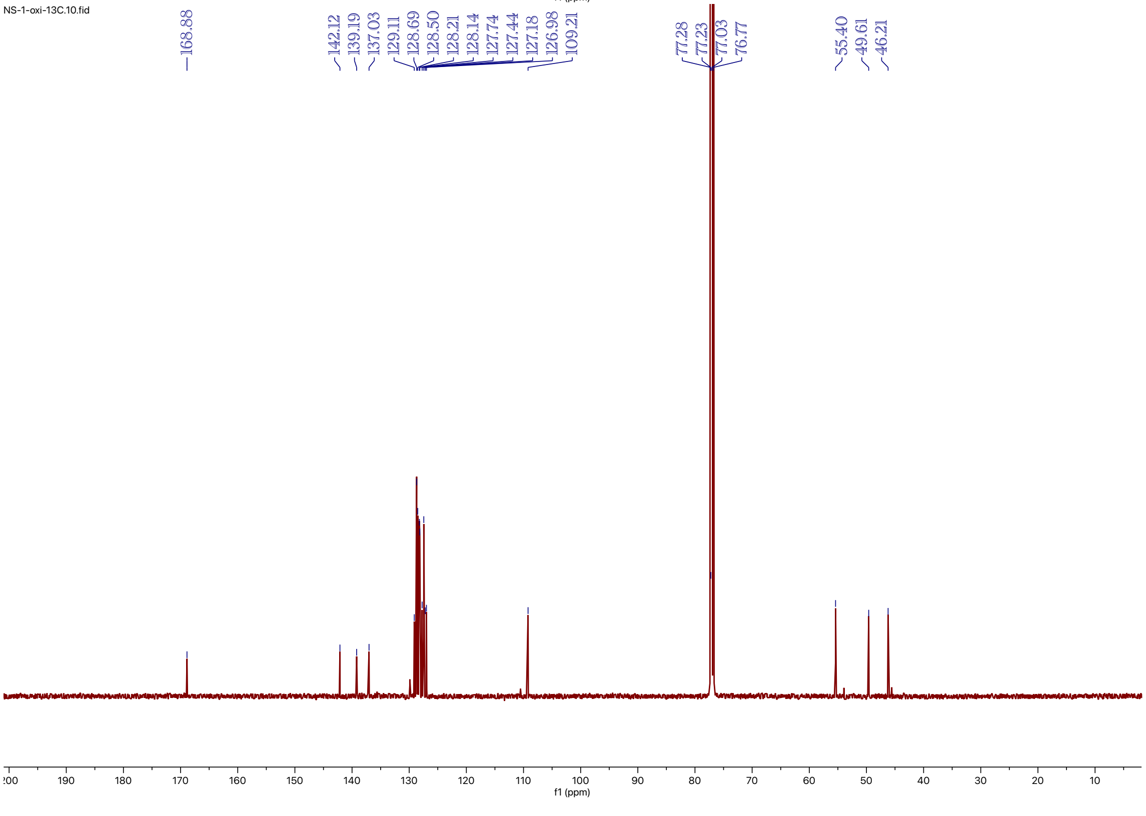
NS-1-oxi-13C.10.fid
168.88
142.12
139.19
137.03
129.11
128.69
128.50
128.21
128.14
127.74
127.44
127.18
126.98
109.21
77.28
77.23
77.03
76.77
55.40
49.61
46.21
f1 (ppm)

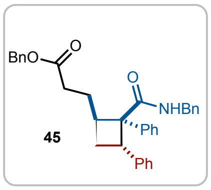
BnO
O
O
NHBn
Ph
Ph

45

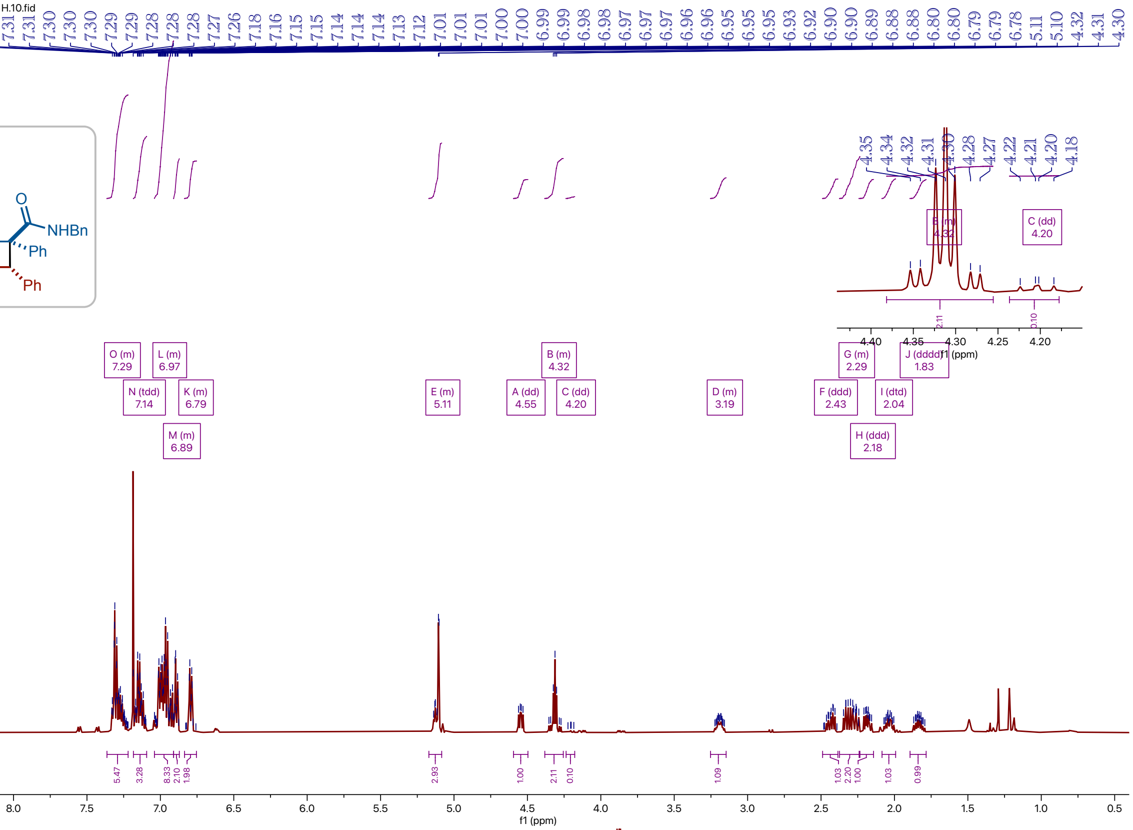
Ns-1-897-1H.10.fid
f1 (ppm)

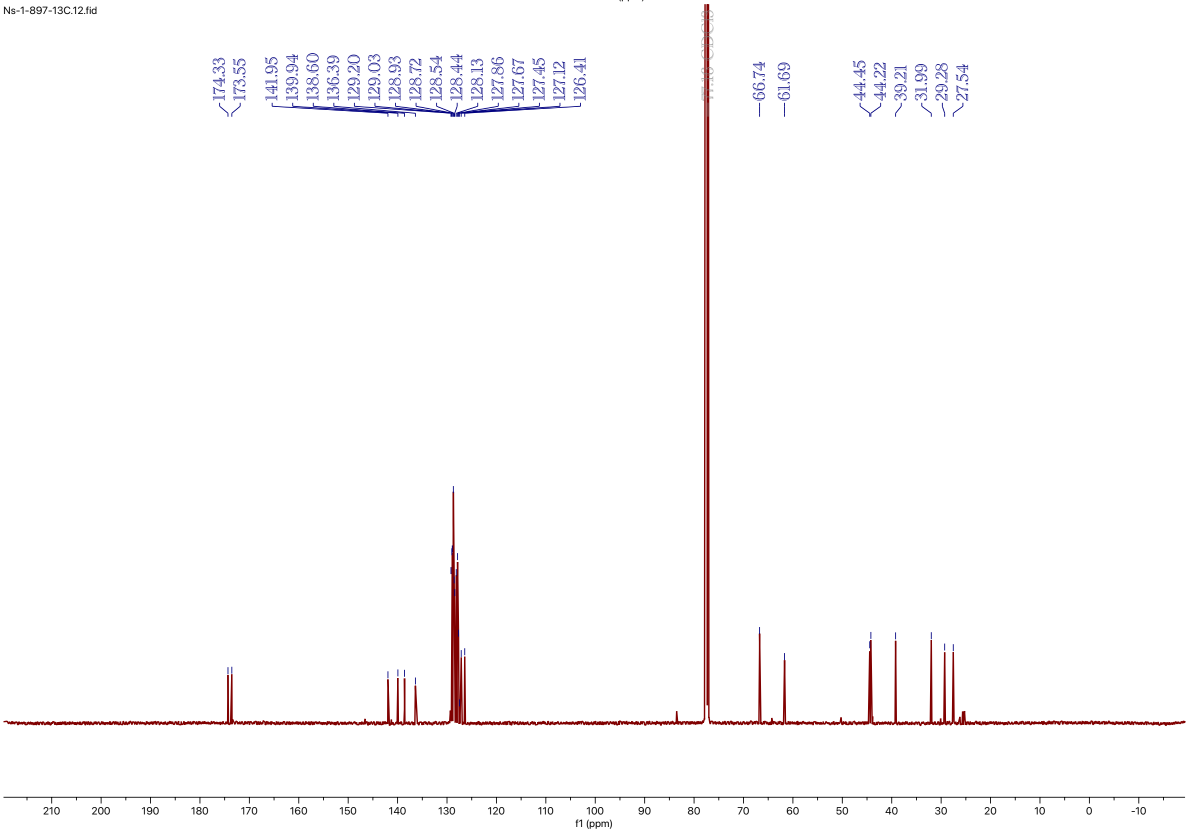
Ns-1-897-13C.12.fid
174.33
173.55
141.95
139.94
138.60
136.39
129.20
129.03
128.93
128.72
128.54
128.44
128.13
127.86
127.67
127.45
127.12
126.41
66.74
61.69
44.45
44.22
39.21
31.99
29.28
27.54
f1 (ppm)

NOE

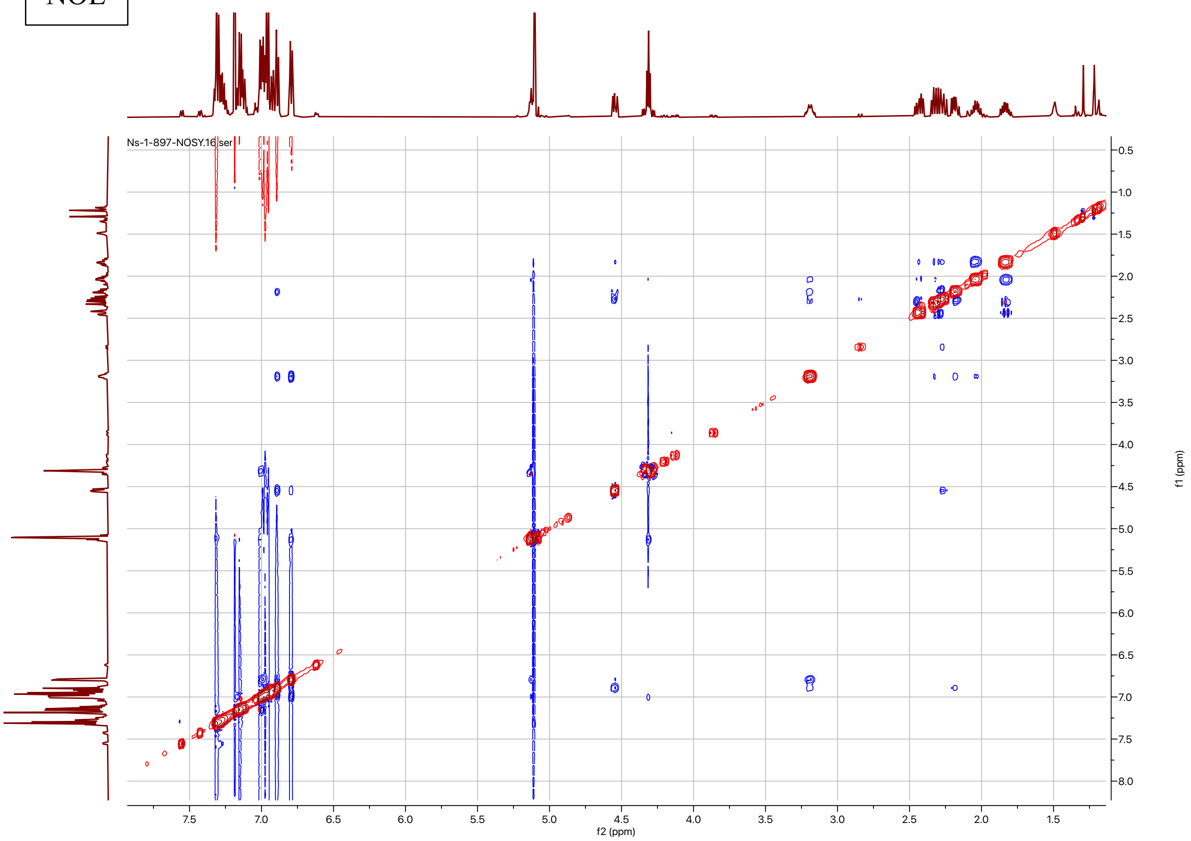
Ns-1-897-NOSY.16.ser
f2 (ppm)
f1 (ppm)

COSY

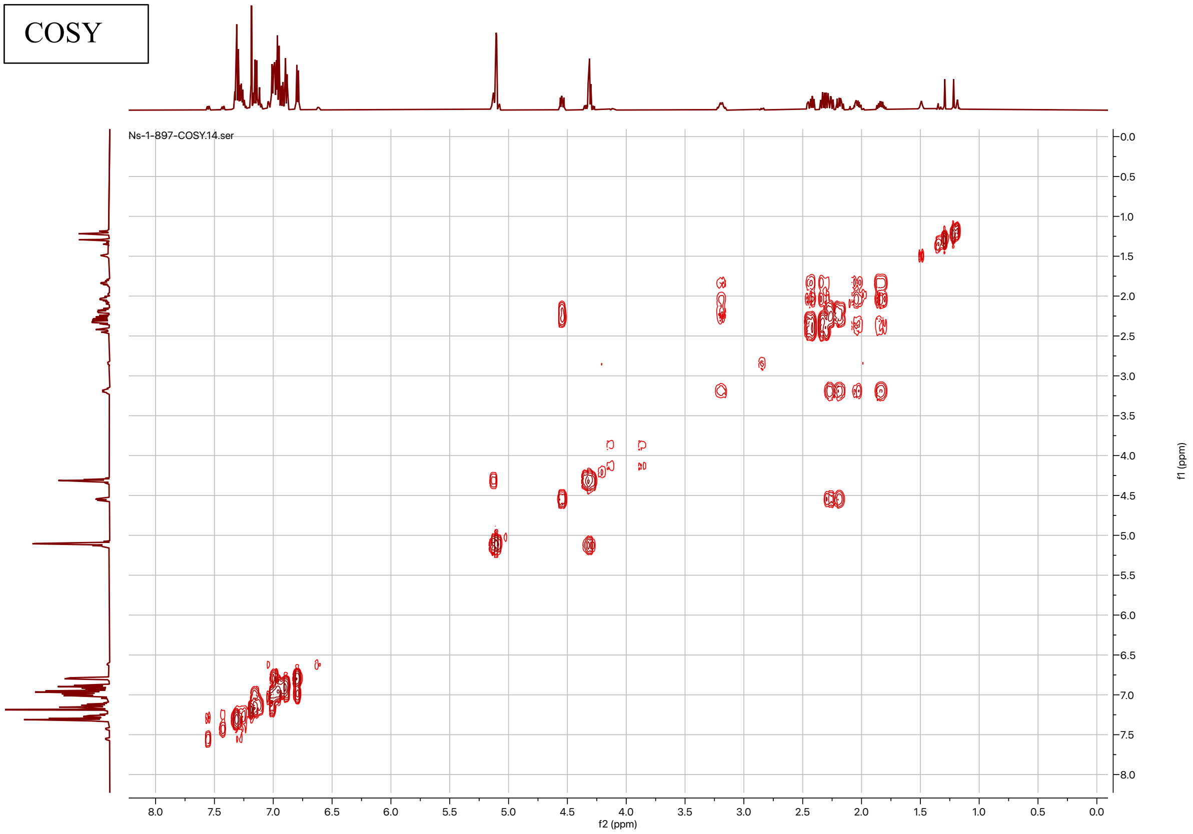
Ns-1-897-COSY.14.ser
f2 (ppm)
f1 (ppm)

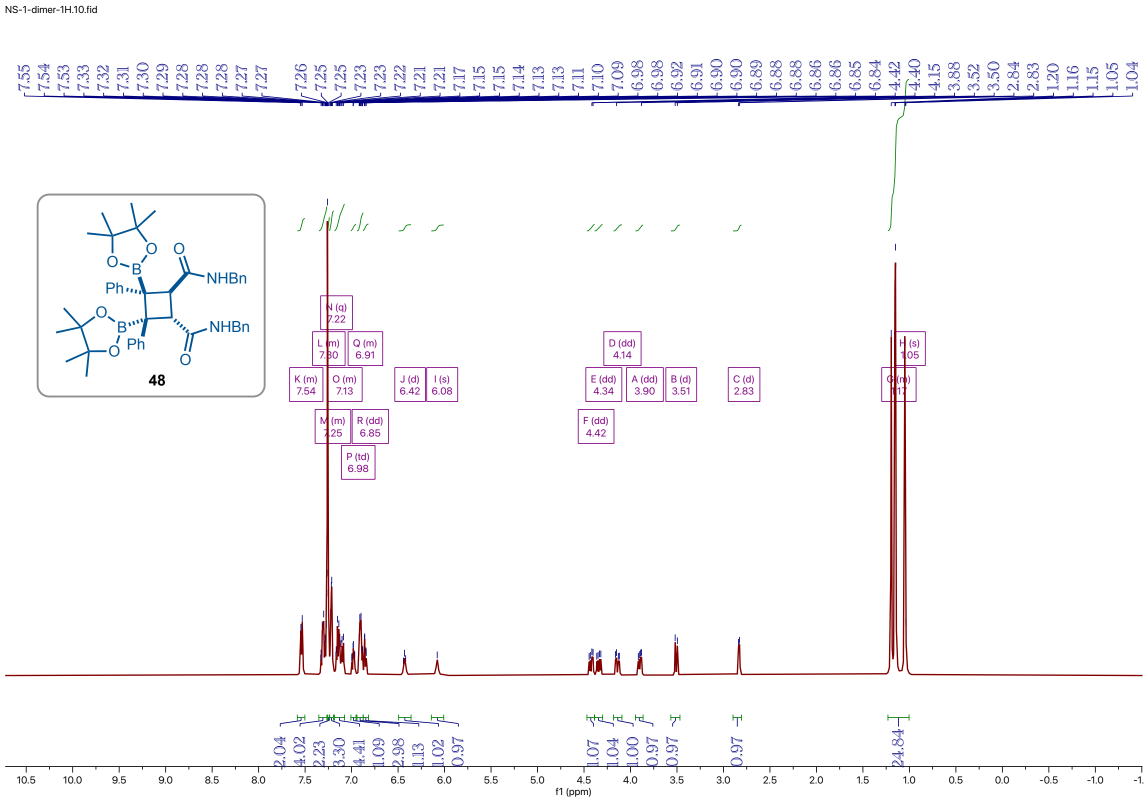
NS-1-dimer-1H.10.fid
48
NHBn
NHBn
Ph
Ph
K (m) 7.54
L (m) 7.30
M (m) 7.25
N (q) 7.22
O (m) 7.13
P (td) 6.98
Q (m) 6.91
R (dd) 6.85
J (d) 6.42
I (s) 6.08
F (dd) 4.42
E (dd) 4.34
D (dd) 4.14
A (dd) 3.90
B (d) 3.51
C (d) 2.83
G (m) 1.17
H (s) 1.05
f1 (ppm)

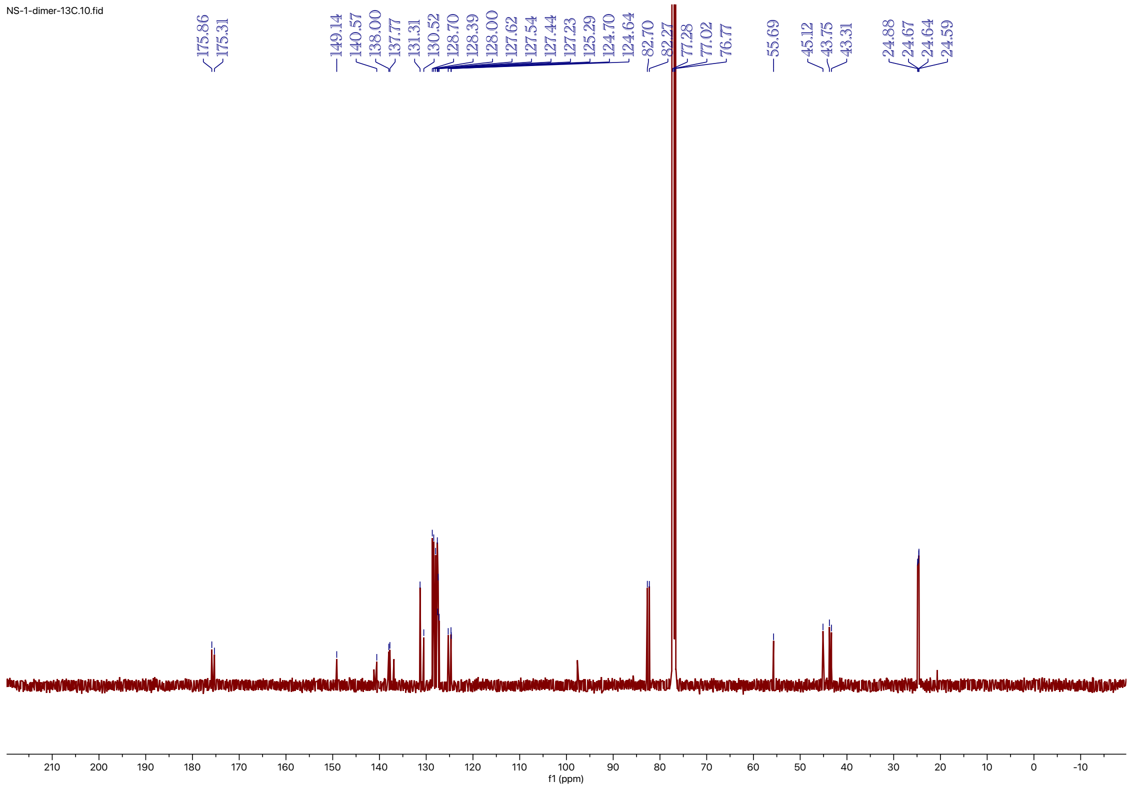
NS-1-dimer-13C.10.fid
175.86
175.31
149.14
140.57
138.00
137.77
131.31
130.52
128.70
128.39
128.00
127.62
127.54
127.44
127.23
125.29
124.70
124.64
82.70
82.27
77.28
77.02
76.77
55.69
45.12
43.75
43.31
24.88
24.67
24.64
24.59
f1 (ppm)

References

[i] C. C. Le, M. K. Wismer, Z.-C. Shi, R. Zhang, D. V. Conway, G. Li, P. Vachal, I. W. Davies, D. W. C. MacMillan, *ACS Cent. Sci.* **2017**, *3*, 647–653.

[ii] P. Garrido-García, I. Quirós, P. Milán-Rois, S. Ortega-Gutiérrez, M. Martín-Fontecha, L. A. Campos, Á. Somoza, I. Fernández, T. Rigotti, M. Tortosa, *Nat. Chem.* **2025**, *17*, 734–745.

[iii] J. Long, L. Wang, X. Liu, J. Liu, S.-P. Luo, X. Fang, *Org. Lett.* **2024**, *26*, 3945–3950.

[iv] Zhang, X.-X.; Xu, S.-T.; Li, X.-T.; Song, T.-T.; Ji, D.-W.; Chen, Q.-A. Dearomative Skeletal Editing of Benzenoids via Diradical. *J. Am. Chem. Soc.* **2025**, *147*, 11533–11542.

[v] P. Lorenz, T. Luchs, A. Hirsch, *Chem. Eur. J.* **2021**, *27*, 4993–5002.

[vi] T. J. O'Connor, F. D. Toste, *ACS Catal.* **2018**, *8*, 5947–5951.

[vii] G. Liu, A. Li, X. Qin, Z. Han, X.-Q. Dong, X. Zhang, *Adv. Synth. Catal.* **2019**, *361*, 2844–2848.

[viii] M. Wienhold, J. J. Molloy, C. G. Daniliuc, R. Gilmour, *Angew. Chem. Int. Ed.* **2021**, *60*, 685–689.

[ix] R. Fritzemeier, A. Gates, X. Guo, Z. Lin, W. L. Santos, *J. Org. Chem.* **2018**, *83*, 10436–10444.

[x] R. G. Fritzemeier, E. J. Medici, C. Szwetkowski, L. G. Wonilowicz, C. D. Sibley, C. Slebodnick, W. L. Santos, *Org. Lett.* **2019**, *21*, 8053–8057.

[xi] T. W. Liwosz, S. R. Chemler, *Chem. Eur. J.* **2013**, *19*, 12771–12777.

[xii] J. M. Posz, N. Sharma, P. A. Royalty, Y. Liu, C. Salome, T. C. Fessard, M. K. Brown, *J. Am. Chem. Soc.* **2024**, *146*, 10142–10149.

[xiii] J. He, Q. Shao, Q. Wu, J.-Q. Yu, *J. Am. Chem. Soc.* **2017**, *139*, 3344–3347.

[xiv] Chen, Y.; May, O.; Blakemore, D. C.; Ley, S. V. *Org. Lett.* **2019**, *21*, 6140–6144.

[xv] Lima, F.; Kabeshov, M. A.; Tran, D. N.; Battilocchio, C.; Sedelmeier, J.; Sedelmeier, G.; Schenkel, B.; Ley, S. V. *Angew. Chem. Int. Ed.* **2016**, *55*, 14085–14089.
