# Supplementary material for: Reenvisioning the De Mayo Reaction: A Boron‐Enabled Cycloaddition Approach
Source: Angew Chem Int Ed Engl. 2026 Jun 3;65(29):e25317. doi: 10.1002/anie.202525317 (PMC13360748; doi:10.1002/anie.202525317)

checkCIF/PLATON report

Structure factors have been supplied for datablock(s) 24066

THIS REPORT IS FOR GUIDANCE ONLY. IF USED AS PART OF A REVIEW PROCEDURE FOR PUBLICATION, IT SHOULD NOT REPLACE THE EXPERTISE OF AN EXPERIENCED CRYSTALLOGRAPHIC REFEREE.

No syntax errors found. CIF dictionary Interpreting this report

Datablock: 24066

Bond precision:	C-C = 0.0030 A		Wavelength=0.71073
Cell:	a=21.8877(18)	b=14.1739(11)	c=8.6957(6)
	alpha=90	beta=90.179(3)	gamma=90
Temperature:	173 K		

	Calculated	Reported
Volume	2697.7(4)	2697.7(4)
Space group	P 21/c	P 21/c
Hall group	-P 2ybc	-P 2ybc
Moiety formula	C31 H33 B N2 O3	C31 H33 B N2 O3
Sum formula	C31 H33 B N2 O3	C31 H33 B N2 O3
Mr	492.40	492.40
Dx,g cm-3	1.212	1.212
Z	4	4
Mu (mm-1)	0.077	0.077
F000	1048.0	1048.0
F000'	1048.43	
h,k,lmax	26,16,10	26,16,10
Nref	4800	4791
Tmin,Tmax	0.990,0.996	0.558,0.745
Tmin'	0.990	

Correction method= # Reported T Limits: Tmin=0.558 Tmax=0.745
AbsCorr = MULTI-SCAN

Data completeness= 0.998 Theta(max)= 25.093

R(reflections)= 0.0509(3733) wR2(reflections)= 0.1481(4791)

S = 1.086 Npar= 338

The following ALERTS were generated. Each ALERT has the format
test-name_ALERT_alert-type_alert-level.
Click on the hyperlinks for more details of the test.

Alert level C

```
PLAT905_ALERT_3_C Negative K value in the Analysis of Variance ...     -0.041 Report
PLAT911_ALERT_3_C Missing FCF Refl Between Thmin & STh/L=    0.597          6 Report
                0  0  2, -16  7  7,  -4  8  9,   9  6  9,  -7  0 10,   3  4 10,
```

Alert level G

```
PLAT007_ALERT_5_G Number of Unrefined Donor-H Atoms .............          1 Report
              H1
PLAT395_ALERT_2_G Deviating  X-O-Y   Angle From 120 for O2       .      108.1 Degree
PLAT395_ALERT_2_G Deviating  X-O-Y   Angle From 120 for O3       .      107.3 Degree
PLAT909_ALERT_3_G Percentage of I>2sig(I) Data at Theta(Max) Still        63% Note
PLAT910_ALERT_3_G Missing # of FCF Reflection(s) Below Theta(Min).          3 Note
                1  0  0,   1  1  0,   2  0  0,
PLAT913_ALERT_3_G Missing # of Very Strong Reflections in FCF ....          1 Note
                0  0  2,
PLAT969_ALERT_5_G The 'Henn et al.' R-Factor-gap value ..........       3.10 Note
              Predicted wR2: Based on SigI**2  4.78 or SHELX Weight 14.14
PLAT978_ALERT_2_G Number C-C Bonds with Positive Residual Density.          2 Info
```

0 **ALERT level A** = Most likely a serious problem - resolve or explain
0 **ALERT level B** = A potentially serious problem, consider carefully
2 **ALERT level C** = Check. Ensure it is not caused by an omission or oversight
8 **ALERT level G** = General information/check it is not something unexpected

0 ALERT type 1 CIF construction/syntax error, inconsistent or missing data
3 ALERT type 2 Indicator that the structure model may be wrong or deficient
5 ALERT type 3 Indicator that the structure quality may be low
0 ALERT type 4 Improvement, methodology, query or suggestion
2 ALERT type 5 Informative message, check

It is advisable to attempt to resolve as many as possible of the alerts in all categories. Often the minor alerts point to easily fixed oversights, errors and omissions in your CIF or refinement strategy, so attention to these fine details can be worthwhile. In order to resolve some of the more serious problems it may be necessary to carry out additional measurements or structure refinements. However, the purpose of your study may justify the reported deviations and the more serious of these should normally be commented upon in the discussion or experimental section of a paper or in the "special_details" fields of the CIF. checkCIF was carefully designed to identify outliers and unusual parameters, but every test has its limitations and alerts that are not important in a particular case may appear. Conversely, the absence of alerts does not guarantee there are no aspects of the results needing attention. It is up to the individual to critically assess their own results and, if necessary, seek expert advice.

Publication of your CIF in IUCr journals

A basic structural check has been run on your CIF. These basic checks will be run on all CIFs submitted for publication in IUCr journals (*Acta Crystallographica*, *Journal of Applied Crystallography*, *Journal of Synchrotron Radiation*); however, if you intend to submit to *Acta Crystallographica Section C* or *E* or *IUCrData*, you should make sure that full publication checks are run on the final version of your CIF prior to submission.

Publication of your CIF in other journals

Please refer to the *Notes for Authors* of the relevant journal for any special instructions relating to CIF submission.

PLATON version of 06/01/2024; check.def file version of 05/01/2024

Datablock 24066 - ellipsoid plot

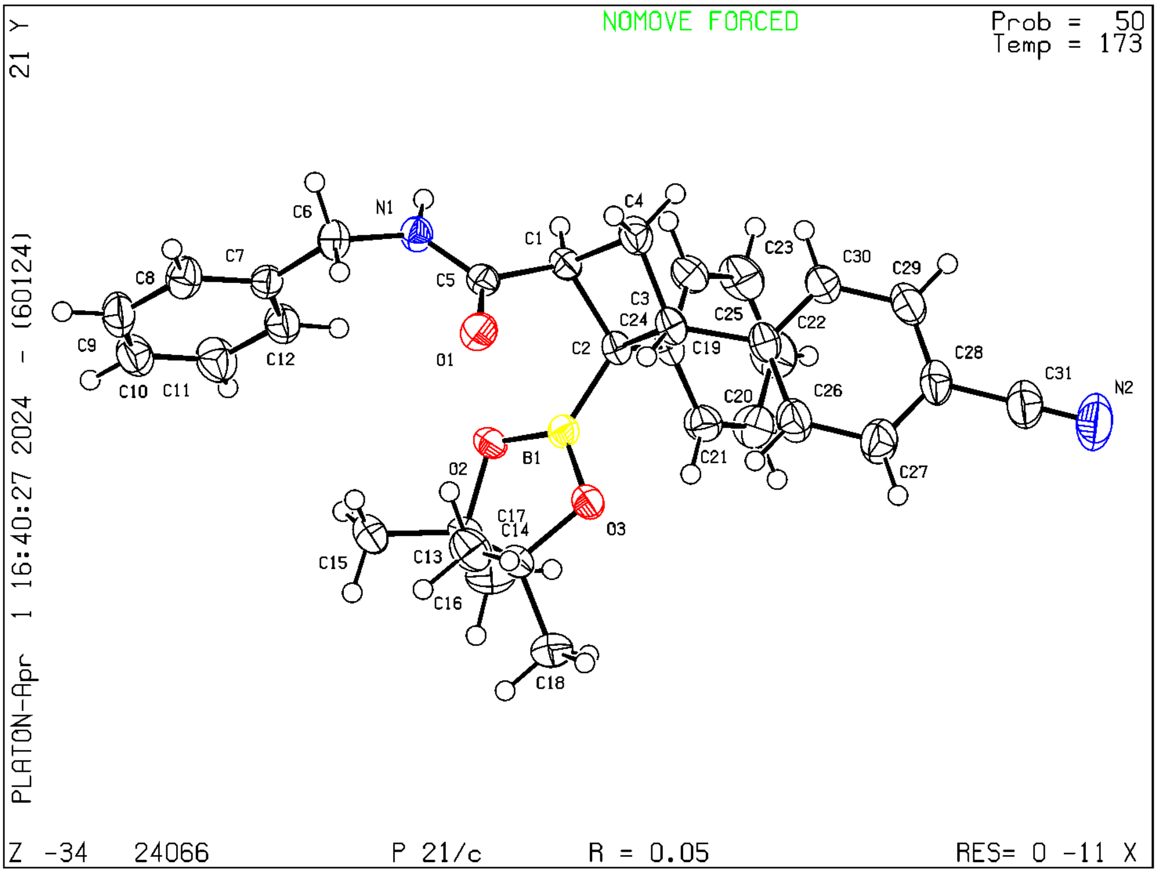

Supplement: Supplementary file 2 — Supporting File 2: anie72919‐sup‐0002‐DataFile.zip. [file ANIE-65-e25317-s002.zip › 24066_Cifcheck.pdf]
